# Association between Frailty, Osteoporosis, Falls and Hip Fractures among Community-Dwelling People Aged 50 Years and Older in Taiwan: Results from I-Lan Longitudinal Aging Study

**DOI:** 10.1371/journal.pone.0136968

**Published:** 2015-09-08

**Authors:** Li-Kuo Liu, Wei-Ju Lee, Liang-Yu Chen, An-Chun Hwang, Ming-Hsien Lin, Li-Ning Peng, Liang-Kung Chen

**Affiliations:** 1 Aging and Health Research Center, National Yang Ming University, Taipei, Taiwan; 2 Institute of Public Health, National Yang Ming University, Taipei, Taiwan; 3 Center for Geriatrics and Gerontology, Taipei Veterans General Hospital, Taipei, Taiwan; 4 Department of Family Medicine, Taipei Veterans General Hospital Yuanshan Branch, I-Lan County, Taiwan; Harvard Medical School, UNITED STATES

## Abstract

**Background:**

Association of frailty with adverse clinical outcomes has been reported in Western countries, but data from the Asian population are scarce. This study aimed to evaluate the epidemiology of frailty among community-dwelling middle-aged and elderly population and to explore its association with musculoskeletal health in Taiwan.

**Methods:**

I-Lan Longitudinal Aging Study (ILAS) data were retrieved for this study. Frailty was defined by the Fried’s criteria; a comparison of demographic characteristics, physical performance, and body composition, including skeletal muscle mass and bone mineral density (BMD), as well as recent falls, history of hip fractures and the functional status of subjects with different frailty statuses were accomplished.

**Results:**

Overall, the data of 1,839 participants (mean age: 63.9±9.3 years, male 47.5%) were obtained for analysis. The prevalence of pre-frailty was 42.3% in men and 38.8% in women, whereas the prevalence of frailty was 6.9% and 6.7% in men and women, respectively. Frailty was significantly associated with older age, the male gender, larger waist circumference, lower skeletal muscle index, lower hip BMD, poorer physical function, poorer nutritional status, and poorer cognitive function. Also, frailty was significantly associated with osteoporosis (OR: 7.73, 95% CI: 5.01–11.90, p<0.001), history of hip fractures (OR: 8.66, 95% CI: 2.47–30.40, p = 0.001), and recent falls (O.R: 2.53, 95% CI: 1.35–4.76, p = 0.004).

**Conclusions:**

Frailty and pre-frailty, in Taiwan, was closely associated with recent falls, history of hip fractures and osteoporosis among community-dwelling people 50 years of age and older. Furthermore, frailty intervention programs should take an integrated approach towards strengthening both and muscle mass, as well as prevention of falls.

## Introduction

Frailty is a well-recognized geriatric syndrome,[1] which features the loss of function, loss of physiologic reserve, and an increased vulnerability to diseases and death.[2] In addition, frailty is also associated with cognitive impairment,[3] multimorbidity, impaired functional status,[4] risk of falls and fractures,[5] medical and surgical outcomes,[6,7] hospitalizations, institutionalization and mortality.[8] Moreover, frailty is closely associated with body compositional changes and osteoporosis,[9] and may overlap with the pathogenesis of sarcopenia.[10] Despite extensive reports regarding frailty and related adverse health outcomes, the association of frailty and the changes of body composition has not been well understood.[11]

The prevalence of frailty varies greatly from study to study in its use of different diagnostic criteria in different settings.[2,12,13] Although the epidemiology may vary greatly, the age-related increasing trend of frailty prevalence has been clearly shown indifferent studies. As one of the fastest aging countries in the world, Taiwan needs to face the challenges related to population aging as most Western countries are doing.[14,15] Among all health care challenges, the impact of frailty to health and health care outcomes is of great importance. Previous studies have disclosed that frailty was associated with the decline in lean muscle mass, bone mass and the presence of sarcopenia,[16–18] which may result in a greater negative impact on older people. Although these associations have been reported in previous studies, little is known regarding the association among Asian populations. Therefore, this study aimed to evaluate the prevalence and clinical characteristics of frailty among the community-dwelling middle aged and elderly population in Taiwan, and to explore the associations of frailty and musculoskeletal health.

## Materials and Methods

### Study subjects

The I-Lan Longitudinal Aging Study (ILAS) is a community-based aging cohort study in I-Lan County of Taiwan, which aimed to evaluate the complex interrelationship between aging, frailty, sarcopenia and cognitive decline. Community-dwelling people aged 50 years and older were randomly selected for study from the I-Lan County of Taiwan.[19] Selected inhabitants were invited via mail or telephone to participate with the research team, and were enrolled when they signed the consent forms as study participations. The inclusion criteria for ILAS were: (1) inhabitants who presently live in I-Lan County without a plan of moving in the near future, and (2) residents 50 years of age or older. Subjects with the following conditions were excluded: (1) those who were unable to adequately communicate with the research nurses, (2) those unable to complete all evaluation tests due to poor functional status, (3) those who had a limited life expectancy due to major illnesses, and (4) current residents in long-term care facilities. Overall, the data of 1,839 participants of ILAS were retrieved for study. All participants signed a written informed consent. The whole study and the consent procedure had been approved by the Institutional Review Board of National Yang Ming University.

### Demography, physical examinations and laboratory examinations

A questionnaire consisting of demographic information, socioeconomic condition, medical history and the burden of chronic diseases was evaluated using Charlson’s Comorbidity Index.[20] Tobacco usage was categorized into three classes: non-smoker, ex-smoker (quit in past 6 months) and current smoker. Participants who consumed alcohol were categorized as drinkers and non-drinkers. A comprehensive functional assessment was performed on all participants by using the following: the Functional Autonomy Measurement System for physical function test,[21] the Center for Epidemiologic Studies Depression Scale (CES-D) for measuring the mood status,[22] the Mini-Nutrition Assessment (MNA) for nutritional status measurement,[23] and the Mini–Mental State Examination (MMSE) for cognitive function measurement.[24]

All subjects underwent anthropometric measurements by research nurses, including height and body weight, and the body mass index (BMI) was calculated accordingly. Baseline blood samples were obtained for each participant in the morning after an overnight fasting of at least 10 hours. Serum levels of albumin and total cholesterol were measured using an automatic analyzer (ADVIA 1800, Siemens, Malvern, PA, USA). Whole-blood glycated hemoglobin A1c (HbA1c) was measured by an enzymatic method using the Tosoh G8 HPLC Analyzer (Tosoh Bioscience, Inc., San Francisco, CA, USA). Serum levels of intact-parathyroid hormone (i-PTH) (Siemens Advia Centaur) and 25-hydroxyvitamin D (25(OH)D) (Diasorin Liaison) were also measured by ELISA methods. High-sensitivity C-reactive protein (hs-CRP) was determined by an immunoturbidimetric assay (Siemens Advia 1800) for further analysis.

### Muscle strength and physical performance

For all participants, handgrip strength of the dominant hand was measured using digital dynamometers (Smedlay’s Dynamo Meter; TTM, Tokyo, Japan), with participants standing in an upright position with both arms down on their sides. The best results of three tests were used for further analysis. Moreover, participants performed a timed 6-meter walk for each participant to evaluate their physical performance.

### Bone mineral density (BMD) and body composition

A whole body dual-energy X-ray absorptiometry (DXA) scan was performed on each participant to measure their total body fat mass and fat-free lean body mass (LBM) by using a Lunar Prodigy instrument (GE Healthcare, Madison, WI, USA). Appendicular skeletal muscle mass (ASM) was calculated as the sum of the lean soft tissue mass of all four limbs. In this study, height-adjusted muscle index, or relative appendicular skeletal muscle (RASM),[25] was calculated by appendicular skeletal muscle mass divided by height (m) square (ASM/height^2^, kg/m^2^). BMD at the lumbar spine and bilateral hip joints were measured for analysis.

### Definition of Frailty

In this study, frailty is defined by Fried’s criteria, which includes exhaustion, weakness, slowness, physical inactivity and weight loss.[26] Exhaustion was defined using the 2 statements by the Center for Epidemiologic Studies-Depression scale (CES-D). Weakness was defined by low handgrip strength, and slowness was defined by slow gait speed. Physical inactivity was evaluated by using the International Physical Activity Questionnaire (IPAQ).[27,28] Weight loss was defined as having involuntary weight loss of >5% in the past year or 3kgs within past 3 months. Weakness, slowness and physical inactivity referred to those who performed lower than the gender-specific lowest quintile of the study population. A participant was classified as frail if he/she was positive for three of more items on the Fried’s criteria, and those who were positive for one or two items were classified as pre-frail. Those who were negative on all 5 items of Fried’s criteria were considered robust.

### Statistical analysis

In this study, continuous variables were expressed as the mean ± standard deviation, and the categorical data was expressed by percentages. Comparisons of continuous data between groups were done by Student’s t test and comparisons of categorical data were done by Chi square test when appropriate. Comparisons between groups of different frailty statuses were performed by one-way ANOVA. To study the cross-sectional association between bone health, muscle quality and the frailty syndrome, multinomial logistic regression was used, allowing the modeling of the prefrail and frail states by using robust as reference group. Further gender-specific analysis was also performed for the above-mentioned conditions.

The covariates of interest, waist circumference, muscle index, bone mineral density were also analyzed. Other covariates included age, gender, functional status, cognition status, nutrition, and comorbid conditions. Finally, serum 25(OH)D and i-PTH level were added to the model because they were closely related to bone mineral density and fall.

The first sequential model included basic characteristics, bone density and muscle quality. The second model added functional confounders, and the serum markers related to bone and fall were added to the third model.

## Results

Overall, data of 1,839 participants 50 years of age and older (mean age: 63.9±9.3 years, 47.5% males) from ILAS were retrieved for study. Table 1 summarized the comparisons of demographic characteristics of study participants between genders. In this study, BMI was similar between men and women, but men had significantly higher lean body mass, appendicular skeletal muscle mass, and skeletal muscle index (RASM) than women. In contrast, the women had higher total body fat percentage and more total body fat mass than men. Also, men had significantly stronger handgrip strength (35.1±8.3 Kg vs. 21.8±5.4 Kg, P<0.001), and faster gait speed (1.6±0.5 vs. 1.4±0.4 m/s, P<0.001) than women. Moreover, men also had significantly higher bone mineral density in both their lumbar spine and femoral neck (Table 1).

**Table 1:** Demographic characteristics of participants of the I-Lan Longitudinal Aging Study.

	Total (N = 1839)	Men (N = 873)	Women (N = 966)	P value
**Age (years)**	63.9±9.3	65.1±9.7	62.9±8.7	<0.001
**Anthropometric measurements**				
Height (cm)	158.6±8.0	164.3±6.2	153.5±5.6	<0.001
Weight (Kg)	62.7±11.0	67.4±10.5	58.4±9.6	<0.001
Body mass index (Kg/m^2^)	24.9±3.6	24.9±3.3	24.8±3.8	0.433
**Dual-energy X-ray absorptiometry**					
Lean body mass (Kg)	41.7±8.2	48.6±5.6	35.5±4.3	<0.001
ASM (Kg)	17.9±4.1	21.4±3.0	14.8±2.0	<0.001
RASM (kg/m^2^)	7.0±1.1	7.9±0.8	6.3±0.7	<0.001
Total fat mass (Kg)	19.5±7.0	17.2±6.8	21.6±6.6	<0.001
Total body fat percentage (%)	31.6±8.8	25.3±6.6	37.1±6.4	<0.001
Lumbar BMD	1.030±0.182	1.091±0.176	0.976±0.169	<0.001
Hip BMD	0.839±0.140	0.890±0.132	0.793±0.130	<0.001
**Physical performance**				
Walking speed (m/s)	1.5±0.5	1.6±0.5	1.4±0.4	<0.001
Handgrip strength (Kg)	28.1±9.6	35.1±8.3	21.8±5.4	<0.001
**Frailty status (%)**				
Robust	52.7	50.9	54.5	0.289
Pre-frail	40.5	42.3	38.8	
Frail	6.8	6.9	6.7	
**Cigarette Smoking (%)**				
Never smoker	69.5	40.2	96.0	<0.001
Ex-smoker	12.2	24.7	0.9	
Current smoker	18.3	35.1	3.1	
**Alcohol drinker (%)**	33.0	49.8	17.8	<0.001
**Functional status**				
SMAF	-0.18±1.63	-0.20±1.79	-0.16±1.47	0.575
CES-D	2.4±4.6	2.0±3.7	2.8±5.2	<0.001
Mini-nutrition assessment	27.2±1.8	27.4±1.7	26.9±1.9	<0.001
MMSE	25.6±4.0	26.2±3.5	25.1±4.4	<0.001
Education(years)	6.2±5.0	7.1±5.0	5.4±4.8	<0.001

ASM = appendicular skeletal muscle mass; RASM = relative appendicular skeletal muscle; BMD = bone mineral density; SMAF = the Functional Autonomy Measurement System; CES-D = the Center for Epidemiologic Studies Depression Scale; MMSE = Mini–mental state examination.

Table 2 summarized the comparisons of clinical characteristics between subjects in different frailty statuses. The prevalence of pre-frailty was 42.3% in men and 38.8% in women, while frailty was 6.9% and 6.7% in men and women, respectively. Overall, frail subjects were significantly older but pre-frail and frail participants had higher waist circumference than those robust subjects, although the BMI did not differ significantly between them. Also, smoking was not significantly different between frailty groups but frail people were less likely to consume alcohol habitually.

**Table 2:** Baseline association of demographic and health characteristics with frailty: the *I-Lan* Longitudinal Aging Study.

	Total	Robust	Pre-frail	Frail	p
**Number (%)**	1839	970 (52.7)	744 (40.5)	125 (6.8)	
**Age (year)**	63.9±9.3	60.7±7.5	66.3±9.3	74.6±9.2	<0.001
**Sex (%)**					
**Women**	52.5	54.2	50.4	52.0	0.289
**Men**	47.5	45.8	49.6	48.0	
**BMI (Kg/m^2^)**	24.9±3.6	24.8±3.5	24.9±3.6	24.7±4.0	0.655
**Waist circumference (cm)**	84.8±9.8	83.8±9.4	85.5±9.9	88.1±10.8	<0.001
**Cigarette Smoking (%)**					
Never smoker	67.4	69.3	65.4	60.9	0.359
Ex-smoker	19.7	18.5	21.4	20.3	
Current smoker	12.9	12.2	13.2	18.8	
**Alcohol drinker (%)**	35.0	38.7	32.1	15.4	<0.001
**Dual-energy X-ray absorptiometry**				
Lean body mass (Kg)	41.7±8.2	42.3±8.4	41.2±8.0	39.7±7.0	0.001
ASM (Kg)	17.9±4.1	18.3±4.3	17.6±3.9	16.5±3.6	<0.001
RASM (Kg/m^2^)	7.0±1.1	7.1±1.1	7.0±1.1	6.7±1.0	<0.001
Total fat mass (Kg)	19.5±7.0	19.7±7.1	19.4±6.9	18.9±7.8	0.463
Total body fat percentage (%)	31.6±8.8	31.5±8.7	31.7±8.6	31.5±10.0	0.917
**Physical performance**					
Walking speed (m/s)	1.5±0.5	1.7±0.4	1.4±0.4	0.9±0.3	<0.001
Handgrip strength (Kg)	28.1±9.6	30.9±9.0	26.0±9.2	18.8±7.1	<0.001
**Bone mineralization**					
Lumbar BMD	1.0±0.2	1.0±0.2	1.0±0.2	1.0±0.2	<0.001
Hip BMD	0.8±0.1	0.9±0.1	0.8±0.1	0.7±0.1	<0.001
25(OH)D (ng/ml)	23.4±7.1	23.1±6.5	23.9±7.8	22.7±6.8	0.040
i-PTH (pg/ml)	43.7±43.9	42.4±30.0	44.6±58.4	48.2±32.5	0.283
**Osteoporosis (%)**	15.3	8.8	19.7	42.6	<0.001
**Men**	8.4	3.7	10.8	29.8	<0.001
**Women**	21.4	13.0	28.1	55.2	<0.001
**Hip surgery (%)**	1.2	0.5	1.7	4.3	0.001
**Falls within 3 months (%)**					
0	94.8	95.3	95.3	88.8	0.001
1	4.6	4.3	4.3	8.0	
≥ 2	0.6	0.4	0.4	3.2	
**Functional status**					
SMAF	-0.2±1.6	-0.0±0.2	-0.1±0.5	-2.0±5.8	<0.001
CES-D	2.4±4.6	1.5±2.6	2.7±4.4	8.2±10.0	<0.001
Mini-nutrition assessment	27.2±1.8	27.5±1.6	27.1±1.8	25.3±2.6	<0.001
MMSE	25.6±4.0	26.8±3.0	25.0±4.0	20.8±5.8	<0.001
Education, years	6.2±5.0	7.5±4.8	5.2±4.8	2.6±3.5	<0.001
CCI	1.0±1.3	0.7±1.1	1.2±1.3	2.1±1.4	<0.001
**Serum markers of protein-energy nutrition**					
Total lymphocyte count (K/uL)	2.0±0.6	2.0±0.6	1.9±0.7	1.8±0.6	0.020
Albumin (mg/dL)	4.5±0.2	4.5±0.2	4.5±0.3	4.4±0.2	<0.001
Total cholesterol (mg/dL)	195.0±35.3	198.8±35.1	191.8±34.8	184.7±35.8	<0.001
HbA1c (%)	6.1±1.0	6.0±0.8	6.2±1.1	6.4±1.3	<0.001
hs-CRP (mg/dL)	0.2±0.5	0.2±0.4	0.2±0.4	0.4±0.9	<0.001

ASM = appendicular skeletal muscle mass; RASM = relative appendicular skeletal muscle; BMD = bone mineral density; 25(OH)D = 25-hydroxyvitamin D; i-PTH = intact-parathyroid hormone; SMAF = the Functional Autonomy Measurement System; CES-D = the Center for Epidemiologic Studies Depression Scale; MMSE = Mini–mental state examination; CCI = Charlson’s Comorbidity Index; hs-CRP = high-sensitive C-reactive protein.

In the body composition analysis, frail people had significantly lower lean body mass, appendicular skeletal muscle mass, RASM and BMD when compared with other groups. However, the serum levels of total 25-OH vitamin D were similar in robust and pre-frail groups, but significantly lower in the frail group. The serum levels of i-PTH were similar between subjects with different frailty statuses. Comparisons of functional status, depressive symptoms, nutritional status and cognitive function showed a declining trend between different frailty statuses. Also, frail people had the highest CCI scores, followed by pre-frail and robust subjects, which was statistically significant. Comparisons of serum markers for protein-energy nutrition such as albumin and total cholesterol and total lymphocyte counts showed no statistical differences between subjects with different frailty statuses.

Table 3 showed the odds ratios for frailty status in association with poor medical conditions. Frailty was significantly associated with osteoporosis (OR: 7.73, 95% CI: 5.01–11.90, p<0.001), history of hip fractures (OR: 8.66, 95% CI: 2.47–30.40, p = 0.001), and recent falls (O.R: 2.53, 95% CI: 1.35–4.76, p = 0.004). Gender differences were only found in the association between osteoporosis and frailty status. In women, worse frail conditions were found to be at a higher risk of osteoporosis. The odds ratio was 2.62 in the prefrail group and 8.25 in the frail group of women compared with their robust college. Robust and prefrail men had a lower risk of osteoporosis compared with robust women, but the odds ratio increased to 2.85 when it came to frail men.

**Table 3:** Odds ratios for frailty status in association with poor medical conditions.

		Odds Ratio	95% CI		p value
	Participant number/Total number		Lower	Upper	
**Osteoporosis**					
**Total**					
robust	84/958	Reference			
prefrail	142/722	2.55	1.91	3.40	<0.001
frail	49/115	7.73	5.01	11.90	<0.001
**Women**					
robust	68/524	Reference			
prefrail	104/370	2.62	1.86	3.69	<0.001
frail	32/58	8.25	4.64	14.69	<0.001
**Men**					
robust	16/434	0.26	0.15	0.45	<0.001
prefrail	38/352	0.81	0.53	1.24	0.332
frail	17/57	2.85	1.53	5.31	0.001
**Hip surgery**					
**Total**					
robust	5/958	Reference			
prefrail	12/722	3.22	1.13	9.19	0.029
frail	5/115	8.66	2.47	30.40	0.001
**Women**					
robust	4/524	Reference			
prefrail	5/370	1.78	0.48	6.68	0.392
frail	2/58	4.64	0.83	25.92	0.080
**Men**					
robust	1/434	0.30	0.03	2.70	0.283
prefrail	7/352	2.64	0.77	9.08	0.124
frail	3/57	7.22	1.58	33.12	0.011
**Fall**					
**Total**					
robust	46/970	Reference			
prefrail	35/744	0.99	0.63	1.56	0.971
frail	14/125	2.53	1.35	4.76	0.004
**Women**					
robust	29/526	Reference			
prefrail	20/375	0.97	0.54	1.73	0.907
frail	7/65	2.07	0.87	4.93	0.101
**Men**					
robust	17/444	0.68	0.37	1.26	0.221
prefrail	15/369	0.73	0.38	1.38	0.326
frail	7/60	2.26	0.95	5.42	0.067

In multinomial logistic regression analysis, we found that older, male, with larger waist circumference, lower muscle mass index, lower hip BMD, lower SMAF scores (poorer functional status), lower MNA score (higher malnutrition or undernutrition risk), and lower MMSE score (poorer cognition) were all independent risk factors for pre-frailty and frailty (Table 4).

**Table 4:** Association between frailty, physical performance and body composition as described by Odds Ratios (ORs) for multinomial logistic regression models.

	Model 1^a^		Model 2^b^		Model 3^c^	
	Pre-frail	Frail	Pre-frail	Frail	Pre-frail	Frail
	versus Robust		versus Robust		versus Robust	
Characteristic	OR (95% confidence interval)					
Age(years)	1.065(1.051, 1.079)^†^	1.142(1.109,1.176)^†^	1.043(1.027, 1.060)^†^	1.075(1.038, 1.114)^†^	1.043(1.026, 1.060)^†^	1.077(1.039, 1.116)^†^
Gender						
Women	Reference	Reference	Reference	Reference	Reference	Reference
Men	1.772(1.280, 2.453)^†^	3.312(1.719, 6.382)^†^	1.945(1.387, 2.727)^†^	3.872(1.889, 7.936)^†^	1.896(1.339, 2.686)^†^	4.118(1.983, 8.554)^†^
WC(cm)	1.021(1.008, 1.034)^†^	1.065(1.038, 1.094)^†^	1.023(1.010, 1.037)^†^	1.088(1.058, 1.118)^†^	1.024(1.010, 1.037)^†^	1.086(1.056, 1.117)^†^
RASM(kg/m^2^)	0.712(0.605, 0.837)^†^	0.441(0.314, 0.618)^†^	0.721(0.609, 0.852)^†^	0.493(0.338, 0.718)^†^	0.720(0.609, 0.851)^†^	0.504(0.346, 0.735)^†^
Lumbar spine BMD	1.134(0.521, 2.470)	3.634(0.775, 17.054)	1.300(0.589, 2.870)	5.013(0.896, 28.055)	1.293(0.586, 2.856)	5.059(0.915, 27.963)
Hip joint BMD	0.268(0.089, 0.809)^†^	0.002(0.000, 0.022)^†^	0.330(0.107, 1.016)	0.005(0.000, 0.071)^†^	0.327(0.106, 1.008)	0.006(0.000, 0.075)^†^
SMAF			0.621(0.376, 1.026)	0.478(0.284, 0.804)^†^	0.622(0.377, 1.026)	0.482(0.287, 0.809)^†^
MNA			0.927(0.864, 0.995)^†^	0.666(0.586, 0.757)^†^	0.928(0.865, 0.997)^†^	0.662(0.582, 0.754)^†^
MMSE			0.929(0.898, 0.962)^†^	0.842(0.793, 0.894) ^†^	0.930(0.898, 0.962)^†^	0.840(0.791, 0.893) ^†^
CCI			1.060(0.962, 1.169)	1.133(0.937, 1.371)	1.059(0.961, 1.168)	1.134(0.937, 1.372)
25(OH)D(ng/ml)					1.005(0.989, 1.022)	0.976(0.940, 1.015)
i-PTH(pg/ml)					1.000(0.998, 1.003)	1.000(0.996, 1.004)

OR = odds ratio; WC = waist circumference; RASM = relative appendicular skeletal muscle; BMD = bone mineral density; SMAF = the Functional Autonomy Measurement System; MNA = Mini-nutrition assessment; MMSE = Mini–mental state examination; CCI = Charlson’s Comorbidity Index; 25(OH)D = 25-hydroxyvitamin D; i-PTH = intact-parathyroid hormone.

^a^ Includes age, gender, bone and muscle quality

^b^ Includes age, gender, bone and muscle quality, and functional parameters

^c^ Includes age, gender, bone and muscle quality, functional parameters, serum 25(OH)D and i-PTH levels

^†^ Significant association.

## Discussion

The prevalence of frailty in different epidemiological studies varied from 4% to 13% by using different diagnostic criteria,[3,29–31] whereas the prevalence of pre-frailty ranged from 28% to 44%.[12,26] In this study, the prevalence of frailty and pre-frailty was 6.8%, and 40.5%, respectively. The results were compatible to the report from the CHS study,[26] and the prevalence was in between the two previous Taiwanese studies.[32,33] However, the inclusion/exclusion criteria of the study participants for ILAS were more similar to the CHS in that both studies focused on otherwise healthy community-dwelling older people. Therefore, we considered the prevalence of frailty in ILAS to be more feasible for international comparisons than those studies carried out Taiwan.

Obesity paradox of older people is a challenging public health issue in the aging society, which should be managed by a life course approach.[34,35] In this study, frailty was not associated with BMI and the percentage of body fat, but the waist circumferences of pre-frail and frail subjects were significantly larger than the robust subjects. Some studies suggested that central obesity and fat redistribution were important predictors of frailty,[36–38] rather than general body mass or fat mass. On the other hand, pre-frail and frail subjects had lower lean body mass, appendicular skeletal mass and lower skeletal muscle index than the robust subjects despite having similar BMI between the groups. Overall, frailty is significantly associated with the decline of physical function and changes of body composition, which may be mainly due to loss of bone and muscle mass without the significant increase in fat mass.

In this study, a strong association between frailty and lower BMD, in both the lumbar spine and hips of older adults, was identified, even after adjusting for age, gender and functional status so that they were compatible with previous studies.[18,39,40] A significant health hazard of frailty was falls and related fragility fractures.[41,42] In this study, frail subjects were more likely to fall, and to have osteoporosis, as well as sarcopenia and a history of hip fractures. Newton et al. demonstrated that the BMD was significantly lower in frail elderly people, especially among those with recurrent falls.[43] Also, the higher fracture risk of frailty was independent of BMD measurements among the elderly population.[44] Hence, a comprehensive survey of the musculoskeletal health and implementation of fall prevention was of great importance while frailty is identified in clinical practice.[45] Similar to previous studies,[46,47] frailty was associated with lower serum levels of vitamin D in this study, but the serum levels of i-PTH were similar between groups. Besides musculoskeletal health, frailty was also associated with poorer functional status, poorer cognitive function, higher malnutrition risk, and higher burden of chronic conditions as in some of the previous studies.[4,41,48] Moreover, frail elderly people also had higher serum levels of HbA1c and hs-CRP, which was related to chronic inflammation and insulin resistance.[49–51]

Despite all the effort that went into the research, there were some limitations in this study. First, the cross-sectional study design may have limited the possibilities of exploring the causal relationship of frailty and poorer musculoskeletal health of the elderly. However, since ILAS is a longitudinal cohort study, we believe that the follow-up data will facilitate in building the causal relationship between frailty and its adverse health impacts. Second, the determination of cut-offs for individual items of the frailty definition, including low physical activity, low handgrip strength and low walking speed were obtained from the study sample from the original frailty definition. Since ILAS excluded subjects with disabilities, determination of the diagnostic cutoffs may not be applied to the general population. As a result, the study may underestimate the actual prevalence of pre-frailty and frailty. Third, participants were only included when they were able to complete their physical tests. Hence, those who were unable to complete the physical function assessments were excluded, which may underestimate the true condition in the general population.

In conclusion, frailty is closely associated with lower bone mineral density, lower skeletal muscle mass, recent falls and history of hip fractures, which denotes a strong risk of further fragility fractures and associated adverse clinical outcomes. Therefore, a frailty intervention programs should take an integrated approach to strengthen both bone and muscle mass, as well as fall prevention.

## Supporting Information

S1 File ILAS frailty data. (PDF)

## References

[1] Ahmed N, Mandel R, Fain MJ. Frailty: an emerging geriatric syndrome. Am J Med. 2007;120: 748–53. 17765039 10.1016/j.amjmed.2006.10.018

[2] van Iersel MB, Rikkert MG. Frailty criteria give heterogeneous results when applied in clinical practice. J Am Geriatr Soc. 2006;54: 728–9. 16686901 10.1111/j.1532-5415.2006.00668_14.x

[3] Shimada H, Makizako H, Doi T, Yoshida D, Tsutsumimoto K, Anan Y, et al Combined prevalence of frailty and mild cognitive impairment in a population of elderly Japanese people. J Am Med Dir Assoc. 2013;14: 518–24. 10.1016/j.jamda.2013.03.010 23669054

[4] Fried LP, Ferrucci L, Darer J, Williamson JD, Anderson G. Untangling the concepts of disability, frailty, and comorbidity: implications for improved targeting and care. J Gerontol A Biol Sci Med Sci. 2004;59: 255–63. 15031310 10.1093/gerona/59.3.m255

[5] Fang X, Shi J, Song X, Mitnitski A, Tang Z, Wang C, et al-Frailty in relation to the risk of falls, fractures, and mortality in older Chinese adults: results from the Beijing Longitudinal Study of Aging. J Nutr Health Aging. 2012;16: 903–7. 10.1007/s12603-012-0368-6 23208030 PMC12878524

[6] Yamada M, Arai H, Nagai K, Uemura K, Mori S, Aoyama T. Differential determinants of physical daily activities in frail and nonfrail community-dwelling older adults. J Clin Gerontol Geriatr. 2011;2: 42–6.

[7] Makary MA, Segev DL, Pronovost PJ, Syin D, Bandeen-Roche K, Patel P, et al Frailty as a predictor of surgical outcomes in older patients. J Am Coll Surg. 2010;210: 901–8. 10.1016/j.jamcollsurg.2010.01.028 20510798

[8] Shamliyan T, Talley KM, Ramakrishnan R, Kane RL. Association of frailty with survival: a systematic literature review. Ageing Res Rev. 2013;12: 719–36. 10.1016/j.arr.2012.03.001 22426304

[9] Blain H, Rolland Y, Beauchet O, Annweiler C, Benhamou CL, Benetos A, et al Usefulness of bone density measurement in fallers. Joint Bone Spine. 2014;81: 403–8. 10.1016/j.jbspin.2014.01.020 24703626

[10] Cruz-Jentoft AJ, Baeyens JP, Bauer JM, Boirie Y, Cederholm T, Landi F, et al Sarcopenia: European consensus on definition and diagnosis: Report of the European Working Group on Sarcopenia in Older People. Age Ageing. 2010;39: 412–23. 10.1093/ageing/afq034 20392703 PMC2886201

[11] Delgado C, Doyle JW, Johansen KL. Association of frailty with body composition among patients on hemodialysis. J Ren Nutr. 2013;23: 356–62. 10.1053/j.jrn.2013.02.010 23648049 PMC4384655

[12] Cawthon PM, Marshall LM, Michael Y, Dam TT, Ensrud KE, Barrett-Connor E, et al Frailty in older men: prevalence, progression, and relationship with mortality. J Am Geriatr Soc. 2007;55: 1216–23. 17661960 10.1111/j.1532-5415.2007.01259.x

[13] Gobbens RJ, Luijkx KG, Wijnen-Sponselee MT, Schols JM. Toward a conceptual definition of frail community dwelling older people. Nurs Outlook. 2010;58: 76–86. 10.1016/j.outlook.2009.09.005 20362776

[14] Chen LK, Rockwood K. Planning for frailty. J Clin Gerontol Geriatr; 2012;3: 3–4.

[15] Chen LK, Inoue H, Won CW, Lin CH, Lin KF, Tsay SF, et al Challenges of urban aging in Taiwan: Summary of urban aging forum. J Clin Gerontol Geriatr. 2013;4: 97–101.

[16] Jung HW, Kim SW, Lim JY, Kim KW, Jang HC, Kim CH, et al Frailty status can predict further lean body mass decline in older adults. J Am Geriatr Soc. 2014;62: 2110–7. 10.1111/jgs.13107 25370293

[17] Cooper C, Dere W, Evans W, Kanis JA, Rizzoli R, Sayer AA, et al Frailty and sarcopenia: definitions and outcome parameters. Osteoporos Int. 2012;23: 1839–48. 10.1007/s00198-012-1913-1 22290243

[18] Rolland Y, Abellan van Kan G, Benetos A, Blain H, Bonnefoy M, Chassagne P, et al Frailty, osteoporosis and hip fracture: causes, consequences and therapeutic perspectives. J Nutr Health Aging. 2008;12: 335–46. 18443717 10.1007/BF02982665 PMC12892620

[19] Liu LK, Lee WJ, Chen LY, Hwang AC, Lin MH, Peng LN, et al Sarcopenia, and its association with cardiometabolic and functional characteristics in Taiwan: results from I-Lan Longitudinal Aging Study. Geriatr Gerontol Int. 2014;14 Suppl 1: 36–45. 10.1111/ggi.12208 24450559

[20] Charlson ME, Pompei P, Ales KL, MacKenzie CR. A new method of classifying prognostic comorbidity in longitudinal studies: development and validation. Journal of Chronic Diseases. 1987;40: 373–83. 3558716 10.1016/0021-9681(87)90171-8

[21] Hebert R, Carrier R, Bilodeau A. The Functional Autonomy Measurement System (SMAF): description and validation of an instrument for the measurement of handicaps. Age Ageing. 1988;17: 293–302. 2976575 10.1093/ageing/17.5.293

[22] LS R. The CES-D Scale: a self-report depression scale for research in the general population. Appl Psychol Meas. 1977;1: 385–401.

[23] Guigoz Y. The Mini Nutritional Assessment (MNA) review of the literature—What does it tell us? J Nutr Health Aging. 2006;10: 466–85; discussion 485–7. 17183419

[24] Folstein MF, Folstein SE, McHugh PR. "Mini-mental state". A practical method for grading the cognitive state of patients for the clinician. J Psychiatr Res. 1075;12: 189–98. 1202204 10.1016/0022-3956(75)90026-6

[25] Liu LK, Lee WJ, Liu CL, Chen LY, Lin MH, Peng LN, et al Age-related skeletal muscle mass loss and physical performance in Taiwan: Implications to diagnostic strategy of sarcopenia in Asia. Geriatr Gerontol Int. 2013;13: 964–71. 10.1111/ggi.12040 23452090

[26] Fried LP, Tangen CM, Walston J, Newman AB, Hirsch C, Gottdiener J, et al Frailty in older adults: evidence for a phenotype. J Gerontol A Biol Sci Med Sci. 2001;56: M146–56. 11253156 10.1093/gerona/56.3.m146

[27] Qu NN, Li KJ. [Study on the reliability and validity of international physical activity questionnaire (Chinese Vision, IPAQ)]. Zhonghua Liu Xing Bing Xue Za Zhi. 2004;25: 265–8. 15200945

[28] Liou YM, Jwo CJ, Yao KG, Chiang LC, Huang LH. Selection of appropriate Chinese terms to represent intensity and types of physical activity terms for use in the Taiwan version of IPAQ. J Nurs Res. 2008;16: 252–63. 19061172 10.1097/01.jnr.0000387313.20386.0a

[29] Moreira VG, Lourenco RA. Prevalence and factors associated with frailty in an older population from the city of Rio de Janeiro, Brazil: the FIBRA-RJ Study. Clinics (Sao Paulo). 2013;68: 979–85. 23917663 10.6061/clinics/2013(07)15 PMC3714993

[30] Jurschik P, Nunin C, Botigue T, Escobar MA, Lavedan A, Viladrosa M. Prevalence of frailty and factors associated with frailty in the elderly population of Lleida, Spain: the FRALLE survey. Arch Gerontol Geriatr. 2012;55: 625–31. 10.1016/j.archger.2012.07.002 22857807

[31] Jung HW, Kim SW, Ahn S, Lim JY, Han JW, Kim TH, et al Prevalence and outcomes of frailty in Korean elderly population: comparisons of a multidimensional frailty index with two phenotype models. PLoS One. 2014;9: e87958 10.1371/journal.pone.0087958 24505338 PMC3913700

[32] Lin CC, Li CI, Meng NH, Lin WY, Liu CS, Lin CH, et al Frailty and its associated factors in an elderly taiwanese metropolitan population. J Am Geriatr Soc. 2013;61: 292–4. 10.1111/jgs.12103 23405925

[33] Chen CY, Wu SC, Chen LJ, Lue BH. The prevalence of subjective frailty and factors associated with frailty in Taiwan. Arch Gerontol Geriatr. 2010;50 Suppl 1: S43–7. 10.1016/S0167-4943(10)70012-1 20171456

[34] Strandberg TE, Stenholm S, Strandberg AY, Salomaa VV, Pitkala KH, Tilvis RS. The "obesity paradox," frailty, disability, and mortality in older men: a prospective, longitudinal cohort study. Am J Epidemiol. 2013;178: 1452–60. 10.1093/aje/kwt157 24008903

[35] Kim YP, Kim S, Joh JY, Hwang HS. Effect of interaction between dynapenic component of the European working group on sarcopenia in older people sarcopenia criteria and obesity on activities of daily living in the elderly. J Am Med Dir Assoc. 2014;15: 371 e1–5. 10.1016/j.jamda.2013.12.010 24512811

[36] Shah K, Hilton TN, Myers L, Pinto JF, Luque AE, Hall WJ. A new frailty syndrome: central obesity and frailty in older adults with the human immunodeficiency virus. J Am Geriatr Soc. 2012;60: 545–9. 10.1111/j.1532-5415.2011.03819.x 22315957 PMC3302930

[37] Hubbard RE, Lang IA, Llewellyn DJ, Rockwood K. Frailty, body mass index, and abdominal obesity in older people. J Gerontol A Biol Sci Med Sci. 2010;65: 377–81. 10.1093/gerona/glp186 19942592

[38] Goulet ED, Hassaine A, Dionne IJ, Gaudreau P, Khalil A, Fulop T, et al Frailty in the elderly is associated with insulin resistance of glucose metabolism in the postabsorptive state only in the presence of increased abdominal fat. Exp Gerontol. 2009;44: 740–4. 10.1016/j.exger.2009.08.008 19723576

[39] Crepaldi G, Maggi S. Sarcopenia and osteoporosis: A hazardous duet. J Endocrinol Invest. 2005;28(10 Suppl): 66–8. 16550726

[40] Sternberg SA, Levin R, Dkaidek S, Edelman S, Resnick T, Menczel J. Frailty and osteoporosis in older women—a prospective study. Osteoporos Int. 2014;25: 763–8. 10.1007/s00198-013-2471-x 24002542

[41] Tom SE, Adachi JD, Anderson FA Jr., Boonen S, Chapurlat RD, Compston JE, et al Frailty and fracture, disability, and falls: a multiple country study from the global longitudinal study of osteoporosis in women. J Am Geriatr Soc. 2013;61: 327–34. 10.1111/jgs.12146 23351064 PMC3602412

[42] Womack JA, Goulet JL, Gibert C, Brandt CA, Skanderson M, Gulanski B, et al Physiologic frailty and fragility fracture in HIV-infected male veterans. Clin Infect Dis. 2013;56: 1498–504. 10.1093/cid/cit056 23378285 PMC3634308

[43] Newton JL, Kenny RA, Frearson R, Francis RM. A prospective evaluation of bone mineral density measurement in females who have fallen. Age Ageing. 2003;32: 497–502. 12957998 10.1093/ageing/afg062

[44] Cheung EY, Bow CH, Cheung CL, Soong C, Yeung S, Loong C, et al Discriminative value of FRAX for fracture prediction in a cohort of Chinese postmenopausal women. Osteoporos Int. 2012;23: 871–8. 10.1007/s00198-011-1647-5 21562875

[45] Frisoli A Jr., Chaves PH, Ingham SJ, Fried LP. Severe osteopenia and osteoporosis, sarcopenia, and frailty status in community-dwelling older women: results from the Women's Health and Aging Study (WHAS) II. Bone. 2011;48: 952–7. 10.1016/j.bone.2010.12.025 21195216

[46] Hirani V, Naganathan V, Cumming RG, Blyth F, Le Couteur DG, Handelsman DJ, et al Associations between frailty and serum 25-hydroxyvitamin D and 1,25-dihydroxyvitamin D concentrations in older Australian men: the Concord Health and Ageing in Men Project. J Gerontol A Biol Sci Med Sci. 2013;68: 1112–21. 10.1093/gerona/glt059 23657973

[47] Wong YY, McCaul KA, Yeap BB, Hankey GJ, Flicker L. Low vitamin D status is an independent predictor of increased frailty and all-cause mortality in older men: the Health in Men Study. J Clin Endocrinol Metab. 2013;98: 3821–8. 10.1210/jc.2013-1702 23788685

[48] Fried LP, Kronmal RA, Newman AB, Bild DE, Mittelmark MB, Polak JF, et al Risk factors for 5-year mortality in older adults: the Cardiovascular Health Study. JAMA. 1998;279: 585–92. 9486752 10.1001/jama.279.8.585

[49] Morley JE, Haren MT, Rolland Y, Kim MJ. Frailty. Medical Clinics of North America. 2006;90: 837–47. 16962845 10.1016/j.mcna.2006.05.019

[50] Morley JE, Baumgartner RN. Cytokine-related aging process. J Gerontol A Biol Sci Med Sci. 2004;59: M924–9. 15472157 10.1093/gerona/59.9.m924

[51] Abbatecola AM, Ferrucci L, Grella R, Bandinelli S, Bonafe M, Barbieri M, et al Diverse effect of inflammatory markers on insulin resistance and insulin-resistance syndrome in the elderly. J Am Geriatr Soc. 2004;52: 399–404. 14962155 10.1111/j.1532-5415.2004.52112.x

