# Supplementary material for: Association between Frailty, Osteoporosis, Falls and Hip Fractures among Community-Dwelling People Aged 50 Years and Older in Taiwan: Results from I-Lan Longitudinal Aging Study
Source: PLoS One. 2015 Sep 8;10(9):e0136968. doi: 10.1371/journal.pone.0136968 (PMC4562637; doi:10.1371/journal.pone.0136968)
Supplement: S1 File — (PDF) [file pone.0136968.s001.pdf]

ILAS frailty data

No.	Age	Gender	Smoking	Alcohol	WC	BMI	WS	GS	Frail	Fat%	Fat	LBM	ASM	RASM
1	55	2	0	0	105.1	35.89	0.84	18	1					
2	52	1	1	0	82	24.56	1.5	35	0	29.3	19.07	46.04	20.37	7.31
3	69	2	0	0	82	26.65	1.2	17	1	36	19.88	35.35	15.95	7.01
4	68	2	0	0	94	29.52	1	25	1	36.6	22.69	39.34	17.10	7.21
5	52	2	0	0	79	22.67	1	21	1	32.5	14.99	31.13	10.50	4.92
6	53	1	1	1	81	23.35	2	42	0	22.1	12.17	42.80	19.31	8.11
7	83	1	1	0	67	15.55	0.6	23.5	2	10.4	4.31	37.04	14.69	5.08
8	53	2	0	0	94	24.97	1.2	25	0	42.6	24.19	32.56	14.53	6.05
9	62	1	0	1	70	22.99	1.2	46	1	15	8.23	46.68	20.91	8.42
10	74	1	0	1	85.5	25.42	1	30	1	29.9	18.47	43.27	18.08	7.26
11	61	1	1	1	93	26.20	1.2	46	1	27	19.78	53.43	25.40	8.73
12	55	2	0	0	78	23.67	1.5	23.5	1	33.1	17.63	35.66	14.99	6.43
13	51	2	1	1	80.5	21.97	1.5	21	0	42.3	21.85	29.78	11.47	4.82
14	56	1	0	0	95	28.60	2	46	0	34.5	26.65	50.54	23.93	8.65
15	69	1	3	1	87	25.34	1.5	32	0	24.3	14.49	45.09	20.46	8.53
16	50	2	0	0	70	19.56	2	33	0	25.7	10.69	30.84	13.36	5.94
17	75	1	1	1	92	22.83	0.98	40	1	24.7	14.74	44.84	18.58	6.91
18	61	2	0	0	70	20.22	1.6	19	0	32.5	14.46	30.04	12.68	5.64
19	82	2	0	0	96	25.33	1.04	16	2	36.5	20.81	36.24	15.30	6.8
20	70	1	1	0	90	24.53	1.47	28	1	23.4	14.20	46.54	19.04	7.44
21	58	2	0	0	76	21.91	1.04	10	1	26.5	11.51	31.97	12.76	5.67
22	71	2	0	0	72	27.97	1.1	22.5	1	36.7	21.03	36.24	14.90	7.08
23	81	2	0	0	104	28.44	0.7	20	2	44.8	28.12	34.67	13.61	6.05
24	58	1	0	0	88	22.81	1.35	36	0	23.6	14.22	46.13	21.39	7.86
25	78	1	1	0	94	25.33	1.17	25	1	27.4	15.25	40.46	16.38	7.28
26	82	2	0	0	74	18.90	0.84	5	2	32.8	14.20	29.13	11.22	4.83
27	74	2	0	0	80	28.54	0.88	15	2	23	9.63	32.31	14.18	6.74
28	80	1	3	1	104	29.52	0.7	31	1	33.9	23.21	45.27	18.62	7.85
29	82	2	0	0	97	30.41	0.77	16	1	43.4	24.49	31.97	11.10	6.03
30	91	1	1	0	106	26.60	0.59	7	2	28.5	19.90	49.82	21.55	8.12
31	67	2	0	0	87	26.97	1.17	28	1	39.1	27.12	42.32	18.16	7.01
32	61	2	0	0	85.7	26.40	1.17	26	1	40.1	22.60	33.81	13.93	6.43
33	51	2	0	0	66	21.38	0.98	32	1	29	14.9[illegible]	[illegible]	[illegible]	[illegible]
34	68	2	0	0	80	27.10	0.88	20	1	37.9	[illegible]	[illegible]	[illegible]	[illegible]
35	53	1	0	0	76	23.22	1.1	30	1	25.6	14.47	42.13	18.40	7.28
36	78	1	1	0	72	20.37	0.88	25	2	13.3	6.46	42.18	18.41	7.82
37	60	1	1	0	84	24.34	1.04	38.5	1	20.3	12.10	47.44	19.90	8.07
38	62	1	1	1	88.5	24.59	1.96	35	0	27.9	19.88	51.36	23.57	7.88
39	86	2	3	0	81	23.19	0.7	8.5	1	41.4	22.60	32.06	13.47	5.68
40	71	1	0	0	90	26.08	1.35	43	2	32.8	22.84	46.73	21.46	7.88
41	77	1	0	0	80.2	22.83	0.84	21.5	1	26	14.76	41.90	18.77	7.56
42	59	2	0	0	90	27.34	0.8	25	1	41.5	28.83	40.70	17.58	6.87
43	73	2	0	0	87.5	26.10	0.7	19	1	42	26.37	36.37	14.74	6.09
44	57	2	0	0	71	22.56	1.1	24	1	32.1	15.97	33.72	13.72	6.06
45	55	2	0	1	82	23.05	1.76	22.5	1	35.5	19.49	35.36	15.12	6.3
46	69	2	1	0	80.9	25.76	1.04	22	1	35.7	20.64	37.20	15.67	6.94
47	77	1	3	0	89	24.09	1.47	34	0	30.5	18.54	42.28	17.59	6.96
48	68	1	1	0	98	27.48	0.39	21	2	31	22.50	50.07	20.78	7.82
49	56	1	1	1	91.5	26.78	1.17	23	1	26.8	18.35	50.18	21.79	8.39
50	83	2	0	0	83	20.10	0.7	10	2	28.6	12.17	30.40	12.64	5.92
51	61	1	0	0	84	24.46	1.26	26	1	22.8	13.81	46.63	20.36	8.13
52	57	1	0	1	75	17.38	1.35	35	1	14.5	6.89	40.66	17.44	6.28
53	88	1	3	0	92	25.26	0.77	20	1	28.9	17.34	42.63	17.19	7.37
54	69	1	0	0	89.1	24.94	0.98	30	1	22.8	14.26	48.38	20.02	7.89
55	68	2	0	0	96	29.01	0.98	23.5	1	40	25.60	38.42	15.36	6.92
56	70	2	0	1	79	22.12	0.88	22	1	26.8	13.44	36.72	15.79	6.97
57	75	2	0	0	76.5	22.63	1.26	20	0	34.6	16.94	31.96	14.16	6.5
58	81	1	0	0	95	25.90	0.88	27	2	34.6	22.43	42.32	17.13	6.74
59	56	2	0	1	69.5	20.03	0.98	23	1	28.7	13.14	32.72	13.80	6
60	80	2	0	0	90	27.64	0.98	24	1	42.6	24.81	33.47	13.05	6.11
61	61	2	0	0	80	25.11	1.17	25.5	1	39.1	22.54	35.16	15.07	6.56
62	67	1	1	0	68.5	17.71	0.98	32	1	8.4	3.95	43.11	18.76	7.07
63	61	1	0	0	91	28.62	1.1	54	1	27	19.96	53.96	24.66	9.36
64	63	2	0	0	78	23.92	1.1	17.5	1	42.8	26.17	34.90	14.15	5.48
65	82	1	1	0	89	24.20	0.98	30	1	30.9	18.96	42.42	18.16	7
66	55	2	0	0	70	21.24	0.88	13	2	30.2	14.34	33.13	13.35	5.85
67	73	1	3	0	84	23.14	1.04	32	1	21.8	12.81	46.01	18.91	7.41
68	62	2	0	1	68	17.93	1.17	17.5	1	21.8	8.93	32.09	13.23	5.7
69	64	2	0	0	79	21.56	0.98	18	1	32.9	16.42	33.56	13.47	5.7
70	63	2	0	0	75	23.78	1.17	19.5	0	39.6	20.97	32.00	13.69	6.11
71	56	2	0	0	96	32.11	1.17	28	0	36.5	26.75	46.51	19.58	8.55

72	63	2	0	0	83	26.15	1.17	26.5	1	38.1	23.24	37.68	16.71	7.13
73	53	1	3	1	96.5	29.74	1.76	47	0	38.9	30.33	47.61	21.80	8.1
74	79	1	0	0	73.1	21.96	0.88	35	1	20.7	9.93	38.06	16.93	7.52
75	76	1	3	1	78	24.44	0.5	21	1					
76	53	2	0	0	92	28.73	0.93	15	1	46.1	29.33	34.24	13.27	5.87
77	60	2	0	0	71	25.89	1.17	20.5	0	39.8	22.75	34.38	13.17	5.91
78	54	1	0	1	69	21.57	1.76	41	1	13.2	6.90	45.24	20.78	8.41
79	76	1	3	0	98	26.05	1.17	31	1	34.1	22.20	42.90	19.02	7.44
80	79	2	0	0	64.5	16.80	0.8	8	2	16.1	5.81	30.36	11.80	5.46
81	53	1	0	0	73	22.41	1.17	26	1	18.7	9.73	42.25	18.91	7.99
82	61	1	0	0	76.5	19.53	1.47	36	0	16.9	8.21	40.47	18.43	7.23
83	80	1	1	0	72	17.28	0.98	15	2	14.8	7.17	41.21	17.92	6.24
84	51	1	1	1	87	24.23	1.96	40	1	25.4	19.49	57.34	27.02	8.34
85	74	1	0	0	83	22.06	1.35	44	1	23.9	13.76	43.85	19.44	7.16
86	53	1	3	1	93	27.92	1.17	45	1	35.7	25.08	45.16	20.07	7.67
87	63	1	1	1	85	23.04	1.26	44.5	0	24.1	15.76	49.57	22.25	7.54
88	64	1	3	1	87	22.72	0.24	22	1	27.7	16.05	41.96	18.23	7.13
89	73	1	1	1	84.5	22.21	1.96	40	0	28.6	20.87	51.97	23.32	7.05
90	52	2	0	0	90	27.81	1.47	32	0	41.2	28.94	41.30	17.67	6.9
91	52	2	0	0	72	24.03	1.1	20	1	45.7	25.33	30.09	12.29	5.24
92	52	2	0	0	89	26.32	1.17	26	0	36.2	22.84	40.18	16.03	6.51
93	74	1	1	0	78	21.32	1.1	41	1	12.8	8.03	54.71	22.89	7.68
94	54	2	0	1	78.5	25.62	1.47	26	0	36.4	22.49	39.26	17.71	7.2
95	78	1	0	1	85.5	23.77	1.76	32	1	31.6	18.70	40.43	18.63	7.33
96	60	2	0	0	74	19.36	1.04	23	1	23.7	11.02	35.43	14.83	6.11
97	63	1	1	0	83	25.50	1.17	33	1	24.9	16.16	48.83	21.85	8.43
98	58	2	0	0	82	23.56	0.93	23	1	36.2	19.10	33.69	14.18	6.21
99	54	2	0	0	82	24.57	1.26	21.4	0	41.7	23.15	32.38	13.52	5.89
100	68	1	0	1	83	22.93	1.17	35	1	22.2	12.81	45.00	19.51	7.54
101	58	2	0	0	98.5	30.87	1.17	22	0	46.3	32.09	37.20	14.95	6.46
102	69	2	0	0	92	25.52	1.17	18	0	34.9	21.87	40.74	16.55	6.67
103	65	2	0	0	86	28.79	1.47	28	0	48.4	33.79	36.02	15.86	6.38
104	69	2	0	0	67.6	20.22	1.1	16	1	27.7	12.76	33.33	14.39	6.07
105	70	1	1	1	80	22.35	1.96	40	0	23.1	14.36	47.94	21.52	7.6
106	66	2	0	0	80	23.10	1.17	25	0	34	19.22	37.31	15.39	6.23
107	63	1	1	1	98	30.43	0.88	35	2	33.5	25.67	50.84	22.57	8.78
108	77	1	0	0	84	24.55	1.17	35	1	26.3	15.61	43.74	19.69	7.98
109	55	1	3	1	88	25.28	1.17	50	1	26.6	18.41	50.85	23.36	8.31
110	79	1	3	0	92	27.09	1.76	38	0	32.3	21.95	46.07	20.67	8.04
111	61	2	0	0	80.1	20.84	0.98	25	1	32.5	18.43	38.28	15.89	6.15
112	69	1	3	0	99.5	29.82	1.35	37	1	31.1	23.52	52.15	21.71	8.04
113	67	1	0	0	92	26.83	1.04	38	1	34.4	25.29	48.30	21.89	7.83
114	75	1	1	1	97	24.82	1.17	18	1	29.5	17.87	42.71	18.24	7.36
115	59	2	0	0	86	27.86	1.17	27	0	45.8	29.68	35.18	15.16	6.34
116	57	2	0	0	79	23.83	1.35	24	0	42	22.88	31.62	13.45	5.75
117	56	2	0	1	83	23.51	1.04	18	1	42.6	21.98	29.60	12.05	5.36
118	69	1	1	0	110	34.44	1.47	25	1	33	25.53	51.76	19.02	8.16
119	59	1	0	1	82	21.43	1.76	44	0	11	6.88	55.93	24.69	8.27
120	70	1	1	0	89	25.62	1.6	33	0	25.3	15.85	46.90	19.66	7.9
121	64	1	1	1	95	27.68	1.26	49	0	32.4	23.95	50.00	22.27	8.12
122	59	2	0	0	82	25.20	1.35	25	0	40.8	22.14	32.17	13.16	6.01
123	55	1	1	1	91	24.18	1.76	50	1	28.2	18.75	47.85	21.35	7.55
124	66	1	1	0	80	19.66	1.76	28	1	25.3	13.36	39.43	16.03	5.82
125	63	1	3	1	94	25.33	1.04	32	1	25.2	16.43	48.72	20.96	8.01
126	59	2	0	0	79	23.56	1.17	15	1	36.5	19.03	33.17	13.26	5.9
127	55	1	1	1	81	21.24	1.35	42	1	20.5	12.05	46.63	21.00	7.34
128	72	1	1	1	93	30.34	1.04	45	1	32.2	25.39	53.48	23.85	8.91
129	65	1	0	0	105	33.68	0.98	32	1	33.2	27.92	56.29	21.90	8.59
130	61	1	3	1	78	24.26	1.1	41.5	1	22.8	14.35	48.73	22.11	8.25
131	53	2	0	0	90	25.29	1.76	30	0	36.1	24.62	43.50	19.03	6.91
132	58	1	0	0	98	31.77	1.47	40	1	31.7	24.34	52.52	21.76	8.83
133	53	1	1	0	79	22.46	1.47	41.5	1	21.6	11.80	42.80	19.57	7.82
134	60	1	0	0	96	28.77	1.26	34	1	35	30.10	55.79	25.18	8.28
135	56	1	0	1	73	20.81	2.2	41	0	16.2	10.92	56.46	25.18	7.56
136	80	2	0	0	98.2	29.79	0.63	10	2	47.4	31.14	34.59	13.25	5.87
137	52	2	1	1	80	20.85	1.17	20	1	35.7	16.09	28.96	12.33	5.61
138	77	1	1	0	87	23.10	1.1	32	2	30.4	19.86	45.43	19.36	6.79
139	51	1	0	1	80	23.39	1.17	39	1	25	15.59	46.88	21.46	7.85
140	53	2	0	1	77	24.83	2.2	31	0	34.5	20.98	39.90	18.74	7.38
141	82	1	0	1	86	23.59	1.17	27	1	28	16.45	42.25	18.60	7.23
142	53	1	1	1	84	23.99	1.17	47	1	22	14.24	50.34	21.87	7.93
143	80	1	3	0	88	22.31	0.88	20	2	23.2	13.69	45.26	18.79	6.99

144	51	1	1	1	87	23.07	1.76	46	0	24.4	16.24	50.36	22.90	7.72
145	72	2	1	1	84	25.29	0.98	25	1	41.9	24.67	34.27	14.15	6
146	64	2	0	0	86.5	28.07	1.35	31	1	45.9	34.14	40.28	17.80	6.63
147	60	2	0	0	67	21.36	0.88	23	2	29.6	12.87	30.63	12.92	6.01
148	56	2	0	1	83	23.71	1.76	25	1	38.1	21.25	34.51	14.12	6
149	60	2	0	0	95	32.47	0.98	28	1	42.5	30.08	40.64	17.05	7.26
150	66	2	1	0	99	28.48	1.6	35	0	39.6	28.20	42.95	18.34	7.25
151	51	1	1	1	90	25.42	1.76	45	0					
152	53	2	0	0	74.5	20.51	1.96	26	1	32.8	16.31	33.48	13.86	5.65
153	63	1	1	1	80	24.89	1.1	40.5	1	22.6	13.44	46.01	20.74	8.49
154	63	2	0	0	80	24.00	0.88	17	1	33.5	19.80	39.34	16.73	6.56
155	73	2	0	0	72	19.99	1.35	20	0	24.9	10.88	32.75	13.04	5.9
156	56	1	0	1	85	23.36	2.51	52	0	18.6	12.55	54.77	24.92	8.47
157	58	1	0	1	82	21.77	1.76	35	0	21.1	10.97	40.92	17.46	7.07
158	65	1	1	1	83	24.14	1.17	48	1	19.7	12.68	51.57	23.07	8.57
159	64	1	3	1	105	30.05	1.35	40	0	27.9	22.54	58.27	24.88	9.05
160	54	2	0	0	82	26.15	0.98	25	1	34.5	20.63	39.12	15.16	6.47
161	60	2	0	1	80	23.59	1.6	27	0	32.3	17.83	37.38	15.22	6.47
162	56	2	0	0	88.5	23.84	1.17	26	0	33.5	20.99	41.64	16.84	6.36
163	61	2	1	0	90	22.57	0.98	18	1	45.6	24.24	28.89	11.40	4.72
164	56	2	1	0	80	25.26	0.88	26	1	43.2	25.65	33.76	13.76	5.6
165	60	1	0	1	89	27.15	1.96	41	0	25.5	18.54	54.07	24.82	9.1
166	57	1	0	1	94.5	30.31	1.6	46	0	35.7	25.37	45.61	20.90	8.7
167	54	2	0	0	75.5	23.33	1.47	24	0	38.9	20.39	32.01	13.62	5.9
168	64	1	0	0	89	24.32	1.17	44	1	29.4	19.66	47.11	20.17	7.23
169	71	2	0	0	85	35.21	1.04	22	1	48.2	28.73	30.92	12.98	6.35
170	60	2	0	0	107	32.91	1.35	21	0	49.9	39.21	39.30	17.27	7.1
171	72	2	0	1	86	24.36	1.26	20	0	32.1	18.57	39.23	16.45	6.84
172	61	1	0	1	67	18.59	1.76	40	0	16.2	8.04	41.47	18.51	6.97
173	70	2	0	0	89	28.94	1.17	8.5	1	45	27.15	33.15	14.00	6.57
174	57	1	3	1	106	32.08	1.76	42	0	37	29.40	49.95	22.01	8.7
175	70	2	0	0	90	28.85	1.26	22	0	41.8	25.98	36.16	15.07	6.79
176	66	2	0	0	86	26.44	0.98	25	1	37.2	23.77	40.06	17.09	6.93
177	63	2	0	0	86	22.27	1.35	26	1	39.8	22.45	33.92	13.78	5.32
178	57	2	0	0	89	28.04	1.1	26	1	42	29.81	41.11	16.75	6.54
179	57	1	1	1	87	25.00	1.35	43	1	19.7	12.45	50.59	22.36	8.73
180	67	1	1	1	100	25.08	1.17	32	1	27.1	18.37	49.42	20.70	7.42
181	52	2	0	0	77	25.14	1.35	30	0	44.7	26.94	33.36	14.92	6.05
182	57	1	0	0	94	28.34	1.26	50	0	35.8	28.59	51.36	23.40	8.1
183	55	2	0	1	78	22.18	1.35	26.5	0	29	15.83	38.73	16.88	6.76
184	82	1	0	0	80	23.45	1.26	20	1	25.3	12.62	37.24	15.00	7.23
185	68	1	3	1	90	25.48	1.47	43	0	20.8	15.07	57.41	26.02	9.27
186	63	1	0	1	96	26.64	1.17	22	2	32.7	22.00	45.23	19.33	7.28
187	57	1	0	1	93	27.46	1.35	44	1	33.8	25.25	49.49	22.83	8.21
188	57	2	0	1	82	25.07	1.35	23	0	37.4	20.99	35.18	15.00	6.58
189	54	1	0	0	84	22.73	2.51	30	1	23.4	14.50	47.43	21.22	7.61
190	53	2	0	1	82	22.42	1.26	30	0	41.3	22.08	31.34	13.54	5.43
191	73	1	1	1	99	26.26	1.17	28	1	24	17.79	56.48	24.19	8.18
192	70	1	3	1	90.6	24.89	1.17	32	1	30.9	18.45	41.26	18.13	7.17
193	62	2	0	0	74	20.00	1.17	21.5	0	32.6	15.13	31.23	11.04	4.78
194	61	1	1	1	89	24.84	1.6	40	0	21	13.76	51.61	23.04	8.46
195	70	1	3	0	101	31.66	1.47	41	0	36.4	30.80	53.73	22.59	8.3
196	71	1	0	1	89	25.06	1.6	35	1	28.2	17.70	45.07	19.79	7.73
197	62	1	3	0	89	24.87	1.35	35	0	30.2	19.15	44.23	18.56	7.25
198	61	2	0	0	73	24.66	1.17	17	1	43.7	20.75	26.76	10.07	5.07
199	59	2	0	0	80	22.03	1.1	13	1	36.9	17.61	30.07	12.78	5.76
200	55	2	0	0	83	23.38	1.6	20.5	0	35.3	18.57	33.98	14.41	6.32
201	69	2	0	1	89	24.44	1.6	21	0	33.6	15.61	30.81	11.38	5.81
202	66	2	0	0	92	28.98	1.17	19	1	40.5	25.68	37.68	14.58	6.48
203	71	1	1	1	99	27.93	1.35	35	1	26.2	18.37	51.81	20.95	8.08
204	51	2	0	0	68	21.57	1.96	5	1	33.5	18.36	36.45	16.03	6.25
205	51	1	0	0	95	29.28	1.17	32.5	1	30.8	24.36	54.66	24.99	9.07
206	52	1	1	1	90	25.13	1.04	33	1	21	14.25	53.53	22.38	8.22
207	58	1	0	1	90	26.36	1.6	32	0	30.1	21.61	50.25	23.27	8.34
208	66	1	1	0	86.5	25.22	1.35	30	0	25	16.54	49.63	23.17	8.72
209	55	2	0	0	94.5	32.13	1.26	25	0	50.9	37.75	36.38	15.49	6.53
210	56	2	0	0	96	25.57	1.1	24	1	28	17.74	45.55	16.47	6.6
211	62	1	1	1	87	24.84	1.17	30	1	23.3	14.04	46.09	19.86	8.04
212	56	2	0	1	76.5	23.05	1.04	23	1	38.7	23.53	37.26	16.45	6.12
213	57	2	0	0	82	26.06	1.6	25	0	38.2	22.66	36.70	9.81	4.19
214	65	2	0	1	91.5	27.56	0.98	19	1	39.1	23.58	36.72	14.06	6.25
215	77	1	0	0	85	22.86	1.47	30	0	24.1	14.75	46.49	19.84	7.2

216	65	2	0	0	91	27.57	1.04	21	2	39.4	26.42	40.59	18.03	7.42
217	53	2	0	0	80	21.88	0.98	29	1	30.7	16.93	38.22	16.23	6.34
218	56	1	3	1	89	22.12	1.76	47	0	13	8.54	57.12	26.80	8.96
219	71	2	0	0	77	23.46	1.17	23	1	27.4	16.34	43.36	18.19	7.26
220	57	2	0	0	124	39.38	1.17	30	0					
221	66	2	0	0	87	26.41	1.1	18	1	41	25.12	36.17	14.74	6.29
222	61	1	0	0	81	22.54	1.17	37	1	19.2	11.30	47.47	20.73	7.92
223	64	2	0	0	88	29.10	0.88	20	2	44.9	27.61	33.84	14.46	6.77
224	79	1	1	0	88	25.85	1.47	18	1	25.8	17.12	49.35	21.11	8.26
225	72	2	0	0	88	25.30	2	28	0	38.6	25.44	40.49	16.11	6.18
226	71	2	0	0	95.5	25.16	0.86	21	2	36	18.51	32.87	13.79	6.7
227	67	2	0	0	88	30.75	0.75	15	1	44.9	30.13	36.92	15.38	7.07
228	53	1	1	1	77	22.28	1.5	45	0	13.7	8.89	55.84	25.90	8.74
229	64	1	1	0	99	29.96	1.2	40.5	1	31.9	23.67	50.54	21.58	8.67
230	73	2	0	1	85	23.69	0.67	20	1	34.3	17.07	32.76	13.28	6.19
231	53	2	0	0	113	37.04	1.5	32	0	43	38.44	51.00	20.41	8.16
232	71	1	1	0	67	18.93	1	28	1	10.5	5.12	43.64	19.52	7.3
233	74	2	0	1	83	26.29	1.5	19	0	33.5	19.32	38.34	16.12	7.05
234	78	2	0	1	72.5	18.95	0.86	17	2					
235	57	2	0	1	80	22.75	1.2	24.5	0	37	19.74	33.59	13.96	5.72
236	55	1	3	0	90	26.04	1.5	49.5	0	27.5	20.17	53.19	23.54	8.17
237	53	1	0	1	96	28.18	2	54	1	28.4	24.74	62.37	28.24	8.87
238	53	1	0	1	102.5	30.65	1.2	41.5	1	31.6	29.03	62.98	29.97	9.73
239	73	1	1	0	80	21.53	2	25	1	22.9	12.24	41.12	18.51	7.26
240	54	1	0	1	83	24.79	2	46	1	23.6	16.83	54.62	25.44	8.5
241	72	1	3	0	84	22.18	1.2	34	1	24.8	15.59	47.39	20.45	6.94
242	67	1	1	0	103	27.87	1.2	37	1	30.7	22.93	51.83	20.65	7.38
243	56	1	1	1	86.5	25.17	1.5	49	0	30.6	23.58	53.48	24.17	7.66
244	69	2	0	0	81	24.07	1.5	24	0	40.4	24.84	36.58	15.85	6.29
245	53	1	0	1	94	26.26	2	34	0	28	21.43	55.11	24.97	8.45
246	57	1	0	0	82.5	22.28	1.5	41	0	21.1	13.44	50.23	22.51	7.83
247	54	2	1	1	97	30.56	1.5	23	1	45.1	35.06	42.60	17.10	6.51
248	55	1	0	1	79	22.55	3	45.5	0	15.9	8.46	44.78	19.50	8.13
249	54	1	1	1	84	24.15	1.2	50	1					
250	52	2	0	0	79	21.14	1.2	17.5	1					
251	72	1	0	1	83	23.78	2	32	0	24.7	14.58	44.43	19.59	7.9
252	59	1	1	1	88	23.31	3	36	1	23.4	13.56	44.51	19.26	7.46
253	74	1	1	0	88	23.37	1.5	31	0					
254	78	2	3	1	86	22.54	1.2	26	1	32.8	15.73	32.24	11.88	5.47
255	88	1	3	0	91	25.70	1.2	29	2	27.6	20.02	52.44	23.12	7.95
256	60	1	0	1	96	31.28	1.5	43.5	1	34.7	30.39	57.12	26.89	9.29
257	59	1	1	1	90	26.06	2	34	0	26	18.56	52.93	22.77	8.11
258	71	1	1	1	74	17.58	1.5	35	1	9.3	4.55	44.08	18.76	6.61
259	75	2	0	0	83	26.74	1	21	1	43.1	25.53	33.76	13.32	5.9
260	63	2	0	0	69	19.18	1.2	28	0	31	15.82	35.26	15.31	5.83
261	56	2	0	1	87.5	28.33	1.2	16	1	42.5	24.23	32.77	13.64	6.61
262	54	2	0	0	71	19.84	2	37	0	27.4	14.40	38.20	16.87	6.28
263	68	2	0	0	85.5	24.49	1.2	14	1	40.1	21.30	31.87	11.40	5.17
264	61	2	0	0	97	32.80	1.5	22	0	43.3	31.89	41.68	16.85	7.5
265	55	2	0	0	70	22.18	1.5	29	0	40.2	22.22	33.02	14.39	5.6
266	74	1	3	0	91	25.87	1.2	27.5	1	33.6	23.37	46.16	19.54	7.21
267	62	2	0	0	73.5	21.59	2	17	1	35.8	18.41	32.96	13.19	5.56
268	83	1	3	1	85	21.94	1.5	26	1	26.3	15.16	42.53	18.94	7.29
269	77	1	3	1	96.5	26.20	1.5	28	1	25	17.59	52.72	24.04	9.26
270	55	1	1	1	83	21.27	3	40	0					
271	58	2	0	1	73	21.73	2	21	0	31	16.01	35.57	14.23	5.97
272	55	1	0	1	91	28.33	2	49	0	33	25.01	50.83	22.72	8.28
273	65	1	1	0	90	25.31	1	45	1	28.4	19.26	48.56	20.97	7.59
274	72	2	0	0	85.5	27.07	1.5	22	0	41.3	24.37	34.65	15.04	6.68
275	60	2	0	0	77	24.63	1.5	18	0	33.8	17.34	33.98	14.83	6.96
276	73	2	0	0	85	27.79	1.2	26	0	42.1	28.03	38.52	16.48	6.89
277	82	2	0	0	93	23.95	1.2	16	2					
278	51	1	0	1	82	23.86	3	48	0	17.7	10.37	48.30	21.81	8.57
279	73	1	1	0	87	24.83	1.2	38	1	30.6	20.26	45.89	18.77	6.84
280	72	1	1	0	91.5	25.87	1.5	29.5	1	31.7	21.34	46.01	19.42	7.29
281	56	1	1	1	76	19.17	2	41	0	14	7.47	45.86	19.30	6.86
282	72	2	0	0	86	26.96	0.43	10	2	42.3	26.72	36.41	14.79	6.31
283	64	1	1	1	84	24.15	2	42	0	20.4	13.48	52.50	22.82	8.15
284	90	1	1	0	81	21.10	1.2	19	2	19.3	10.43	43.61	18.38	7.26
285	60	2	0	0	66	22.23	1.5	16.5	1	32.1	13.98	29.61	12.07	5.93
286	78	1	1	0	93	24.46	1.5	35	0	26.9	19.86	53.99	25.34	8.16
287	65	2	0	0	77	22.92	1.5	20	0	33.1	18.39	37.15	15.82	6.59

288	75	1	3	0	76	18.97	1.5	31	0	9.2	4.58	45.00	18.96	7.21
289	86	1	1	0	85.5	23.62	1.2	38	1	28.6	17.02	42.53	19.02	7.33
290	66	2	0	0	84.5	25.51	1	25	1	40.6	25.22	36.98	14.82	6.06
291	70	1	1	0	81	24.00	1.5	41	0	13.8	9.66	60.25	26.60	8.93
292	58	2	0	1	81	24.63	1.5	27	1	31	18.06	40.15	17.75	7.4
293	60	2	0	1	73	23.23	1.5	22	1	43.9	24.07	30.77	12.47	5.29
294	53	1	1	1	90	27.05	1.5	42	1	31.1	22.00	48.64	21.38	8
295	69	1	1	1	99	27.37	1.5	38	1	34.4	25.87	49.34	21.34	7.56
296	72	2	0	0	94	24.67	0.5	12	2	40.2	21.91	32.63	13.04	5.91
297	84	1	1	1	82.5	22.26	2	32	0	22.1	12.00	42.28	18.41	7.48
298	80	2	3	1	73	16.07	1	5	2	20.3	6.52	25.65	9.53	4.64
299	55	1	1	1	79	20.84	2	38	0					
300	69	2	0	0	97	34.00	1	12	1	45.9	32.00	37.69	15.55	7.5
301	76	2	0	1	99	30.35	1.5	29	1	38.5	28.88	46.09	18.19	7.36
302	72	2	0	1	93.5	24.94	1.2	25	0	30.8	17.84	40.05	15.13	6.61
303	76	1	1	1	108	27.56	1.2	30	1	33.9	27.21	53.04	24.50	8.2
304	64	2	0	1	86	27.79	1.5	29	1	37.4	24.28	40.65	17.49	7.37
305	63	1	1	1	85.3	26.82	1.5	32.5	0	25.6	15.47	44.91	19.04	8.32
306	61	2	0	0	74	22.40	1.5	20	1	30.1	14.62	34.02	14.36	6.59
307	56	1	1	1	98	28.67	2	44	0	26.2	21.93	61.89	29.65	9.97
308	64	1	1	1	106	33.25	1.5	31	0	35.6	31.88	57.65	27.43	9.75
309	83	1	0	0	75	21.80	1.2	28	1	20.2	11.12	43.81	19.23	7.51
310	55	1	0	1	80.5	21.37	2	38	0	16.3	8.71	44.69	19.98	7.8
311	63	1	0	1	92.5	25.97	1.2	42	1	26.5	20.76	57.51	26.34	8.55
312	52	2	0	1	86	23.89	2	30	0	34	22.07	42.81	19.00	7.05
313	70	1	1	1	103	27.86	1	26	1	27.7	17.65	46.09	19.33	8.25
314	81	1	0	0	85	24.27	1.5	29	0	25.1	14.21	42.51	19.51	8.04
315	75	2	0	1	80	22.42	1.5	16	1	34.6	15.87	30.00	12.26	6.08
316	72	2	0	0	90	22.61	1.2	23	0	29.1	13.67	33.28	15.68	7.36
317	61	2	0	0	78	20.96	1.2	23	0	37.9	20.85	34.21	14.94	5.58
318	61	1	3	1	83	21.27	1.5	34	0	21.4	13.34	48.90	21.53	7.62
319	90	1	3	0	91.5	24.78	1	29.5	1	26.6	17.68	48.89	20.40	7.57
320	56	2	0	0	73.5	20.99	2	30	0	33	15.80	32.14	12.96	5.61
321	69	1	1	0	97.5	23.80	1.2	17	1	26.1	16.40	46.55	19.32	7.3
322	70	1	1	1	85	23.29	1.5	28	1	21.8	12.96	46.63	20.02	7.77
323	64	1	0	0	94	27.67	1.5	40	0	32.5	23.70	49.25	23.59	8.85
324	65	1	0	0	94	26.03	1.5	34	0	32.2	21.69	45.66	20.53	7.63
325	64	1	1	1	89	24.57	1.5	39	0	20.4	13.23	51.49	22.41	8.28
326	63	2	0	0	96.5	24.52	1	20	2	39.3	22.45	34.60	13.53	5.84
327	76	1	3	1	90.5	23.28	1.2	31	1	24	14.10	44.55	19.11	7.68
328	55	1	1	0	82	22.15	1.5	38	0	16.2	9.33	48.20	19.28	7.24
329	59	2	0	1	89	27.62	1.5	22	0	44.3	26.86	33.78	13.19	5.98
330	77	2	0	0	76	21.14	1.2	16.5	2	34.6	15.90	30.02	12.75	5.88
331	56	2	0	0	80	24.39	1.5	22	0	40.2	25.98	38.70	16.67	6.16
332	67	1	3	1	93	26.75	2	42	0	28.8	20.97	51.95	24.10	8.75
333	74	2	0	0	97	28.37	1	22	1	41.8	30.25	42.20	17.34	6.64
334	67	1	3	0	84	21.64	1.2	22	1	16.9	8.57	42.11	18.50	7.56
335	53	2	0	0	91	27.94	1.2	23	0	45.1	32.91	40.03	16.86	6.32
336	87	2	1	0	68	20.60	0.6	12	2					
337	62	1	0	1	89	23.76	2	32	0	24	14.94	47.45	22.04	8.19
338	78	1	3	1	77	19.98	1.2	26	1	18.5	8.60	37.83	14.42	5.94
339	62	2	0	1	95	31.87	1.2	18	1	43.5	28.87	37.56	16.26	7.59
340	73	2	0	0	116	36.75	0.55	20	1	45.1	39.83	48.55	20.87	8.52
341	73	1	3	1	88	23.95	2	25	1	28.3	17.04	43.17	18.70	7.11
342	63	1	3	1	100	26.34	1.5	34	0	27.3	20.21	53.75	23.18	7.92
343	62	2	0	1	77	21.71	2	18	1	28.7	14.71	36.61	15.78	6.43
344	60	2	0	0	86	28.15	0.86	27.5	1	47.8	29.86	32.64	13.31	5.85
345	76	1	0	1	102	32.01	1	20	2	36.4	28.77	50.22	21.99	8.89
346	66	1	0	1	100	28.81	2	51.5	0	33.8	25.61	50.09	22.65	8.22
347	81	1	1	0	94	24.83	1	26	2					
348	81	1	0	0	96	28.85	1	35	1					
349	83	1	3	1	105	30.59	1.2	30	1	39.7	29.86	45.38	19.87	7.77
350	53	1	0	1	93	29.30	2	29	0	33.3	25.24	50.53	21.62	8.27
351	61	1	1	1	90	27.82	2	35	0	30.5	20.54	46.79	20.66	8.32
352	54	2	0	0	95.5	30.36	1.5	29	1	45.5	32.97	39.45	15.99	6.74
353	71	1	3	0	104	28.97	1.5	30	0	32.5	24.76	51.44	23.68	8.75
354	72	1	3	0	91	24.23	1.2	30	1	27.6	18.14	47.57	21.88	7.9
355	65	1	3	0	84	23.64	1.5	43	0	21.2	13.58	50.40	22.61	8.09
356	68	1	3	1	82	24.51	2	34	0					
357	83	1	1	0	74.5	19.45	1	26	2	15.9	8.18	43.40	18.94	7.08
358	82	1	1	0	82	20.18	1	24.5	1	12	5.48	40.06	16.07	6.74
359	68	2	0	0	90	27.21	1.2	21	1	40	23.19	34.81	14.51	6.81

360	52	1	3	1	84	21.29	2	42	1	22.7	13.84	47.14	21.63	7.16
361	73	2	0	0	91	25.13	1	19	1	40.2	23.76	35.37	14.08	5.91
362	67	2	0	0	91.5	26.67	1	15	1	40.6	24.03	35.13	15.08	6.66
363	65	1	3	0	86	22.88	1.2	31	1	26.9	15.00	40.79	17.80	7
364	79	1	3	1	77	18.96	1	21.5	1	15.2	6.49	36.13	15.68	6.27
365	64	2	0	0	95.5	29.33	1.2	28	1	42.9	30.28	40.34	17.67	7.28
366	53	2	0	0	77	21.48	1.2	30	0	33.6	17.83	35.21	15.83	6.28
367	61	2	0	0	73	18.23	1.5	19	1	23	8.97	29.96	11.61	5.12
368	77	2	0	0	78	20.04	1.2	19	0	20.8	9.14	34.85	14.61	6.76
369	57	1	0	0	77.5	23.22	1.5	36	0	21.4	12.09	44.49	20.40	8.22
370	63	2	0	0	90.5	25.94	1.5	26	0	40.4	26.76	39.47	15.73	6.14
371	73	2	0	0	82	24.11	1	20	1	34.9	19.79	36.94	15.39	6.53
372	63	1	3	1	99	28.40	2	46	0	29.4	24.51	58.90	27.09	9.12
373	63	1	0	1	87	22.55	1.5	34	0	20.7	11.68	44.88	19.70	7.69
374	76	1	1	1	104.5	28.78	1.2	31	1	32.8	23.07	47.38	20.20	8.16
375	62	1	1	1	80	23.78	1.2	29	1	22.4	12.30	42.65	18.33	7.73
376	57	1	3	1	93	27.05	2	50	0	32.2	25.59	53.89	23.69	7.85
377	70	2	0	0	104	34.81	1.2	20	0	44.4	34.92	43.77	17.75	7.74
378	58	1	1	0	71	19.94	1.5	34	0	8.4	4.02	43.71	19.21	8.1
379	61	2	0	1	86.5	24.97	1.2	30	0	38.1	22.56	36.70	15.21	6.3
380	52	1	0	1	106	31.24	2	40	0	35.3	30.74	56.24	26.07	9.16
381	65	1	3	1	83	21.91	1.5	39	0	14.8	7.93	45.62	20.21	7.9
382	53	1	0	1	99	29.87	2	45	0	30.1	24.48	56.78	25.87	9.32
383	57	1	3	1	115.5	38.97	2	42	0	37.2	36.27	61.14	22.60	8.86
384	75	1	3	0	86	24.17	0.67	29	1	19.9	12.67	50.92	22.89	8.51
385	59	2	0	0	89	26.47	2	17	1	41.8	22.23	30.96	12.67	5.97
386	73	2	0	1	84	26.15	1.2	22	0	36.3	20.38	35.75	15.37	6.9
387	57	2	0	0	77	25.39	1.5	28	0	38.2	22.94	37.13	16.85	6.89
388	75	2	0	0	93	27.05	1.2	22	0	37	20.95	35.70	14.55	6.62
389	52	1	1	1	90	22.02	1.5	28	2	24.8	15.68	47.58	20.49	6.77
390	82	1	1	0	88	23.65	0.6	28	1	22	13.00	45.97	20.63	8.08
391	52	1	1	1	94	25.02	1.5	35	0	25.4	18.64	54.62	26.21	8.63
392	89	1	3	0	74	18.96	1	21.5	2	15.5	7.35	40.08	17.69	6.86
393	73	2	0	0	81	20.13	1.2	14	1	26.1	10.33	29.30	11.73	5.74
394	70	1	1	1	94.5	20.29	1.2	28	1					
395	64	1	1	1	80	20.90	1	34	1	10.3	4.77	41.38	18.09	7.72
396	65	1	1	0	93	29.58	2	45	0	30.5	25.39	57.75	27.61	9.55
397	83	2	0	0	101	32.50	0.75	18	2					
398	91	1	3	0	96	27.24	0.67	20	1	16.8	9.49	46.90	20.38	9.77
399	77	1	3	0	95	25.71	1	20	1	34	21.31	41.30	17.04	6.26
400	65	1	1	0	95	24.70	1.2	39	1					
401	82	1	3	0	90	23.42	0.86	18	2	21.3	12.84	47.49	21.02	7.82
402	58	1	3	1	101	30.34	1.5	41	1	37	29.78	50.80	23.20	8.38
403	74	1	1	1	81.5	22.15	2	27	1	23.9	13.48	42.84	18.31	6.88
404	55	1	1	1	85	23.18	3	40	0	28.5	18.22	45.63	19.66	6.88
405	55	2	0	0	93	29.41	3	24	0	42.5	28.67	38.72	16.73	7.2
406	73	2	1	1	94	31.59	1	21	1	44.2	28.04	35.43	14.44	7.02
407	63	2	0	0	70	22.74	1.2	21	0	26.3	12.96	36.24	15.61	7.13
408	59	2	0	1	79	23.58	2	25	0	33.2	16.28	32.77	13.39	6.15
409	57	2	0	0	74	25.58	2	24	0	49.6	31.44	31.96	14.17	5.61
410	58	2	0	0	102.5	30.05	0.86	20	1					
411	79	1	0	0	83.5	21.16	1.2	23	2	17.7	8.68	40.23	17.17	7.14
412	53	2	0	1	70	19.78	2	22	1	28.3	13.37	33.83	14.06	5.81
413	72	2	0	1	97	29.35	1.2	19.5	0	45.5	34.80	41.71	18.38	6.75
414	58	1	1	0	88.5	26.03	1.5	32	0					
415	54	2	0	0	86.5	26.87	1.5	22	0	36.3	22.11	38.83	16.57	7.18
416	83	2	0	0	106	30.06	0.6	10	1	40.5	28.58	41.99	16.97	7.03
417	56	1	0	0	93	26.53	2	47	0	26.2	19.13	54.01	24.84	8.93
418	59	1	0	1	81	22.67	1.5	36	1	25.3	15.70	46.27	21.78	7.88
419	54	1	1	1	89	24.36	1.5	40	1	25	15.95	47.86	20.43	7.54
420	57	2	0	0	89.5	28.54	1	24	1	46.6	30.21	34.55	14.63	6.18
421	61	1	3	1	89	24.09	1.5	42.5	0					
422	54	2	0	1	69	18.18	1.2	20	0	25.1	10.51	31.32	13.20	5.48
423	59	2	1	1	88	27.37	1.5	29	0	36	23.27	41.39	16.74	6.88
424	60	2	0	1	99	32.89	1.2	21	0	48.5	36.98	39.19	15.73	6.8
425	58	2	0	1	70	20.06	1.5	21	0	30.8	15.47	34.78	14.55	5.73
426	68	1	3	0	90	25.29	1.5	37	0	21.4	15.10	55.40	25.25	8.96
427	59	1	0	0	95.5	30.48	2	36	1					
428	70	1	1	1	82	21.08	1.5	34	0	19.2	11.27	47.33	21.00	7.44
429	72	1	0	1	90	24.10	1	32	1					
430	69	2	0	0	86	24.29	0.67	17	2					
431	63	1	3	1	80	23.31	1.2	44	1	27.1	16.33	43.93	19.88	7.55

432	58	2	0	0	77.5	25.02	1.5	30	0	38	22.34	36.44	16.35	6.76
433	53	2	0	0	87	28.96	1.5	24	1	47.8	32.23	35.23	15.02	6.34
434	58	1	1	0	95	26.12	0.86	21	1	33	22.39	45.46	19.73	7.44
435	55	1	1	1	90	25.40	1.2	40	1	26.7	19.10	52.41	21.74	7.58
436	77	2	0	0	77	23.56	0.86	16	1	30.7	14.27	32.22	12.86	6.4
437	81	1	3	1	79.5	22.05	1.5	19	1	21.6	11.36	41.26	17.97	7.27
438	71	2	0	0	69.5	20.48	1.5	17	1	24.4	11.66	36.14	15.14	6.5
439	66	2	0	0	78	22.59	1.2	31.5	0	34	17.63	34.30	15.19	6.41
440	84	1	3	1	95	26.98	1	30	1	34.6	21.90	41.43	17.45	7.19
441	58	1	0	0	86	25.79	1.5	41	0	27.8	17.93	46.58	20.91	8.32
442	71	2	0	0	106	28.41	0.75	14	2	41.2	26.23	37.44	14.71	6.4
443	60	1	0	0	83	22.97	1.5	38	0	19	12.48	53.12	23.69	7.76
444	62	2	0	1	84	28.11	1.5	21	0	47.4	29.17	32.34	13.52	6.09
445	89	1	1	0	92	23.87	1.2	26	2					
446	63	1	3	1	88	27.38	1.5	39	1	32.7	22.51	46.24	20.32	7.7
447	74	2	0	0	71.5	20.78	1	20	1	31	13.55	30.15	12.27	5.72
448	52	2	1	1	83	26.52	1.5	20	0	45.5	27.69	33.21	14.64	6.26
449	72	2	0	0	82	22.58	1	20	1	32.9	16.19	33.03	14.57	6.53
450	59	2	0	0	78	27.47	1.2	25	0	31.9	16.97	36.23	15.47	7.77
451	61	2	0	0	79	24.14	2	19	0	40.1	20.63	30.83	13.15	5.96
452	60	1	0	1	74	19.76	3	41	0	18.7	9.43	41.03	18.59	7
453	55	2	0	1	83	27.16	3	35	0	42.9	24.53	32.62	13.78	6.29
454	79	1	1	1	104	25.76	0.86	31	1	33.3	24.85	49.84	22.79	7.62
455	56	2	0	1	86.5	29.32	1.5	30	1	39.4	25.25	38.85	16.01	7.21
456	64	1	0	1	81	24.77	1.5	42	1	19	12.76	54.42	25.18	9.04
457	57	1	1	1	86.5	25.56	1.5	29	0	25.5	18.27	53.27	23.19	8.05
458	75	2	0	1	84.5	24.31	0.86	20	1	38.8	22.68	35.85	14.42	5.68
459	72	1	0	0	75	20.81	1.5	34	0	25.6	13.32	38.75	17.81	6.81
460	58	2	0	0	74.5	24.09	1.2	12	1	46.4	22.98	26.55	11.62	5.49
461	59	1	0	1	95.5	28.78	2	36	0	27.6	20.75	54.57	26.80	9.97
462	56	1	1	1	76.5	20.77	2	44	1	19.4	10.83	45.06	20.21	7.33
463	63	2	1	1	95	27.79	2	20	0	38.9	25.27	39.66	16.50	7.26
464	51	1	1	1	86	26.03	3	46	0	26.6	20.12	55.47	24.95	8.43
465	53	2	0	1	77	21.45	2	25	0	36.2	18.47	32.62	14.13	5.76
466	66	2	0	1	81.5	22.51	1	26	1	34.8	18.60	34.85	14.56	5.96
467	54	2	0	1	78	24.04	2	33	0	37.3	21.75	36.63	16.19	6.48
468	58	2	0	0	90	27.37	1.5	25	0	42.4	28.48	38.67	16.28	6.5
469	57	2	0	0	71	21.67	2	20	0	33.1	17.04	34.50	13.24	5.47
470	61	1	3	0	105.6	31.76	1.5	35	0	34.4	28.51	54.30	22.61	8.49
471	53	1	3	1	87	27.96	2	59	0	20.9	17.39	65.81	29.53	9.91
472	72	1	0	0	115.5	30.23	0.75	29	2	36.7	28.20	48.55	21.65	8.06
473	55	2	0	0	78.5	21.22	1.5	28	0	37.7	18.74	30.94	13.37	5.44
474	68	2	1	0	72.5	20.93	1.5	23	1	26.8	11.94	32.56	12.49	5.63
475	63	2	0	1	90	29.90	1.5	18	0	42.3	25.85	35.24	14.41	6.95
476	67	1	3	0	75.5	22.50	1.5	37	0	19.4	10.27	42.61	19.26	7.86
477	78	1	3	1	81	18.88	1.2	25	1	20.3	9.93	38.93	15.97	6.87
478	59	2	0	0	87.5	26.97	1.5	22	0	36.7	25.18	43.34	18.86	7.28
479	68	2	0	0	85	26.34	1.2	20	1	42.1	25.36	34.85	14.52	6.22
480	57	1	3	1	104	32.22	1.5	43	0	36.2	32.13	56.66	25.39	8.92
481	58	1	0	1	86	25.54	2	43	0	18.3	13.01	58.24	25.75	9.14
482	57	2	0	0	78.5	23.96	1.2	20	1	42.8	21.99	29.42	11.72	5.45
483	79	2	0	0	75	21.24	0.67	13	2	28	14.07	36.12	14.92	6.22
484	70	1	0	0	93	26.50	1	32	1	31.7	23.13	49.75	23.12	8.29
485	79	1	1	0	96	26.70	0.75	19	1	25.4	16.84	49.45	20.76	8.21
486	69	1	0	0	81	23.31	2	30	0	24.2	13.74	42.93	19.46	7.87
487	78	1	3	1	67.5	17.32	1	25	1	14.2	6.02	36.22	12.81	4.79
488	71	2	0	1	67	17.04	0.75	14	2	21.9	8.15	29.08	11.52	5.1
489	53	1	0	1	88	25.14	2	53	1	17.8	12.36	56.94	26.27	9.34
490	52	2	0	0	95	34.46	1.5	24	0	47.4	37.27	41.35	17.60	7.68
491	56	2	0	0	73	21.91	1.5	19	0	40.5	21.02	30.88	13.03	5.31
492	54	1	1	1	77	22.25	2	41	0	21.5	12.18	44.39	18.39	7.01
493	55	2	0	0	64	19.53	1.5	22	1	30.5	15.36	32.79	13.93	5.7
494	59	2	0	1	76.5	23.74	1	17	1	33.5	18.05	35.79	14.41	6.37
495	63	1	3	1	80	22.47	2	41	0	18.5	11.28	49.70	22.34	8.1
496	56	2	0	0	91	33.73	1.5	25	1	51	39.78	38.24	16.00	6.72
497	72	2	0	0	77	25.01	1.2	16	1	36.1	18.76	33.17	14.20	6.69
498	53	1	1	1	82	24.15	2	42	0	27.2	17.38	46.53	20.83	7.58
499	52	2	0	0	84.5	26.77	2	24	0	44.7	29.12	36.04	15.92	6.36
500	59	2	0	1	70	23.40	1.5	25	1	33.3	17.67	35.34	14.88	6.39
501	52	2	0	0	98	34.47	2	21	0	46.9	37.57	42.56	16.96	7.22
502	79	2	0	0	99	33.32	0.55	19	2					
503	63	2	0	0	80	26.21	1	21	1	38.7	22.62	35.88	14.83	6.57

504	53	2	0	0	74	25.27	1	21	2	39.4	23.46	36.08	16.04	6.67
505	76	1	3	0	89	23.96	1.5	33	1	31.4	20.71	45.21	19.20	6.58
506	64	1	3	1	92	24.87	1.5	45	0	29.1	20.61	50.33	22.91	7.88
507	57	1	1	1	71.5	22.84	1.5	45	0	10.6	7.13	60.15	28.03	9.29
508	63	2	0	0	78.5	25.05	1.2	22	0	41.6	25.72	36.05	16.34	6.5
509	54	1	1	0	80.5	22.34	1.5	29	0	27	15.38	41.54	18.33	7.04
510	57	1	1	1	98	27.84	2	38	0	33.9	25.38	49.44	20.94	7.58
511	58	1	3	1	99	32.12	1	25	2	33.8	27.87	54.56	23.32	8.9
512	62	2	0	0	94	28.43	1.5	38	0	38.7	27.53	43.52	17.81	6.97
513	61	2	0	0	86.5	24.72	1.5	17	1	42.4	21.99	29.90	12.24	5.74
514	57	2	0	1	70	19.70	2	18	0	29.5	13.03	31.12	13.60	5.98
515	74	1	3	1	97	26.99	1.5	33	0	26.6	19.29	53.10	23.66	8.72
516	54	2	0	0	76	21.42	1.2	29	1	34.6	19.20	36.28	16.60	6.3
517	60	2	0	0	71	22.33	2	24	0	31.1	15.78	35.05	15.49	6.71
518	59	2	0	0	86	25.01	2	17	1	36.7	25.61	44.25	20.82	7.44
519	51	1	0	1	98.5	33.33	1.2	41	2					
520	80	1	0	1	91.5	24.44	2	29	0	30.7	20.19	45.47	20.06	7.58
521	65	1	1	1	78	21.58	2	25	1	14.4	7.12	42.33	18.75	7.49
522	72	1	3	1	96	26.98	1.2	28.5	1					
523	57	2	0	1	71.5	18.19	2	14	1	29.8	11.89	27.95	11.47	5.15
524	74	1	0	1	102	29.13	1	37.5	1	27	23.75	64.26	28.84	9.42
525	55	1	1	0	88.5	25.69	2	37	1	23.5	16.29	53.10	24.01	8.79
526	85	1	0	1	91	26.41	1.2	29	1	25.5	16.76	48.85	22.40	8.82
527	55	2	0	0	106	35.01	1.5	26	0	48.9	42.35	44.24	15.35	6.14
528	67	1	0	0	89.5	25.57	0.86	27	1	30	19.87	46.37	20.73	7.83
529	60	1	0	1	103	28.68	3	42	0	35.3	28.76	52.68	21.56	7.54
530	58	1	0	1	85	22.00	2	42	1	24.4	14.69	45.61	20.52	7.34
531	69	2	0	0	84	24.79	1.2	25	0	37.5	20.31	33.81	13.68	6.15
532	57	1	1	0	79	21.84	1.2	27.5	2	21.5	12.21	44.50	17.53	6.42
533	65	1	1	1	80	22.94	2	36	0	23.2	13.85	45.79	20.45	7.6
534	69	1	1	0	89	25.88	1.5	36	0	26.3	16.84	47.17	19.70	7.78
535	60	2	0	1	83.5	24.62	2	32	1	33.4	18.31	36.54	16.41	7.25
536	55	2	0	1	74	21.72	1.5	18	0	36.2	18.45	32.56	13.69	6.02
537	56	2	0	0	95.5	28.08	1.5	22	0	44.4	27.73	34.78	14.85	6.3
538	70	2	0	0	84.5	21.20	1.2	15	1	36	17.54	31.15	12.98	5.58
539	64	2	0	0	77.5	24.18	1.2	23	1	39.5	20.10	30.83	13.17	6.22
540	59	2	0	1	79	23.24	1.2	21	0	40.8	23.64	34.28	14.27	5.74
541	80	2	0	0	73	18.77	1.2	20	0	28.3	12.07	30.62	12.60	5.38
542	71	1	1	0	86	23.33	2	36	1	21.4	11.53	42.28	18.72	8.03
543	75	1	0	0	97	30.61	1.5	19	1	36	26.15	46.55	20.75	8.54
544	52	1	3	0	78.5	20.49	2	47	0	14	8.58	52.57	23.89	7.95
545	62	2	0	0	64	20.69	1.5	24	1	32.7	15.16	31.20	13.08	5.78
546	74	2	0	0	82	27.30	1	19	1	34.9	18.61	34.70	14.14	7.12
547	54	1	0	0	92.5	26.15	1.5	25	1	34.7	25.53	47.94	22.55	7.83
548	68	2	0	0	78.5	21.27	1.5	31	0	31.8	16.49	35.38	15.25	6.06
549	52	2	0	0	63	19.70	1.2	31	0	29.5	13.34	31.93	13.08	5.46
550	52	1	0	1	92	28.95	3	47	0	32.8	26.30	53.77	24.67	8.76
551	54	2	0	1	70	18.79	2	31	1	27.7	13.38	34.91	15.23	6.02
552	75	1	0	0	78	22.28	0.86	28	1					
553	71	1	1	1	83	22.55	1.5	26	1	22.6	12.17	41.57	17.56	7.32
554	67	1	1	0	84	23.54	1	34	1	21.8	13.73	49.09	21.90	8.14
555	86	1	0	0	95	25.60	2	30	0	38.5	27.25	43.61	18.30	6.49
556	58	1	0	1	81.5	21.75	1.5	50	0	22.3	13.08	45.58	20.79	7.6
557	59	2	0	0	84.5	24.73	1.5	32	0	35.3	22.57	41.36	19.30	7.41
558	71	1	0	0	96	25.09	2	23	1	24.5	17.94	55.26	24.09	8.13
559	63	1	0	1	77.5	23.79	1.2	38	1	17.2	10.26	49.56	21.11	7.58
560	76	2	0	0	100	34.39	1	17	2	44	30.80	39.17	13.46	6.45
561	62	1	0	0	84	27.65	1.2	43	1	29.6	19.02	45.35	17.32	7.06
562	59	2	0	0	69	20.45	1.5	18	0	33.8	17.67	34.61	14.97	5.78
563	79	1	1	0	71.5	19.61	1.5	28	1	16.1	7.72	40.31	17.31	6.83
564	59	2	0	1	78	22.52	1.5	24	0	45.9	25.11	29.56	13.03	5.26
565	74	2	0	0	89	25.88	1	23	1	38	25.62	41.80	19.54	7.44
566	54	1	0	1	86	22.91	3	47	0	15.9	10.70	56.73	27.21	9.21
567	84	2	0	0	93	25.13	0.5	16	2	32.1	17.93	37.99	16.00	7.25
568	69	1	0	1	84	22.51	1.5	41	0	17.9	12.84	58.90	25.86	8.01
569	71	2	0	1	82	24.30	1	18	1	32.5	16.24	33.78	14.25	6.84
570	59	2	0	0	70	21.77	1.5	22	1	35.6	16.96	30.63	12.80	5.81
571	69	1	1	1	83	22.15	1.5	30	0	19.4	9.64	40.08	15.21	6.67
572	64	2	1	0	89	27.85	1.5	20	0	46.9	28.60	32.42	12.84	5.86
573	59	1	0	1	100	30.39	1.5	31	0	39.9	27.91	42.11	18.67	7.77
574	63	1	3	1	96	26.36	2	35	0	31.7	23.59	50.79	20.99	7.44
575	60	2	0	0	78	26.54	1.2	34	0	43	27.19	35.97	15.76	6.43

576	67	1	1	1	98	28.55	1.5	33	1	31.3	21.99	48.35	19.40	7.8
577	50	1	1	0	96.5	28.34	1.5	44	0	33.7	28.34	55.87	24.82	8.24
578	62	2	0	0	90	25.31	1.5	27	1	44.7	29.48	36.45	15.73	6.06
579	63	1	1	0	92	25.51	1.5	27	1	23.4	14.60	47.73	20.54	8.29
580	56	1	0	0	91	28.55	1.5	40	0	26.5	20.07	55.77	28.47	10.42
581	56	1	3	0	83	23.82	2	50	0	19.6	12.93	52.99	26.16	9.3
582	78	1	3	1	69.5	18.96	2	25	1	16.3	7.20	37.03	15.30	6.64
583	59	1	3	0	88	26.76	1.5	42	0	21.8	13.16	47.28	21.42	9
584	74	1	0	1	84	21.04	1.2	24	1	21.5	12.15	44.46	18.81	6.83
585	54	1	0	0	90	27.78	1.5	49	0	27.5	20.44	53.80	25.39	9.43
586	57	2	1	1	84.5	24.39	2	26	1	40.6	21.98	32.20	13.39	5.96
587	63	2	0	0	79	20.75	1.5	23	0	35	17.93	33.35	13.48	5.42
588	59	1	0	1	94	25.99	2	44	0	25.2	18.85	56.03	24.94	8.53
589	66	2	0	0	75	23.94	1.5	24	0	34.2	19.91	38.36	16.48	6.69
590	53	1	1	1	114	33.08	1.5	46	1	31.6	30.43	65.71	28.61	9.56
591	60	1	3	0	97	31.09	1.5	38	1	29.3	22.41	54.00	24.76	9.83
592	55	2	0	0	81	25.66	1.2	24	0	36.7	22.24	38.37	15.25	6.37
593	59	2	0	0	78.5	22.79	2	31	0	34.3	18.33	35.05	14.22	5.98
594	61	2	0	0	69	21.37	1.2	20	1	33.3	15.95	31.88	13.54	6
595	72	1	3	0	98	27.48	1.5	34	0	28.6	19.82	49.50	22.30	8.67
596	54	2	0	0	79	19.88	1.5	23	0	28	13.31	34.15	14.44	5.96
597	58	2	0	0	96.5	25.38	1.5	24	0	42	24.79	34.20	14.96	6.31
598	57	2	0	0	84	25.36	1.2	30	0	30.6	17.25	39.20	17.37	7.65
599	59	1	0	0	90	28.20	2	41	0	27.5	20.34	53.63	25.36	9.52
600	54	1	1	0	76	19.51	1.5	35	0	17.7	8.71	40.44	16.80	6.43
601	68	1	3	0	88	24.00	2	22	1	24.8	16.18	49.17	22.24	8.01
602	82	1	3	0	96	26.56	0.67	28	1	32.1	19.83	41.91	19.10	8.05
603	81	2	0	0	94	29.48	1.5	23	0	46.5	29.37	33.75	13.63	6.18
604	70	1	1	0	84	23.18	1.5	43	0	18.6	11.62	50.80	22.83	8.38
605	69	1	1	0	97	25.70	2	36	0	22.1	15.34	54.08	25.20	9.13
606	62	1	3	1	90	26.62	1.5	44	0	31.8	27.03	57.91	27.78	8.47
607	63	1	1	1	74	21.65	2	37	0	11	5.70	46.30	20.45	8.25
608	61	2	0	0	70	21.66	1.5	25	0	32.5	15.56	32.32	13.40	5.92
609	61	1	0	1	93	26.61	2	41	0	28.2	20.29	51.78	23.69	8.48
610	52	2	0	1	91	28.09	1.5	22	0	40	26.61	39.87	16.71	7.03
611	58	2	0	0	77	23.81	1.2	18.5	0	35.7	17.45	31.50	13.23	6.1
612	63	1	3	0	83	23.96	2	40	0	27.9	16.74	43.21	18.50	7.29
613	67	1	0	1	80	23.05	1.5	34	0	20.5	11.90	46.18	20.49	7.81
614	53	2	0	0	59	18.71	1.5	20	0	31.7	11.67	25.11	10.59	5.33
615	58	1	1	1	99.5	31.11	2	32	0	33.6	23.28	45.91	19.23	8.55
616	74	1	1	0	101	33.50	1.2	41	1	37.8	28.63	47.10	20.21	8.36
617	74	1	0	0	71	19.80	1.2	36	1	11.5	5.98	45.79	19.87	7.71
618	56	1	0	1	100	28.54	2	29	0	26.4	21.51	59.92	28.67	9.99
619	70	2	0	0	81	25.00	1	17	2	36.9	22.50	38.42	17.00	6.93
620	78	1	3	0	120	27.53	0.5	17	2	41.5	34.10	48.01	20.95	6.82
621	62	2	0	0	100	29.75	2	30	1	41.9	32.74	45.50	19.43	7.45
622	57	2	0	0	75	20.10	2	30	0	35.3	17.67	32.39	13.87	5.46
623	57	2	0	0	74	20.44	1.2	25	0	35.1	17.22	31.80	13.23	5.51
624	53	2	0	1	78	23.52	2	28	0	30.6	17.36	39.34	16.69	6.91
625	52	1	1	1	93	28.01	2	42	1	32.1	23.73	50.16	23.26	8.73
626	76	2	0	0	77	22.01	1.2	19	0	27.2	12.35	33.08	14.16	6.92
627	61	2	0	0	80	21.50	2	18	0	34.7	18.28	34.46	15.13	6.14
628	65	1	0	1	75	22.41	2	42	0	21.4	12.61	46.36	20.48	7.52
629	92	1	3	0	83	22.50	0.86	30	1	26.1	15.18	43.06	19.42	7.45
630	57	2	0	0	82	20.98	2	24	0	35.7	18.86	33.97	15.21	5.82
631	68	1	0	0	75	21.74	2	42	0	16.7	9.35	46.48	20.76	7.86
632	57	2	0	1	85.5	24.79	1.5	17	1	47.7	27.07	29.68	11.80	5.01
633	74	1	1	0	98.5	29.47	1.2	32	1	33.5	26.73	52.96	23.40	8.41
634	64	1	1	1	77.5	20.34	2	45	0	19.1	11.81	50.14	21.74	6.93
635	54	1	0	0	76.5	22.04	1.5	40	0	18.7	9.90	43.05	19.13	7.82
636	57	2	0	0	91	26.76	1.2	26	1	41.7	26.92	37.66	16.86	6.86
637	63	1	0	0	104	31.68	0.86	32	1	39.6	31.35	47.80	20.04	7.85
638	62	2	0	0	73	22.24	1.2	21	0	35.7	19.32	34.72	13.77	5.61
639	55	1	1	0	82	21.69	2	43	0	18.1	10.91	49.27	22.77	7.99
640	52	2	0	1	95	22.69	2	28	0	39.9	22.42	33.71	13.74	5.44
641	61	1	1	0	82	24.00	1.5	32	0					
642	61	1	1	1	86.5	26.02	1.5	27.5	1	22.8	14.76	50.05	21.86	8.4
643	58	1	0	0	72.5	23.45	1.5	38	0	22.4	13.84	47.89	21.25	7.83
644	52	1	1	0	84	22.09	1.5	39	0	25.9	14.65	41.83	18.89	7.27
645	62	1	0	1	93.5	27.81	1.5	47	0	27.6	21.14	55.45	24.83	8.63
646	62	2	0	0	68	18.84	1.5	24	0	32.1	14.01	29.60	12.65	5.32
647	53	1	0	1	92	25.52	1.2	44	1	31.9	22.60	48.13	22.26	8

648	58	2	0	0	82	24.81	1.5	22	0	42.2	25.79	35.28	15.18	5.96
649	71	2	0	0	86.5	23.71	1.2	17	1	31.4	17.21	37.54	16.03	6.81
650	54	2	0	1	85	26.23	1.5	21	0	38.8	25.21	39.81	16.96	6.76
651	58	2	0	0	76	21.05	2	23.5	0	35.1	17.24	31.92	13.41	5.67
652	70	2	0	0	82	26.73	1.2	19	0	40.5	24.40	35.90	14.64	6.16
653	61	1	1	1	86	24.51	1.5	41	0	23.6	14.06	45.49	19.93	7.9
654	83	1	0	0	103.5	27.32	1.5	29	0	35	25.08	46.66	18.72	7.2
655	74	1	1	0	87	24.32	1.5	37	0	20.5	13.11	50.77	23.06	8.47
656	55	2	0	0	94.5	32.30	1.5	21	1	47.4	38.97	43.33	19.00	7.4
657	64	1	0	1	91	23.57	2	36	0	28.1	18.22	46.74	19.66	7.43
658	57	2	0	0	110.5	32.79	1.5	27	1	48.7	41.53	43.77	17.91	6.77
659	87	1	3	0	92.5	23.67	1	28	1	30.8	19.09	42.88	17.90	6.67
660	62	2	0	0	79	20.46	1.5	23	0	28.4	12.46	31.35	11.95	5.47
661	61	2	0	0	86.5	25.54	1.5	20	0	40.4	23.91	35.32	14.44	6.14
662	57	1	0	1	77.5	23.59	2	40	0	20	12.60	50.25	23.77	8.68
663	69	1	1	0	105	29.55	1.5	43	0	33.7	28.72	56.63	23.72	8.11
664	55	2	0	0	87	25.68	1.5	22	0	41	24.20	34.85	14.66	6.1
665	69	1	1	1	91	26.58	1.2	36	1	24.9	17.18	51.76	22.50	8.26
666	67	1	0	0	77.5	22.44	1.5	41	0	13.3	7.98	52.21	24.12	8.79
667	71	1	0	1	84	21.70	1.5	29	0	27.9	16.45	42.58	18.43	6.51
668	71	2	0	0	74.5	22.31	1.5	23	0	33.9	17.27	33.63	14.43	6.42
669	69	2	0	0	72	21.95	1	20	1	37.5	17.22	28.69	11.39	5.31
670	71	1	1	1	97	27.30	1.5	30	1	30.4	21.77	49.95	22.24	8.15
671	61	1	3	0	91	26.94	1.5	46	1	31.9	23.37	49.89	23.47	8.27
672	73	2	0	0	113	31.69	0.6	25	1	44.7	33.24	41.18	16.63	7.16
673	55	2	0	0	92	29.30	1.5	29	0	43.7	29.42	37.86	15.51	6.55
674	56	2	0	0	77.5	22.26	1.5	22	0	38.2	20.24	32.77	14.28	5.88
675	73	2	0	0	107	32.38	1.5	15	1	38.4	29.67	47.56	18.37	7.64
676	52	1	0	0	82	25.67	1.5	43	0	28.8	19.44	47.95	22.75	8.3
677	54	2	0	0	70	18.41	2	30	0	27.2	12.05	32.28	13.51	5.55
678	57	1	1	0	81	22.78	1.2	28	2	18.7	11.76	51.10	22.64	7.86
679	64	2	0	0	87.5	21.18	2	27	0	32.7	17.62	36.28	14.08	5.56
680	52	1	0	1	75	22.08	1.5	33	0	22.7	12.62	42.97	19.82	7.6
681	52	1	3	1	98	29.27	2	43	0	33.1	27.49	55.68	24.65	8.53
682	82	1	3	0	93	23.66	0.6	15	2	25.7	16.73	48.25	19.23	6.95
683	69	1	3	0	80	21.26	1.5	32	0	7.2	3.65	46.97	19.90	8.23
684	58	2	0	0	75	22.10	1.5	21	0	34.8	18.80	35.23	14.31	5.74
685	60	2	1	0	85.5	28.47	1.2	32	0	44.1	29.28	37.05	16.59	6.76
686	55	1	1	1	95.5	26.53	1.2	42	1					
687	55	1	3	1	96	30.11	1.5	40	1	26.8	20.79	56.64	25.82	9.76
688	54	1	1	1	78.5	21.67	3	35	0	15.7	9.18	49.24	22.12	7.88
689	59	2	0	0	86	26.87	1.5	29	1	38.7	24.97	39.62	17.47	7.12
690	72	1	0	1	103	26.72	1.5	30	0	26.7	18.77	51.44	21.67	7.91
691	63	1	3	1	98	28.66	1.5	30	0	33.3	26.31	52.82	21.72	7.74
692	77	1	0	0	86.5	22.70	2	30	0	18.3	11.24	50.22	22.39	7.83
693	57	1	1	1	86	25.78	2	45	0					
694	54	2	0	0	74	18.70	1.2	21	0	35.9	15.43	27.61	11.26	4.71
695	64	2	0	0	88	29.36	1.2	25	1	37.7	24.85	41.01	16.44	7.26
696	56	1	0	0	84	25.20	2	38	0	32.4	21.71	45.32	20.48	7.57
697	63	1	1	0	97	27.52	1.5	34	1	32.8	22.28	45.67	20.55	8.07
698	54	2	0	0	86	28.63	1.5	25	0	45.3	31.62	38.26	17.24	6.85
699	68	2	0	0	92	23.53	1.5	17	1	33.7	17.08	33.58	13.95	6.16
700	62	2	0	0	82	23.19	1.5	22	0	38.4	19.17	30.70	13.10	5.93
701	54	1	3	1	78.5	22.48	2	52	0	19.4	12.01	49.84	23.55	8.24
702	59	1	1	1	80	21.60	1.5	37	0	15.7	8.92	48.01	20.89	7.81
703	59	1	3	1	99.5	29.15	1.5	47	1					
704	61	1	0	1	86.5	26.14	2	44	1	25	18.94	56.81	24.60	8.38
705	76	2	0	0	66	21.55	0.75	17	1	38.3	16.02	25.82	9.61	4.7
706	57	1	1	1	75	20.70	1.5	35	1	16.5	9.10	45.92	19.97	7.4
707	54	1	0	1	93	27.66	2	36	0	27.6	18.15	47.60	20.03	8.38
708	85	2	1	0	78	21.55	0.86	15	2	26.8	12.10	33.02	13.29	6.43
709	55	1	1	0	90	26.92	2	46	1	26.2	19.18	53.92	24.35	8.54
710	66	2	1	0	114	31.59	1.5	22	0	46.1	32.75	38.35	15.38	6.71
711	85	2	0	0	87	21.99	0.67	13	2	25.1	11.21	33.49	12.20	5.95
712	77	2	0	0	98	28.38	1.5	14	1	35.9	22.28	39.72	16.49	7.43
713	74	2	0	0	82	28.17	1.2	17	1	41.9	23.88	33.16	12.99	6.26
714	70	1	3	1	87	25.25	1.2	30	1	25	15.83	47.38	20.54	7.95
715	72	2	0	0	80.5	23.49	3	23	0	38.6	20.24	32.25	13.70	6.02
716	74	1	3	0	81	22.99	1.5	36	1	25.1	14.21	42.29	17.58	6.75
717	68	2	0	0	86	23.78	1.5	21	1	42	23.55	32.46	13.19	5.4
718	69	2	0	0	79.8	24.90	2	18	0	38.1	20.98	34.10	13.63	6.08
719	80	2	0	0	74	22.69	1	23	1	36.8	18.61	32.00	13.26	5.88

720	77	2	0	0	82	23.71	0.67	20	1	33.7	17.65	34.70	13.90	6.28
721	79	1	3	0	89	24.97	1.5	31	1	26.8	16.62	45.41	20.77	8.1
722	76	2	0	0	94	27.50	1	16	2	42.9	24.53	32.66	14.00	6.42
723	71	1	1	1	73	19.82	1.5	25	1	21.7	10.35	37.30	15.39	6.45
724	74	1	1	0	88	24.68	2	42	0	28	16.92	43.60	18.47	7.3
725	70	1	3	0	103	26.89	1.2	19	2	35.8	23.66	42.36	17.95	7.05
726	72	2	0	0	85.5	26.38	1.5	26	0	37.7	22.08	36.51	15.31	6.87
727	76	1	3	0	90	26.70	1.5	19	1	32.4	21.77	45.51	19.86	7.8
728	79	1	1	0	84.5	21.87	0.75	31	2	21.7	12.34	44.66	18.82	7.05
729	70	2	0	0	80	23.79	2	17	1	36.8	21.31	36.65	15.48	6.3
730	78	1	0	0	95	26.77	0.6	34	1	32.3	22.90	48.06	21.54	7.83
731	73	1	1	1	83	22.63	1.5	23	1	24.8	12.37	37.60	16.17	6.83
732	77	2	0	0	74	19.87	0.86	11	1	28.6	12.46	31.09	12.88	5.89
733	74	2	0	0	101.5	26.37	1.2	20	0	30.8	18.35	41.13	15.99	6.91
734	76	1	1	0	88	22.97	1.2	20	1	24.4	14.37	44.51	19.18	7.38
735	69	2	0	0	64.5	18.77	1.5	18	0	17.2	7.47	36.06	14.04	6.14
736	73	1	3	1	98	29.38	2	34	0	28	21.44	55.17	25.47	9.36
737	68	2	0	0	88	24.71	1.5	21	0	26.6	14.79	40.90	15.61	6.65
738	67	1	3	1	83	24.10	1.5	42	0	21.8	13.64	48.90	22.51	8.56
739	65	2	0	0	71	19.81	1.5	22	0	31.8	14.85	31.88	13.87	5.87
740	78	2	0	0	90.5	25.11	0.86	16	1	43	25.14	33.36	12.87	5.44
741	68	1	1	0	74.5	19.98	2	29	0	14.9	7.77	44.32	19.24	7.32
742	85	1	1	1	81	20.30	1.5	42	0	13.3	8.49	55.34	25.12	7.63
743	77	1	0	0	76	19.66	1.2	31	1	18.5	9.09	40.17	16.47	6.21
744	78	1	3	0	90	26.91	1.5	37	0	30.3	22.54	51.94	23.03	8.12
745	75	2	0	0	82	27.31	1	16	1	41.7	24.58	34.39	13.81	6.43
746	68	1	0	0	92	24.11	3	42	0	28.9	20.49	50.31	21.74	7.29
747	76	1	1	1	75	21.19	1	25	2	20.9	11.16	42.33	17.97	6.63
748	74	1	1	1	91	25.13	1.2	36	1	33.4	21.59	43.10	19.22	7
749	65	2	0	0	83	24.63	2	25	0	36.5	20.60	35.81	15.60	6.75
750	74	2	0	0	91	29.88	1	22	1	44.3	29.21	36.68	15.57	6.99
751	79	1	3	1	90	23.95	3	27	1	26.9	17.15	46.56	19.33	7.22
752	76	2	0	0	89	24.26	1.2	24	0	32.2	18.75	39.48	15.98	6.52
753	65	2	0	0	88	25.09	2	33	0	38.7	25.44	40.35	18.00	6.79
754	73	2	0	0	90	22.85	1.2	21	0	34	17.09	33.14	13.21	5.97
755	72	2	0	0	79	23.71	1.5	22	0	41.4	22.64	32.09	12.96	5.75
756	77	1	1	1	86	24.70	1.5	31	0	28.5	16.55	41.44	18.89	7.8
757	77	1	3	1	97	26.40	1	27	2	30.4	21.41	48.98	22.82	8.33
758	72	1	1	0	73	19.09	2	39	1	16.1	7.57	39.41	16.93	6.55
759	72	1	3	1	88	23.82	1.5	40	0	21.4	12.47	45.85	19.70	7.82
760	72	1	0	0	95	22.72	1.5	36	1	33.2	24.87	50.05	21.50	6.79
761	67	1	3	1	80	23.85	3	35	0	20.7	12.45	47.70	19.71	7.84
762	74	1	0	1	93	25.40	1.5	34	0	23	15.55	52.18	22.06	7.88
763	70	2	0	0	79	20.51	2	25	0	29.3	15.35	37.01	15.97	6.35
764	63	2	0	0	78	22.15	1.5	22	0	30.1	15.64	36.40	14.69	6.39
765	69	2	0	0	91	31.15	1.5	25	0	47.5	33.29	36.78	15.39	6.75
766	66	1	1	0	86	25.51	3	35	0	26.8	18.83	51.32	23.63	8.37
767	80	1	0	0	80	21.81	1.2	16	1	24.4	12.14	37.64	16.67	6.97
768	67	2	0	0	84	26.60	1.2	16	1	43	27.65	36.72	16.71	6.85
769	79	1	3	0	83	22.02	1.5	20	1	26.2	15.71	44.29	19.55	7.29
770	73	2	0	0	80	22.66	1.5	15	1	32	17.69	37.59	16.02	6.63
771	71	2	0	0	83	27.81	1.5	20	0	45.9	24.80	29.23	11.62	5.93
772	70	2	0	0	83.5	23.73	1	24	1	33.1	17.97	36.37	16.23	7.12
773	67	2	1	0	90	23.16	0.86	22	1	33.6	15.90	31.47	12.88	6.25
774	68	1	3	0	100	29.67	2	22	1	32.1	20.99	44.36	18.96	8.25
775	69	1	0	0	88.5	27.60	1.5	35	0	22.1	14.29	50.27	20.96	8.71
776	77	1	0	0	87	24.96	2	32	0	24.1	14.95	47.11	20.35	7.95
777	81	2	0	0	107	32.76	0.67	15	1	48	30.38	32.96	13.35	6.79
778	75	1	0	0	89	24.32	2	28	1	32.6	21.81	45.10	21.86	7.63
779	72	1	1	0	79	21.29	1.5	40	0	15.8	8.58	45.87	18.15	6.98
780	72	1	1	0	111	33.35	1.5	36	1	35.2	30.34	55.75	23.86	8.88
781	66	1	1	1	100	25.75	2	26	1	34.1	26.31	50.85	23.25	7.63
782	81	1	0	0	74.5	22.26	0.75	16	2	23	11.54	38.75	16.32	6.97
783	77	2	0	0	74	19.24	1.2	10	1	30.1	13.28	30.88	12.38	5.36
784	70	1	1	1	86.5	23.93	1.2	32	1	25.2	14.25	42.29	17.89	7.48
785	77	1	3	0	77	22.21	1	32	1	20.2	11.45	45.14	19.78	7.51
786	72	1	1	1	88	25.83	1.5	37	0	27.8	16.55	42.89	18.92	7.9
787	71	2	0	0	71.5	22.05	1.2	19	0	29.9	14.19	33.60	13.30	6.09
788	67	2	0	0	85	27.66	1.5	20	0	44.7	25.00	30.90	11.19	5.41
789	83	2	1	0	96	27.70	1	13	1	40.4	24.78	36.60	14.00	6.27
790	67	1	0	0	86	26.63	2	39	0	22.1	15.25	53.64	24.04	9.16
791	76	2	0	1	80	22.98	1.5	25	0	30.5	16.76	38.15	15.71	6.71

792	74	1	1	0	92	39.86	1.5	30	1	26.8	16.54	45.17	18.57	7.55
793	80	2	0	0	70	17.94	2	18	0	20.5	9.00	34.83	13.97	5.97
794	76	2	0	0	89	21.37	0.4	12	2					
795	77	2	0	0	92	22.80	1	20	1	40.1	18.27	27.30	10.44	5
796	88	2	3	0	99	26.64	1	15	2	43.3	26.63	34.94	13.99	5.86
797	82	1	3	1	98	26.54	1.2	22	1	35.7	24.33	43.80	19.32	7.26
798	75	1	0	1	78	21.38	1.2	23	1	25.8	13.18	37.95	16.07	6.55
799	76	2	0	0	82	20.79	1.5	13	1	41	19.26	27.73	11.90	5.33
800	79	2	0	0	92.5	26.31	1.2	19	0	37.7	22.33	36.94	15.63	6.9
801	79	2	0	0	97.5	28.15	1.2	16	1	41.1	27.94	40.01	16.01	6.5
802	80	2	0	0	82	23.62	0.55	16	2	36.7	18.73	32.37	12.13	5.45
803	72	1	3	0	85	23.32	2	30	0	22.4	12.00	41.52	18.51	7.95
804	74	2	0	0	102	32.45	0.86	17	1	52.9	38.29	34.16	12.79	5.62
805	66	1	0	0	86	22.39	1.5	30	0	25.4	13.53	39.64	16.99	7.02
806	74	1	1	1	81	20.82	1.5	31	0	23.9	11.76	37.43	14.33	5.89
807	66	2	0	1	85	22.51	1.5	25	1	36.2	18.21	32.11	12.24	5.38
808	67	1	1	0	76	21.54	1.5	24	2	17.3	9.93	47.56	20.03	7.31
809	78	1	0	0	91	24.01	1.5	24	1	28.1	16.73	42.85	19.44	7.6
810	72	2	0	0	83	21.94	1	15	1	31.7	16.78	36.16	13.62	5.76
811	64	2	0	1	74.5	21.99	2	23	0	34.4	17.46	33.35	13.95	5.99
812	73	1	3	0	85	22.10	2	34	0	24.2	13.90	43.43	18.23	6.8
813	83	1	3	0	91.5	23.62	2	28	1	23.4	14.46	47.44	20.95	7.99
814	79	2	0	0	94	24.55	0.29	13	2	27	13.47	36.44	14.50	6.86
815	68	2	0	1	82	24.27	2	23	0	36.4	20.64	36.13	14.75	6.2
816	73	1	1	0	67.5	18.97	2	27	1	7.6	3.89	47.43	21.18	7.52
817	75	2	0	0	87	20.89	1	13	2	30.7	14.78	33.40	13.58	5.89
818	76	2	0	0	77	21.22	1	19	1	27.1	12.36	33.23	13.15	5.96
819	74	1	3	0	94	24.75	0.86	17	2	27.1	18.06	48.51	19.91	7.18
820	70	2	0	0	79.5	25.25	1.2	22	0	36.9	23.29	39.90	17.37	6.81
821	73	2	0	0	88	26.60	2	23	0	36.3	21.19	37.12	14.06	6.59
822	74	1	3	1	86	23.98	2	29	0	24.3	14.32	44.54	20.04	7.88
823	75	2	0	1	91	23.18	1.2	23	0					
824	80	2	0	0	94	28.42	1	20	1	33.6	22.50	44.45	18.42	7.92
825	72	2	0	0	78.5	38.85	1.5	16	1	35.9	21.86	39.09	17.05	6.86
826	76	2	0	0	82	21.02	1.2	22	0	28.6	13.48	33.70	13.95	6.22
827	87	1	0	0	105	26.23	1.5	37	0	29.4	20.81	50.08	23.17	8.5
828	72	1	3	0	90	24.89	2	13	1					
829	69	2	0	0	97	34.26	1.2	17	1	45.2	31.74	38.45	13.95	6.97
830	71	1	0	1	87.5	25.32	1.5	33	0	32.1	19.48	41.25	17.42	6.88
831	68	2	0	0	94	25.32	1.5	23	0	34.5	23.00	43.63	19.82	7.47
832	77	2	0	0	83	22.10	1.2	11	1	31.3	15.41	33.81	13.27	5.89
833	77	2	0	0	85	26.57	1	17	1	36.7	19.39	33.40	10.96	5.44
834	82	1	3	0	98.5	28.02	0.86	25	1	33.8	22.30	43.70	19.66	8.09
835	65	1	3	1	86	24.04	2	44	0	21.7	13.51	48.72	21.98	8.37
836	81	1	0	0	83	21.50	1	22	2	21.1	11.02	41.16	17.71	7.16
837	67	1	0	1	84	22.88	2	39.5	0	21.9	13.91	49.65	22.10	7.6
838	73	1	0	0	84	23.24	1.5	25	1	27.9	16.86	43.56	19.89	7.35
839	66	2	0	0	87	25.19	2	22	0	38.9	22.55	35.36	14.72	6.4
840	71	2	0	1	103	26.01	1	17	1	40	22.48	33.79	13.86	6.15
841	72	1	1	0	94	27.11	1.2	32	1	29.6	21.32	50.69	21.53	8
842	74	2	0	0	80	24.61	1	20	1	33.1	17.71	35.78	15.34	7.18
843	72	1	3	1	88	20.74	1.5	26	1	18.6	10.01	43.94	17.95	6.86
844	73	2	0	0	91	25.28	0.86	13	1	33.6	19.02	37.51	15.93	6.89
845	68	1	0	0	80	20.85	2	32	0	14.1	7.83	47.86	20.41	7.45
846	78	2	0	0	89	23.95	1	20	1	35.3	19.62	36.00	15.00	6.42
847	69	1	1	0	75	22.45	1.2	36	1	15.6	8.68	46.96	21.03	8.44
848	70	2	0	0	85	19.93	1.5	22	0	22.6	10.39	35.59	14.95	6.55
849	71	1	0	1	99	27.47	2	25	1	31.9	23.62	50.53	21.49	7.79
850	82	1	3	1	96	25.85	1.5	28	1	34.1	20.03	38.69	15.77	6.73
851	74	2	0	0	81	21.27	1.2	16	1	24.1	9.97	31.32	13.02	6.74
852	65	2	0	1	99	34.80	1.5	20	0	50.4	40.01	39.44	18.29	7.91
853	77	1	1	1	82	22.34	1.2	28.5	1	26.9	14.39	39.01	16.43	6.53
854	77	2	0	0	105.5	29.93	0.6	19	1	40.9	26.82	38.79	15.27	6.84
855	64	2	0	0	92	29.63	1	20	1	40.1	27.12	40.52	15.92	6.64
856	62	2	0	0	101	30.27	1.2	19	0	36.9	23.42	40.02	14.74	7.05
857	62	2	1	0	94	28.95	1.5	20	0	40.8	27.64	40.07	15.76	6.53
858	71	2	0	0	81	25.40	1.5	18	0	33	19.35	39.32	14.50	6.14
859	82	1	1	0	84.5	22.76	1.5	18	1	24.3	13.91	43.43	19.00	7.33
860	77	1	3	0	89	27.14	3	30	1	31.6	22.76	49.19	20.86	7.76
861	70	2	0	0	86	21.90	0.67	21	1	30.4	15.54	35.53	13.31	5.81
862	54	1	1	1	86	25.63	2	42	0	26.7	18.75	51.44	22.58	8.12
863	76	1	1	0	90	23.99	1	22	1	28.2	16.75	42.57	17.74	6.89

864	76	1	3	0	86	25.17	1.5	37	1	24.5	17.23	53.05	23.46	8.17
865	69	2	0	0	90.5	26.90	2	22	0	41.8	25.34	35.25	15.19	6.6
866	74	2	0	0	79	19.82	1.5	22	0	34.5	19.19	36.49	14.75	5.41
867	66	2	0	0	78.5	26.30	1.2	20	0	37.9	20.93	34.25	12.92	6.18
868	61	2	0	1	73	22.59	2	28	0	35	18.77	34.94	14.84	6.18
869	71	2	0	0	95	24.83	0.86	28	1	31	18.57	41.42	16.11	6.69
870	70	1	1	1	93.4	25.91	1.5	32	0	28.5	20.84	52.19	23.89	8.28
871	66	1	0	1	83	23.22	2	42	0	21.3	12.97	47.81	20.77	7.89
872	79	1	0	1	92	25.25	1.5	20	1	29	18.22	44.51	18.55	7.32
873	75	2	0	1	82	27.73	1.2	23	0	35.5	23.20	42.16	17.69	7.55
874	73	2	0	0	80	24.23	2	28	0	37.7	21.82	36.10	15.43	6.38
875	76	2	0	0	88	22.84	1	26	1	27.1	13.79	37.03	13.77	6.28
876	68	1	0	0	94	26.76	1.2	34	1	26.9	17.36	47.15	21.21	8.64
877	70	1	3	0	100	28.19	1.5	32	0	36.4	25.82	45.11	18.86	7.29
878	69	1	0	0	80	21.17	3	32	0	19.5	10.70	44.23	20.11	7.62
879	60	2	0	0	74	23.60	1.5	21	1	32.8	16.39	33.58	14.03	6.64
880	65	1	1	1	104.5	30.92	1.5	39	0	29.9	25.54	59.82	26.78	9.28
881	85	1	3	1	99	31.75	1.5	35	1	40.6	28.05	41.07	18.45	8.09
882	83	2	0	0	74	18.91	1.5	21	0	29.9	12.39	29.05	11.99	5.27
883	60	2	0	1	86	22.95	2	28	0	35.1	19.49	36.01	15.91	6.4
884	61	1	1	0	90.5	28.45	2	38	0	31.4	24.76	54.17	24.27	8.8
885	62	2	0	0	120	39.98	1	17	1	51.7	49.10	45.96	19.06	7.67
886	81	1	3	0	91	27.98	1.5	35	1	30.7	20.30	45.83	21.52	8.76
887	75	2	0	0	77	22.55	1	16	2	36	18.51	32.94	13.86	6
888	72	1	3	0	112	28.26	2	33	0	34.1	24.66	47.69	20.10	7.45
889	67	2	0	0	76	20.80	1.5	18	0	29.4	14.67	35.31	14.90	6.19
890	58	2	0	1	80	22.74	2	28	0	39.7	20.50	31.14	12.58	5.28
891	74	1	3	0	89	24.46	1.2	30	1	23.9	15.04	47.98	21.01	8.06
892	78	1	0	0	98	23.58	1.5	31	0	29.3	20.28	48.95	22.05	7.28
893	73	2	0	0	110	36.14	0.86	29	1	48	43.15	46.77	20.32	7.89
894	61	2	0	0	75.5	20.80	2	27	0	25.5	12.54	36.61	15.36	6.35
895	78	2	0	0	83	24.03	1.5	19	1	41.5	21.33	30.04	11.13	5.09
896	72	2	0	0	81	23.29	1.5	22	1	40.7	21.80	31.81	13.53	5.57
897	67	2	0	0	73	23.45	2	28	0	38.3	19.12	30.84	12.88	5.79
898	66	2	0	0	87	28.43	1.5	20	0	44.8	28.13	34.63	14.77	6.47
899	69	1	3	0	89	25.47	1	28	1	23.7	15.13	48.64	20.49	7.84
900	74	1	0	1	88	22.88	0.75	22	1	27.8	15.77	41.05	15.97	6.04
901	62	1	1	1	106.5	32.61	3	31	0	33	26.03	52.86	23.04	9.26
902	76	1	3	0	101	30.03	1.5	36	0	39.2	28.43	44.15	20.00	7.85
903	67	1	1	1	83	20.49	2	34	0	10.6	5.44	45.80	19.55	7.37
904	64	2	0	0	74	24.72	1.2	23	0	47.9	27.41	29.80	12.12	4.96
905	56	1	1	1	93	26.98	2	51	0	34.5	27.24	51.67	22.67	7.38
906	71	2	0	0	95	26.12	1.2	16	1	39.5	22.11	33.87	14.27	6.55
907	73	2	0	0	96	20.56	1.2	15	1	33	17.63	35.74	14.40	5.41
908	78	1	1	0	78	19.97	1	24	2	17	8.81	42.90	17.55	6.48
909	77	2	0	0	79	24.56	1.2	14	1	40.6	20.86	30.57	12.41	5.86
910	69	2	0	0	90	27.28	1.5	22	0	45.7	26.37	31.30	13.38	6.08
911	68	2	0	0	87	27.63	1.5	28	0	42.8	31.30	41.80	18.81	7.08
912	70	1	0	0	85	26.10	2	40	0	32.6	23.36	48.24	20.72	7.37
913	63	2	0	0	92	28.83	2	22	0	45	26.96	32.91	13.37	6.25
914	62	2	0	0	86	24.95	1.5	24	0	35.5	19.28	35.39	14.27	6.03
915	71	2	0	0	89	29.95	1.2	21	0	45.6	30.72	36.60	14.91	6.45
916	67	1	1	1	79	19.94	2	32	0	18.8	9.68	41.79	17.06	6.31
917	59	2	0	0	74	24.60	2	28	0	36.2	17.88	31.51	12.84	6.1
918	53	2	0	0	84	26.90	1.5	30	1	38.1	23.97	38.89	17.45	7.3
919	61	1	1	0	85	22.46	1.5	38	0	21.6	11.97	43.44	18.13	7.12
920	71	2	0	1	74	23.92	1.2	22	0	36.8	18.99	32.62	13.99	5.82
921	51	2	0	0	76	20.49	2	29	0	32.4	16.70	34.91	14.58	5.58
922	68	2	0	1	74	20.87	2	23	0	28.7	13.24	32.92	13.71	6.04
923	77	1	3	1	84	24.83	1	30	1	23	13.27	44.44	20.71	8.6
924	72	1	0	0	91	20.96	1.5	32	0	19.2	10.84	45.62	19.83	7.07
925	50	2	0	1	82	24.56	2	33	0	31	19.41	43.19	19.72	7.4
926	84	2	0	0	92	25.58	1	20	1	40	26.10	39.16	17.73	6.89
927	59	1	0	0	86	25.69	1.5	40	0	28.1	17.59	44.97	19.87	7.45
928	60	1	1	0	100	29.91	2	43	1	28.4	24.82	62.54	26.25	8.8
929	61	1	0	0	91	28.47	3	30	0	22.1	15.79	55.77	24.28	9.36
930	58	2	0	1	76.5	22.02	1.2	22	0	33.9	18.34	35.68	14.93	5.82
931	59	1	0	0	89	25.90	2	40	0	25.4	17.41	51.19	23.93	8.55
932	72	1	3	1	86	26.07	3	28	1	24.6	15.01	45.90	20.10	8.24
933	65	2	0	0	76	22.82	1.2	19	0	32.7	17.58	36.21	15.22	6.33
934	74	1	0	0	91	26.06	1.2	33	1	27.2	18.34	48.97	21.25	7.9
935	78	2	0	0	98	28.21	0.75	16	2	53.2	35.45	31.21	13.38	5.8

936	52	1	0	1	94	27.42	2	41	0	30.9	23.07	51.53	22.30	7.51
937	52	2	0	1	78.5	22.14	2	22	0	37.2	19.09	32.29	13.98	5.94
938	78	2	0	0	84	24.39	1.5	16	1	33.4	16.60	33.15	12.81	6.21
939	78	2	0	0	111	32.39	1.2	15	1	40.4	27.42	40.52	16.43	7.62
940	71	1	1	0	78	24.33	1.5	46	0	25.1	17.58	52.47	23.77	7.93
941	69	2	0	0	93.5	24.86	3	22	0	36.9	20.17	34.44	14.50	6.44
942	61	2	0	0	89	27.01	1.5	25	0	35.5	19.65	35.77	16.10	7.71
943	55	2	0	1	74.5	24.67	2	25	0	37.9	21.44	35.12	15.08	6.53
944	61	2	0	0	77	29.34	1.2	25	0	41.1	26.43	37.94	16.72	7.42
945	51	2	0	0	70.5	18.15	1.5	15	1	30.3	13.08	30.10	13.20	5.51
946	79	2	0	0	79	24.77	1	18	1	31.3	15.44	33.88	13.32	6.5
947	66	2	0	0	96	21.79	1.5	19	0	39.5	22.37	34.21	14.21	5.51
948	55	1	0	0	98	34.23	1.5	39	0	37.8	31.51	51.96	22.08	8.79
949	72	1	0	0	90.5	25.11	0.6	25	1	25.6	15.72	45.62	19.71	7.81
950	59	2	0	0	91	28.42	1.5	24	0	46	31.08	36.49	15.51	6.33
951	62	2	0	0	80	22.46	1.5	25	0	29.2	14.68	35.62	14.87	6.44
952	56	2	0	0	80	22.66	3	22	0	31.9	19.66	41.94	18.05	6.54
953	57	2	0	0	94.5	28.30	1.5	29	1	49.3	34.22	35.20	17.05	6.87
954	59	2	0	1	68	19.75	2	30	0	24.2	11.37	35.63	14.21	5.81
955	52	2	0	0	80	26.02	1.5	28	0	34.9	23.64	44.05	20.18	7.63
956	64	2	0	0	90	29.17	1	21	1	43.8	31.39	40.22	15.40	6.15
957	63	2	0	0	74	22.16	2	18	0	44.8	20.94	25.83	10.26	4.88
958	60	2	0	0	76	20.47	2	26	0	37.2	20.67	34.86	15.61	5.6
959	56	2	0	0	89	27.10	2	21	0	37.4	24.02	40.27	18.25	7.59
960	51	1	3	1	85	25.98	3	52	0	19.7	13.94	56.85	26.85	9.5
961	62	1	3	0	76	17.88	1.2	27	2	14.3	7.69	46.25	18.87	6.25
962	50	2	0	0	85	26.93	1.5	22	0	37.4	24.08	40.24	16.25	6.87
963	60	2	0	1	80	25.02	2	29	0	36.5	22.88	39.87	16.84	6.65
964	52	2	0	0	74	20.96	2	27	0	37.7	17.60	29.12	12.61	5.55
965	59	2	0	0	75	19.79	1.5	16	1	30.3	13.06	30.04	12.82	5.49
966	57	2	0	0	97	28.19	2	25	0	46.7	30.87	35.26	14.73	6.05
967	62	2	0	0	76	22.17	3	14	1	38.2	20.17	32.62	13.60	5.58
968	53	2	0	0	91	29.21	1.5	30	0	33.9	24.15	47.18	20.09	8.15
969	53	1	3	1	81	26.46	2	48	0	22.7	15.77	53.75	25.31	9.53
970	51	2	0	0	74	23.30	2	30	0	27.6	14.53	38.08	16.83	7.44
971	53	1	0	0	88	25.95	2	39	0	26.5	18.11	50.18	22.98	8.62
972	54	2	0	0	83	26.25	2	19	0	37.9	23.32	38.24	16.44	7.13
973	50	2	0	0	70	20.20	2	21	0	25.5	11.56	33.84	15.31	6.73
974	53	1	3	0	86	28.68	2	52	0	23.2	17.70	58.66	26.61	9.93
975	61	1	0	0	75	20.50	2	41	0	10	5.09	45.75	19.86	8.05
976	55	1	0	0	98	29.14	2	34	0	30.7	26.16	59.17	26.30	9.02
977	50	1	1	1	82	22.58	1.5	44	0	14.8	9.16	52.64	22.93	8.31
978	52	2	0	0	103	34.49	1.5	29	0	37	32.58	55.52	20.56	7.85
979	56	1	1	1	83	22.05	2	42	0	22.6	14.75	50.39	22.66	7.69
980	58	2	0	0	77	22.37	1.5	26	0	36.7	19.16	33.07	13.90	5.58
981	61	1	0	0	86.5	23.86	1.5	37	0	23.9	15.63	49.71	22.66	7.91
982	56	2	0	1	84	28.66	1	20	1	35.8	21.69	38.83	17.88	8.32
983	62	2	0	1	78	21.70	2	21	1	35.4	17.22	31.43	13.67	5.7
984	61	2	0	1	73	21.68	2	20	0	37.2	17.06	28.75	10.61	4.86
985	59	2	0	1	87	24.43	1.2	27	0	32.8	17.30	35.50	15.01	6.82
986	55	2	1	0	70	22.66	2	26	0	26.8	13.24	36.12	15.81	6.17
987	56	2	0	0	76	21.90	1.5	21	0	34.3	17.36	33.23	14.60	6.22
988	53	1	1	1	89	24.44	2	38	0	28.9	18.08	44.40	19.62	7.36
989	59	2	0	1	85	26.61	3	28	0	30.8	18.94	42.61	17.42	7.5
990	58	1	1	0	76.5	21.91	2	37	0	6.7	3.75	51.78	22.62	8.65
991	67	2	0	0	70	21.52	2	24	0	27.5	12.58	33.16	13.83	6.61
992	50	2	0	1	64	20.00	2	20	0	35.1	18.02	33.33	13.35	5.23
993	59	1	3	1	84.5	26.83	2	35	0	27.5	18.99	49.95	23.46	8.75
994	51	1	0	1	74	19.47	3	42	0	13.1	6.84	45.36	19.79	7.2672
995	59	2	0	0	73.4	19.71	2	22	0	33.6	16.69	33.06	13.77	5.3525
996	60	2	0	1	88	26.11	2	25	0	41.1	24.25	34.74	15.26	6.6376
997	57	2	0	1	70	21.19	2	30	0	28	12.81	32.88	14.27	6.3924
998	67	2	0	0	97	26.82	0.86	26	1	38.6	28.26	44.93	19.87	7.9914
999	63	1	0	0	79	21.48	3	39	0	19.6	11.73	48.25	22.81	7.692
1000	56	1	0	1	91	27.22	3	43	0	32.3	25.56	53.64	25.30	8.4932
1001	57	2	0	0	84.5	23.49	2	30	0	41.8	24.57	34.22	15.34	6.0434
1002	52	1	1	1	117.5	38.26	1.5	14	1	38.3	38.74	62.39	25.44	9.3652
1003	51	1	1	0	88	26.04	2	34	1	31.6	24.18	52.40	23.75	7.9083
1004	58	1	3	1	87	25.44	1.5	36	0	22.8	13.90	47.01	19.78	8.1705
1005	57	2	0	0	75	20.78	2	21	0	38.1	19.65	31.98	12.72	4.9691
1006	57	2	0	1	72	25.33	3	23	0	40.1	22.53	33.62	14.70	6.5338
1007	76	1	1	0	87	23.33	1.5	30	0	21	11.26	42.38	16.90	7.0504

1008	61	2	0	1	90.5	25.11	3	20	0	36	18.75	33.29	13.94	6.6302
1009	54	1	1	1	92	26.02	3	56	0	28.5	21.16	53.16	24.30	8.3019
1010	54	2	0	1	91	30.12	1.5	21	0	49.8	35.70	36.00	14.97	6.0199
1011	62	2	0	0	86	23.42	2	27	0	36.7	21.70	37.40	15.86	6.4257
1012	54	1	0	0	94	28.98	2	48	0	26.8	20.35	55.48	26.56	9.7447
1013	56	1	1	1	94	26.42	3	50	0	28.8	22.55	55.65	24.93	8.0127
1014	62	1	0	1	88	25.71	3	57	0	27.9	19.97	51.62	23.24	7.8838
1015	64	1	0	1	80	20.46	1.5	27	1	24.9	12.60	37.95	16.64	6.3394
1016	63	1	0	1	96	25.94	2	43	0	27	17.11	46.27	20.27	7.8891
1017	54	2	0	1	85	24.67	1.5	22	0	35.5	17.46	31.71	12.41	6.1094
1018	73	2	0	0	76.5	23.42	1.5	13	1	35.1	20.60	38.14	15.30	5.9335
1019	78	1	0	0	94	24.96	2	22	1					
1020	77	2	0	0	102	29.42	1	16	1	41.3	26.39	37.55	15.06	6.7748
1021	64	1	0	0	82	28.18	2	40	0	23	15.12	50.74	22.95	9.6519
1022	75	1	1	1	84	24.61	1.2	22	2	21.6	11.20	40.57	14.74	6.63
1023	74	2	0	0	99	27.20	1	18	1					
1024	61	2	0	0	82	28.74	1.5	24	0	45.4	30.22	36.33	15.64	6.3189
1025	61	2	0	0	73	22.79	3	30	0	32.7	16.47	33.92	14.42	6.5474
1026	61	2	0	0	77	23.78	2	26	0	35.9	19.22	34.29	14.61	6.1608
1027	81	2	0	0	95	22.24	0.67	8	1	18.2	7.29	32.70	13.12	7.1364
1028	62	1	0	1	63	17.54	2	34	0	9.7	4.50	41.75	18.77	6.8438
1029	61	2	0	0	72	22.36	2	22	0	26.6	14.02	38.77	15.17	6.256
1030	75	2	0	0	90	28.55	0.98	26	1	39.7	23.47	35.63	15.19	6.9147
1031	76	2	0	0	93	30.42	0.86	12	2	35	22.34	41.42	14.11	6.5036
1032	56	2	0	0	92	33.49	1.33	20	0	48	31.25	33.79	14.24	6.991
1033	82	2	0	0	80	23.83	1.2	15	1	29.3	14.18	34.20	13.47	6.2086
1034	77	2	0	0	96	28.11	2	20	0	41.7	30.42	42.52	18.69	7.1381
1035	52	2	0	1	106	41.11	1.5	22	0	56.7	48.15	36.76	13.66	6.255
1036	54	2	0	0	83	27.37	2	30	0	44	30.59	38.92	16.48	6.3267
1037	64	1	0	0	91.5	26.45	1.69	34	1	28.1	18.78	48.16	20.56	7.8164
1038	72	1	1	1	89	25.60	2	30	1	25.3	17.14	50.62	22.72	8.4765
1039	55	2	0	0	71.5	23.37	1.5	24	0	34.8	17.46	32.73	13.22	5.9854
1040	53	1	0	1	101	35.16	2	45	0	41	37.98	54.57	21.59	8.0764
1041	59	2	0	0	96	26.14	1.5	22	0	36.8	20.22	34.66	13.64	6.4258
1042	64	2	0	0	83	23.81	1.2	19	1	40.7	22.98	33.53	14.17	5.8065
1043	63	2	0	0	76	24.58	2	23	0	36.2	20.00	35.21	14.56	6.2361
1044	70	1	0	1	84	24.36	1.5	28	1	20.6	12.97	49.97	21.61	8.3154
1045	77	1	0	0	85	20.89	1.5	36	0	26.5	14.70	40.83	17.99	6.6404
1046	71	2	0	0	84	22.30	0.92	22	1	40	21.86	32.83	13.24	5.3092
1047	64	2	0	0	89	24.20	2	22	0	40.6	23.62	34.53	14.83	6.0314
1048	67	1	0	0	92	26.11	1.5	30	0	31.7	21.18	45.67	19.93	7.5832
1049	62	1	1	1	84	25.97	2	28.5	0	23.1	14.78	49.08	21.01	8.3937
1050	68	1	3	0	81	20.31	2	28	1	14.1	7.14	43.42	18.42	7.1969
1051	66	2	0	0	90	27.16	1.69	20	0	46.5	30.11	34.59	13.97	5.8386
1052	53	1	0	0	91	27.93	2	35	0	30.9	22.09	49.44	22.20	8.4572
1053	55	2	0	0	90.5	27.73	1.2	22	0	45.8	31.01	36.73	16.73	6.7524
1054	59	2	0	1	71.5	23.97	1.5	25	0	41.9	25.55	35.42	15.88	6.2491
1055	50	2	0	0	82.5	25.28	2.07	23	0	38.4	22.96	36.88	15.55	6.2274
1056	71	1	0	1	72	19.77	1.08	30	1	24	10.67	33.72	13.98	6.0753
1057	53	2	0	0	84	27.58	1.61	18	0	32.2	20.19	42.55	17.52	7.3104
1058	70	2	0	0	98	25.18	1.2	20	0	36.1	20.92	37.11	15.83	6.5111
1059	56	1	0	0	86	24.57	1.79	49	0	21.9	13.54	48.26	22.07	8.3176
1060	70	1	0	1	99	28.43	2	38	0	29.4	23.48	56.42	26.16	9.1269
1061	68	2	0	0	82	26.74	1.2	14	1	46.2	27.22	31.68	12.91	5.7935
1062	68	2	0	0	102	29.27	1.2	26	1	37.4	27.74	46.48	20.99	7.9874
1063	71	2	0	0	88	26.99	1.2	17	1	40.9	21.66	31.36	12.26	6.039
1064	76	2	0	1	77	23.58	1.2	14	1	30.7	16.92	38.19	16.86	7.0371
1065	71	2	0	0	80	25.60	2	19	0	38	22.22	36.26	15.25	6.6193
1066	54	1	1	1	76	24.85	2	41	0	22.2	13.77	48.20	21.93	8.5541
1067	53	2	0	0	85	27.13	1.2	25	0	41	27.10	38.92	16.26	6.3754
1068	53	2	0	0	87	25.91	2	17	1	44.3	28.94	36.45	15.36	5.9416
1069	52	2	0	0	80	25.55	3	27	0	36.2	23.44	41.33	16.80	6.6033
1070	54	2	0	0	90	24.64	1.73	27	0	41.8	26.35	36.67	16.56	6.2478
1071	50	1	0	0	92	27.15	2	40	1	23.6	18.01	58.19	25.59	8.7805
1072	56	1	1	0	94	26.67	1.85	32	0	28.7	18.33	45.60	19.08	7.892
1073	62	2	0	0	66.5	20.74	2	26	0	35.9	15.37	27.45	11.20	5.2552
1074	58	2	0	0	69.5	26.80	1.5	22	0	43.9	15.62	32.77	13.73	6.1858
1075	62	2	0	1	101	31.46	1.2	30	0	51.7	34.77	32.54	12.30	5.6145
1076	73	2	0	0	87	25.04	1.2	17	1	43.8	22.38	28.68	12.09	5.8224
1077	70	2	0	0	85	23.75	1.5	20	0	35.6	20.80	37.57	16.82	6.9818
1078	61	2	0	0	82	23.96	1.5	22	0	33	16.23	3.29	12.79	6.007
1079	63	2	0	0	67	21.20	1.5	25	0	31.7	14.86	32.01	13.09	5.8785

1080	74	2	0	0	74	28.61	1.2	20	1	46.1	27.36	32.04	13.55	6.4705
1081	57	1	1	0	80	24.61	2	40	0	28.3	18.68	47.28	18.82	6.7331
1082	57	2	0	1	80	21.39	2	28	0	32.5	18.77	38.98	17.18	6.358
1083	63	2	0	0	83	25.33	1.5	17	1	35.7	19.68	35.50	14.63	6.7713
1084	53	2	0	0	77	23.51	2	26	0	36.5	21.08	36.72	15.63	6.1438
1085	68	1	3	0	79.5	27.48	2	35	0	28.9	18.89	46.40	20.36	8.3861
1086	58	1	1	1	93	24.73	1.5	44	0	24.5	17.82	54.86	25.43	8.557
1087	65	1	0	0	85	26.13	2	46	0	20.2	13.73	54.24	25.63	9.3811
1088	66	1	0	0	91	26.79	1.5	44	0	29.2	20.30	49.22	21.59	8.227
1089	69	2	0	0	85	23.35	1	20	1	35.4	17.66	32.27	13.38	6.066
1090	57	1	0	0	80	24.17	3	39	0	27.4	16.07	42.61	18.81	7.4764
1091	76	1	1	0	83	22.93	1.2	42	1	16.7	10.21	51.00	22.60	8.5379
1092	73	2	0	0	89	26.98	1.5	13	1	40.7	27.04	39.33	15.36	6.2887
1093	51	2	0	1	76.5	24.76	3	36	0	40.5	23.63	34.74	14.84	6.0444
1094	63	2	0	0	88	27.44	1.5	17	1	39.1	22.37	34.85	12.40	6.0564
1095	52	1	0	0	84	24.63	3	41	0	24.7	16.16	49.28	22.55	8.3637
1096	51	1	0	0	83	26.70	1.5	42	0	29.2	20.02	48.65	22.43	8.4016
1097	63	2	0	0	76	28.83	2	24	0	39.7	26.69	40.62	16.42	7.172
1098	67	1	0	0	73.5	23.42	3	30	0	18.2	10.29	46.14	20.81	8.2331
1099	65	1	3	1	90	24.92	1.5	41	0	27.6	18.99	49.74	20.92	7.3417
1100	78	2	0	0	87	29.92	1.2	21	0	47.5	31.57	34.94	14.12	6.2681
1101	81	1	0	0	73	18.36	1.5	27	1	14.7	7.00	40.46	17.22	6.6413
1102	64	1	0	0	95	28.73	2	40	0	37.9	27.93	45.72	20.11	7.65
1103	63	2	0	0	80.5	24.56	2	29	1	36.7	22.39	38.55	16.84	6.6707
1104	59	2	0	0	70	19.68	2	26	0	28.5	13.21	33.19	13.83	5.7789
1105	77	1	0	0	91	25.36	1.2	22	2	22.5	15.04	51.91	23.62	8.8451
1106	74	2	0	0	96	31.94	1.2	24	0	38.4	26.10	41.93	16.92	7.7987
1107	52	2	0	0	63	17.96	2	18	0	32.3	14.60	30.65	12.69	5.3998
1108	53	1	1	1	80.5	24.08	2	38	0	21.6	13.06	47.47	20.09	7.9969
1109	51	2	0	0	71	21.23	1.5	21	0	38.1	17.41	28.32	12.08	5.4348
1110	54	1	3	1	110	34.59	3	52	1					
1111	51	1	0	1	78	22.89	2	43	0	13.8	8.29	51.92	23.06	8.6694
1112	78	1	0	0	90	23.79	1.5	26	1	26.2	16.00	44.99	20.99	7.9775
1113	63	1	0	1	98	29.86	2	30	0	34.8	27.01	50.66	20.80	7.7248
1114	65	1	0	1	83	23.83	1.5	35	0	20.5	12.35	47.85	21.51	8.3593
1115	63	2	0	0	71	23.75	1.5	23	0	28.6	13.63	33.98	14.08	6.8946
1116	59	1	1	1	91	27.46	2	30	0	27	18.68	50.54	22.23	8.7916
1117	55	2	0	1	74	22.03	2	25	0	33.7	18.80	36.93	16.05	6.3244
1118	55	1	3	1	85.5	29.18	2	37	1	22.6	15.03	51.51	22.08	9.5455
1119	55	2	0	0	81	28.58	2	24	0	40.8	25.36	36.84	16.41	7.4214
1120	59	2	0	0	93	32.17	1.5	24	0	40.2	30.59	45.58	19.24	7.9352
1121	50	2	0	0	83	25.85	1.5	31	0	35.1	20.82	38.58	17.02	7.2213
1122	55	2	0	1	80	26.09	1.5	30	0	35.5	22.63	41.15	18.89	7.6522
1123	60	1	0	1	86	24.57	3	46	0	25.1	17.99	53.74	24.39	8.1288
1124	58	2	0	0	79	25.34	2	25	0	28.7	16.56	41.07	16.70	7.2948
1125	61	2	0	0	98.5	32.17	1.5	28	0	45	33.78	41.22	17.45	7.245
1126	68	1	0	1	75.5	20.79	1.5	23	1	12.4	6.25	44.09	18.90	7.5997
1127	57	1	0	0	94	29.71	1.5	38	1	38.9	27.79	43.64	18.95	7.4844
1128	54	2	0	0	78	23.82	2	24	0	40.8	22.18	32.24	13.68	5.7699
1129	71	1	1	0	84.5	23.36	2	33	0	26.4	14.81	41.37	17.98	7.0938
1130	57	1	0	1	95	25.88	2	48	0	30.1	21.30	49.54	21.61	7.7017
1131	56	1	1	1	93	26.53	2	52	0	19.7	16.40	66.70	29.57	9.2173
1132	51	1	1	0	85	24.39	3	50	0	16.9	12.27	60.35	27.47	8.9099
1133	50	2	0	0	75	23.81	2	24	0	35.9	21.54	38.41	15.91	6.1604
1134	55	1	3	1	80	24.43	2	39	0	22.2	14.27	49.93	23.35	8.6692
1135	53	1	1	1	80	19.32	2	32	0	16.6	9.25	46.43	19.82	6.8267
1136	59	2	0	0	88	28.15	1.5	26	0	43.4	29.84	38.94	16.43	6.6826
1137	76	2	0	1	86	21.20	2	22	0	29.5	13.94	33.28	14.37	6.3195
1138	68	1	1	1	78	22.55	1.5	41	0	25.5	13.54	39.64	17.32	7.0897
1139	56	1	0	1	92.5	27.26	1.2	25	1	27.7	19.62	51.27	22.95	8.6047
1140	52	1	1	0	81	22.49	2	26	1	16.7	10.29	51.33	21.25	7.797
1141	57	1	0	1	99	31.31	2	40	0	22.4	19.46	67.31	31.01	11.174
1142	62	1	0	0	91	26.47	2	48	1	27.5	21.35	56.33	24.55	8.1821
1143	58	2	0	1	79	22.89	1.5	16	1	40.2	20.12	29.95	12.04	5.3928
1144	60	2	0	0	96.5	31.14	2	27	0	45.4	33.94	40.77	16.51	6.8526
1145	60	1	1	0	83	23.92	1.5	30	0	22.2	13.70	48.08	20.07	7.7408
1146	78	2	0	0	85	22.26	1.5	19	1	38	19.90	32.49	13.12	5.4905
1147	76	2	0	0	94	25.29	1.2	15	1	38.7	21.84	34.64	12.70	5.7038
1148	64	2	0	0	96.5	34.96	1.2	25	1	48.3	40.19	43.02	18.02	7.6085
1149	62	1	3	1	83	24.13	2	48	0	24.6	15.87	48.64	22.07	8.1062
1150	62	1	1	1	87	25.51	2	38	1	24.7	17.22	52.40	23.18	8.3497
1151	56	1	0	0	93	28.68	2	46	0	28.4	22.62	56.96	26.00	9.3436

1152	53	2	0	0	68	22.74	1.5	24	0	40.8	21.73	31.59	13.58	5.7722
1153	59	2	0	1	79	24.20	1.5	30	0	34.4	20.17	38.44	15.07	6.226
1154	70	1	0	0	72	19.91	1.5	31	0	17.7	8.74	40.63	17.22	6.733
1155	63	2	0	0	82.5	26.14	1.2	19	0	33.2	18.54	37.36	16.28	7.3622
1156	54	2	0	0	93.5	22.15	2	22	0	32.7	18.39	37.86	15.35	6.0698
1157	59	1	0	1	90	26.75	2	46	0	31.2	21.87	48.13	22.34	8.4278
1158	52	2	0	0	78	27.41	2	21	0	41.7	27.20	37.96	16.03	6.7575
1159	55	1	1	1	83	27.10	2	43	0	24.3	17.16	53.57	23.97	9.0218
1160	75	1	0	0	93.5	25.68	1.5	34	0	29.2	21.67	52.46	23.69	8.1003
1161	74	2	0	0	96.5	28.95	1.2	25	0	37.4	26.82	44.86	16.99	6.6367
1162	61	2	0	1	80.5	25.65	1.5	25	0	38.8	24.23	38.17	16.80	6.7627
1163	63	2	0	0	80	24.11	1	24	1	35.6	20.59	37.32	15.44	6.2871
1164	50	2	0	1	65	19.72	2	27	0	21.8	11.14	39.93	17.93	6.8899
1165	52	2	0	0	69	21.63	2	19	0	32.8	16.09	32.94	13.87	6.0354
1166	53	2	0	1	76	22.88	1.5	24	0	38.5	20.30	32.46	13.32	5.5506
1167	58	1	1	1	91	25.41	2	40	0	20	13.54	54.21	23.70	8.7256
1168	59	1	3	1	89	23.47	2	34	1	29.5	20.88	49.97	22.02	7.2912
1169	60	1	1	0	76	21.37	2	40	0	14.4	8.33	49.71	22.04	7.9393
1170	56	2	0	0	57.5	17.13	1.5	24	1	17	7.09	34.55	13.98	5.7307
1171	65	1	0	1	87	22.33	2	27	1	17.3	10.97	52.59	23.55	8.2146
1172	51	1	0	0	84	24.28	2	36	1	27.2	18.83	50.37	21.89	7.7547
1173	59	1	1	1	90	26.51	2	39	0	27.5	19.82	52.26	24.05	8.5812
1174	61	2	0	0	84	30.17	1.5	32	0	46.9	32.73	37.00	17.17	7.1749
1175	85	1	0	0	86	24.73	1.2	25	1	26.9	16.62	45.10	19.57	7.6434
1176	57	1	3	0	89	26.97	1.5	39	0	32.9	25.87	52.70	23.74	8.0537
1177	75	2	0	0	101	29.10	0.22	11	2	44.8	28.23	34.81	15.33	6.894
1178	81	1	3	1	87	22.72	1.5	24	1	22.6	13.34	45.63	20.02	7.5815
1179	51	2	0	0	66	20.40	2	23	0	27.8	11.97	31.12	12.22	5.7961
1180	50	1	0	0	86.5	27.94	2	50	0	29	21.54	52.85	24.86	9.1858
1181	57	2	0	0	89	30.18	1.5	29	0	39	28.69	44.92	19.69	8.0286
1182	58	1	1	0	97	28.92	2	40	1	31.4	21.80	47.57	20.63	8.3897
1183	57	1	0	1	81	22.63	3	45	0	20	13.49	53.92	24.48	8.1591
1184	57	2	0	0	71	22.29	2	39	0	28.7	16.07	39.99	18.17	7.1061
1185	51	2	0	0	82.5	22.74	3	30	1	35.4	20.06	36.53	15.23	5.9704
1186	63	1	1	0	103	27.35	2	42	0	24.9	22.06	66.68	29.62	8.9801
1187	55	2	0	0	75	22.16	1.5	24	0	40.4	19.36	28.62	12.10	5.4292
1188	57	1	0	1	82	26.06	2	39	0	20.9	14.39	54.52	24.59	9.0001
1189	88	2	0	0	88	24.74	0.86	15	2	39.6	21.52	32.78	11.41	5.162
1190	51	2	0	0	77	21.88	2	28	0	38.4	21.22	34.05	15.14	5.9571
1191	54	1	0	0	82	22.11	3	42	0	11.7	6.81	51.56	23.75	8.8073
1192	50	2	1	0	84	29.03	1.5	27	0	34.5	22.72	43.04	16.55	7.0965
1193	61	2	0	1	69.5	24.01	2	23	0	38	21.83	35.68	14.99	6.2144
1194	52	2	0	0	86	24.75	1.5	32	0	38.2	24.95	40.34	17.21	6.4945
1195	59	2	0	1	80	24.16	1.2	30	0	36.2	21.12	37.19	15.98	6.5308
1196	55	2	1	0	78	24.17	1.5	28	0	36.1	20.71	36.73	15.21	6.1233
1197	65	2	0	0	81	26.52	1.5	15	1	43.8	29.49	37.89	17.68	6.9145
1198	50	1	3	1	90	27.41	3	45	0	30	21.43	50.09	22.75	8.5327
1199	54	2	0	0	63	16.98	2	16	1	13.8	5.54	34.50	14.54	6.0681
1200	61	1	0	0	94.5	29.04	2	42	0	28.6	22.94	57.16	25.20	9.0347
1201	65	2	0	0	86	25.43	1.5	27	0	37.7	23.59	38.93	16.71	6.9548
1202	51	2	0	0	72.5	21.62	1.5	27	0	29.1	15.15	36.87	15.65	6.2997
1203	51	1	0	1	79	25.89	2	47	0	26.6	19.07	52.73	23.89	8.4634
1204	54	2	0	0	88	27.30	2	21	0	37.4	22.83	38.58	15.22	6.8551
1205	52	2	0	0	87	28.88	2	21	0	42.8	28.59	38.13	15.90	6.7056
1206	77	2	0	0	94	30.67	1	16	2	38.9	31.37	49.27	21.33	8.116
1207	69	2	0	0	79	23.07	1.5	20	0	35.4	18.86	34.48	14.22	6.0989
1208	71	1	0	0	86	23.94	1.2	24	1	21.3	13.37	49.36	21.83	8.0785
1209	64	1	0	0	79	20.51	1.5	31	0	19.3	10.25	42.85	18.70	7.039
1210	59	2	0	0	87	25.23	1.2	23	0	37.4	17.91	29.95	11.63	5.9486
1211	62	2	0	0	86	25.66	1.2	20	0	36.6	21.10	36.59	15.38	6.8621
1212	51	1	1	1	71	22.03	1.2	34	1	11.5	6.79	52.33	24.51	8.8501
1213	61	1	3	0	85	26.45	2	35	1	30.7	20.26	45.66	20.38	8.0222
1214	53	2	0	0	74	23.46	1.5	33	0	32	19.41	41.22	19.62	7.2139
1215	54	1	1	0	82	23.73	2	40	0	20.3	12.18	47.75	20.87	8.1716
1216	50	1	3	1	85	23.89	2	41	0	20.6	13.17	50.62	22.73	8.3284
1217	66	1	1	1	91.5	25.35	2	31	0	37.6	27.59	45.75	18.29	6.1817
1218	57	2	0	0	98.5	27.69	2	26	0	48.7	37.36	39.38	15.55	6.1505
1219	53	1	0	0	89	24.64	2	45	0	27.5	18.51	48.83	21.93	7.7512
1220	55	1	0	0	82	22.15	2	24	1	20	12.99	51.87	22.92	7.6565
1221	50	2	0	0	60.5	20.25	3	29	0	28.6	12.24	30.55	12.60	5.797
1222	56	2	0	0	63	20.20	2	23	0	33.6	16.82	33.27	14.05	5.5836
1223	64	2	0	0	71	20.12	2	27	0	32.4	16.96	35.45	14.87	5.6448

1224	71	1	0	0	97	27.35	1	19	2	28	19.45	50.00	22.89	8.9171
1225	62	2	0	0	78	26.48	1.5	28	0	31.7	18.50	39.95	16.64	7.4045
1226	67	1	1	1	85	27.17	2	34	1	25	16.55	49.75	21.89	8.9822
1227	66	2	0	1	60	20.16	1.5	26	0	29.4	13.45	32.35	12.89	5.1827
1228	70	1	0	0	86	23.97	2	32	0	32.7	21.12	43.47	18.05	6.607
1229	72	1	3	0	90	25.60	1.5	38	0	25.8	17.94	51.49	19.49	6.9288
1230	71	2	0	0	94	33.84	1.2	25	1	49.5	39.37	40.21	19.65	8.168
1231	61	1	1	1	84	25.40	3	36	0	24.8	17.96	54.55	26.09	9.4669
1232	65	2	0	0	83	22.03	1.2	13	1	37.5	17.60	29.38	12.82	5.8841
1233	53	1	3	1	74	22.51	2	38	0	15.4	9.66	53.03	25.20	8.7006
1234	52	2	0	0	72	21.51	1.5	21	0	25.6	13.84	40.25	18.35	7.2955
1235	82	1	3	1	89	25.51	1.5	24	1	23.2	15.10	50.04	21.24	8.2331
1236	50	2	0	1	80	23.61	2	25	1	39	23.97	37.41	16.23	6.1984
1237	65	2	0	1	76	18.90	2	23	0	25.2	11.18	33.16	13.33	5.6362
1238	57	2	0	0	78	20.40	2	20	0	27.8	13.32	34.58	13.76	5.8264
1239	53	2	0	0	73.5	24.19	1.5	34	0	39.5	22.59	34.61	15.21	6.454
1240	55	1	1	1	85	24.83	1.5	38	1	26.8	18.78	51.34	22.34	7.8687
1241	79	2	0	0	69	20.12	1.5	19	0	30.2	13.63	31.42	12.63	5.5905
1242	53	2	0	0	87	30.21	1.5	27	0	41.6	26.87	37.68	16.12	7.3802
1243	59	2	0	0	74	20.44	1.5	20	0	39.6	19.78	30.17	12.63	5.1617
1244	50	2	0	0	76.5	24.21	1.5	26	0	40.3	24.12	35.80	15.74	6.2268
1245	54	1	1	1	94	27.89	3	39	0	31.6	24.57	53.25	23.86	8.2762
1246	63	1	3	0	82	22.21	2	31	0	17.5	11.08	52.16	22.18	7.5672
1247	62	2	0	0	83	25.63	2	22	0	42	25.03	34.55	14.41	6.1144
1248	59	2	0	1	80	24.97	1.5	27	0	44.1	26.42	33.84	14.68	6.1116
1249	54	2	0	0	78	24.50	2	23	1	40.2	24.48	36.44	15.32	5.9773
1250	74	1	0	1	87	23.93	2	28	1	26.5	16.58	46.05	19.93	7.5112
1251	53	1	3	1	82	23.81	2	39	0	21.5	12.96	47.20	21.04	8.1084
1252	60	2	0	0	74.5	21.89	1.5	27	0	35.7	19.52	35.23	14.77	5.7336
1253	50	1	0	1	81	21.97	1.5	40	0	19	11.34	48.40	21.25	7.8705
1254	60	2	0	0	79	24.18	1.15	31	0	32.2	18.68	39.30	15.93	6.5606
1255	54	1	3	0	90	26.74	2	36	0	31.5	22.39	48.59	21.23	7.8085
1256	62	1	1	0	81	21.48	2	45	0	17.2	10.57	50.98	22.66	7.6757
1257	61	1	3	0	87	25.52	2	34	0	24.6	17.26	53.00	23.89	8.5867
1258	61	2	0	0	91	29.18	1.5	20	0	40.6	25.80	37.81	14.75	6.7244
1259	59	2	0	1	87	28.51	1.5	24	0	44.2	27.70	34.90	14.36	6.3572
1260	77	1	0	1	92	22.60	1.5	30	0	19.8	11.34	45.87	19.35	7.3994
1261	55	2	0	0	73	21.85	2	21	0	33	18.14	36.81	16.20	6.2665
1262	55	2	0	0	81	20.64	2	25	1	31.7	15.40	33.16	13.35	5.5006
1263	53	2	0	0	80	24.27	2	30	0	37.1	21.10	35.78	15.83	6.6909
1264	76	2	0	0	76	25.12	1.2	21	1	36	20.54	36.47	15.40	6.5795
1265	53	1	3	0	102	34.54	2	35	0	37.5	36.81	61.29	25.91	8.7973
1266	55	2	1	1	66	19.58	1.5	28	0	29.9	15.25	35.66	14.88	5.6154
1267	57	2	0	0	75	23.45	1.5	17	1	40.9	22.63	32.76	13.75	5.6565
1268	53	1	0	0	84	24.57	2	43	0	19.6	12.06	49.46	22.68	8.8574
1269	54	2	0	1	64	19.66	2	32	0	27.8	13.80	35.76	15.25	5.9122
1270	70	1	3	1	74	20.46	1.2	25	2	20.7	9.76	37.30	15.19	6.5738
1271	63	2	0	0	90	33.56	1.2	16	1	51.6	38.62	36.21	14.65	6.6063
1272	67	1	0	0	89	25.62	1.2	32	1	24.2	16.35	51.15	23.30	8.6521
1273	62	2	0	0	75	26.17	0.67	21	1	40.2	21.59	32.12	13.46	6.4266
1274	56	2	0	0	76	21.22	2	27	0	38.2	19.88	32.21	13.31	5.3576
1275	59	1	1	0	84	23.31	1.5	34	0	23.3	13.23	43.56	19.23	7.6345
1276	77	2	0	0	97	27.61	1.5	19	1	43.2	27.04	35.56	11.73	5.0417
1277	53	2	0	0	84	27.31	2	25	0	44.6	31.47	39.12	17.50	6.6102
1278	59	2	0	0	82	26.63	1.5	28	0	40.3	23.56	34.83	14.88	6.6479
1279	61	1	1	1	90	26.92	1.5	33	1	30.5	22.31	50.85	23.01	8.2498
1280	53	2	0	0	69	22.59	2	24	0	32.4	17.23	35.98	15.37	6.4045
1281	65	2	0	0	88	28.49	1.5	24	0	44.9	28.46	34.91	14.28	6.459
1282	71	1	0	0	92	26.06	2	24	1	27.6	19.17	50.20	11.98	4.3833
1283	71	1	0	0	89.5	25.51	2	41	0	28.3	23.84	60.50	27.23	8.2309
1284	58	2	0	0	80.5	27.67	2	32	0	41.8	28.27	39.37	16.11	6.3305
1285	58	2	0	0	95	32.80	1.2	22	0	46.9	36.41	41.25	18.43	7.6428
1286	52	1	3	0	99	26.38	2	39	0	31.3	22.84	50.12	21.87	7.7947
1287	70	1	0	0	97	29.75	2	38	0	31.9	25.85	55.10	24.48	8.9906
1288	61	2	0	0	69	22.67	1.2	27	0	38.3	20.66	33.24	13.88	6.168
1289	53	1	1	1	84	26.16	1.5	44	0	29	20.04	49.16	21.26	7.9253
1290	53	2	0	0	77.5	24.82	1.5	23	0	44.8	23.18	28.51	10.86	5.0962
1291	52	1	0	1	87	27.68	1.5	50	1	28.1	21.23	54.18	25.23	8.8876
1292	83	1	0	0	87	23.69	1.2	22	1	36.3	22.64	39.78	16.39	6.3234
1293	76	1	0	0	89	25.57	1.2	26	1	32.1	20.73	43.82	19.92	7.6381
1294	73	2	0	0	82	24.70	1.5	16	1	31.6	18.26	39.55	16.99	7.2115
1295	58	2	0	1	78.5	22.60	2	27	0	40.5	22.91	33.72	14.79	5.761

1296	59	2	0	0	80	28.78	1.2	30	0	44.9	28.30	34.80	15.66	7.0528
1297	66	1	0	1	85	24.93	1.2	37	1	24.5	17.15	52.73	24.26	8.4235
1298	69	2	0	0	85	27.82	1.5	20	0	37.8	24.27	39.99	17.74	7.5774
1299	55	2	0	1	90	30.51	2	23	0	48.9	35.42	36.97	16.69	6.7785
1300	58	1	0	0	78	23.13	2	38	1	26.1	16.78	47.55	21.36	7.4979
1301	84	2	0	0	86.5	25.20	0.35	11	2	43.8	26.73	34.31	13.91	5.6058
1302	79	2	0	1	92.5	28.91	0.67	14	2	45.7	29.27	34.79	13.73	5.9431
1303	71	1	3	0	86	24.54	1.5	25	1	28.4	18.49	46.64	20.16	7.4497
1304	58	2	0	1	87	28.60	1.5	27	0	42.8	30.55	40.81	17.41	6.9732
1305	71	1	0	1	72	21.29	3	32	0	22.5	12.24	42.18	18.09	6.8764
1306	60	1	1	1	79	20.71	1.5	38	0					
1307	50	2	0	0	72	22.30	1.5	33	0	28.3	15.47	39.11	17.10	6.7132
1308	59	1	1	1	90	25.51	2	40	0	27.2	18.99	50.80	24.13	8.5684
1309	57	1	1	1	78	22.51	1.2	26	2	8.7	4.73	49.33	21.84	8.6716
1310	57	1	3	1	84	26.49	2	40	0	22.1	15.73	55.58	25.12	9.2027
1311	54	2	0	0	95	24.93	2	26	0	34.4	21.63	41.19	18.44	7.2405
1312	52	2	0	0	72	23.77	1.5	34	0	36.7	21.28	36.68	15.99	6.4219
1313	72	2	3	0	89	29.11	0.86	19	1	43	27.71	36.77	13.78	6.2492
1314	54	2	3	0	80	22.06	1.5	19	0	33.6	16.39	32.37	12.10	5.41
1315	62	2	0	0	92	27.65	1.2	29	0	40.1	27.06	40.38	17.26	6.97
1316	73	1	3	1	96	26.53	1.5	31	1	23.5	16.87	54.81	24.40	8.75
1317	53	1	0	1	99	28.97	3	47	0	32.1	27.75	58.61	28.23	9.4
1318	75	2	0	0	72	24.17	1	29	1	42.4	25.00	33.92	14.52	5.84
1319	66	1	0	1	90	24.25	2	34	0	23.6	16.06	52.07	23.56	8.38
1320	62	1	1	1	96	26.51	1.5	31	0	35.7	24.84	44.68	19.75	7.41
1321	57	1	0	0	81	24.54	2	45	0	26.4	17.47	48.74	22.45	8.29
1322	52	2	0	0	73	26.27	1.5	29	0	37.9	22.09	36.14	15.41	6.85
1323	60	1	1	1	80	21.72	2	39	0	21.5	13.03	47.69	20.93	7.41
1324	63	2	0	0	90	31.00	1.5	31	0	42.4	29.88	40.63	17.99	7.5
1325	54	1	1	0	90	27.64	2	43	0	27	22.97	62.16	29.69	9.55
1326	85	1	0	1	87	23.00	1.5	23	1	15.9	10.73	56.94	24.34	8.39
1327	70	1	0	0	99	28.75	1.2	27	2	30.7	24.23	54.67	23.61	8.22
1328	67	2	0	1	79	23.80	2	11	1	39	22.25	34.81	15.27	6.3
1329	55	1	3	1	94	27.47	1.5	47	0	31.9	22.31	47.65	22.08	8.41
1330	60	1	0	1	88	27.95	1.5	39	0	32.8	23.02	47.14	22.17	8.49
1331	63	1	0	1	79	24.42	1.5	29	1	21.9	12.93	46.14	20.14	8.31
1332	55	2	0	0	82	26.56	2	18	0	41.8	26.80	37.32	16.40	6.77
1333	67	2	0	0	82	25.55	1.5	19	0	38.6	24.88	39.64	16.80	6.56
1334	53	1	0	1	121	43.54	2	51	0	41.8	48.91	68.02	28.44	9.99
1335	62	2	0	0	69	23.88	2	22	1	35.8	18.61	33.39	14.09	6.37
1336	69	2	0	0	85	24.89	1.2	32	0	36.9	22.93	39.25	17.39	6.91
1337	70	2	0	0	86	28.81	1.2	16	1	42.9	25.57	34.07	14.62	7.02
1338	56	1	0	0	80	25.16	2	48	0	24.7	18.81	57.34	25.62	8.23
1339	67	1	3	0	84	23.85	1	42	2	25.3	16.02	47.30	19.95	7.52
1340	55	2	0	0	95	26.51	2	18	0	35.7	21.89	39.41	16.55	6.99
1341	70	2	0	0	88	22.92	1.5	25	0	38.1	21.41	34.80	14.93	6.03
1342	60	2	0	0	67	17.51	1.5	26	0	23.4	10.83	35.45	14.97	5.6
1343	55	1	1	0	79	22.38	2	36	1	25.3	15.25	45.06	20.39	7.44
1344	55	1	3	0	87	25.27	1.5	28	1	27.8	19.30	50.05	22.53	8.04
1345	55	1	0	1	89	26.07	2	39	0	30.4	19.05	43.65	18.71	7.55
1346	62	2	0	1	64	19.40	1.2	16	1	33	15.61	31.69	12.88	5.17
1347	59	1	3	0	85	24.99	2	50	0	29.1	22.11	53.91	24.25	7.96
1348	52	2	0	0	75.5	22.02	2	23	0	38.6	20.53	32.70	14.48	5.98
1349	58	2	0	0	70	19.45	1.5	21	0	23.6	11.18	36.19	14.69	6.03
1350	61	2	0	0	89	30.07	1.5	23	0	43.7	31.57	40.68	17.87	7.53
1351	80	1	1	0	82.5	22.44	1.5	32	0	22.9	12.81	43.09	18.22	7.29
1352	72	2	0	0	76	21.44	1.5	22	0	34.4	17.69	33.69	14.08	5.81
1353	75	2	0	0	89	24.71	1.5	22	0	34.1	20.17	39.01	15.73	6.45
1354	59	1	0	1	85	24.00	2	55	0	20.1	14.68	58.34	25.73	8.52
1355	62	1	0	1	95.5	31.50	2	50	0	27.9	25.06	64.67	28.90	10.05
1356	66	2	0	1	70	22.61	2	23	0	39.9	22.05	33.15	15.14	6.19
1357	61	1	0	0	100	31.11	1.5	42	1	35.4	32.23	58.83	26.23	8.77
1358	54	2	0	1	75	23.40	1.5	29	0	43.6	25.37	32.75	13.33	5.29
1359	65	2	0	0	89	27.01	2	22	0	44.9	26.75	32.84	14.99	6.68
1360	70	1	0	1	86	25.91	1.5	42	0	28.3	17.14	43.44	19.81	8.26
1361	64	1	0	0	93	26.96	2	35	0	27.9	20.41	52.75	23.01	8.37
1362	62	2	0	1	93.5	27.23	1.2	20	0	40.6	25.98	38.02	14.99	6.38
1363	57	1	3	1	85	27.18	2	39	0	28.9	21.75	53.39	25.28	8.95
1364	56	1	0	0	90	29.28	3	40	0	39.1	28.87	45.05	20.38	7.71
1365	52	1	1	1	85	24.73	2	36	0	23.4	16.37	53.49	24.02	8.26
1366	52	2	0	0	78	23.06	2	24	0	30.8	16.37	36.84	15.02	6.42
1367	59	2	0	0	95	31.57	1.2	24	0	47.6	33.08	36.38	14.81	6.82

1368	75	2	0	0	78	24.00	0.86	13	2	42.9	24.58	32.68	13.67	5.7
1369	57	1	1	1	86.5	25.38	1.5	42	0	26.9	19.56	53.10	24.02	8.28
1370	54	2	0	1	79	24.79	1.5	30	0	32.5	18.23	37.89	17.08	7.48
1371	76	1	3	0	84	27.37	1.2	32	1	30.6	19.31	43.72	20.21	7.99
1372	72	1	0	0	87	24.24	0.6	24	2	27.9	17.33	44.81	18.97	7.38
1373	60	2	0	0	79	23.97	1.5	14	1	31.6	16.39	35.53	13.60	6.15
1374	57	2	0	0	75	22.07	2	21	0	32.6	18.24	37.65	16.79	6.64
1375	61	2	0	0	94	27.50	1.5	18	1	36.4	26.19	45.82	20.13	7.51
1376	73	1	0	1	83	22.96	2	34	0	21.7	12.65	45.70	19.76	7.61
1377	57	2	0	0	85	25.07	2	16	1	44.1	27.59	34.99	15.22	6.03
1378	58	1	0	0	85	22.44	2	42	0	24.3	15.09	46.93	21.75	7.66
1379	63	2	0	0	79	20.95	1.5	18	0	41.6	21.46	30.14	12.55	4.97
1380	58	2	0	0	89.5	27.47	2	27	0	37.1	28.01	47.47	21.05	7.43
1381	72	2	0	0	70.5	20.60	1.2	22	0	32.5	14.74	30.61	12.17	5.57
1382	58	2	0	0	74	22.16	1.5	16	1	33.3	16.68	33.38	14.50	6.3
1383	53	2	0	0	81	20.63	2	23	0	32.9	16.08	32.78	13.95	5.73
1384	52	1	0	1	78	21.22	2	33	0	20	10.76	42.96	18.91	7.29
1385	63	1	1	1	104	31.71	1.5	32	0	37.5	30.35	50.54	21.94	8.46
1386	54	1	0	1	82	24.26	2	43	0	24	14.88	47.00	21.12	8.11
1387	56	2	0	0	90	22.29	2	21	1	37.9	19.47	31.89	13.54	5.86
1388	60	2	0	0	83	23.18	2	18	1	38.8	21.08	33.18	13.57	5.68
1389	61	1	1	1	70	23.93	1.5	33	0	14	7.45	45.86	20.09	8.74
1390	59	2	0	1	110	33.10	2	30	0	52	37.22	34.33	14.31	6.48
1391	58	1	0	0	87	25.34	3	33	0	28	19.95	51.30	22.87	7.95
1392	53	1	0	0	82.5	26.85	1.5	39	0	26.1	19.06	53.88	24.60	8.95
1393	75	1	0	0	95	24.37	1.2	27	1	32.9	21.65	44.23	19.93	7.16
1394	65	2	0	0	68	20.73	1	12	1	37.7	17.53	28.94	11.70	5.19
1395	54	2	0	0	67	18.98	1.5	29	0	26.5	11.86	32.96	14.56	6.12
1396	58	1	0	0	91	26.58	2	41	0	23.4	16.94	55.39	25.40	9.26
1397	56	1	1	1	93	27.93	2	41	1	29.6	24.34	57.99	25.85	8.65
1398	55	2	0	0	88	24.06	2	23	0	38.4	20.31	32.54	12.52	5.71
1399	57	1	1	1	82	24.25	2	41	0	21.1	13.05	48.85	20.98	8.06
1400	79	1	0	1	92	23.86	0.75	25	2	33.2	21.56	43.43	18.40	6.65
1401	70	1	0	0	85	23.26	1.5	36	1	29.8	19.75	46.49	19.98	6.83
1402	59	2	1	0	77	24.79	1.5	25	0	43.5	27.58	35.83	15.84	6.17
1403	57	2	0	0	91	28.20	1.2	19	1	45.6	27.60	32.97	12.87	5.94
1404	54	1	0	1	79	22.18	2	38	0	24.8	14.96	45.28	20.78	7.47
1405	62	2	0	0	82	25.55	1.2	22	0	42.9	25.91	34.45	14.75	6.15
1406	76	1	0	0	78.5	21.65	2	29	0	18.4	10.47	46.28	20.11	7.75
1407	53	2	0	1	79	24.37	2	17	1	26.8	15.34	42.00	17.83	7.44
1408	72	2	0	0	80	21.60	0.86	8	1	23.6	11.41	36.85	16.37	7.38
1409	63	2	0	0	87	24.30	1.5	26	1	39.3	22.27	34.42	14.98	6.37
1410	56	1	1	0	75	21.36	2	32	0	11.1	6.82	54.48	23.77	8.25
1411	55	1	3	1	83	27.41	3	53	0	27.7	21.83	56.89	26.28	9.06
1412	53	1	1	0	72	18.79	1.5	40	0	13.6	6.81	43.19	18.51	6.88
1413	79	2	0	0	88	28.88	0.75	7	2	37	21.54	36.74	13.57	6.71
1414	79	1	0	0	77	22.74	1	18	2	20.4	10.18	39.83	16.78	7.66
1415	57	2	0	0	79	24.47	1.5	22	1	30.3	18.49	42.51	18.28	7.33
1416	54	2	0	0	71.5	23.23	3	28	1	37.7	20.55	34.02	15.17	6.4
1417	50	2	0	0	83	21.02	2	25	0	25	12.22	36.64	15.28	6.53
1418	55	1	3	1	68	19.54	2	44	0	8.3	4.72	52.24	23.48	7.99
1419	56	1	3	0	92	29.39	2	43	0	29.1	23.18	56.41	24.62	8.93
1420	54	2	0	1	74	20.95	2	14	1	42.1	21.31	29.29	12.45	5.06
1421	81	1	0	0	94	25.74	2	25	1	32.1	21.04	44.43	19.05	7.18
1422	80	2	0	0	74	20.38	1.5	14	1	30.6	13.30	30.11	11.57	5.2
1423	60	2	0	0	84	26.01	1.5	15	1	38.9	25.26	39.67	16.45	6.38
1424	57	1	3	0	86	24.80	2	47	1	21.1	14.62	54.69	24.71	8.51
1425	62	2	0	0	99	30.56	1.2	19	1	48.3	34.20	36.56	16.62	7.1
1426	63	1	0	0	103	29.69	2	28	1	36.5	29.82	51.97	21.98	7.92
1427	55	2	0	0	78	22.76	2	25	1	35.6	18.87	34.13	14.63	6.17
1428	64	2	0	0	86.5	28.57	1.5	24	1	44.6	28.51	35.35	14.02	6.07
1429	54	1	0	0	86	25.25	2	48	0	25.4	17.26	50.71	23.81	8.53
1430	56	1	3	0	83	25.84	2	51	0	24	16.38	52.01	23.34	8.6
1431	59	2	0	0	88	24.92	1.5	19	0	31.4	17.77	38.85	16.05	6.95
1432	64	1	0	0	83.5	23.43	1.5	43	1	31.4	20.69	45.18	18.16	6.21
1433	57	2	0	0	92	33.78	1.5	17	1	51.6	37.42	35.12	15.10	6.89
1434	79	1	0	0	94.5	26.56	1.5	26	1	27.8	21.98	57.19	25.10	8.37
1435	55	1	0	0	79	19.96	3	36	0	19.6	11.06	45.47	20.31	7.11
1436	55	2	0	0	60	14.85	2	17	1	23.2	8.57	28.41	11.38	4.5
1437	55	1	1	1	87	28.46	2	44	0	30	24.56	57.34	24.90	8.42
1438	50	2	0	0	72	22.76	2	22	1	31.8	17.71	37.92	16.85	6.77
1439	66	1	0	0	72	21.77	2	44	0	11.2	6.09	48.15	22.18	8.65

1440	74	2	0	0	70	19.63	1.5	22	1	20.9	9.11	34.48	13.14	6
1441	52	1	0	1	90	22.59	2	42	0	22.5	15.06	52.00	23.66	7.65
1442	54	2	0	0	92	28.21	1.5	15	1	47.9	30.02	32.60	13.62	5.97
1443	58	2	0	0	82	24.26	2	22	0	37.5	20.85	34.70	15.19	6.36
1444	61	1	0	0	81	25.64	2	30	0	26.8	16.06	43.87	20.16	8.23
1445	54	2	0	1	77	26.85	2	25	1	40.1	24.50	36.58	15.13	6.63
1446	58	2	0	0	67	20.09	1.39	20	1	35.7	16.54	29.81	12.69	5.44
1447	54	2	0	1	76	24.17	1.92	25	0	40.2	26.09	38.88	16.87	6.27
1448	68	2	0	0	75	18.86	1.7	20	1	28.8	13.12	32.52	13.55	5.65
1449	61	1	0	1	76	22.15	1.71	40	1	23.2	15.41	50.96	23.24	7.57
1450	73	1	3	1	82.5	19.01	1.27	35	0	11.7	5.61	42.50	18.29	6.97
1451	71	2	0	0	83	28.17	0.95	15	2	42.1	26.18	36.02	14.43	6.36
1452	54	1	1	1	75	22.72	2.13	47	0	14.4	8.92	53.09	24.74	9.07
1453	51	2	0	1	77	22.73	1.88	22	0	36.2	18.44	32.48	13.82	6.14
1454	53	2	0	0	78	21.93	1.4	18	0	34.7	20.59	38.79	17.06	6.24
1455	59	1	0	0	82	25.36	2	32	1	23.5	14.13	46.02	19.69	8.23
1456	65	1	1	0	89	27.58	0.93	34	1	25.6	17.88	51.84	23.21	8.96
1457	54	2	0	0	69	21.43	2.52	31	1	31.6	17.97	38.93	16.91	6.28
1458	56	2	0	0	68	20.90	1.5	28	0	37.7	19.28	31.83	13.76	5.48
1459	54	1	1	1	90	25.58	1.75	48	0	33.3	25.31	50.75	23.56	7.74
1460	54	2	0	0	82	24.17	1.83	28	1	38.9	24.64	38.66	14.02	5.21
1461	57	2	0	0	97.5	38.28	1.19	29	0	53.1	45.75	40.44	17.03	7.34
1462	58	2	0	0	68.5	19.38	1.72	23	0	24.7	12.17	37.07	17.41	6.84
1463	51	2	0	0	83	26.94	1.37	18	1	39.7	26.28	39.85	16.88	6.56
1464	71	2	0	0	70	21.33	1.12	18	0	31.1	16.16	35.83	14.58	6.02
1465	73	2	0	0	77	23.42	1.35	16	1	32.4	16.65	34.68	14.76	6.65
1466	58	1	3	0	78	22.07	2.04	36	0	17	11.24	55.02	25.49	8.37
1467	58	2	0	0	74	22.19	2.18	29	0	43	25.19	33.38	14.22	5.22
1468	57	2	0	0	70	19.76	1.74	25	0	24.5	11.62	35.80	15.18	6.24
1469	54	2	0	0	72	23.86	1.56	31	0	37.1	20.04	33.93	14.39	6.19
1470	63	1	0	0	92	25.12	1.35	40	1					
1471	64	1	0	0	90	23.26	1.14	40	1	22.9	16.37	55.11	24.19	7.78
1472	56	1	1	0	79	26.21	1.1	34	1	26.1	16.35	46.32	20.10	8.39
1473	61	2	0	0	76	24.49	1.85	26	0	42.2	25.35	34.77	13.99	5.69
1474	54	1	0	0	80	21.36	1.4	38	0	22.3	12.41	43.26	17.96	6.91
1475	51	2	0	0	90	25.46	1.71	25	0	36.4	25.85	45.17	19.80	7.1
1476	79	2	0	0	73	18.85	1	13	1	18.7	7.23	31.40	12.71	5.93
1477	78	1	1	0	90	21.41	0.85	17	2	15	8.56	48.53	19.32	7.01
1478	80	2	0	1	78	20.41	1.2	11	1	32.5	17.01	35.27	13.40	5.17
1479	60	1	1	1	80	21.46	1.64	32	1	25	14.91	44.66	19.67	6.98
1480	52	2	0	0	87	24.65	1.54	28	1	31	17.95	39.87	17.41	7.32
1481	58	1	3	1	89	25.21	1.2	34	1	21.6	15.11	54.85	23.82	8.54
1482	57	2	0	0	85	21.53	1.53	17	1	34.4	16.83	32.16	13.23	5.72
1483	55	2	0	0	80	22.50	1.58	27	0	39.8	21.05	31.84	13.37	5.48
1484	60	2	0	0	78	21.46	1.49	15	1	34.8	15.39	28.79	12.10	5.71
1485	58	1	3	0	85.5	26.39	1.93	33	1	28.8	20.69	51.21	23.04	8.34
1486	85	1	0	0	100	28.36	1.45	25	1	29.7	22.69	53.59	21.67	7.8
1487	53	2	0	0	94	29.20	1.01	23	1	44.1	29.35	37.23	15.40	6.62
1488	59	2	0	0	70	25.20	1.38	22	1	37.7	19.20	31.67	13.57	6.56
1489	59	1	0	0	74	22.11	1.08	29	1	19	11.23	47.75	20.72	7.47
1490	72	1	3	0	103	29.13	1.69	36	0	34.4	26.50	50.64	22.48	8.23
1491	65	1	0	0	94	23.31	2	38	0	26.9	19.92	54.03	24.19	7.61
1492	56	2	0	0	67.5	19.03	1.32	13	1	24.8	11.21	34.03	14.30	6.06
1493	55	1	0	0	87	26.20	1.67	43	0	27.3	19.01	50.67	23.34	8.47
1494	54	2	0	0	67	20.73	2.43	23	0	28.6	13.32	33.27	13.95	6.23
1495	71	1	0	1	85	24.38	1.85	32	0	18	12.02	54.65	23.81	8.33
1496	55	2	0	0	80	23.28	2.34	21	1	35.7	19.60	35.27	14.42	5.88
1497	63	2	0	0	85.5	25.85	1.25	13	1	41.2	23.33	33.33	14.17	6.24
1498	59	2	0	0	77.5	24.43	1.19	23	0	34.2	18.11	34.80	14.99	6.7
1499	58	1	0	0	79	21.85	1.44	22	1	28.8	17.05	42.09	18.73	6.56
1500	54	2	0	1	67	20.13	2.75	24	1	31.5	14.36	31.17	13.19	5.42
1501	57	2	0	0	84	23.40	1.5	30	1	37.7	23.04	38.13	16.40	6.02
1502	72	2	0	0	79.5	25.31	0.95	9	2	39.4	20.55	31.59	12.72	6.17
1503	50	2	0	1	60	18.96	1.67	27	0	25.8	11.57	33.31	14.78	5.84
1504	66	2	0	0	76	21.66	1.09	16	2	39.7	21.09	32.01	13.66	5.48
1505	63	2	0	0	92	26.37	1.06	19	1	39.8	24.48	37.05	13.97	5.87
1506	72	1	3	1	91	28.29	1.45	32	0	25	78.58	55.81	25.07	9.18
1507	55	1	0	0	86	23.88	1.45	37	0	29.3	18.65	44.97	19.31	7.17
1508	50	2	0	0	72	23.59	1.75	23	1	39.4	23.45	36.03	15.80	6.23
1509	61	1	0	1	94	24.03	1.6	31	1	23.9	15.96	50.77	22.50	7.76
1510	60	2	0	0	76	20.34	1.33	29	0	32.2	17.28	36.39	15.55	5.76
1511	55	1	0	1	83	24.66	1.25	28	1	31.9	21.23	45.32	20.38	7.41

1512	69	2	0	0	82	30.22	1.09	18	1	44.6	26.02	32.38	13.96	6.21
1513	69	1	3	1	75	21.72	1.26	17	1	9.3	5.10	49.63	22.15	8.44
1514	79	1	1	0	88	23.68	0.94	16	2	21.3	13.13	48.49	22.22	8.22
1515	70	2	0	0	74	24.91	1.02	16	1	34.6	18.29	34.61	12.67	5.56
1516	62	1	1	1	95.5	27.77	1.03	31	1	31	23.51	52.26	22.55	8.28
1517	55	2	0	0	82	27.49	1.49	21	0	38.1	23.65	38.47	16.27	6.86
1518	76	2	0	0	94	26.25	1.14	27	1					
1519	63	1	1	0	83	22.30	1.51	35	0	20.3	12.85	50.53	22.57	7.72
1520	56	2	0	0	89	27.71	1.24	31	0	42.9	30.71	40.83	17.53	6.5
1521	50	2	0	0	93	26.96	1.48	26	1	32.8	19.18	39.36	16.61	7.25
1522	77	1	1	0	90.5	24.06	2	30	0	24.6	15.76	48.42	22.00	8.17
1523	55	1	3	1	90	26.88	2.06	35	0	28.4	22.31	56.26	24.32	8.21
1524	55	2	0	0	72	23.56	1.38	8	1	37.7	21.73	35.85	15.11	6.11
1525	59	2	0	0	80	22.41	1.29	24	0	32.5	18.39	38.11	16.51	6.46
1526	59	2	0	0	91	24.52	1.56	25	0	33.3	21.49	42.97	18.09	6.85
1527	60	2	0	0	62	21.23	1.22	21	0	32.1	14.54	30.73	12.39	5.66
1528	52	2	0	0	78	21.99	1.35	24	0	32.8	17.73	36.25	15.61	6.15
1529	66	2	0	0	96	25.28	0.94	14	2	36	18.55	32.94	14.18	6.97
1530	71	2	0	0	74	22.07	1.08	21	1	28.3	14.96	37.85	16.56	6.69
1531	68	2	0	0	71	20.83	1.48	24	0	31.1	15.86	35.11	14.84	5.94
1532	73	1	3	0	104	25.86	0.42	27	2	33.9	23.10	45.06	19.39	7.12
1533	63	2	0	0	74	22.59	1.63	21	0	36.8	19.14	32.85	14.16	6
1534	65	2	0	0	82	26.55	1.11	16	1	41.7	23.11	32.32	12.41	5.75
1535	70	2	0	0	69.5	20.87	1.55	18	0	28.6	14.29	35.74	14.20	5.88
1536	52	2	0	1	88	25.35	1.4	27	0	41.1	27.39	39.26	17.13	6.53
1537	52	2	0	0	91	25.94	1.26	17	1	39.7	21.55	32.78	13.67	6.45
1538	77	1	0	1	86	22.98	0.98	21	1	26.3	14.92	41.90	17.18	6.93
1539	52	2	0	0	79	22.29	1.57	25	0	35.4	17.07	31.16	12.71	5.54
1540	65	1	1	1	98	26.60	1.33	31	0	30	21.25	49.52	21.89	7.95
1541	77	1	3	0	84.5	22.91	1.55	30	0	25.2	14.64	43.45	19.17	7.33
1542	57	1	1	1	90	24.23	1.3	39	1	24.2	17.34	54.40	23.86	7.9
1543	54	2	0	0	94	30.05	1.3	21	0	43.2	31.49	41.45	17.95	7.15
1544	58	1	1	0	82	20.36	1.53	40	1	15.3	8.78	48.54	19.01	6.52
1545	71	2	0	0	76	21.34	1.24	20	0	29.9	15.90	37.21	16.11	6.24
1546	74	1	3	0	72.5	21.94	0.93	26	1	24.3	13.41	41.76	17.73	6.76
1547	61	2	0	0	98	29.93	1.04	12	2	40.1	24.92	37.20	12.91	6.03
1548	67	1	0	0	87	24.95	1.61	35	1	23.5	15.49	50.39	22.57	8.5
1549	75	2	0	1	96.5	26.76	0.72	16	1	36.4	20.24	35.41	14.28	6.72
1550	77	1	3	1	89	25.53	1.16	34	1	23.3	14.63	48.05	19.89	7.95
1551	50	2	0	0	98.5	34.71	1.16	16	2	53	40.90	36.31	16.00	6.9
1552	53	2	0	0	72	23.13	1.41	28	1	37	19.46	33.10	13.54	5.82
1553	61	1	3	1	78	21.76	2	34	0	16.5	9.15	46.30	20.11	8.04
1554	57	2	0	0	94.5	23.81	1.74	23	1	37.7	20.35	33.68	14.14	6.14
1555	53	2	0	0	76	25.77	1.38	23	0	37.6	21.30	35.34	14.70	6.53
1556	58	2	0	0	78	23.56	1.83	26	0	38.5	21.58	34.54	15.37	6.28
1557	57	1	1	0	88.5	25.62	1.6	43	0	29	22.10	54.13	24.09	7.99
1558	56	2	0	0	59.5	18.30	1.57	14	1	21.4	9.22	33.88	14.53	6.06
1559	71	1	0	1	93.5	26.17	1.81	34	0	33.2	22.96	46.19	19.31	7.31
1560	66	2	0	0	88	24.22	1.43	18	0	36.5	20.35	35.36	14.41	6.15
1561	61	2	0	0	82	25.86	1.52	22	0	41.6	24.64	34.62	14.23	5.99
1562	56	2	0	0	88	32.66	1.55	17	1	50.5	33.83	33.13	13.45	6.29
1563	55	1	3	0	103	32.57	1.52	42	0	34	31.99	62.23	27.48	9.44
1564	59	2	0	0	83.5	21.10	1.41	27	0	28.6	13.49	33.70	14.80	6.53
1565	77	2	0	0	79	25.00	1.48	29	0	31	19.33	42.98	18.97	7.08
1566	53	2	0	0	78	23.93	2.03	20	0	37.2	20.86	35.28	14.48	6.03
1567	62	2	0	0	79	23.77	1.18	23	0	34.2	17.51	33.67	14.24	6.51
1568	74	2	0	0	81	23.75	1.51	19	0	39	23.09	36.06	14.95	6.02
1569	64	1	1	1	78	20.68	1.5	28	1	15.5	8.33	45.28	18.91	6.97
1570	63	2	0	0	84	25.00	1.64	27	0	31.7	19.52	41.99	18.84	7.55
1571	69	2	0	0	81	24.62	1.53	25	0	41.8	25.92	36.11	13.44	5.24
1572	53	1	1	1	74	21.05	1.12	45	1	7.9	4.44	51.55	22.11	7.93
1573	75	2	0	0	84.5	17.31	0.9	14	1	30.2	11.90	27.49	10.73	4.62
1574	52	1	0	1	101	35.57	1.32	40	0	33.1	29.31	59.33	27.69	10.87
1575	70	1	3	0	72	18.81	1.23	22	1	13.6	6.97	44.18	18.96	6.74
1576	69	2	0	0	97	25.50	0.98	16	1	34.1	17.47	33.75	15.06	7.31
1577	52	1	1	1	101	31.78	1.86	45	0	34.3	31.28	59.81	25.78	8.91
1578	51	2	0	1	88.5	23.17	1.49	35	0	37.5	21.93	36.58	15.36	5.91
1579	55	1	0	1	67.5	21.79	1.65	42	0	16.3	9.17	47.21	21.07	8.07
1580	51	1	0	0	77.5	21.86	2.28	35	0	22.8	12.73	42.19	19.32	7.37
1581	73	1	0	1	85	24.06	1.44	31	0	23.5	14.06	45.83	19.85	7.58
1582	73	2	0	0	82	24.56	1.24	15	1	33.1	17.15	34.73	15.32	6.93
1583	59	1	3	0	75	21.17	1.21	34	0	15.5	8.38	45.65	19.92	7.84

1584	58	2	0	0	80	23.65	1.59	32	1	34.4	22.05	41.96	18.79	6.83
1585	86	1	0	0	97	25.64	0.7	19	2	35.3	22.08	40.48	15.01	5.88
1586	54	1	1	1	70	19.21	1.64	41	0	18	8.96	40.87	18.41	6.92
1587	63	1	0	1	88	25.72	1.85	40	0	25.9	16.22	46.35	20.87	8.13
1588	56	2	0	1	72	19.77	1.36	24	0	28.8	13.00	32.11	13.12	5.59
1589	53	2	0	1	67	21.78	1.69	30	0	28.5	16.02	40.19	18.46	7.04
1590	76	1	0	0	74	21.60	0.86	14	1	22.2	11.40	39.99	17.54	6.97
1591	70	2	0	0	78.5	27.88	0.91	15	1	44.5	24.06	29.96	12.12	6
1592	74	2	0	0	103	29.56	0.95	9	2	44.3	26.68	33.50	13.68	6.42
1593	58	2	0	0	79.5	26.14	1.39	26	1	48.3	32.84	35.08	15.08	5.75
1594	59	1	1	1	84	22.69	1.56	38	1	25	15.19	45.48	18.66	6.94
1595	51	1	1	0	84.5	27.48	2.02	55	0	28	20.22	51.89	25.47	9.47
1596	52	1	1	1	86.5	25.61	2.02	45	0	22.9	14.45	48.51	21.15	8.45
1597	51	1	0	0	75	34.59	1.5	38	0	16.2	7.72	40.05	15.97	6.35
1598	68	2	0	0	101	30.55	1.21	25	1	40	27.69	41.51	18.05	7.83
1599	60	2	0	0	64.5	22.31	1.27	22	0	33.6	15.17	29.93	12.76	6.26
1600	62	2	0	0	93	31.40	1.1	19	1	49.2	31.26	32.33	12.31	5.93
1601	73	2	0	0	78	23.29	1.36	20	1	38.4	20.61	33.08	12.03	5.09
1602	80	1	3	1	96	28.47	1.37	32	0	29.3	20.63	49.77	20.92	8.3
1603	76	2	0	0	70	17.39	1.17	15	1	21.3	8.43	31.12	12.72	5.78
1604	68	2	0	0	75	21.19	1.05	21	1	34.7	16.04	30.11	11.92	5.46
1605	66	2	0	0	90	22.64	1.12	23	0	36.1	19.31	34.20	14.42	6.16
1606	79	1	3	1	100	29.34	1.19	31	1	33.2	26.87	54.13	25.10	8.79
1607	52	1	1	0	80	20.45	1.38	37	0	18.7	9.75	42.50	18.31	6.61
1608	73	1	1	1	91	23.10	1.56	35	0	22.9	15.07	50.69	21.62	7.25
1609	56	1	1	1	98	28.01	1.57	42	0	34.4	28.63	54.52	25.97	8.57
1610	68	1	0	0	76	22.36	1.83	31	1	19.4	10.81	44.86	18.90	7.3
1611	52	2	0	1	68	22.28	1.83	27	0	37.7	21.25	35.10	15.51	6.02
1612	62	2	0	0	67	18.53	1.65	19	0	33.5	14.31	28.38	11.67	4.96
1613	62	2	0	0	87	26.96	1.08	30	1	39.7	23.22	35.25	15.46	6.98
1614	83	1	3	0	93	25.28	0.8	21	2	33.3	21.02	42.07	18.24	7.26
1615	59	1	3	1	67.5	25.59	1.71	41	0	29.5	21.48	51.34	21.88	7.47
1616	68	1	0	1	96	28.88	1.16	39	1	29.2	23.40	56.67	25.13	8.87
1617	64	2	0	0	65	19.70	2	24	0	25.1	12.01	35.92	14.82	6.08
1618	71	1	0	0	85	24.32	1.64	27	1	28.8	16.84	41.73	17.53	7.13
1619	72	1	0	1	98	26.92	1.35	31	0	27.6	21.46	56.26	26.77	8.88
1620	66	2	0	0	78	22.93	1.43	26	0	31.9	17.79	38.03	16.67	6.73
1621	62	1	1	0	87	21.44	1.19	37	1					
1622	65	2	0	0	81	24.42	1.39	15	1	40.6	20.75	30.35	12.23	5.53
1623	53	1	0	0	82	22.96	2.21	39	0	17.3	10.75	51.32	22.80	8.16
1624	78	2	0	0	47.5	19.62	1.25	7	1	19.2	8.45	35.59	13.33	5.72
1625	85	1	1	1	72.5	20.46	1.42	26	1	16.9	7.86	38.57	15.63	6.75
1626	79	1	1	0	77	21.87	1.15	40	1	15	8.41	47.73	20.71	7.93
1627	61	2	0	0	81	23.91	1.44	23	0	30.5	15.74	35.89	15.23	7.03
1628	72	1	3	0	83	20.87	1.13	20	2	18.2	8.95	40.09	15.36	5.75
1629	64	2	0	0	74	22.85	1.64	12	1	28.4	13.91	35.12	14.61	6.85
1630	77	2	1	1	91	24.73	0.69	15	2	33.8	17.67	34.61	12.83	5.95
1631	70	2	0	0	77	23.03	1.46	22	0	37.2	18.94	32.04	13.32	5.84
1632	52	1	1	1	93.5	26.56	1.81	38	0	29.3	24.84	59.89	26.22	7.93
1633	63	2	0	0	76	23.75	1.42	20	0	36.1	18.35	32.46	13.32	6.13
1634	53	2	0	0	67	20.71	1.78	20	0	30.9	13.03	29.12	11.62	5.6
1635	51	1	0	0	82	24.76	1.67	29	0	26.6	18.02	49.81	23.23	8.41
1636	59	2	0	1	84	22.82	1.49	21	0	34.2	19.38	37.32	16.25	6.55
1637	52	2	0	1	74.5	21.74	1.63	28	0	33.8	17.51	34.27	13.30	5.49
1638	69	1	3	0	99	28.72	0.66	28	1	29.6	23.50	55.82	23.25	8.76
1639	62	2	0	0	86	20.31	1.31	23	0	33.4	18.11	36.06	14.92	5.67
1640	65	2	0	0	110	34.52	0.91	24	1	41.5	35.79	50.52	18.29	7.21
1641	69	2	0	0	92.5	27.78	0.91	21	1	44.2	31.33	39.48	15.26	5.89
1642	53	1	0	0	102	32.37	1.39	38	0	38.8	35.29	55.55	23.65	8.14
1643	52	2	0	0	62	19.67	1.69	16	1	33.9	15.27	29.73	12.81	5.43
1644	55	2	0	0	69	23.73	1.19	19	0	36	19.13	33.95	13.64	6.01
1645	50	2	0	0	77	24.87	1.64	25	0	38.1	21.96	35.63	11.07	4.7
1646	74	1	3	0	92	25.58	1.2	34	1	27.9	20.33	52.66	22.53	7.64
1647	63	2	0	0	85	25.45	1.26	22	0	37.7	24.95	41.25	16.70	6.32
1648	65	2	0	0	86	26.97	1.48	30	0	40.9	26.48	38.25	16.34	6.65
1649	70	1	0	0	90	26.93	2.02	26.6	1					
1650	69	2	0	0	77	24.25	1.12	17	1	36.7	19.25	33.27	15.05	6.96
1651	54	2	0	0	73	24.46	1.51	20	0	42.9	22.33	29.73	12.37	5.79
1652	68	2	0	0	69.5	20.26	1.35	17	1	30.4	14.20	32.59	13.54	6.09
1653	72	1	1	1	90.5	25.70	1.36	31	0	36.3	22.50	39.48	17.77	7.18
1654	71	2	0	0	93	29.74	1.24	18	0	41.9	29.26	40.59	17.11	7.3
1655	69	2	0	0	87.5	30.03	1.27	17	1	43.3	29.15	38.20	15.33	6.74

1656	72	2	0	0	93	26.92	1.29	14	1	39.5	22.36	34.25	13.27	6.31
1657	55	2	0	0	69	20.34	1.57	16	1	29.5	11.77	28.16	11.99	6.2
1658	51	1	1	1	82	21.54	2.86	50	0	22	14.98	53.05	23.43	7.24
1659	52	2	0	0	72	21.31	1.45	18	0	39.7	20.62	31.32	12.33	4.96
1660	54	2	0	0	79	24.10	1.45	21	1	37.9	22.26	36.41	15.78	6.27
1661	65	1	3	1	90	25.50	1.71	30	0	26.3	18.26	51.26	23.57	8.43
1662	62	2	0	0	87	28.73	1.13	20	0	44.9	32.43	39.76	17.32	6.81
1663	51	2	0	0	83	24.48	1.4	12	1	36.5	21.74	37.81	16.62	6.8
1664	56	2	0	0	80	19.46	1.42	21	0	33.6	17.10	33.77	13.41	5.03
1665	74	1	1	1	91	26.27	1.25	33	0	26.4	16.82	47.00	20.29	8.02
1666	52	2	0	1	75	21.42	1.57	30	0	40.9	21.33	30.83	13.05	5.23
1667	59	1	1	0	70.5	18.17	1.86	38	0	18.7	9.42	40.94	17.83	6.3
1668	82	1	0	1	85	25.64	1.22	19	1	25.1	15.70	46.92	20.91	8.21
1669	58	1	0	0	76	23.43	1.96	36	0	28	16.98	43.68	18.89	7.24
1670	76	2	0	0	92	27.02	1.1	20	1	36.9	22.82	38.96	14.38	6.02
1671	60	2	0	0	85	22.07	1.64	24	0	36.9	20.99	35.87	15.57	6.01
1672	54	1	0	0	70	20.31	1.57	45	0	12.4	6.67	47.07	21.25	7.89
1673	56	2	0	1	75	23.23	1.5	26	0	34.3	17.55	33.68	13.41	6.07
1674	81	1	0	0	94	25.49	1.02	20	2	31.7	19.58	42.17	16.11	6.58
1675	60	2	0	0	84	26.59	1.74	20	0	42	29.38	40.52	18.35	6.88
1676	62	1	0	0	93	19.82	1.21	29	0	20.8	11.24	42.86	19.32	6.76
1677	68	1	0	0	75	19.29	1.07	29	1	23.8	11.19	35.74	14.88	5.78
1678	74	1	0	0	80	22.53	1.85	40	0	24	13.38	42.31	18.77	7.47
1679	64	2	0	0	70	22.32	1.03	14	1	37	18.20	30.98	13.00	6.06
1680	60	2	0	0	64	19.47	1.74	20	0	29.1	12.90	31.37	12.90	5.6
1681	79	1	0	0	79.5	22.62	1.5	32	0	24.1	13.39	42.13	18.99	7.59
1682	68	2	0	0	71	20.43	1.26	16	1	35.6	17.34	31.37	12.71	5.24
1683	73	1	1	1	78	20.32	1.69	25	1	19.7	10.00	40.74	17.62	6.78
1684	54	1	3	1	82	24.46	2.21	51	0	24.8	16.50	49.92	22.05	8.05
1685	52	2	0	0	82	22.54	1.56	24	0	35.4	21.03	38.33	17.29	6.39
1686	56	2	0	0	67	22.96	1.83	17	1	41.7	22.53	31.49	13.16	5.41
1687	58	2	0	0	65	20.16	1.2	20	1	33.6	14.07	27.78	11.60	5.5
1688	66	2	0	0	78.5	24.27	1.08	18	1	39	22.26	34.75	14.81	6.16
1689	79	2	0	0	74	20.09	1.46	19	0	25.5	10.15	29.63	11.31	5.37
1690	61	1	3	1	92	27.39	1.78	40	0	35.6	27.71	50.23	22.48	7.84
1691	50	2	0	0	70	22.29	1.73	20	0	37.8	17.59	28.96	12.19	5.62
1692	61	2	0	0	81	22.58	1.65	21	0	36.2	19.17	33.79	12.48	5.3
1693	71	1	3	1	89	26.09	1.59	29	1	18.4	11.18	49.47	19.28	8.14
1694	59	1	1	1	106	29.54	1.11	31	1	31.7	24.38	52.47	19.70	7.45
1695	62	1	0	0	75	22.03	1.57	37	0	19.1	10.86	46.06	20.24	7.61
1696	56	2	0	1	87	27.62	1.61	28	0	40.1	29.06	43.49	20.07	7.57
1697	58	1	1	0	91	24.34	1.64	33	1	33.4	24.74	49.34	20.96	6.48
1698	70	2	0	0	93	22.26	1.33	15	1	34	17.67	34.24	13.82	5.95
1699	74	1	0	1	90	24.89	1.82	26	1	28.9	20.22	49.69	21.75	7.56
1700	63	2	0	0	81	26.47	1.3	20	0	43.1	24.16	31.92	13.49	6.24
1701	69	2	0	0	104	30.49	1.13	16	1	45.4	37.16	44.72	18.51	6.77
1702	72	2	0	0	72.5	22.88	1.76	37	1	43	23.86	31.58	12.90	5.14
1703	82	2	0	0	83	21.70	0.28	11	2	23.8	10.62	33.96	12.41	5.77
1704	84	1	0	0	73	19.70	0.85	28	2	17.3	8.45	40.32	16.58	6.67
1705	60	1	1	1	74	21.03	1.33	19	1	20.7	8.92	34.25	14.74	6.81
1706	64	1	0	0	81	22.06	1.55	33	0	20.5	11.48	44.51	18.11	6.93
1707	85	1	3	0	93	24.12	1.02	26	1	31.5	18.42	40.13	16.12	6.38
1708	73	2	0	0	73	21.59	1.42	12	1	32.7	13.51	27.85	11.24	5.88
1709	69	2	3	1	68	19.26	1.59	22	1	34.1	15.33	29.57	11.94	5.02
1710	58	1	0	0	93	25.15	2.09	38	0	34.1	24.40	47.20	20.56	7.02
1711	51	2	0	0	85	28.75	2.11	24	1	40.1	29.15	43.51	17.55	6.96
1712	79	1	3	0	82	22.14	1.29	21	1	26.5	15.89	44.14	18.44	6.54
1713	76	1	1	1	91	24.05	1.21	26	1	30.8	19.84	44.60	18.79	6.87
1714	74	2	0	0	84	25.15	1.33	17	1	38.3	21.85	35.25	13.90	6.02
1715	70	2	0	0	102	29.76	1.75	21	0	45.6	32.84	39.18	14.60	5.99
1716	74	2	0	0	78.5	22.10	1.4	15	1					
1717	64	2	0	0	83	22.04	0.86	14	2	33.3	17.24	34.49	13.89	5.82
1718	52	2	0	0	80	24.28	1.76	27	0	38.9	21.43	33.64	13.98	5.96
1719	56	2	0	0	76.5	24.95	1.59	26	0	37.6	21.57	35.78	13.97	6
1720	60	1	3	1	111	32.61	1.47	35	0	38.4	33.41	53.67	20.74	7.59
1721	59	1	1	0	78	21.56	1.22	34	0	7.2	3.46	44.61	18.36	7.79
1722	78	1	3	1	92	24.32	1.04	30	1	23.1	17.39	57.93	22.93	7.55
1723	80	2	0	0	102	26.87	1.12	23	1	34.2	18.64	35.83	14.41	6.82
1724	73	2	0	0	75	21.38	1.18	20	0	31.4	15.27	33.38	12.82	5.36
1725	60	2	0	0	88	28.73	1.48	23	0	38.6	25.49	40.59	16.88	7.32
1726	70	2	0	0	93	28.57	1.36	28	0	43.2	27.70	36.49	16.23	6.94
1727	66	1	0	0	93.5	26.81	1.53	46	0	28.4	23.27	58.75	27.89	8.94

1728	61	2	0	0	86	25.20	1.02	18	1	36	19.75	35.10	14.38	6.46
1729	67	2	1	1	90	26.36	1.38	20	0	32.5	17.57	36.51	15.90	7.5
1730	75	2	0	0	89	25.62	1.2	24	0	40.4	23.16	34.12	14.86	6.51
1731	68	1	0	0	94	25.73	1.73	35	1	32.5	24.46	50.77	22.11	7.31
1732	75	2	0	0	110	30.91	1.21	20	1	44.4	31.92	39.93	14.81	6.22
1733	83	1	1	0	98	25.12	1.03	17	1	35.8	25.15	45.17	19.16	6.73
1734	53	2	0	0	93	31.96	1.73	19	0	42.6	28.87	38.95	16.45	7.52
1735	58	2	0	0	76	23.91	1.42	25	1	39	22.04	34.55	14.93	6.26
1736	61	1	1	0	96	25.19	1.55	41	0	26.3	18.22	50.96	22.24	7.88
1737	68	2	0	1	94	25.68	1.33	26	0	33.7	20.65	40.63	17.43	7.21
1738	69	1	0	0	79.5	21.70	2.02	36	1	18	10.92	49.64	19.28	6.61
1739	70	2	0	0	75	24.22	1.7	20	0	32.6	17.65	36.50	15.51	6.83
1740	71	1	3	1	69	18.95	1.23	35	0	14.8	7.49	43.29	19.26	6.87
1741	70	1	1	1	87	22.27	1.02	17	1	17.4	9.52	45.13	17.98	7.02
1742	64	2	0	1	81	21.91	0.91	20	1	21.6	11.01	39.92	16.18	6.83
1743	72	1	1	0	88.5	22.68	1.17	29	1	28.2	16.28	41.43	17.75	6.57
1744	65	2	1	0	90	26.22	0.99	21	1	35.9	18.94	33.81	13.59	6.64
1745	65	2	0	0	92	30.72	1.28	24	0	38.7	25.61	40.64	15.90	6.79
1746	57	2	0	0	86	25.51	1.43	26	0	37.8	24.11	39.70	17.41	6.8
1747	62	1	0	0	94	29.31	2.26	39.5	1	28.3	23.58	59.66	25.20	8.69
1748	68	2	0	0	83	24.62	1.37	21	1	36.6	21.13	36.58	15.05	6.31
1749	68	2	0	0	83	27.67	1.64	22	0	38.9	25.13	39.51	16.34	6.88
1750	74	2	0	0	89	23.74	0.84	11	2	42	22.41	30.94	12.69	5.48
1751	82	1	0	0	100	29.48	1.17	23	2	31.9	23.04	49.25	18.52	7.14
1752	60	2	0	0	86	21.91	1.57	22	0	35.2	18.19	33.49	13.79	5.62
1753	82	2	0	0	90	25.80	1.05	16	2	36	21.19	37.63	15.94	6.9
1754	78	2	0	1	100	23.06	0.92	4	1	28.5	17.07	42.76	18.80	7.19
1755	56	2	0	0	68	22.00	1.39	27	0	37.6	19.30	31.98	14.14	5.83
1756	51	2	0	0	82	22.92	1.56	26	1	38.4	21.55	34.50	14.10	5.7
1757	54	2	0	1	76	18.83	0.96	17	2	39.1	16.89	26.27	8.86	3.74
1758	67	1	0	1	94	26.55	1.15	18	2	32.1	22.44	47.47	22.00	8.18
1759	56	1	1	1	80	21.89	1.67	42	1	21.6	13.40	48.54	21.43	7.33
1760	58	2	0	0	78	24.58	1.88	22	0	38.9	22.05	34.57	13.86	6
1761	55	1	1	1	83	24.55	1.96	42	0	25	15.45	46.46	20.02	7.61
1762	62	2	0	0	85	24.30	1.82	22	0	30.3	16.48	37.84	14.88	6.66
1763	67	2	0	0	85	24.93	1.6	20	0	43.4	27.55	35.93	15.85	5.99
1764	55	1	0	1	89.5	29.43	1.42	30	1	35.3	23.24	42.67	18.68	8.19
1765	66	1	3	1	88	24.68	1.55	41	0	28	18.64	47.91	20.78	7.54
1766	78	1	0	1	88	22.54	1.47	28	1	27	15.66	42.35	18.22	6.96
1767	74	2	0	0	77	23.29	1.63	21	1	33.8	18.78	36.74	14.69	5.96
1768	75	1	0	0	83	26.47	1.67	31	0	29.2	16.52	40.11	17.08	6.85
1769	57	1	1	0	77	23.75	1.35	28	1	24.4	13.59	42.15	18.09	7.51
1770	73	1	0	0	82	21.86	1.74	36	0	23.4	13.41	43.97	19.81	7.05
1771	55	1	1	0	96	25.96	1.85	47.5	1	32.9	21.75	44.33	18.72	6.89
1772	75	1	0	0	79.5	20.06	1.31	28	1	25.8	12.10	34.84	13.87	5.89
1773	72	1	0	1	90	25.83	1.11	21	2	38.2	23.56	38.12	16.35	6.61
1774	65	2	0	0	81	24.65	1.3	16	2	41.6	22.02	30.93	11.79	5.38
1775	74	2	0	0	85	24.96	0.93	21	1	39.1	24.30	37.81	16.80	6.56
1776	61	1	3	1	83	23.66	1.5	30	1	21	12.28	46.34	20.17	8.05
1777	55	2	0	0	85	26.47	1.61	23	0	42.2	27.90	38.22	15.95	6.25
1778	59	1	1	1	91.5	23.99	1.3	41	1	29.1	18.86	45.90	19.93	7.05
1779	67	2	0	0	95	31.70	1.14	30	0	42.2	30.87	42.24	17.84	7.59
1780	81	2	0	0	97	27.62	0.52	11	2	44.4	27.10	33.88	12.93	5.9
1781	78	1	1	0	80	18.90	1.44	34	1	7.4	3.88	48.83	19.05	6.61
1782	52	2	0	0	83	26.02	0.07	12	2	42.8	23.86	31.92	12.44	6.02
1783	64	1	3	0	88	26.48	1.01	31	1	27.5	19.01	50.22	22.28	8.11
1784	50	2	0	0	61.5	19.35	1.74	23	1	30.2	14.83	34.29	14.84	5.64
1785	52	2	0	1	69	24.28	1.36	20	0	40.9	22.01	31.77	12.84	5.84
1786	54	2	0	1	70	19.76	1.09	22	1	31.6	15.81	34.22	13.38	5.17
1787	53	2	0	1	75	22.92	1.4	22	1	35.6	17.87	32.31	12.56	5.49
1788	61	2	0	0	97.5	29.71	1.17	15	1	42.2	29.56	40.42	16.45	6.62
1789	52	2	0	0	83	27.21	1.43	32	0	47.5	31.12	34.43	14.51	5.58
1790	59	2	0	0	79	25.71	1.5	17	1	41.6	26.76	37.54	16.87	6.67
1791	53	1	1	1	82	26.91	1.36	43	1	27.8	18.45	47.94	20.74	8.1
1792	60	2	0	0	74	22.33	1.49	25	1	39	21.38	33.47	13.70	5.38
1793	50	2	0	0	70	20.33	1.67	22	0	28.4	13.11	33.11	13.99	5.98
1794	59	2	0	1	96	32.13	1.22	23	0	48.1	36.67	39.56	15.01	6.11
1795	63	1	3	1	95	26.45	1.49	39	1	24.3	17.66	55.11	24.02	8.47
1796	55	1	1	1	82	25.89	1.4	42	1	23.5	17.47	56.91	26.04	8.91
1797	60	2	0	0	66	20.72	1.47	20	1	29.7	13.01	30.84	12.11	5.63
1798	50	2	0	0	71	23.10	1.46	29	1	38.3	21.66	34.86	15.20	5.98
1799	70	1	1	0	74	21.84	1.33	32	1	13	6.86	46.09	19.48	7.67

1800	56	2	0	0	81	23.14	1.37	24.5	0	37.6	22.84	37.86	15.25	5.78
1801	51	1	3	0	97	26.88	1.6	38	0	25	23.05	69.17	31.94	8.96
1802	53	2	0	0	93	29.69	1.36	17	1	49.7	37.05	37.43	16.82	6.55
1803	59	2	3	0	96	26.99	1.43	28	1	40.2	27.96	41.57	16.07	6.28
1804	51	2	3	1	83	26.12	1.57	25	0	42.4	29.17	39.58	17.20	6.42
1805	56	2	0	1	92	37.05	0.32	22	1	58.7	45.31	31.87	11.50	5.22
1806	53	2	0	0	76	24.86	1.85	30	1	35.3	21.45	39.31	17.42	6.83
1807	52	1	0	0	96	26.60	1.78	45	1	28.8	23.31	57.60	26.73	8.47
1808	50	2	0	0	75	23.93	1.83	24	1	40.6	22.92	33.51	14.41	5.88
1809	60	1	3	1	91	21.99	1.97	39	0	27	20.75	56.22	25.28	8.15
1810	56	2	0	0	64.5	20.82	1.46	20	0	36.2	16.54	29.16	11.50	5.14
1811	53	2	0	0	99	29.50	1.6	18	0	38.8	28.60	45.10	18.11	7.06
1812	57	2	0	0	77	22.23	1.37	25	0	36.2	18.88	33.20	13.14	5.51
1813	63	2	0	0	75	21.92	1.88	22	0	32.6	19.02	39.38	16.92	6.22
1814	75	2	0	0	84	25.84	1.22	20	0	40.4	24.32	35.89	15.25	6.51
1815	53	1	1	0	74	22.39	2.35	39	1	17.4	10.10	48.04	21.51	8.17
1816	52	2	0	0	65.5	20.32	1.79	21	0	34.1	17.20	33.17	14.05	5.54
1817	50	1	0	0	81	24.48	1.9	41	0	20.1	13.25	52.75	24.10	8.94
1818	50	2	0	0	87	29.15	1.81	37	0	40.4	29.42	43.42	18.93	8.3
1819	66	2	0	0	79	24.21	1.44	18	0	36	19.90	35.37	13.07	5.73
1820	66	2	0	0	76	23.94	1.61	24	0	36.8	20.51	35.21	13.88	5.83
1821	52	1	0	1	80	21.93	2.06	41	0	15.7	10.31	55.27	24.43	7.96
1822	58	1	0	1	80	20.42	2.27	39	0	20.3	12.34	48.30	21.34	6.98
1823	52	1	0	1	81	23.41	1.76	48	0	23.2	14.82	49.13	22.28	7.99
1824	51	1	0	1	98	32.24	1.46	49	1	38.2	35.19	57.03	24.07	7.96
1825	57	1	0	1	86	25.90	1.7	37	1	30	21.12	49.26	21.81	7.66
1826	81	1	3	0	100	27.20	1.32	26	2	37.6	30.48	50.53	22.36	7.3
1827	83	2	0	0	106	33.80	0.46	12	2	53.9	41.12	35.23	15.01	6.5
1828	55	2	0	0	65	21.48	1.55	23.5	0	36.1	21.10	37.38	16.37	6.04
1829	60	2	0	0	65	18.26	1.26	20	1	30.6	13.81	31.39	11.78	4.78
1830	63	1	3	1	94	26.34	1.44	43	1	28.8	21.12	52.20	23.97	8.36
1831	54	2	0	0	69	20.84	1.49	25	1	38.8	22.17	34.90	15.78	5.65
1832	59	2	0	0	79	26.42	1.29	30	0	44.8	27.09	33.40	14.69	6.25
1833	74	1	0	0	89	23.96	1.55	38	0	26.4	18.43	51.37	23.54	7.97
1834	70	2	0	0	68	18.21	1.54	20	0	26.4	12.72	35.52	14.04	5.24
1835	60	1	1	0	95	27.74	1.24	31	1	34.1	27.14	52.50	20.69	6.87
1836	62	2	0	0	67	17.95	1.16	9	1	30.1	12.75	29.66	11.35	4.76
1837	54	1	1	1	84	26.55	1.17	35	1	26	20.05	57.09	25.74	8.75
1838	59	1	0	0	95	27.55	1.81	36	1	31.5	25.09	54.60	23.84	8.06
1839	59	2	0	0	76	21.89	1.44	25	1	36	21.13	37.58	14.95	5.49

BMDspin	BMDhip	SMAF	MMSE	CESD	MNA	CCI	TLC	ALB	TC	A1c	hsCRP	VitD3	iPTH
1.01	0.79	0	29	2	28.5	2	1742.5	4.4	158	8.9	0.385	16.2	56
0.94	0.83	0	29	10	24	0	1422.4	4.6	175	5.8	0.055	11.7	28.6
0.88	0.54	0	20	8	27	0	2008.5	4.4	173	6.4	0.901	27.2	48
1.31	1.08	0	24	4	25.5	3	1866.5	3.4	239	6.3	0.06	17.9	25.4
0.82	0.84	0	26	5	23	0	2290.6	4.5	233	6.2	0.289	18.8	50
1.04	1.01	0	27	1	28	0	1888.5	4.7	244	5.7	<0.012	25.7	23.1
1.08	0.52	0	29	16	19	3	1178	4.4	176	6.2	0.406	24.2	43.9
0.96	0.76	0	24	1	29	0	1021.2	4.7	242	5.3	<0.012	22.5	30.2
1.27	0.98	0	28	1	27.5	2	2252.9	4.1	307	5.6	0.264	21.3	36.2
	0.78	0	23	2	26.5	3	1430.6	4.7	181	5.1	0.05	24.2	19.5
1.18	0.97	0	27	8	29	0	1787.7	4.6	218	5.8	0.192	27.5	28.5
1.09	0.94	0	19	7	28	0	1080	4.5	215	5.2	<0.012	18.9	34.6
0.79	0.70	0	28	0	27.5	0	2256.5	4.3	243	5.7	0.064	23.7	35.9
1.26	0.99	0	29	0	28	0	1938.4	4.5	175	5.4	0.132	20.5	53.6
1.21	0.85	0	27	0	28	2	2566.6	4.4	175	6	0.033	33.1	37.8
1.00	0.84	0	24	0	24	0	1561.3	4.6	235	5.6	0.037	20.9	30.4
1.30	0.80	0	21	4	28.5	4	1080.6	4.1	179	6	0.331	26.9	54.5
0.98	0.76	0	22	10	26.5	0	2077.3	4.8	210	5.6	<0.012	21.6	30.5
1.00	0.67	0	25	11	25	3	1849.1	4.2	142	9.1	0.195	31.6	35.4
1.09	0.79	0	25	8	27.5	2	1401.4	4.3	161	6	<0.012	28.7	99.7
0.99	0.82	0	18	16	26	2	970.08	4.3	187	5.3	0.015	36.1	47.8
0.70	0.63	0	16	5	27.5	4	2800	4.4	131	8.3	0.874	15.9	61.3
0.82	0.61	-2	24	33	21.5	1	1826.8	4.2	193	5.7	0.093	18.8	60.2
1.00	0.81	0	29	1	28.5	0	2088	4.1	135	5.3	0.094	38.3	21.6
1.12	0.67	0	20	20	24.5	3	2020.3	4.3	182	8.4	0.481	31	12.1
0.79	0.53	-14	5	16	24	6	1200	4.2	149	6.6	0.044	10.3	51.1
1.04	0.57	-4	24	2	23.5	3	1020.8	4.4	155	5.5	<0.012	23.1	64.2
0.89	0.71	-1	21	11	25	2	1290.6	4.5	201	6.4	0.478	13.2	83.7
0.82	0.65	0	28	12	26	4	2392.8	4.4	196	7.8	0.221	22.5	47.4
1.45	0.84	-35.5	13	27	23	4	1370	4.2	148	6.9	0.492	20.8	55.3
0.84	0.79	0	27	11	25	0	1622.1	4.1	148	6.7	0.407	18.1	75
0.79	0.69	0	25	10	27.5	2	2060.5	4.4	267	6.9	0.086	24.9	34
1.36	0.98	0	30	0	27	0	1621.8	4.5	186	5.2	<0.012	26.6	32.9
0.79	0.63	-3	21	5	27.5	0	1708.3	4.4	165	5.3	0.123	18.8	115.4
1.08	0.79	0	29	1	29.5	0	1449.2	4.5	187	5.4	0.017	30.6	27.4
0.87	0.57	-9	16	27	22	3	1771.2	4.2	116	6.3	0.055	32.5	31.8
0.85	0.73	0	26	0	24.5	2	1681.7	4.2	209	12.8	0.056	19	44.8
1.11	1.05	0	28	3	28	2	2369	4.3	215	9.8	0.04	21.7	47.2
0.89	0.46	-10	25	9	26	4	1009.1	4.1	179	6.2	0.036	18.9	62.4
1.15	0.80	0	27	14	29	1	1260.4	4.2	173	6.7	0.033	30	43.1
1.08	0.80	-5	26	12	24	3	982.08	4.6	171	5.5	<0.012	34.7	107
0.96	0.80	0	29	10	26.5	1	1771.8	4.4	194	5.9	0.447	14.8	44.5
1.03	1.09	0	24	18	26.5	4	1924	4.5	244	7	0.042	14.3	72.8
0.95	0.72	0	28	4	24.5	1	1790.5	4.2	165	6.6	0.022	25.9	29.7
0.93	0.83	0	29	14	27	2	1268.3	4.8	188	5.4	0.031	27.9	36
0.89	0.67	0	23	6	25.5	1	2281.7	4.4	175	6.3	0.14	23.4	48.7
0.89	0.73	0	30	3	28	1	2109.8	4.2	175	5.8	0.034	23.4	73
1.30	0.75	-7	19	20	23.5	3	1950.4	4.8	186	7	0.356	18.1	42.4
1.22	0.89	0	30	7	27.5	0	2407.2	4.6	171	5.2	0.088	30.3	37.6
0.92	0.61	0	18	23	23.5	5	1548.1	4.2	177	6.4	0.436	37.1	49.8
0.84	0.82	0	28	0	25.5	1	2142.9	4.1	151	7.6	0.119	33.3	45.4
0.90	0.67	0	25	3	20.5	0	1169.4	4.7	166	4.5	0.026	37.1	40.4
1.05	0.77	-5	15	12	26.5	3	780.88	4.3	169	5.3	<0.012	28.6	40.8
1.12	0.86	0	27	0	28	2	1898.6	4.6	190	6.7	0.061	43.2	32.3
0.90	0.78	0	20	0	24.5	1	2138.9	4.4	192	7.1	0.452	28.3	72.7
	0.75	0	22	0	25.5	3	1749.2	4.4	193	5.6	0.043	29.5	26
0.76	0.64	0	27	9	26.5	4	1407.3	4.4	199	6.2	0.036	33.2	32.4
1.17	0.72	-8	16	3	28	3	2322.3	4.4	241	8.2	0.403	20.2	29
0.75	0.58	0	25	4	25	0	1691.9	4.2	205	5.6	0.034	27.1	12.8
0.75	0.73	0	20	0	28.5	4	2616.3	4.3	191	6	0.042	24.2	32.1
0.81	0.70	0	29	12	26	1	1848	4.3	215	5.9	0.055	28.6	57.6
1.17	0.84	0	25	6	22.5	3	1961.4	3.9	149	6.6	0.144	73.1	33.6
0.92	0.81	0	30	1	25.5	1	1280	4.6	167	5.3	0.094	42.4	54.7
1.04	0.71	0	28	37	25	3	1801.4	4	187	5.5	<0.012	19	49.8
1.43	0.78	0	27	2	27.5	2	2868.8	4.4	192	5.7	0.207	14.9	81.7
0.91	0.74	0	12	39	17.5	0	2407.9	4.5	214	5.9	<0.012	17.9	54.1
0.87	0.69	0	25	1	27	1	2257.6	3.7	99	5.5	0.853	31	26.1
0.72	0.74	0	19	7	20	2	2209	4	179	5.9	0.018	20.2	22.4
0.84	0.64	0	28	1	24.5	1	2824.7	4.2	193	5.8	0.024	18.1	38.2
0.76	0.71	0	24	4	27	0	1801.2	4.3	190	5.9	0.111	22.1	39.4
1.04	0.76	-1	12	6	26	0	2578.5	4.7	221	5.9	0.066	16.5	47

0.93	0.81	0	21	3	27.5	0	2351.4	4.5	160	6	0.263	15.7	18.4
1.25	1.08	0	28	8	27.5	0	1982	4.4	254	5.8	0.175	23.4	45.6
1.13	0.81	0	17	1	21.5	2	2171.6	4.4	156	6	0.079	39.9	44.5
		0	17	4	28	2	2588	4.9	202	5.6	0.191	37.1	26
1.11	0.82	0	24	7	26	0	1948.8	4.3	197	5.7	0.628	22.2	26.1
0.93	0.86	0	28	0	27.5	3	1739.2	4.4	216	5.8	0.041	26.2	46.6
0.95	0.79	0	30	8	29	0	2681.6	4.5	232	6	<0.012	42.5	13.9
1.37	0.92	0	23	0	29	3	1389	4.2	136	6.6	0.065	41.9	54.8
0.74	0.58	0	19	13	18	3	1800.2	4.4	262	6.1	0.035	38.6	33
1.12	0.80	0	28	0	26.5	0	1910.4	4.3	179	5.7	0.169	45.9	35.2
0.85	0.69	0	27	3	26	0	1719.6	3.9	182	5.8	0.036	31.8	64
0.92	0.74	0	25	2	20.5	1	1351.4	4	198	5.8	0.13	21.9	34.3
1.27	1.16	0	26	2	30	0	1469.1	4.6	233	5.6	0.015	17.7	24.5
1.13	0.98	0	30	1	25	1	1802.3	4.6	131	5.8	<0.012	24.2	71.5
0.98	0.85	0	28	3	28.5	0	2547.8	4.8	172	6.4	0.028	35.7	28.5
1.19	1.00	0	28	3	28.5	0	1961.8	4	192	5.8	0.029	67	29.3
1.05	0.78	0	28	2	26.5	2	1278.4	4.2	188	8	0.018	44.4	36.6
0.99	0.81	0	27	0	26	0	1387.1	4.5	193	6	0.034	31.8	43.7
1.34	1.09	0	28	0	28	1	2511.3	4.3	183	6.5	0.16	35.3	24.4
1.13	0.63	0	29	0	26.5	0	1989	4.3	221	5.8	0.733	26.7	27.9
0.88	1.01	0	27	1	29	3	2050.1	4.6	217	6.4	0.152	38.2	21.8
0.97	0.69	0	22	0	26.5	0	1590.2	3.9	130	5.8	0.021	34.1	47.4
1.16	1.09	0	27	2	28.5	1	2419.1	4.3	148	5.8	0.023	31.8	30.5
0.92	0.71	0	26	17	26.5	2	3578.3	4.1	166	5.6	2.175	50.2	38.5
0.71	0.70	0	28	1	25.5	0	2777.2	4.5	169	5.7	0.015	18.5	96.8
1.30	1.00	0	28	0	28	0	1140.4	4.4	164	5.8	<0.012	16	24.2
0.97	0.70	0	24	1	28.5	0	1997.2	4.6	265	6	0.067	22.1	48.3
1.10	0.85	0	28	4	27	0	2147.5	4.3	187	6	0.069	18.4	49.2
0.91	0.78	0	24	2	27.5	1	1309.7	4.1	215	6.1	0.046	24.2	79.5
1.07	0.88	0	20	6	25	2	2022.1	4.8	268	6.4	0.056	16.9	36.6
0.75	0.65	0	22	5	26.5	5	2642	4.4	201	9.1	0.217	24.7	28.4
0.92	0.76	0	29	0	29.5	1	1678.4	5	237	5.8	0.03	20.6	42.2
1.09	0.81	0	28	20	23	4	2886.8	4.7	189	7	1.056	13.5	27.2
1.09	0.76	0	24	2	25.5	1	1740.5	4.7	226	6	<0.012	34.8	35.2
0.75	0.71	0	24	15	27	2	1781.6	4.3	216	5.9	0.022	17.8	37.6
1.34	1.16	0	25	2	22.5	1	2581	4.4	185	8.2	0.042	25.2	23.2
0.97	0.90	0	27	0	29	3	2321.4	4.4	193	5.9	0.028	39	33.1
0.97	0.91	0	25	0	27	0	2219.4	4.6	201	5.5	0.145	36.9	34.6
1.02	0.81	0	24	0	27.5	3	2200.4	4	146	5.9	0.543	31.7	64.3
1.04	0.74	0	23	28	21	2	3258.9	4.6	208	7.1	0.11	17.5	29.3
1.48	1.00	0	28	1	26	1	2261.3	4.1	186	5.7	0.062	31.7	13
0.95	0.82	0	26	5	28.5	1	1979.6	4.1	186	6.4	0.107	26.6	46.9
1.09	0.74	0	23	2	27	2	1328.4	4.3	149	5.4	0.456	32.9	20
0.89	0.71	0	28	4	28	0	1978.8	4.5	215	5.7	0.367	18.3	56.7
1.01	0.86	0	30	2	27.5	0	1717.8	4.3	257	5.6	0.237	17.9	24.5
0.97	0.71	0	26	0	24.5	0	1328	4.4	232	5.3	<0.012	24.8	27.6
1.14	0.77	0	22	4	25.5	1	2326.3	4.2	170	8.2	0.068	26	31.9
1.18	0.94	0	28	13	28	2	1521.6	4.4	135	5.9	0.015	42.1	44
1.05	0.91	0	28	0	29.5	1	1909.4	4.2	207	5.3	0.044	20.7	24.5
1.38	0.91	0	27	1	27.5	2	2157.6	4.8	192	5.6	0.191	29	24
0.99	0.81	0	29	2	24.5	0	1519.2	4.2	199	4.8	0.049	30.1	26.1
1.10	0.80	0	27	5	25.5	1	3450.3	4.6	222	5.6	0.346	38.7	26.2
1.10	1.00	0	27	0	25	2	2920.2	4.2	211	6.3	0.413	31.8	27.6
1.29	0.98	0	29	0	26.5	0	1981.1	3.6	173	5.3	0.855	33.4	71.2
0.88	0.66	0	29	0	29.5	0	4268.9	4.7	248	6.1	0.098	11.7	39.4
1.19	0.92	0	29	0	28	0	1711.7	4.6	212	5.6	0.057	34.6	26.4
1.29	0.91	0	24	0	28	0	2470.4	4.4	170	6.9	0.173	22.6	43.2
1.00	0.88	0	25	4	28.5	1	2049.4	4.3	195	6.8	0.021	26.8	37.4
1.09	0.80	0	30	2	29	0	2298.9	4.5	187	5.9	<0.012	39.1	32.1
1.09	0.99	0	25	4	27	0	2108.1	4.7	229	7.2	0.632	20.1	41.1
0.91	0.85	-1	30	0	28	1	1467.8	4.6	149	6.1	0.015	42.1	50
1.02	0.80	0	30	0	27.5	0	1987.4	4.5	172	5.7	0.318	40	32.5
1.03	0.85	0	29	0	28	0	2061.9	4.4	176	5.7	0.041	17.4	49.3
1.19	0.95	0	30	0	27.5	0	1767.1	4.5	153	5.9	0.404	38.3	49.5
0.87	0.61	0	26	6	24	2	1751.4	4.2	155	6.2	0.031	21.5	47.4
0.78	0.67	0	26	2	26	0	2368.8	4.6	161	6	0.389	27.4	34.5
0.82	0.74	-1	24	6	27	3	1578.1	4.1	196	5.9	0.05	23.3	41.7
1.17	0.91	0	29	0	27.5	0	1997	4.5	186	6.2	0.034	29.9	40.9
1.39	1.02	0	28	0	29.5	0	2311	4.4	174	5.1	0.081	21.9	34.4
1.19	0.97	0	26	1	26.5	3	3152.9	4.3	184	6	0.09	33.4	50.1
0.92	0.90	0	30	0	29.5	0	1551.1	4.3	205	5.6	0.014	32.3	16.9
0.80	0.68	0	16	3	25	4	1621.9	4.6	173	5.8	0.111	28.5	64.6

1.13	1.08	0	29	1	28.5	0	1269.4	4.8	242	5.7	0.014	38.9	29.7
0.90	0.78	0	23	0	26	1	2592.1	4.3	241	6.4	0.172	16.9	37.6
1.12	0.87	0	25	2	29.5	0	2801.4	4.7	247	6.5	0.133	14.8	23.8
1.03	0.84	0	25	42	24	0	3208.8	4.2	197	5.8	0.136	21.7	16.6
1.04	0.77	0	20	1	28	1	1768.6	4.6	192	5.5	0.039	31.8	29.1
0.96	0.68	0	26	0	29	0	2858.4	4.6	211	6	0.096	26.1	35.2
0.97	0.78	0	27	0	29	1	3198.5	4.7	258	6.4	1.335	18.3	55.5
		0	29	0	28.5	2	1407.3	5.2	229	7.8	0.129	24.8	29.6
0.98	0.78	0	30	4	26.5	1	1909.2	4.6	199	5.6	0.344	32.4	36
0.98	0.72	0	30	6	28	0	1667.2	4.6	248	6.2	0.176	33.9	55.2
0.92	0.74	0	22	15	26.5	4	2593.5	5	129	6.9	0.443	23.6	33.9
0.66	0.65	0	23	2	21.5	4	1871.5	4	164	5.7	0.069	25.4	51.9
1.18	1.05	0	30	1	29.5	0	1599.3	4.5	191	5.6	0.019	30.6	34
1.22	0.99	0	29	18	25.5	4	2078.6	4.5	178	6.5	<0.012	29.9	15.2
1.08	0.85	0	24	0	28.5	0	1840	4.4	169	5.5	0.268	26.6	68.3
1.10	0.81	0	30	0	27.5	3	1522.8	4.4	233	5.7	0.109	17.2	42.5
1.21	0.96	0	25	0	28.5	1	1271.7	4.6	159	7	0.039	19.7	61.2
0.92	0.80	0	30	1	29.5	0	1609.9	4.4	238	6.1	0.035	23.4	40.4
0.79	0.77	0	29	7	28	2	3113	4.7	176	6.4	0.12	22.5	51.8
0.87	0.62	0	26	3	25	1	2390.4	4.8	235	6.1	0.211	25	33.2
1.01	0.78	0	27	5	28.5	0	2089.5	4.7	237	5.8	0.043	23.1	10.1
1.16	0.67	0	29	13	28	2	2160	4.8	208	7.1	0.321	25.4	14.9
0.97	0.97	0	26	7	28.5	0	3041.5	4.5	220	6	0.075	27.4	46
1.10	0.91	0	30	0	28	0	1210.3	4	148	5.6	<0.012	21	71.8
1.47	1.13	0	29	8	28	3	1039.9	4.2	205	6	1.088	31.6	74.9
0.93	0.73	0	24	0	28.5	1	1940.5	4.2	232	5.9	0.724	20.2	35.3
1.46	0.96	0	23	9	25.5	3	1598.3	4.3	182	6.8	0.131	26.4	21.3
0.91	0.60	-3	17	4	27.5	2	1506.8	4.3	225	5.6	0.066	22.1	56.7
0.93	0.70	0	28	1	23.5	2	1642	4.5	240	5.7	<0.012	29.9	24.5
0.83	0.64	0	23	0	28	0	2399.4	4.4	246	5.8	0.168	14.8	47.9
1.17	0.74	0	29	0	29	1	2213.7	4.6	183	7.2	0.057	18.9	79
	0.78	0	27	17	26	0	1070.7	4.2	249	6	1.575	21.5	28.1
1.07	0.89	0	21	19	25.5	0	2022.5	4.7	208	6.3	0.252	29.7	35.2
0.85	0.70	0	30	7	28.5	0	2127.4	4.4	196	6	0.026	32.2	22.3
0.85	0.89	0	30	11	26.5	2	1972.8	4.3	176	6.2	0.735	21	38.8
1.14	1.15	0	29	6	23.5	1	5059.8	5	195	16.1	0.156	24.5	14.9
1.13	0.95	0	27	6	26	1	1382	3.8	131	6	0.104	44.1	41
1.11	1.05	0	29	0	30	0	1999.8	4.2	208	5.6	0.022	25	38.3
1.09	0.78	0	29	0	28.5	1	1978	4.6	134	7.5	0.07	26.5	46.9
1.13	0.89	0	25	8	26	1	2711.7	4.8	174	7.9	0.03	28	22.6
0.91	0.63	0	14	5	26	3	1141.1	4.6	168	5.7	0.075	35.9	36.3
1.10	0.87	0	27	6	28.5	1	1739.9	4.5	168	6	0.024	27	39.6
0.83	0.89	0	17	31	25	2	1769.8	4	169	6.2	0.05	28.8	47.6
1.10	0.92	0	27	0	28.5	0	2620	4.7	258	6.3	0.434	25.4	30.3
1.07	0.84	0	28	0	28.5	0	2130.4	4.3	157	5.6	0.101	22.5	22.7
0.97	0.85	0	26	0	28	0	2118.2	4.3	211	5.3	0.05	38.9	43.4
1.05	0.77	0	29	2	27.5	0	1149.9	4.6	179	5.8	<0.012	21.3	21.5
1.15	0.79	-1	26	0	28.5	1	1498.6	4.3	193	6.6	0.1	43.8	35.7
1.03	0.91	0	26	3	26.5	1	1168.5	4.3	165	5.9	0.034	35.6	38.8
0.76	0.60	0	16	4	21	1	2182.3	4.5	158	7.3	1.038	34.6	57.6
0.92	0.95	0	30	5	28.5	0	2019.3	4.2	152	5.8	0.013	31.2	14.5
1.29	0.84	0	24	3	28	0	2812	4.6	196	6.4	0.155	25.4	70.3
1.03	0.73	0	30	0	28.5	1	2779.9	4.5	255	6.1	0.063	19.5	58.7
1.16	0.83	0	30	14	27.5	0	1306.9	4.4	151	5.4	0.421	29.8	27.2
0.90	0.64	0	22	6	28.5	0	1542.3	4.5	190	5.7	0.534	22.9	41.4
0.73	0.69	0	23	3	26.5	0	2212	4.5	223	5.8	0.029	20	33.5
0.78	0.83	0	28	8	26.5	0	1162.2	4.7	201	5.3	0.096	28.3	32.7
0.73	0.53	0	24	6	29.5	0	1429.1	4.6	206	5.9	0.017	34.6	33.5
0.92	0.64	0	28	0	28	0	2621.4	4.3	228	5.4	0.024	15.1	98.3
1.27	1.03	0	26	11	26.5	1	2553.2	4.8	207	6.8	0.249	26.2	71.2
1.17	0.96	0	28	14	25.5	1	1551.6	4.8	217	5.4	<0.012	37.4	15.6
1.00	1.01	0	30	0	27.5	0	1818.2	4.4	177	6.3	0.089	25.4	51.2
1.37	0.94	0	30	1	24.5	3	1441.4	5	140	9.1	0.052	33.2	26
1.25	0.99	0	30	0	29.5	2	2753.6	4.4	231	8.9	0.22	19.4	28
0.89	0.89	0	26	2	29	0	1448.9	4	169	5.7	0.09	30.3	39.8
1.15	0.90	0	30	4	28.5	0	2292.8	4.7	222	6	0.497	12.8	52.8
1.34	1.14	0	26	24	28	1	2272.7	5.1	248	12.6	0.262	7.4	17.2
1.10	0.82	0	18	5	27	0	1622.4	4.5	161	6.1	0.138	36.4	28.8
1.14	0.89	0	29	5	27.5	0	1241.5	4.3	216	5.8	0.036	19.9	46.2
1.10	0.84	0	29	1	28.5	0	2889.6	4.6	234	5.4	0.146	14.1	17.3
0.94	1.03	0	28	3	26	1	1983.4	4.8	155	8	0.707	20.1	34.9
1.19	0.89	0	24	0	26.5	5	1652	4.7	192	6.7	<0.012	25	58.9

1.27	0.69	0	28	2	27	1	2857.1	4.5	207	6.5	0.09	19.3	38.8
1.03	0.89	0	30	3	27	0	1517.3	4.5	166	5.7	<0.012	24.1	27.1
0.77	0.87	0	28	1	28	0	1842.5	4.3	265	5.6	0.016	29.4	48.2
0.79	0.69	0	20	1	27.5	0	1992.7	4.6	211	5.7	<0.012	25.7	31.5
		0	29	1	27	1	2218.4	4.5	179	7.1	0.269	19.4	70
0.70	0.70	0	21	6	27	8	979.17	3.7	157	5	0.241	21.9	32.7
1.06	0.89	0	29	1	29	1	2121.6	4.3	177	5.7	0.186	22.5	40.3
1.14	0.75	0	29	44	28.5	0	2882.8	4.6	240	6.1	0.411	18.7	39
1.25	0.72	0	26	4	27	0	1330.6	4.2	190	6.1	0.247	26.9	66
0.97	0.85	0	27	1	29	0	1500.9	4.7	185	6.2	0.047	19.9	22.7
0.70	0.57	0	24	21	27	0	1381.6	4.6	220	5.8	0.056	10.1	34.1
1.16	0.80	0	16	6	28.5	1	2051.3	4.5	172	8.2	1.162	21.6	18.2
1.15	0.81	0	29	2	28.5	1	1978	4.5	142	6.5	<0.012	39.2	50.8
1.06	0.94	0	29	4	28.5	1	2831.4	4.4	207	9	0.728	14.5	45.1
0.71	0.64	0	25	1	29	0	1438.1	4.3	222	5.4	0.042	28.8	56.6
1.03	0.83	0	30	2	29.5	1	2497.9	4.6	247	12	0.704	15.5	24
0.94	0.65	-2	20	2	25	4	2852.9	4.3	128	5.7	0.029	28.4	30.5
1.06	0.67	0	27	0	29	0	2542	4.3	187	5.6	0.201	25.3	34.2
		0	19	2	23.5	2	2309.6	4.4	289	6.2	0.195	21.5	22.4
1.00	0.75	0	27	3	29	0	2530.4	4.6	236	6.7	0.016	25.4	46.5
1.06	0.96	0	29	0	30	2	3467	4.6	244	5.1	0.281	25.8	53.6
1.17	1.16	0	30	0	29.5	0	1058.6	4.6	189	6.1	0.06	21.5	30.4
1.31	1.05	0	28	0	29	0	2100.8	4.5	209	5.8	0.089	27.5	34.9
0.98	0.95	-1	26	13	24	1	1909.5	4.4	152	5.4	<0.012	22.4	42.8
0.96	0.76	0	28	0	29	0	1841.9	4.5	213	5.3	<0.012	28	42.8
1.30	0.93	0	25	9	24	5	2299.4	4.6	110	7.3	0.161	41.6	52
0.91	0.92	0	30	4	29	1	2051.6	4.4	176	8	0.26	25.2	44
1.13	0.98	0	29	2	27	0	2087	4.4	157	5.8	0.124	20.6	20.2
0.84	0.73	0	23	4	28	0	1670.6	4.7	205	5.6	0.031	19.8	37.5
0.98	1.03	0	26	2	27.5	1	3079.9	4.6	215	5.8	0.027	24	45.1
0.89	0.78	0	24	2	27.5	1	1471.1	4.4	143	5.2	<0.012	16.1	55.8
1.29	0.96	0	29	4	25.5	3	2891.4	4.6	180	6.5	0.196	26.2	23.8
0.91	0.85	0	29	6	29.5	0	1789.6	4.2	207	5.5	0.037	24.4	28.9
		0	22	2	28.5	0	2537.1	4.2	227	5.7	0.018	19.9	39.3
		0	26	8	27	0	1960.2	4.5	141	5.8	0.166	18.8	50.7
1.01	0.94	0	28	3	30	1	940	4.5	218	5.4	0.042	26.2	47.2
0.97	0.76	0	29	0	29	0	1531.7	4.4	218	5.6	0.192	32.9	25.4
		0	30	1	28	0	1901.8	4.7	156	5.9	0.014	21.7	92.3
0.81	0.56	0	24	1	27.5	2	1906.8	4.3	196	6.1	0.58	19.4	46.1
1.71	1.02	-5	30	15	25.5	7	1939.7	4.1	150	8.2	0.109	29.1	53.7
1.34	1.08	0	28	2	27.5	1	1622.3	4.7	186	6.6	0.176	28.5	41.7
1.18	1.15	0	28	5	28	0	1899.8	4.7	202	5.4	0.031	23.6	34.5
0.78	0.62	0	24	4	18	1	220.66	4.1	182	7.3	0.475	22.4	48.3
0.76	0.76	0	20	2	27.5	2	2390.3	4.4	160	6.5	0.036	26.4	45.1
1.06	0.74	0	29	1	26.5	4	1907.7	4.6	182	5.5	<0.012	24.5	39
1.18	0.68	0	29	19	28	0	1947.7	4.7	179	5.2	0.205	22.1	40.3
1.27	0.93	0	30	0	27	0	3148.2	4.6	179	6.4	0.106	15.1	26
	0.66	0	20	0	29.5	0	2950.7	4.7	175	5.5	0.395	26.7	55.2
1.04	0.78	0	26	2	27	1	1971.1	4.6	187	7.4	0.039	18.8	33.5
0.99	0.89	0	29	0	27.5	0	1641.9	4.6	214	5.7	0.048	25.1	29
1.08	0.80	0	28	8	27	4	1287.8	4.5	149	5.2	0.173	24.1	33.1
0.96	0.71	0	21	3	26.5	3	3933.5	4.5	215	13.3	0.131	27.3	39.6
1.11	0.96	0	27	2	26	2	1562.2	4.4	171	5.9	<0.012	39.5	27
1.20	0.82	-1	30	0	29	7	1401.7	4.6	188	6.5	0.033	29.7	36.4
		0	23	4	28	0	1698	4.8	179	5.3	0.019	32	35.1
1.11	0.91	0	28	0	28.5	0	1179	4.5	139	5.3	0.017	28.1	35.6
1.20	0.93	0	28	0	28.5	1	1511.6	4.5	155	5.4	0.065	28.7	34.6
1.19	0.95	0	25	5	27.5	0	1639.4	4.4	209	5.8	0.066	23.3	33.9
0.83	0.70	0	22	1	30	0	1680.3	4.2	287	5.6	0.055	24.1	46.7
1.06	0.80	0	23	1	27	1	1771.6	4.5	164	6.6	0.015	24.8	32.9
0.81	0.76	0	29	1	28	0	2530	4.5	211	6	0.232	18.2	79.4
		0	16	26	23	0	1421.3	4.3	217	6	0.044	30.8	36.5
1.22	0.95	0	28	1	28.5	0	2070.3	4.6	267	5.2	0.046	30.4	46.1
	0.60	-0.5	24	1	27	0	1300	4.4	186	5.8	0.02	24.3	41.2
1.24	0.84	0	29	3	27.5	1	2090.7	4.6	186	5.9	0.333	26.2	34.2
0.89	0.91	0	28	1	25.5	0	2122.3	4.5	190	4.8	0.325	35.4	24.7
0.93	0.65	-2.5	25	9	26.5	0	2341.9	4.5	197	6.2	0.042	19.9	60.6
1.15	1.04	0	24	0	28	0	1748.5	4.2	174	5.7	0.287	27.4	53.8
0.89	0.60	-2	23	13	25.5	2	1188.2	4	250	6.1	1.539	24.5	25.8
0.94	0.82	0	30	4	24	1	1480.1	4.7	175	6.5	0.042	8.1	84.5
1.15	0.79	0	21	0	28	3	1738.8	4.8	161	6	0.021	29.8	38.1
1.14	0.79	0	25	8	25	2	1430.6	4.5	177	7.1	0.028	26.1	9.7

0.85	0.68	0	23	2	23	2	889.44	4.6	220	5.1	0.023	30.9	34.5
0.77	0.70	-1	26	1	30	2	1700.3	4.2	219	5.8	0.082	21.9	63.6
0.84	0.83	0	26	5	25.5	0	2201.5	4.6	224	5.6	0.04	18.7	50.4
	1.08	0	26	0	29	1	858.88	4.6	114	8	<0.012	31.9	16.4
0.91	0.94	0	27	23	29	0	1450.4	4.1	218	5.5	0.028	20.4	43.3
1.06	0.73	0	27	2	27.5	0	1769.6	4.9	215	5.6	0.143	27.9	40.5
0.94	0.82	0	25	20	28.5	0	1708	4.4	205	5.6	0.017	21.9	30.6
0.95	0.81	0	28	1	29	0	2170	5	216	6.1	0.481	19.7	15.1
	0.58	0	14	9	28.5	2	1531.8	4.3	192	5.4	0.059	10.7	109.5
0.98	0.66	0	26	0	27.5	2	2067.1	4.2	234	6	0.245	24.3	35.7
0.65	0.38	-34.5	11	22	15	3	1649.2	4.7	202	6.7	0.105	18.6	46.5
		0	28	1	25.5	0	2620.8	4.2	128	5.6	0.178	30.3	118.5
0.86	0.75	0	21	4	27.5	1	2338.6	4.7	130	8.9	0.121	23.6	25.8
0.88	0.85	0	24	3	28.5	0	1699.5	4.6	292	4.6	0.063	28.2	40.2
1.10	0.78	0	27	3	28	2	3714.8	4.5	127	8.8	0.366	19.3	33
1.14	1.09	0	22	0	27.5	1	1890.2	4.3	251	4.9	0.151	34.8	53.8
0.89	0.88	0	18	19	28.5	0	1941.4	4.6	163	5.9	<0.012	20.1	63.8
0.85	0.69	0	29	0	29	1	2071.7	4.6	245	8.4	0.715	25	32.4
0.88	0.71	0	28	0	26	0	1852.5	4.7	216	6	1.378	20.6	27.8
1.02	0.93	0	29	3	29	0	2798.2	4.5	175	6	0.067	30.4	36.8
1.10	0.87	0	29	1	28	0	1946.5	4.6	136	5.9	0.081	27.6	33.5
1.20	0.74	-1	23	4	27	0	2090.8	4.1	140	5.3	0.087	34.8	36.8
1.07	0.87	0	25	9	27	1	1281.4	4.3	197	5.7	0.039	25.5	75
1.13	1.12	0	26	2	29.5	0	1916.8	4.7	189	6.6	0.025	24.2	54.7
1.04	0.75	0	30	0	28	1	1188.5	4.6	182	6.2	6.429	26.6	21.2
1.00	0.75	0	24	0	28.5	1	2452.1	4.5	149	7.5	0.064	39.2	35
0.95	0.76	0	23	4	27.5	2	1701.8	4.5	156	5.8	0.051	34.4	35.8
0.74	0.58	0	15	5	26	2	1449.4	4.4	255	6.6	0.028	23.3	21.6
0.78	0.64	0	24	3	28.5	3	1452	4.4	231	5.9	0.159	26.4	44.7
1.08	0.67	0	27	14	24.5	0	1642.3	4.6	135	5.6	0.097	20.8	31.2
0.81	0.85	0	23	2	26	0	1658	4.5	187	5	0.038	36.5	50.2
0.87	0.67	-1	24	0	26.5	5	2651.3	3.7	206	5.3	0.013	19.5	43.1
0.88	0.67	0	30	1	28	2	2960.7	4.6	262	5.6	0.051	25.8	32
0.78	0.61	0	19	6	26.5	0	1548.8	4.8	238	4.9	0.151	29.7	53.2
1.28	0.82	0	25	1	28.5	1	1456.9	4.3	124	5.1	0.298	45	33.8
1.22	1.06	0	29	0	27.5	1	1848	4.7	185	6.5	0.046	33.2	32.7
0.99	0.82	0	27	0	28.5	1	1471.7	4.3	159	5.8	1.96	40.6	50.9
1.15	1.02	0	28	0	27.5	1	2471.6	4.7	228	6.8	0.046	28.5	60.6
0.88	0.57	0	25	18	22	2	1926	4.8	118	7.2	0.539	29.4	37.7
1.39	0.96	0	26	4	29.5	2	1647.8	4.1	159	5.6	0.409	36.5	33.4
1.10	0.97	0	29	0	27.5	0	1822.1	4.7	126	5.3	<0.012	31.2	13.3
1.03	0.82	0	28	0	29.5	0	3638.1	4.3	216	6.6	0.113	21.7	23.4
0.89	0.69	0	16	30	24.5	2	2161	4.6	215	5.7	0.071	18.5	42.3
1.13	0.91	0	29	2	29	0	2612.4	4.3	221	5.7	0.037	30.6	46.6
1.17	0.81	0	29	0	27	2	1910.5	4.8	136	6.3	<0.012	27	30.8
0.86	0.71	0	28	6	26.5	1	2740.2	4.6	164	8.1	0.223	26.7	28.5
1.07	0.95	0	24	0	23.5	1	1461.7	4.9	143	5.3	0.017	24	22.9
1.07	0.80	0	30	0	29	2	2001	4.4	193	5.9	0.069	27	60.3
		-12	15	17	20	2	2111.2	4.4	197	5.6	0.152	19.6	70.4
0.95	0.75	0	29	1	27	1	1937.1	4.6	248	5.5	0.075	37.7	22.8
1.03	0.76	0	26	6	23.5	3	1149.3	4.3	214	5.8	<0.012	26.4	29.4
1.01	0.74	0	28	16	28	1	2661.7	3.9	213	6.2	0.255	25.2	72.4
1.16	0.72	-0.5	29	0	28	5	1501.5	4.1	176	6.8	0.15	21.2	14.4
1.13	0.87	0	27	1	29.5	0	1620.1	4.4	191	5.7	0.128	25.6	54.9
1.34	0.91	0	23	3	27.5	0	1197.8	4.5	140	5.8	0.521	22.9	33.6
0.90	0.75	0	25	3	26.5	1	3318.2	4.3	247	9.7	0.032	21.2	26.4
1.16	0.92	0	25	12	29	0	2343.2	4.5	249	6.1	0.235	18.8	33.4
1.09	0.85	0	21	0	24.5	6	2667	4.6	143	6.8	0.022	23.6	25.3
1.13	0.95	0	28	0	30	0	2209.2	4.7	210	5.9	<0.012	24.4	31.5
		0	27	5	26	1	1408.7	4.5	165	7.6	0.089	18.2	26.9
		0	18	1	25.5	4	2599.7	4.2	198	5.8	0.461	28.9	43.7
1.43	0.92	0	22	4	27.5	0	1808.5	4	168	5.9	0.792	21.8	48.8
1.18	0.89	0	30	1	28.5	1	1740.8	4.4	177	5.6	2.004	25.2	54.8
1.09	0.88	0	30	0	28	1	2218	4.1	166	11.2	0.829	21.4	44.3
1.04	0.74	0	26	0	28.5	1	2091.9	4.8	204	6.1	0.056	22.3	21.5
1.06	0.73	0	20	0	29	0	1870.3	4.5	186	5.5	0.202	14.4	62.9
0.94	0.84	0	19	0	29	0	1881.9	4.3	190	5.6	0.015	21.7	38.1
1.01	0.83	0	28	0	28.5	0	1610.2	4.5	198	5.7	<0.012	30.8	36.3
		0	29	1	30	0	1788.4	4.3	206	5.7	0.11	24.9	65.2
0.98	0.77	-1	19	3	21	3	1622.3	4.3	186	5.8	0.136	23.4	46.8
1.19	0.60	0	20	0	23.5	3	1663.6	4.3	259	6	0.232	22.7	24
0.99	0.76	0	19	28	27	5	971.28	4.1	257	7.4	0.436	21.8	32.4

0.98	0.79	0	30	0	27	0	2500.6	4.7	205	5.7	0.222	30	27.8
0.87	0.51	-0.5	27	1	30	0	2273	4.2	171	6.4	1.128	18.4	19.6
1.05	0.90	0	19	4	28	1	1830.5	4.3	186	6.2	0.196	22	35.3
1.14	0.88	0	29	1	29.5	0	2587.4	4.7	254	5.9	0.735	30.7	31.7
0.92		0	26	1	22.5	3	1081.9	4.3	175	5.6	0.096	34	81.9
0.98	0.82	0	29	0	28.5	0	2561.1	4.4	190	6.5	0.131	18	76.1
1.08	0.80	0	30	0	27.5	0	2070.2	4.5	209	6	0.485	27	31.2
0.93	0.58	0	20	21	24	5	978.6	5	142	7.3	0.022	11.8	20
0.54	0.37	0	26	0	26.5	2	1701.4	4.5	141	5	<0.012	23	76.9
1.01	0.98	0	30	0	29.5	0	1509.7	4.3	187	5.9	<0.012	31.5	23.9
1.16	0.99	0	29	0	26.5	3	1929.3	4.8	225	8.1	0.493	33.8	19.2
0.94	0.81	-1	18	1	29	3	2071	4.4	269	6.5	0.068	23.4	42.7
0.90	0.85	0	28	0	29.5	0	1810.4	5	245	6.2	0.121	31.2	23.1
0.96	0.72	0	30	1	26	0	1300.5	4.8	225	6	<0.012	34.4	24.6
1.17	0.86	0	19	0	28	1	1757.1	4.3	158	7	0.095	21.6	63.2
1.06	0.83	0	20	2	26	0	1341.3	4.7	178	5.7	<0.012	27.6	32.3
1.06	1.01	0	29	0	28	0	1928.6	4.5	184	5.5	0.226	36.8	63.2
1.20	0.90	0	30	1	27	3	2120	4.7	172	8	0.065	33.9	24.1
0.83	0.70	0	29	3	23	3	1848.1	4.6	238	5.6	0.024	23.5	60.1
0.89	0.65	0	22	3	29	0	1980.2	4.4	258	5.8	0.035	18.9	41.3
0.90	0.95	-1	29	3	28.5	0	1230.8	4.5	240	6.3	0.219	24.7	19.2
0.83	0.81	0	27	0	28.5	0	1702	4.7	225	5.7	0.013	32.8	31.1
1.21	0.71	0	29	0	29.5	0	2806.7	4.5	193	6.3	0.02	21.6	40.1
1.46	1.06	0	28	0	28.5	1	1657.5	4.2	154	11.1	0.536	21.6	56.6
0.85	0.78	0	18	3	26.5	1	1947.7	4.7	191	5.6	0.454	44.2	28.3
0.92	0.66	0	29	0	29	0	3439.8	4.6	250	6.2	0.053	16.4	34.5
0.96	0.92	0	19	0	29.5	0	1329.9	4.5	262	5.7	0.077	12.5	47.6
0.87	0.80	0	23	7	24.5	0	1951.6	4.5	202	5.7	0.264	25.3	40.4
0.82	0.66	0	20	1	29	1	1627.2	4.5	221	6.2	0.286	21.1	33.4
0.98	0.77	0	26	7	27	4	2486.9	4.6	193	8.5	<0.012	30.3	47.7
1.07	0.71	0	22	2	28	3	1071	4.2	173	6.2	0.082	36	45.5
1.12	0.92	0	30	2	26	0	2279.7	4.6	218	6.2	0.578	20.9	41.8
1.04	0.65	-24	12	15	18.5	3	1531.2	4.6	250	5.8	0.027	22.4	35.8
0.71	0.63	0	16	0	27	2	1982.1	4.3	212	5.5	0.039	18.5	46.6
		0	24	4	24.5	2	1352.4	4.6	175	6	0.196	18.8	121.7
0.96	0.70	0	16	1	25	2	2050.8	4.6	163	5.8	0.067	26.6	49.9
1.22	0.83	0	25	1	28.5	0	2970	4.6	192	6.1	0.244	22.6	22
		-1	19	16	25.5	3	1140.8	4.5	155	6.2	0.014	18.3	45.8
1.05	0.76	-2	21	1	23.5	4	1689	4.4	189	6	0.075	17.9	52.9
0.89	0.66	0	22	3	28	0	740.88	4.3	131	5.8	0.162	10.5	76.4
		0	28	6	28.5	0	1630.7	4.7	178	4.9	0.031	19.8	27.4
1.11	0.78	0	24	1	28	2	1222.6	4.6	159	5.6	0.878	27.7	111.8
1.03	0.82	0	29	5	28.5	1	2160.4	4.5	156	6.2	1.817	24.2	20.7
1.22	0.92	0	20	0	28	0	1828.2	4.4	166	5.7	0.488	25.5	40.4
1.24	0.98	0	28	3	28.5	0	1990	4.6	240	5.9	0.15	23.7	60.9
0.99	1.00	0	26	11	29	0	1948.5	4.4	192	6.6	0.581	21.3	17.2
0.74	0.73	0	23	0	29.5	0	2221.2	4.8	158	6.4	0.183	21.9	48
0.71	0.65	0	29	0	25.5	1	1830.2	4.8	161	5.5	0.335	23.4	43.1
0.86	0.73	0	28	4	28.5	0	1548.4	4.5	245	6.2	<0.012	29.1	32.2
1.05	0.70	0	30	2	26.5	1	1540	4.8	200	5.4	<0.012	23.9	21.3
		0	29	0	29	1	1989.5	4.5	257	9.6	0.878	18.4	50.7
1.15	0.71	0	15	2	24	2	1517.9	4.1	183	5.8	0.074	28.1	36.6
1.09	0.80	0	29	14	28	0	2051.1	4.7	213	5.5	0.021	24	43.8
0.82	0.83	-1	27	2	26	0	1740.8	4.4	189	6.4	1.53	24.3	43.2
		0	27	10	28.5	0	2280.7	4.8	229	5.6	0.039	18.6	44.2
1.01	0.76	0	27	7	29.5	0	1661.3	4.5	167	5.2	0.229	19.2	48
1.08	0.66	-2.5	21	1	27	4	2768	4.5	192	6.9	0.112	23.6	58.4
1.06	0.94	0	29	0	29.5	0	2262.6	5	174	5.2	0.13	28.4	41.8
0.92	0.85	0	26	2	28	0	2128.5	4.8	223	6.2	0.081	22.9	28.8
0.95	0.79	0	28	15	27.5	1	2306	4.7	188	6.8	0.2	28.3	33
0.95	0.75	0	25	2	27	3	1750.2	4.3	217	9	0.165	23.9	17.5
		0	22	3	26.5	0	2068.3	4.3	223	5.4	0.073	17.3	34.3
1.05	0.74	0	30	4	23.5	0	1100.9	4.7	179	5.8	<0.012	15.3	42.9
0.85	0.86	0	29	16	26.5	0	2477.9	4.7	296	5.8	0.085	12.8	51.4
1.14	0.75	0	22	2	27	0	1528.6	4.8	205	5.9	0.269	19.5	29.2
0.77	0.75	0	28	1	26.5	0	2370	4.8	306	5.8	0.043	15.3	25
1.56	1.09	0	25	0	29.5	2	1732.2	4.2	210	5.7	0.06	28.6	32.8
		0	28	6	30	0	3339.3	4.6	230	5.5	0.161	22.4	40
1.10	0.73	0	29	5	27	0	1518.5	4.8	230	5	0.02	22.7	45.9
		0	25	0	29	0	1900.1	4.6	184	5.5	0.06	20.7	46.8
		0	12	16	25	3	2182.8	4.5	135	5.7	0.873	16.7	98.4
0.99	1.01	0	29	3	28.5	0	1892.2	4.4	223	5.6	<0.012	21.8	53.9

1.00	0.73	0	30	2	29	0	2340.2	4.8	225	6	0.051	21.1	49.9
1.10	0.84	0	30	1	29.5	0	2702.7	4.7	240	6.2	0.763	14.9	54.2
1.21	0.97	-0.5	30	15	26.5	3	1832.3	4.6	219	5.6	0.094	21.4	68.3
1.14	0.89	0	29	5	28.5	0	1589.8	4.8	216	5.5	0.127	16.3	27.8
0.74	0.58	0	18	0	25	0	1869.2	4.6	245	5.8	0.025	21.7	31.5
1.39	0.84	0	25	0	28.5	2	2231	4.5	205	4.9	0.155	18.2	98.7
0.67	0.66	0	23	0	26.5	0	1230.6	4.1	224	5.9	<0.012	21	25
1.22	0.92	0	20	2	26.5	0	2442	4.6	263	6.6	0.075	20.8	39.9
1.31	0.72	0	27	2	29.5	2	1267.6	4	216	5.6	0.246	29.8	63.2
1.05	0.79	0	29	0	28	0	2227.1	4.7	204	5.4	0.08	21.7	46.7
1.04	0.71	0	15	6	26.5	0	1211.5	4.4	206	6.1	0.205	17.2	85
1.07	0.93	0	30	2	28.5	0	2177.4	4.4	219	5.3	0.031	22.4	50.3
0.86	0.71	0	28	7	29	0	2082	4.8	179	5.7	0.053	19.8	33.8
		0	21	2	25	2	1412	4.2	230	5.5	0.066	20.3	52.1
1.06	0.92	-1	26	2	27	0	1199.7	4.5	193	6	0.019	18.1	35.9
1.10	0.74	0	21	11	25.5	2	1561.6	4.7	221	5.7	0.049	24.2	34.7
1.07	0.82	0	30	8	29	0	1931.4	4.4	237	5.2	0.148	21.1	16.9
0.73	0.61	0	19	1	27	0	2610	4.6	189	5.7	0.217	26.7	42.7
0.82	0.72	0	23	2	27.5	0	1872	4.3	192	6	0.022	22	47.1
0.97	0.77	0	26	4	18.5	0	1758.9	4.4	198	6.2	0.19	24.5	46.8
0.98	0.87	0	30	0	26	0	1940.1	4	227	5.5	0.026	35.3	40.6
1.28	0.97	0	28	5	27	0	1620.5	4.6	221	5.5	0.277	13	78
1.05	0.71	-1	21	3	26.5	2	1468.8	4.3	218	6.2	1.906	15.3	66
1.10	0.85	0	28	12	27	1	2808.5	4.8	212	6.8	0.422	25.6	40.7
1.35	1.14	0	30	1	29	1	1933.1	4.4	177	6.8	0.054	26.3	26.1
1.12	0.80	-0.5	28	5	27.5	1	3132.1	4.6	218	7.9	0.064	26.3	32.6
0.87	0.82	0	18	0	29	0	1662	4.2	233	6	0.165	24.2	41.5
0.81	0.68	0	28	5	26	0	1667.7	4.4	199	5.6	0.131	23.8	45.9
0.84	0.70	0	26	4	24.5	0	1911.8	4.5	173	5	0.103	16.5	66.9
1.10	0.93	0	27	3	28.5	0	1912.3	4.6	195	5.8	0.053	12.4	39.5
1.01	0.83	0	29	0	26.5	0	1838	4.5	212	4.9	0.016	20.8	42.1
	0.67	0	24	5	27.5	1	2265.7	4.1	243	5.9	0.019	15.4	85.9
1.02	0.92	0	29	0	28.5	0	1551.8	4.4	216	5.6	0.157	25.6	35.6
1.18	0.74	0	29	3	27	0	1723.7	4.5	217	5.5	0.031	21.3	32.2
1.07	0.86	0	30	0	26	0	1700.3	4.5	215	5.8	0.034	25.8	34.8
1.01	0.81	0	30	2	28	0	2288	4.6	243	5.4	0.017	29.1	32.4
0.96	0.78	0	26	0	28.5	0	2157.9	4.3	228	4.9	0.262	20.8	35.9
1.21	1.02	0	26	2	28	0	1030.3	4.4	191	5.9	0.153	27.5	25.8
1.09	0.85	0	26	0	28.5	0	1567	4.2	150	6.2	0.657	23.8	60
1.26	0.90	0	30	0	28.5	0	3012.2	4.7	170	5.8	0.025	32.5	48.3
1.02	0.81	0	22	9	25.5	1	1512.5	4.5	192	7.1	0.036	21.5	38.7
1.16	0.91	0	29	3	27.5	0	2259.4	4.8	203	5.6	0.132	12.9	19.2
0.99	0.59	0	27	6	24	2	1520.3	4.9	160	6.6	0.046	18	36.8
1.05	1.03	0	28	2	29.5	1	1770.2	4.6	238	7.4	0.063	20.6	34.1
0.78	0.70	0	22	4	28	0	863.1	3.4	119	5.1	2.636	29.9	40.6
1.04	0.81	0	21	0	27.5	3	1397.6	4.5	169	6.5	1.563	37.2	28.5
1.16	1.05	0	27	2	29	0	1661.7	4.9	221	6.2	0.088	25	17.5
1.01	0.74	0	27	0	30	0	1940.6	4.6	244	6	0.672	16.2	23.9
1.02	0.96	0	28	1	28	0	2203.4	4.3	180	6.4	0.038	23.2	31.3
1.09	0.90	0	28	0	28	0	1629	4.6	236	5.7	0.016	16.4	31
0.85	0.67	0	28	5	25	2	520.2	4.4	220	6.2	0.213	21.1	39.8
0.70	0.76	0	15	28	22	2	1722.2	4.5	212	5.7	0.146	31.7	35.6
1.01	0.84	0	21	3	24.5	0	1939.1	4.5	148	6.5	<0.012	25.4	36.4
1.05	0.72	0	28	0	26	3	1170.4	4.3	150	6.6	0.042	18.8	108.8
0.82	0.73	0	30	12	29	2	1868.8	4.2	246	5.8	0.055	31	41.9
0.87	0.47	0	26	6	25.5	0	1710.8	4.3	198	5.7	<0.012	28.4	32.1
0.62	0.48	0	18	19	19.5	0	1799.2	4.2	163	5.9	0.034	30.5	32.4
1.35	1.05	0	30	0	29	0	927.2	5	213	5.2	0.04	25	28.1
1.21	0.96	0	30	3	26.5	0	1350.3	4.4	216	5.9	0.581	22.1	43.3
1.03	0.84	0	27	7	26	0	2132	4.5	282	6.3	0.065	26.3	39
1.04	0.90	0	28	1	28.5	0	2434.7	4	234	6.3	<0.012	25.8	45.4
0.97	0.68	0	28	3	27	2	2277.9	4.4	221	5.6	<0.012	24.3	42.6
1.18	1.02	0	25	1	29	0	2501.3	4.7	220	5.9	0.013	23.4	13.1
1.11	0.73	0	28	2	28.5	0	2268.5	4.4	174	5.5	0.03	24.9	36.8
0.98	0.84	0	26	5	27	1	948.6	4.3	165	5.3	0.633	18	74.1
0.94	0.89	0	21	0	26.5	0	1349.3	4.6	188	5.6	0.042	28.3	32.3
1.01	1.01	0	30	2	29.5	0	2133	4.5	202	5.3	0.022	29.2	56.2
1.15	0.91	0	28	5	24	0	1401.2	4.6	113	5.5	0.059	9.7	57.5
1.28	0.97	0	27	0	28.5	0	1489.9	4.7	199	5.9	0.02	35.2	39.9
0.97	0.78	-2	30	1	27.5	0	2002.8	4.4	210	5.5	0.457	22.5	33
		-2	16	15	27.5	1	1487.6	4.4	191	10.7	0.873	19.8	20
0.99	0.72	0	25	3	27.5	2	2477.6	4.8	176	7	0.199	24.9	37.1

1.49	0.99	0	29	18	27	0	2436.5	4.4	196	5.6	0.315	23.9	15.3
0.96	0.75	0	29	4	28.5	2	3422.4	4.5	157	4.9	0.018	16.9	77
1.14	0.95	0	27	3	28	0	2498.2	4.6	209	5.6	0.013	25.8	16.4
0.99	0.83	0	24	0	28.5	0	1299.2	4.1	200	5.9	<0.012	21	40.4
1.05	0.80	0	26	3	27	1	2193	4.8	164	6.3	0.026	27.3	32.6
0.89	0.69	0	26	6	28	0	1481.5	4.7	185	5.6	0.192	19.4	84.6
1.26	1.10	0	30	0	29.5	0	2397.7	4.6	211	6.2	0.211	31	28.1
1.16	0.94	0	29	8	27	1	2107	4.4	160	6.2	1.5	31.5	28.5
1.16	0.93	0	27	0	27	1	1989.7	4.4	177	7.9	0.056	18.4	27.1
0.93	0.64	0	25	0	29	2	1690.1	4.5	200	6.1	0.172	12.6	57.9
0.82	0.71	0	23	3	22.5	2	1348.5	4.2	281	6.3	0.042	26.8	56.1
0.87	0.78	0	24	1	29.5	1	1308.5	4.4	137	8.2	0.015	27.2	36.9
1.02	0.72	0	29	3	27	0	1892.2	4.4	208	5.6	0.027	12	30.4
0.83	0.88	0	24	2	27	0	1638.7	4.4	268	5.6	0.025	14.9	55.5
1.25	0.94	0	27	11	27	0	2208.1	4.6	164	6.1	0.013	14	31.3
		0	26	4	26.5	0	1777.4	4.7	176	5.8	0.423	28.3	27.7
1.05	0.76	0	24	1	26.5	6	1869.6	4.4	193	6.4	0.032	23.9	60
1.46	1.05	0	28	2	21.5	0	1862.1	4.3	140	5	0.056	39.3	39.2
		0	19	4	25.5	0	1320.2	4.6	150	5.5	0.052	26.3	42.9
0.77	0.58	0	28	0	23	0	1190.8	4.6	203	5.3	<0.012	14.8	43.5
	1.13	0	25	0	30	0	3013.1	4.4	190	5.1	0.105	22.2	35.3
1.22	0.89	0	26	0	28.5	1	1501.9	4.6	179	6.2	0.014	26.5	26.2
1.26	0.81	0	26	3	27	4	1168.4	4.5	147	7.5	0.102	26.7	36
1.41	1.09	0	29	0	27	1	2332.3	4.3	191	10.5	0.972	19.9	50.9
1.21	0.98	0	21	2	29	0	1337.6	4.5	187	6	0.198	23.6	47.6
0.92	0.80	0	27	0	27	1	1960	4.7	207	6.6	0.033	24.4	25.8
0.84	0.75	0	30	1	27	0	2723	4.8	297	5.4	0.091	22	20.4
1.11	0.78	0	23	1	27	2	1957.7	4.4	238	5.9	<0.012	25.8	32.6
0.94	0.72	0	22	11	25.5	2	1607.6	3.4	113	8	0.795	33.9	63.7
1.16	0.87	0	29	6	26.5	0	2378.7	4.6	197	6.4	0.03	26.1	57.6
0.92	0.86	0	29	4	27	0	2027.7	4.3	203	6.5	0.127	26.5	44.6
0.95	0.77	0	27	0	28.5	0	1548.4	4.5	265	6.3	0.995	19.5	27.1
1.06	0.73	0	25	4	26	0	2029.4	4.5	176	5.5	0.022	25	62.6
1.14	0.88	0	29	6	25.5	0	2008.8	4.4	150	6.6	0.446	26.7	73.2
0.62	0.63	0	23	3	25.5	2	1682.1	4.5	229	6.5	0.095	19.5	37.3
1.02	0.89	0	23	6	27.5	2	2923.2	4.5	231	6.4	0.014	19.2	31.1
0.90	0.80	0	26	3	28	0	1678.3	4.5	169	5.5	0.017	27.2	37.5
0.84	0.70	-1	17	1	24.5	3	1340.8	4.3	181	6.5	0.034	17.9	26.5
0.76	0.75	0	26	0	26.5	1	1458.6	4.3	242	5.9	0.248	28.1	20.1
1.00	0.90	0	19	2	27	1	2118.2	4	165	6.5	0.256	15.4	77.9
1.03	0.95	0	28	7	24.5	0	1608	4.6	179	5.5	<0.012	35.6	43.9
0.82	0.63	0	27	0	25	0	1951	4.5	209	5.5	0.741	17.8	36.4
0.97	0.86	0	21	7	28	0	1329.7	4.3	195	6.2	0.179	24.8	23.3
1.11	0.81	0	26	2	26.5	0	2756.5	4.1	213	6.4	0.31	21.6	35.4
1.25	1.00	0	20	7	26.5	0	2582.3	4.7	235	6	0.033	22.2	37.3
1.17	0.92	0	29	0	23.5	0	1040.6	4.5	185	4.8	0.056	15.8	60.2
1.02	0.95	0	30	0	29	0	2497	4.2	216	6	0.386	18.8	31.2
1.19	0.96	0	29	0	25	0	1777.4	4.5	201	5.9	0.015	21.6	18
		0	24	0	27	2	1368.8	4.3	174	5.4	0.077	18.5	36.8
0.96	0.92	0	18	5	28.5	1	1540.4	3.6	157	5.6	0.387	24.3	25.5
0.92	0.76	0	20	11	25	1	2381.4	4.4	197	6	0.201	19	51.2
0.83	0.79	0	30	0	27.5	2	1202.4	4.7	168	6	0.068	21.5	68.5
1.33	1.07	0	26	0	29	0	2669.4	4.1	156	5.9	0.022	32.8	41.2
0.68	0.63	0	22	5	27.5	1	2707.9	4.6	209	6	0.173	13.6	20.7
1.23	0.90	0	27	9	27.5	0	1728.3	4.6	228	5.9	0.032	15.9	40
1.19	0.93	0	26	1	30	0	2102.4	4.1	147	5.2	0.079	30.5	134
0.79	0.83	0	23	14	24.5	2	2688.6	4.5	160	7.1	<0.012	12.4	30
1.52	0.94	-1	25	1	28.5	3	2358.5	4.5	192	6.6	0.021	19.4	17
0.77	0.53	0	29	9	26.5	0	1738.6	4.4	205	5.2	<0.012	18.5	26.1
0.76	0.80	0	20	1	26	2	1009.8	4.4	166	5.4	<0.012	24.5	61.3
0.87	0.91	0	28	0	28.5	0	2539.1	4.6	176	5.8	0.031	25.6	45.7
0.96	0.91	0	15	3	27	2	1621.1	4.2	202	6.6	0.265	12.9	34.9
0.96	0.81	0	28	1	27.5	0	1227.7	4.5	194	5.9	0.65	23.4	26.6
0.76	0.50	0	14	1	27	2		4.1	175	6.1	0.039	27.2	57.3
1.11	0.95	0	24	5	28	0	1321	4.2	170	6.1	0.022	22.2	64.5
0.79	0.70	0	23	6	29	0	971.73	3.8	179	6	<0.012	11.7	48.2
0.83	0.72	0	28	0	26	0	2417.9	4.8	197	6.3	0.074	23.8	47
0.90	0.92	0	22	1	26	2	3413.8	4	197	5.5	0.069	23.1	29
0.88	0.60	0	23	0	27	0	3499.7	4.1	163	5.9	0.172	15.1	60.8
1.29	1.00	0	25	0	28.5	0	2378.7	4.5	159	5.6	0.215	32.3	32.8
1.34	1.13	0	30	1	27.5	1	1240.8	4.1	161	6.5	0.53	31.4	22.7
0.99	0.73	0	22	2	27.5	1	1870.3	4.4	192	6.3	0.114	16.7	43.8

1.10	0.99	0	24	1	27.5	1	1810.4	4.6	158	5.3	0.176	24.2	21.6
1.15	0.70	0	29	0	26.5	2	1141.9	4.4	151	5	0.039	19.5	41.2
0.95	0.80	0	30	3	27	0	1428.8	4.4	219	5.9	0.029	8.8	58.8
0.98	0.89	0	29	6	26.5	0	1572.8	4.1	167	5.7	2.013	28.3	67.2
1.25	1.11	0	28	2	29	0	1391.5	4.2	248	6.1	0.299	33.6	36.4
1.17	0.89	0	29	4	27.5	3	1989.2	4.4	189	5.3	0.047	21.1	16.4
0.87	0.63	0	27	3	25	2	858.39	3.8	139	5.9	0.016	35	23.9
1.32	1.01	0	22	0	29.5	1	1622	4.2	239	7.1	0.014	27	28.8
1.06	1.04	0	18	2	26	1	2080.5	4.3	198	5.5	0.048	20.9	47.7
0.79	0.71	0	30	2	29	0	1539.9	3.6	143	5.4	0.034	38.6	51.9
0.68	0.61	0	21	8	29	0	1610.2	4.5	215	6	0.067	25.5	45.3
1.09	0.88	0	29	2	27	1	1577.7	4.4	193	7.4	<0.012	26.8	68.3
1.12	0.97	0	25	0	29.5	0	2149.7	4.7	233	5.8	0.026	22.8	87
0.90	0.77	0	21	12	28.5	3	1778	4.8	213	5.6	0.112	15.1	50.2
1.20	0.97	0	29	1	27.5	0	2232.9	4.1	207	5.9	0.061	31.4	55.1
0.99	0.85	0	24	0	27	1	1238.4	4.4	195	6.1	0.028	23.8	37.5
0.95	0.76	0	20	0	27	0	2330.7	4.7	242	6.1	0.032	18.2	46.5
1.12	0.92	0	28	0	27.5	1	1439.6	4.6	209	5.6	0.054	23.3	59.5
0.71	0.68	0	22	3	24	0	1288.4	4.3	252	5.8	<0.012	17.9	85.6
0.96	0.79	0	29	0	28	2	1904.9	4.5	149	5.7	0.979	23.1	783.5
1.02	0.82	0	28	3	24.5	1	1530	4.6	252	7	0.074	13.8	25.5
1.26	0.73	0	30	0	28.5	0	1740.5	4.7	157	5.7	0.268	19.9	69.3
0.84	0.70	0	27	0	29	0	2088	4.4	175	5.7	0.057	20.9	43.7
1.14	1.02	0	30	0	28	0	1030.6	4.3	216	6	0.108	12.5	60.2
1.03	0.83	0	27	0	25	0	1649.3	4.4	256	6	0.139	26.6	13.7
1.02	0.92	0	27	1	29	0	1461.2	4.4	183	6	0.055	33.9	54.7
0.88	0.93	0	22	4	26.5	0	2208	4.7	201	6.1	0.067	16.9	53.5
0.96	0.82	0	27	3	27	0	1620	4.5	178	6.4	0.144	20.2	50.5
1.01	0.81	0	29	8	28	1	1780.8	4.4	203	5.7	0.125	22.1	57
0.86	0.90	0	28	2	24	0	2679	4.2	123	5.5	<0.012	32.7	28.1
1.22	1.07	0	30	0	28.5	0		4.7	156	5.5	0.02	27.2	23.1
1.07	0.85	0	29	1	28	2	1832.1	4.1	234	5.5	0.039	25.2	29.4
1.01	0.80	0	24	13	27.5	0	2476.8	4.5	196	6.2	0.027	23.6	24.3
1.15	0.82	0	30	0	28	0	1019.2	4.8	191	5	0.253	21.3	81
1.12	0.87	0	29	0	28	0	2268	4.4	138	6.2	0.324	27	18.1
1.07	0.78	0	26	0	27	2	1079	4.1	266	6.1	0.085	19.4	31.1
1.38	1.06	0	28	13	27	0	797.56	4.7	200	5.9	0.054	29.5	23.8
1.01	0.79	0	23	1	28	2	1637.3	4.3	184	5.6	0.144	30	30.5
0.93	0.66	0	29	1	23	2	1368.3	4.8	201	5.7	0.023	24.6	26.2
1.10	0.98	0	24	0	27.5	1	1741.7	4.8	160	9	0.221	30.4	7.7
1.11	0.84	0	20	11	27.5	1	1261.7	4.4	181	7.8	0.078	21.6	50.9
0.97	0.91	0	29	0	27.5	2	621.32	4	205	5.8	<0.012	24	32.5
0.87	0.88	0	29	0	27	0	2582.6	4.4	173	6.1	0.331	23	33.3
0.78	0.73	0	20	35	23.5	2	1319.5	4.3	168	5.5	0.224	15.2	100
1.32	1.26	-12.5	24	3	27.5	1	981.18	4.4	170	5.5	0.063	35	31.9
0.79	0.73	0	27	17	27	1	2207.7	4.3	139	5.3	0.08	26.8	42.8
0.91	0.71	0	29	0	27	0	2360.8	4.3	211	5.9	<0.012	16.2	41.6
0.86	0.67	0	25	0	26	0	2110.7	4.6	262	5.6	0.054	28.4	14.8
1.24	1.01	0	30	12	28	0	1552.5	4.4	206	5.5	0.283	14	46.9
1.05	0.82	0	29	2	27	3	2872.8	4.4	164	5.6	0.184	14.5	79.2
0.65	0.68	0	27	0	26	2	1740.6	4.2	219	5.8	0.152	22.6	46.1
0.84	0.83	0	30	1	27	0	1699.9	4.5	198	6.1	0.26	30.5	28.2
1.16	1.12	0	29	0	29	2	2561.5	4.3	243	6.1	0.02	30.7	44.8
0.94	0.73	-1	13	6	25	3	3396.6	4.4	188	6.1	1.372	23	29.2
0.96	0.82	0	26	0	26.5	0	2201.5	4.6	181	5.5	<0.012	25.6	24.1
1.05	1.08	0	29	8	27.5	0	1778.6	4.7	178	5.3	<0.012	27.9	48.9
0.88	0.85	0	30	2	29	2	3431.2	4.2	171	5.7	0.179	25.3	43.6
1.23	0.95	0	22	0	26.5	2	1328	4.2	167	6.3	0.174	29.2	62.7
1.22	1.03	0	30	4	25	0	1520	4.5	197	5.2	0.029	26.7	35.7
1.10	0.80	0	30	0	27	0	1921.7	4.5	165	5.3	<0.012	27.1	34.8
1.05	0.92	0	28	9	25	1	2490	4.6	227	6.2	0.154	24.1	61.5
0.87	0.99	-0.5	26	1	26.5	1	2300.4	4.5	172	6.6	0.573	24.4	59.7
0.89	0.67	0	28	3	26.5	0	1258.5	4.4	159	5.9	0.026	23.3	42.8
0.99	0.82	0	27	0	27	0	1770.2	4.7	209	5.7	0.016	22.9	47.5
1.16	0.96	0	30	0	28.5	8	1978.7	4.3	216	5.6	0.066	24.9	28.3
		0	25	0	28.5	0	1972.3	4.4	212	5.9	0.049	26	47.8
1.29	1.19	0	26	8	27.5	0	1640.8	4.2	152	6.8	0.028	28.7	22.4
1.28	1.00	0	30	1	28.5	1	1528.9	4.9	134	6.6	0.014	31.7	20.4
1.03	0.92	0	23	0	29	0	1269.5	3.6	125	5.4	0.051	21.1	19
1.47	1.15	0	28	0	29.5	0	2580.8	4.7	161	6	<0.012	25.5	52.3
0.91	0.75	0	28	8	23	3	2161	4.3	175	5.6	0.1	27	34.6
1.23	1.14	0	30	2	28.5	0	2900.2	4.3	208	5.5	0.186	21.3	33.5

1.09	0.88	0	28	6	26.5	0	1960.6	4.3	203	5.3	0.055	16.6	37.3
0.71	0.62	0	21	1	29.5	0	1271.1	4	170	5.8	<0.012	17.3	54.7
1.02	0.77	0	29	0	28	0	1230.1	4.8	210	5.7	0.023	24	87.1
1.06	0.87	0	25	7	28	0	1611	4.4	172	6.4	<0.012	25.5	18.1
1.03	0.84	0	21	3	27	0	2458.3	4.5	147	6.4	1.424	38.2	17.8
1.24	0.99	0	20	0	27.5	0	3647.2	4.5	172	5.9	1.236	31.5	30.4
1.37	0.61	0	28	0	28.5	1	1209	4	185	5.4	0.293	17.8	33.7
1.21	1.06	0	27	0	28	3	1446.9	4.3	170	5.9	0.387	27.5	65.7
1.28	1.01	0	29	4	28.5	2	2337	4.4	174	7.1	0.059	13	48.2
1.25	0.90	0	28	0	26.5	3	2439.5	4.8	146	5.7	0.898	33.9	15.1
0.93	0.77	0	30	5	25.5	0	1590.9	4.7	164	6.5	0.146	31	34.5
1.15	0.66	-2	24	10	26.5	2	1051.5	4.4	195	6	0.151	23.7	21.7
1.14	0.77	0	29	0	25	0	1441.4	4.2	192	6.2	0.028	22.3	21.1
1.09	0.79	0	30	3	26.5	4	1200.4	4.6	174	6.7	0.055	25.1	53.8
1.09	0.96	0	30	0	30	0	1446.4	4.4	177	5.8	0.038	29.3	43.5
1.12	0.88	0	27	0	28.5	0	1938.8	4.5	172	7.2	0.035	32.9	22.6
0.98	0.69	0	26	0	29	0	3003.5	4.4	247	6.4	0.15	22.6	26.4
1.00	0.85	0	26	2	28	2	2482.2	4.6	171	7.4	0.034	18.8	63.6
1.00	0.84	0	30	12	28.5	3	1290.8	4.2	198	5.8	0.027	26.6	13.1
0.72	0.62	0	22	0	28.5	2	1440.4	4.3	217	5.7	0.04	38.7	98.8
0.91	0.75	0	25	6	27.5	2	1370.6	4.4	172	5.5	0.431	20.6	55.5
0.89	0.74	0	19	1	26	3	1011.4	4.5	213	4.8	0.052	16.3	55.5
1.34	1.08	0	22	3	28.5	3	1732.3	4.3	152	8.5	0.362	26.5	28.3
1.09	0.82	0	29	8	28	0	2852.4	4.1	213	5.8	0.106	22.7	45.2
0.93	0.90	-0.5	21	0	28	0	1010.1	4.6	198	5.8	0.051	19.3	56.5
1.01	0.75	0	30	0	29.5	0	2613.3	4.4	204	5.9	0.102	19.6	68.8
0.83	0.81	0	20	1	28	0	2373.3	4.8	182	6.7	0.019	21.8	22.1
0.89	0.81	0	21	7	24.5	2	1856.9	4.1	167	6.8	0.014	20.5	48.2
1.28	0.99	0	29	0	29	1	2130.1	4.6	240	5.7	0.04	15.5	72.2
0.82	0.69	0	28	0	25	2	1741.5	4.6	204	6	<0.012	30.4	38.5
1.31	1.06	0	25	0	27.5	0	2050.9	4.4	156	6.2	1.477	34.2	15.6
0.79	0.72	0	29	0	26	0	3810.6	4.8	181	5.4	0.128	23.7	53.5
1.16	0.96	0	30	0	28	0	2328.3	4.4	208	5.8	0.043	26.7	37.5
1.11	0.97	0	30	0	28.5	1	1799.7	4.7	150	7.4	0.207	21.5	16.7
1.36	0.78	0	14	19	26	4	1771.6	4.3	152	5.6	0.109	24.7	48.2
0.77	0.69	0	29	0	27	3	760.32	4.2	141	5.7	0.074	25.6	27.6
1.06	0.82	0	25	0	25.5	0	1631.3	4.4	193	5.8	<0.012	22.3	28.4
0.88	0.73	0	26	10	30	0	2381.1	4.2	205	5.5	<0.012	25.2	55
		0	28	2	28.5	1	2775.5	4.4	165	4.3	0.015	47.8	22
1.25	0.85	0	27	0	29.5	0	1511.2	4.5	145	5.4	0.04	32.4	49.5
0.96	0.84	0	28	0	26.5	0	2849.1	4.4	177	5.9	0.053	40.2	45.8
1.26	1.05	0	26	0	29.5	2	2697	4.5	190	5.7	0.908	22.7	52.5
1.03	0.79	0	20	0	29	0	1781.5	4.4	183	5.2	0.053	29.5	56.3
1.18	0.83	0	30	0	29.5	0	2191.6	4.5	180	5.5	0.657	22.7	45.3
1.54	1.10	0	27	0	29	4	1311	4.1	175	5.5	0.019	29.9	65
		0	22	0	27	0	2229.5	4.3	167	5.6	<0.012	32.2	49.6
0.86	0.69	0	28	1	22.5	2	1870.3	4.3	159	5.6	0.022	19.5	45
0.99	0.78	0	28	0	25.5	1	2727.7	4.4	191	7.8	0.158	17.7	26.4
0.84	0.79	0	29	0	28.5	0	1780	4.4	200	5.7	0.084	28.2	41.2
0.77	0.70	0	28	8	26.5	2	4155.3	4.4	155	5.4	0.506	26.4	53
1.05	0.89	0	30	0	28	0	2120.6	4.5	169	6.2	0.133	25.6	19.2
0.88	0.83	0	27	5	28.5	7	1039	4.5	162	5.4	0.013	28.8	52.2
0.77	0.73	0	25	1	26	0	1550	4.2	188	5.9	0.198	19.2	58.4
1.00	0.87	0	27	0	29	0	2801	4.6	197	5.8	0.015	25.4	15
1.16	1.07	0	30	0	28	1	2681.8	4.3	168	5.9	0.161	27.1	29
		0	25	8	27	0	1378.8	4.5	209	5.7	0.026	24.3	31.4
1.23	0.97	0	27	10	29.5	3	1960.5	4.7	190	5.7	0.014	16.1	36.9
0.68	0.54	0	19	0	25	3	2147.5	4.5	200	6	0.429	15	53.7
0.92	0.79	0	30	0	26.5	2	3102.4	5	192	9.4	0.107	19.5	29.9
0.97	1.00	0	28	0	27.5	0	1699.3	4.7	168	5.1	0.29	38.9	16.1
0.92	0.56	0	18	1	23.5	3	1658.8	4.2	195	5.9	1.669	24.7	28.3
1.10	0.97	0	27	1	27.5	2	1795.5	4.5	208	8.3	0.197	23.4	31.9
1.06	0.84	0	25	10	25.5	0	1128.1	4.7	172	5.7	0.079	22.5	38
0.73	0.56	0	9	0	28	2	2501.7	4.4	136	6.2	0.019	30.2	52.6
0.85	0.69	-1	26	0	27.5	3	1608.8	4.2	176	6.8	0.013	25.2	50.2
0.81	0.67	0	23	2	26.5	4	2412.6	4.1	200	7.7	0.042	17.1	25.4
1.04	0.84	0	21	0	28.5	0	1818.5	4.2	164	5.5	1.51	34.9	39.9
0.88	0.80	0	23	0	30	2	2762.8	4.1	192	5.8	0.936	26.1	22.6
1.22	0.93	0	27	0	25	1	1803.4	4.3	172	6.8	0.108	35.4	61.1
1.16	0.87	0	28	0	29.5	3	2141.1	4.6	214	5.7	0.097	25.8	47.6
0.98	0.83	0	25	9	29	2	2099.3	4.5	181	5.2	0.016	23.4	34.6
0.80	0.67	-1	27	5	29	2	1200.1	4.2	169	6.1	0.033	16.1	50.4

0.82	0.78	0	22	0	24.5	0	1890	4.3	177	6.1	0.015	21.8	44.3
1.17	0.76	0	24	10	27	4	1378.4	4.2	130	8.1	0.046	27.3	216
0.97	0.63	0	23	1	29	2	1822.1	4.3	165	6	0.191	34.2	52.5
0.93	0.90	0	27	0	26.5	2	1813.8	4.3	216	4.9	0.296	26.5	32.1
0.93	0.71	0	28	0	28	3	3021.7	4.2	194	6.1	0.022	18.5	60.9
0.73	0.67	-1	22	6	26	5	1386.4	4	209	6	1.43	21.1	28.7
0.96	0.65	0	25	1	26	3	2128	4.4	143	7.4	0.073	19.5	114.6
1.02	0.86	0	18	2	28	0	2271.8	4.6	194	5.6	0.014	34.3	46.8
	0.83	-1.5	23	1	24.5	3	1597.6	4.4	170	5.8	<0.012	22.1	29.1
1.01	0.83	0	27	1	26.5	2	2322	4.6	259	5.7	0.073	21.5	48.4
1.39	0.91	0	17	7	25	3	1447.4	4.3	207	5.6	0.517	32.5	35
1.22	1.01	0	21	1	27.5	1	1020.4	3.8	123	5.6	0.045	40.7	50.7
0.78	0.64	0	23	4	24	3	1518.9	4.5	140	5.9	<0.012	28.5	42.9
0.84	0.82	0	22	0	24.5	2	2107.4	4.3	115	7	0.031	35.6	44.6
0.78	0.73	0	29	0	28	3	1708.2	4.4	187	5.9	0.114	37.9	44.5
0.75	0.61	0	22	0	26	2	1495.5	4.5	184	5.3	<0.012	18.3	53.2
1.14	0.82	0	26	1	29	1	1489.9	4.2	206	5.4	0.031	24	67
1.00	0.77	0	24	1	27	3	1489.6	4.6	234	5.6	0.168	24.6	36.1
1.36	0.92	0	25	2	28.5	2	1869.7	4.2	170	5.8	0.015	31.2	51.2
1.16	0.93	0	27	0	27.5	2	2069.7	4.2	142	5.3	<0.012	30.4	26.3
0.94	0.62	0	19	0	29	3	2186.9	4.3	125	5.9	0.134	47.7	50.6
0.84	0.79	0	23	0	26	1	2122.2	4.2	252	7	0.113	25.3	31.4
1.26	0.94	0	23	2	25	3	1512.3	3.9	180	5.9	0.317	32.9	36
0.84	0.63	0	19	8	25.5	2	1940.4	4.3	153	5.6	0.853	31.6	14.1
1.42	0.94	0	27	0	28	2	1261.4	4.4	184	6.1	0.228	29.5	30.4
0.73	0.67	0	20	4	30	1	1792.5	4.1	202	6.1	0.276	19.6	38.3
1.12	0.87	0	26	1	29	0	2178.1	4.4	239	6.1	0.061	34.4	114.5
1.17	0.87	0	26	4	27.5	2	1502.2	4.6	192	5.6	<0.012	42.7	31.5
1.03	0.77	0	27	0	27.5	0	1623.2	4	173	5.6	0.028	27.6	40.7
1.47	1.05	0	23	0	26.5	4	1548.5	4.6	160	7.3	0.582	23	108.2
0.99	0.84	0	19	1	26.5	1	1650.5	4.6	240	6.2	0.412	17	37.3
1.47	0.92	0	27	8	28.5	3	2102.5	4.4	222	5.8	0.149	33.1	64.1
1.05	1.15	0	13	0	25.5	4	2153	3.8	202	10.9	0.796	15.6	43.1
1.40	0.95	0	26	2	28.5	2	1840	4.4	173	6.3	<0.012	19	33.7
0.63	0.69	0	21	2	27	2	1579.2	4.5	146	5.8	<0.012	12.1	34.9
0.89	0.71	0	28	0	27.5	0	2911.7	4.3	210	5.5	0.112	18.2	52
1.46	1.25	0	28	0	29.5	3	2131.9	4.1	280	6.1	0.156	38.4	38
0.94	0.74	0	17	0	26.5	1	1150.7	4.1	155	6.1	0.1	22.2	45.2
1.10	0.94	0	27	1	25.5	3	2112	4.3	182	5.6	<0.012	23.6	29.4
1.01	1.12	0	26	0	29.5	1	1509.6	4.7	198	5.4	0.018	26.4	22.1
1.13	0.81	0	20	0	26	4	1978.2	4.5	152	7.4	0.021	21.8	20.9
1.05	0.89	0	24	0	27	2	1971.2	4.4	150	5.5	0.091	33.7	40.1
1.34	1.05	0	26	0	27.5	1	2832.5	4.1	196	5.8	0.624	27.4	44
0.69	0.64	0	23	7	25	2	981.75	4.4	213	5.7	<0.012	24.3	32.4
1.15	0.84	0	28	2	26.5	2	2711.5	4.8	190	6.7	0.089	18.3	5.8
0.86	0.78	0	20	0	29.5	1	1712.1	4.2	190	5.9	0.146	23.5	46.7
1.14	1.12	0	27	0	29.5	1	2371.6	4.6	216	6.2	0.035	19.4	49.2
0.86	0.65	0	22	0	24.5	4	2467.5	4.9	153	6	0.388	25.6	52.3
1.09	0.87	0	30	0	28.5	0	2756.6	4	179	6.9	0.076	17.1	58.1
1.34	0.97	0	26	10	26.5	3	942.48	4.3	188	6	0.095	19	54.2
0.89	0.72	0	26	0	25.5	3	1498.2	4.5	201	4.2	<0.012	19.6	47.7
0.83	0.70	0	10	0	26.5	0	2376.3	4.5	205	6	0.033	15.7	72.6
0.74	0.64	0	26	0	27.5	0	1729.4	4.6	171	6.1	0.237	14.7	29
0.70	0.56	0	23	2	27.5	0	2999.5	4.5	159	6.3	0.04	23.1	48.8
1.83	0.88	0	29	0	28	1	1551.8	4.5	195	6.2	0.178	17.1	44.7
1.29	0.93	0	30	0	29.5	0	1918.9	4.5	191	5.9	0.156	25	37.2
1.69	0.91	0	28	1	28	4	1762.5	4.3	251	7.5	0.093	24.3	32.9
1.10	0.92	0	22	1	27	3	3328.4	4.4	152	6.8	0.459	31.9	36.1
1.13	0.91	0	19	0	28	2	1859.6	4.8	234	6	0.233	18.7	27.2
0.90	0.89	0	30	1	28.5	1	1988.5	4.7	174	5.9	2.005	16.3	46
0.92	0.87	0	20	0	28	0	1729.7	4.6	202	6.5	<0.012	18.1	54
1.18	0.96	0	21	2	27.5	0	1862.5	4.9	209	5.6	0.916	23.1	30.8
1.33	0.81	0	12	1	26.5	2	1691.6	4.4	155	5.7	2.514	22.1	23.9
1.08	0.76	0	24	2	24	3	1030.4	4.7	180	6	<0.012	25.8	44.6
0.83	0.77	0	26	0	28.5	3	2430.8	4.6	158	6.9	0.258	27.1	64.8
1.18	0.63	0	28	1	27	3	1642.5	4.2	174	6.2	0.131	27	61.1
1.13	0.77	0	25	4	28	1	2736.4	4.4	178	6.4	0.126	19	41.1
1.03	0.93	0	20	2	26	0	1132.2	4.1	171	6	<0.012	19.5	59
0.84	0.78	0	20	0	27.5	2	1778.5	4.6	228	8.3	0.075	21.2	26.2
0.96	0.70	0	16	2	29	3	2395.4	4.7	314	6.1	0.592	20.2	32.9
1.20	0.92	0	28	0	29.5	0	1481	4.6	187	5.2	0.244	30.3	39.9
0.93	0.74	0	22	2	28	2	2647.4	4.5	200	6	0.028	26.2	28.2

0.71	0.94	0	27	0	27.5	1	1539.8	4.4	202	5.7	0.025	25.4	30.2
0.68	0.56	0	22	4	24	4	1669.6	4.6	166	5.6	0.118	23.8	38.7
		-1.5	14	9	23.5	3	3659.8	4.3	162	8.1	1.16	17.3	23.9
0.85	0.55	0	26	1	24.5	0	2596.2	4.6	181	6.6	0.158	30.2	35.5
0.76	0.54	0	19	0	27.5	3	1722.3	4.3	166	5.9	0.033	20.7	147.7
1.13	0.93	0	28	0	26	4	1072.6	4.1	161	5.6	4.141	23.3	96.1
1.09	0.78	0	17	1	27	2	2790.9	4.3	216	5.5	0.298	24.4	34.1
0.74	0.63	0	26	0	24.5	2	2610	4.3	145	6.1	0.135	29.1	45.6
0.81	0.70	0	19	2	28	3	1519.4	4.5	221	6.1	0.034	15.7	51.2
0.92	0.81	0	23	0	26.5	4	1669.9	4.5	178	7.5	0.048	25	17
0.71	0.67	0	26	2	24	1	2032.3	4.5	217	5.8	0.136	17.4	36.5
0.92	0.70	0	25	0	27.5	0	1720.2	4.2	200	5.6	0.237	24.3	93.6
1.27	1.14	0	28	1	28	5	2660.7	4.2	185	11.5	0.24	23.5	57
0.92	0.74	0	27	0	28.5	3	1321.6	4.5	166	5.6	0.836	22.1	18.4
1.27	0.79	0	24	6	27.5	4	1701.9	4.4	247	6.1	0.106	20.4	59.2
1.01	0.81	0	24	1	26.5	3	3203.9	4.3	173	7.1	0.026	23.5	39.8
0.97	0.72	0	28	15	24.5	2	3340.5	4.6	163	5.1	0.018	24.4	16.8
1.34	1.11	0	26	0	29.5	2	1687.1	4.7	240	5.5	0.103	56.8	23.6
0.97	0.74	0	20	1	27	3	1217.2	4.5	223	6.6	0.076	27.5	49.9
0.86	0.73	0	28	0	27.5	1	2012.8	4.7	172	7.2	0.284	29.4	30
0.94	0.87	0	25	0	27.5	0	1549	4.8	206	5.7	0.019	26.9	70
1.16	0.72	0	28	0	29.5	3	2543.7	4.2	138	5.6	0.837	34.2	24.1
	0.62	-3.5	16	8	26.5	2	789.94	4.4	170	5.6	0.025	41.5	45.9
0.92	0.85	0	20	0	28.5	2	1837.5	4.5	232	6.1	<0.012	23.6	51.7
0.80	0.57	0	24	1	25	2	1500.2	4.4	224	5.4	<0.012	24.6	27.3
0.81	0.71	0	20	0	22	4	2543.8	4.5	224	8.7	0.114	29.9	28.3
0.73	0.65	0	20	1	27	4	2361.6	4.5	135	7.3	<0.012	30.4	36.3
1.08	0.89	-10	23	16	21.5	3	2002.2	4.5	193	6.9	0.507	15	31.8
1.20	0.90	0	19	3	26.5	4	1380.3	4.9	140	6	<0.012	27.8	57.2
0.65	0.60	0	27	1	27.5	1	1550.1	4	197	5.7	0.096	13.6	54.9
1.02	0.97	0	22	1	25	1	1320.2	4.4	194	5.3	0.167	24.7	41
		0	23	0	29.5	3	1640.8	4.6	189	5.7	0.065	21.5	34.8
1.03	0.63	0	19	1	27.5	3	1978	4.5	145	7.9	0.109	22.2	38.6
0.94	0.71	0	21	0	28	2	2167.6	4.3	215	5.6	0.04	14.3	83.6
0.78	0.66	0	20	1	28	2	981.55	4.5	218	4.9	0.054	25.5	47.3
1.01	0.91	0	25	0	27.5	2	3333.3	4.3	180	5.8	0.084	23.1	18.9
		0	16	0	28.5	2	3261.9	4.3	184	6.4	0.333	27.3	30.8
	0.64	0	29	1	28	0	1070.5	4.1	167	5.9	0.07	9.9	60.5
0.95	0.77	0	28	1	27	1	1641.5	4.6	210	6.1	0.072	33.5	19.9
0.90	0.74	0	27	1	28	0	1650.2	4.5	254	6.2	0.023	19.4	36.7
0.65	0.56	0	14	2	27.5	2	2218.6	4.7	172	6.1	0.595	21.6	40
0.78	0.46	0	23	2	29.5	2	2321.3	4.4	188	5.9	0.129	15.1	107.7
0.93	0.92	0	22	2	29.5	2	1439.2	4.3	188	6.2	0.07	32.9	34.1
1.08	0.86	0	28	0	29.5	2	1401.3	4.5	233	5.9	0.068	27.4	24.2
0.83	0.60	0	23	0	26.5	3	1462.9	4.1	175	7	0.033	17.9	49.6
0.94	0.80	0	29	0	25.5	1	2298.2	5.2	217	6.4	0.063	23	46.7
	0.91	0	25	0	27.5	2	1189.6	4.7	239	5.9	0.585	44.5	48
0.87	0.68	0	23	2	29.5	0	2607.6	4.7	196	5.4	0.051	20.6	35.2
0.83	0.72	0	19	0	28	0	2030.4	4.5	257	5.8	0.13	23.7	72.8
1.44	1.13	0	25	0	27.5	2	1480	4.7	119	5.6	0.128	21.4	52.4
0.78	0.61	0	24	0	28	2	838.08	3.2	142	4.7	0.749	12.2	57.7
1.06	0.93	0	24	0	24.5	0	968.2	4.4	209	4.9	0.028	30.5	91.5
0.85	0.77	0	21	2	28	2	2447.8	4.6	204	6	1.335	16.3	32.8
1.23	0.90	0	29	0	26.5	2	1458.9	4.1	165	5	0.046	29.2	22.1
0.75	0.61	0	26	2	27	2	1762.5	4.4	181	6.1	0.441	20.2	90.3
1.01	0.75	-1	17	9	25.5	2	3469.7	4.4	176	7	0.435	28.4	52.7
0.75	0.69	0	26	2	24.5	2	902	4.3	207	5.6	0.031	23.9	61.5
1.14	0.91	0	25	0	30	0	1332	4.7	246	5.7	0.251	45.6	53.8
1.28	0.73	0	25	0	28.5	2	1629.7	4.3	184	6	0.102	31.2	31.8
0.70	0.49	0	25	0	23	5	1351.1	4	145	6.5	0.027	21.9	47.6
0.81	0.69	0	30	0	28	0	2751.8	4.2	228	5.9	0.146	22.3	73.7
1.11	1.12	0	23	2	26.5	2	2257.4	4.5	209	5.9	0.262	34.9	22.2
1.02	0.59	-0.5	11	3	27.5	2	1642.9	4.5	233	6.6	0.149	18.3	49.7
0.86	0.64	0	20	0	26.5	2	2140.4	4.8	179	6.7	0.038	17.1	49.6
0.95	0.80	0	22	2	27	3	2331	4.8	160	9.2	0.263	20.9	43.4
0.89	0.75	0	21	0	28	1	3250.4	4.2	197	5.8	0.597	24.2	42
1.06	0.88	0	29	2	28	4	1412.2	4.8	177	6.5	0.199	36.6	34.8
0.96	0.78	0	26	1	28.5	3	1698.3	4.3	221	5.7	0.574	22.3	51.7
0.89	0.90	0	27	7	26.5	0	2957.8	4.4	226	5.6	0.147	19.7	34.5
0.78	0.87	0	25	2	26.5	3	1409.8	4.7	141	5.9	0.083	30	31.4
1.22	0.98	0	28	0	28.5	1	3004.4	4.5	210	5.6	0.29	19.3	29.2
1.11	0.79	0	25	0	28.5	3	1375.8	4.5	185	11.3	0.251	28.9	39.4

1.06	0.80	0	26	0	28	0	2507.4	4.3	211	6.4	0.116	29.1	27
1.03	0.88	0	24	0	29.5	0	2012.9	4.7	240	6.5	0.097	19.9	35.2
0.87	0.73	0	17	2	25.5	2	2511.6	4.6	193	5.9	0.565	22.4	20.2
0.87	0.74	0	20	0	27	0	2118	4.4	231	6.3	0.118	26.8	33.5
0.82	0.69	0	27	0	26	0	2011.3	4.6	232	5.7	0.277	26.1	38.5
0.84	0.79	0	25	0	28.5	0	2018.5	4.4	182	6.9	0.099	20	35.9
1.04	1.00	0	29	0	28.5	0	1182.2	4.8	172	5.9	1.369	32.3	30
0.94	0.83	0	28	0	26.5	2	1589.5	4.5	180	5.7	<0.012	25.9	66.7
1.24	0.86	0	28	0	28	0	2470.9	4.4	218	6	0.094	30.7	31.2
0.88	0.80	0	19	1	29	0	1599	4.3	208	6.5	0.261	23.3	23.9
0.92	0.84	0	27	0	25	3	2139.1	4.5	178	6	0.229	17.5	56.7
0.90	0.65	0	25	0	24	4	2207.7	4.7	179	6.6	0.155	19.6	41.5
1.23	0.99	0	26	1	28.5	0	2951.2	4.3	220	5.9	0.028	21.8	46.6
1.47	0.91	0	21	0	27.5	2	6012.4	4.6	118	12	0.195	22.2	11.6
1.17	0.90	0	28	1	25	3	1291.2	4.4	158	5.8	0.17	24.4	40.9
0.95	0.89	0	28	2	27.5	0	2038.2	4.1	143	5.7	0.061	20.8	32.6
1.01	0.89	0	26	0	28.5	1	1901.6	4.2	280	7.6	0.095	21.7	18.1
1.17		0	29	4	25.5	2	1868.2	4.6	144	5.9	0.351	22.9	52.2
0.75	0.59	0	22	3	23.5	2	1198.9	4.5	225	6	0.046	20.2	106.2
1.08	0.97	0	30	0	29	1	2381	4.7	193	5.8	0.035	23.2	30.3
1.00	0.91	0	28	1	27.5	0	2543.2	4.5	201	6	0.12	17	34.3
0.91	0.75	0	20	0	26.5	1	1238.9	4.9	175	7.5	0.308	21.5	57.8
1.29	0.86	0	30	0	28.5	2	1721.6	4.1	156	6.2	0.082	21.2	28.7
0.80	0.73	0	13	1	27.5	4	939.28	4.7	155	5.5	0.024	22.6	50.3
0.99	0.77	0	24	2	25.5	2	3136.1	4.2	130	6.1	0.17	25.9	64.9
0.67	0.68	0	21	0	27	0	1029	4.6	202	5.6	0.034	18	36.3
0.81	0.65	0	24	0	25	0	2271.4	4.4	196	5.9	0.024	17.5	18.7
0.76	0.80	0	24	1	28	2	2348.5	4.3	241	5.7	0.09	29	54.2
1.22	1.17	0	23	1	26.5	4	700.77	4.4	156	6.9	1.232	30.2	55.3
0.91	0.87	0	22	0	27.5	0	1151	3.9	163	5.8	0.139	18.8	25
1.37	1.11	0	28	0	27	0	2110.4	4.5	208	5.8	0.074	28.5	36.1
0.65	0.63	0	18	2	28.5	2	1312.4	4.4	187	6.4	0.021	15.5	19.4
0.97	0.94	0	23	4	27.5	0	2198.9	4.5	207	6.3	0.106	18	25.1
1.04	0.93	0	28	0	26.5	1	2312.6	4.5	215	6.9	0.134	16	70.3
0.93	0.90	0	22	1	28	0	1421.3	4.4	226	6	0.152	19	50.8
0.97	0.83	0	23	0	26	1	2846.6	4.4	243	5.9	<0.012	19.9	49.7
0.96	0.87	0	16	0	25.5	2	1431	4.2	200	6	0.052	11.9	89.3
1.22	0.91	0	26	2	30	1	1811.2	4	238	5.6	0.074	18.1	28.2
0.99	0.96	0	22	1	26.5	1	1983.2	4.4	152	5.5	0.256	27.7	35
0.86	0.85	0	21	0	26	1	908.18	4.2	155	5.8	<0.012	28.9	40.9
1.09	0.93	0	27	0	26.5	2	3937.2	4.3	167	6.1	0.082	18.6	99.4
1.25	1.11	0	27	0	28	0	2613.7	4.4	191	5.6	0.206	26.5	117.9
0.89	0.87	0	22	0	25.5	1	1918.1	4.6	190	6.5	<0.012	27.3	43.7
0.79	0.69	0	18	0	24.5	3	1430.5	4.3	183	5.8	0.249	23.9	72.4
0.70	0.47	0	24	1	26	3	1630	4.4	174	5.9	0.717	13.9	73.6
0.76	0.52	0	23	0	28	3	1262.3	4.4	188	5.7	0.12	24.9	48.6
0.85	0.80	0	26	0	30	1	1708.5	4.6	237	5.6	0.242	28.5	21.9
0.87	0.83	0	23	0	28	0	2158	4.5	233	6.5	0.111	12.3	55.2
1.29	0.98	0	24	1	27.5	1	1421.2	4.5	192	6.1	0.982	34.7	47.7
1.15	0.86	0	28	2	27	0	2289.8	4.7	201	6.5	0.176	22.9	46.7
1.17	0.90	0	28	0	26.5	0	1982.4	4.4	202	6.3	0.699	17.9	26
1.05	0.78	0	28	0	28	0	1550.8	4.5	204	5.2	0.333	29.3	37.1
1.12	0.98	0	29	2	24.5	0	2913.4	5	204	5.6	0.117	33.7	35.6
0.89	0.72	0	29	3	27	0	1960.4	5.2	225	5.7	<0.012	28.7	32.3
1.11	0.96	0	26	1	28.5	0	1741.9	4.6	195	5.7	0.132	30.5	39.6
0.92	0.88	0	25	0	24	4	1409.2	4.9	184	5.2	0.094	22.6	31.1
0.98	0.73	0	26	0	27	3	1877.8	4.8	255	6.1	0.076	16.1	29.6
1.17	1.00	0	28	1	27	0	2182.6	4.6	181	5.6	0.251	28.7	51.4
0.81	0.73	0	26	0	27.5	2	1809.4	4.7	250	5.8	0.271	24.8	34.2
1.16	1.10	0	26	0	28.5	2	1148.6	4.3	177	5	0.037	29.3	30.5
1.18	0.84	0	27	1	25.5	1	1449.2	4.5	185	4.9	0.084	25.8	34
1.06	0.92	0	28	1	28	0	1710.5	4.7	242	5.5	0.035	10.2	34
1.06	0.81	-4	14	1	27	2	1891.2	4.8	181	6.8	<0.012	26.1	25.2
1.33	1.17	0	28	1	28	1	1881.3	4.8	257	5.5	0.03	23.8	22.9
1.14	0.92	0	28	2	27	1	1521	4	189	7.9	0.274	17	29.1
0.96	0.82	0	25	0	28	1	1687	4.3	123	6.8	<0.012	29.5	26.5
1.01	0.81	0	27	1	25.5	1	1318.6	4.5	166	5.1	<0.012	25.6	46.1
1.08	1.02	0	27	2	28	0	1988.4	4.6	198	6.1	0.085	27.9	44.8
0.77	0.80	0	28	0	28	1	1466.9	4.6	195	5.8	0.245	20.2	41
0.78	0.57	0	26	1	28	0	1319.7	5	251	4.9	0.093	23.3	35.7
0.91	0.84	0	29	0	27.5	0	2009.1	4.9	231	5.9	0.111	24	22.8
0.80	0.72	0	25	1	26	3	996.66	4.2	217	6.5	0.214	16	126

1.47	0.97	0	30	1	27.5	0	3546.3	4.5	234	5.4	0.505	18.8	63.9
1.04	0.92	0	28	1	28	1	1869.1	4.5	222	5.6	0.054	19.1	35.5
1.11	0.73	0	21	0	28.5	3	2201.8	4.4	210	5.6	0.307	25.1	32.5
0.79	0.66	0	19	1	27	4	1721	4.4	187	6	0.342	17	55.8
1.69	1.16	0	27	1	29	1	1698.9	4.6	187	5.6	0.078	22.7	27.7
0.87	0.79	0	26	1	26	4	1611	4.2	210	8.7	0.072	28.5	52
0.97	0.80	0	25	0	27.5	2	2057.6	4.7	231	5.8	0.134	19.2	28.9
1.32	0.89	0	29	2	28	3	2216.5	4.5	205	6.4	0.042	19.5	33.3
1.09	0.89	0	26	0	26.5	2	3337.2	4.7	186	6.5	0.798	22.1	41.1
0.80	0.75	0	27	1	24.5	1	1189.8	4.5	151	4.5	<0.012	32	31.2
0.75	0.64	0	14	0	27	2	1799.5	4.2	202	6.1	0.172	34.3	101.9
0.92	0.76	0	20	2	25.5	3	1560	4.8	259	5.4	0.032	23.7	47
1.19	1.03	0	28	0	29.5	0	1689.3	4.6	238	6.5	0.115	20	57.4
0.90	0.87	0	14	0	30	2	1352.8	4.5	262	5.6	0.411	30.6	50
1.15	0.82	0	29	0	28.5	1	1461.2	4.5	186	6.3	0.774	23	44.1
0.94	0.79	0	26	0	26	2	911.25	4.4	143	6.2	1.704	19	57.4
1.23	0.91	0	27	0	28	0	1179.1	4.8	246	5.5	0.026	21.6	26.2
0.94	0.87	0	23	3	28	0	2087.5	4.6	216	5.6	0.157	18.3	31.2
0.98	0.75	0	27	4	26.5	0	2268.5	4.6	194	5.6	0.064	14	30.9
1.37	1.22	0	24	15	25	1	2578.5	4.6	204	5.6	0.062	21.6	46.2
1.04	0.87	0	21	0	25.5	2	1949.1	4.2	161	5.5	0.692	11.8	52.1
0.82	0.61	0	28	8	25.5	1	1658	4.5	201	5.9	0.377	16.2	49.7
1.04	0.76	0	29	1	25.5	0	1229.8	4.6	220	6	0.099	20.3	54.3
1.12	0.93	0	28	0	29	1	1850.5	4.8	170	6.4	0.149	18.3	45.7
1.15	0.82	0	29	2	28.5	0	1331.6	4.4	183	5.2	<0.012	14.6	39.9
0.78	0.58	0	29	3	24.5	3	2099.2	5.1	177	15.4	0.359	31.6	22.9
1.08	0.95	0	25	0	27.5	0	1737.3	4.5	240	6.1	0.09	14.7	34.3
1.24	0.83	0	25	0	28.5	0	979.8	4.3	210	5.6	0.046	20.6	37.1
0.93	0.75	0	28	2	25.5	0	2660.8	4.7	260	5.4	0.042	20.6	23.1
0.98	0.67	0	28	1	23.5	2	570	5	232	5.9	2.464	11.9	91.3
1.07	0.84	0	29	1	27.5	1	2069.4	4.8	249	5.8	0.037	15.5	53.6
0.90	0.75	0	27	0	29	2	1361.6	4.9	166	5.7	0.2	17.3	32.8
1.09	0.99	0	28	0	27	3	2477.5	4.6	213	6.6	0.41	16.2	27.5
1.01	0.91	0	29	0	29.5	1	2661.7	4.2	199	5.5	0.103	19.8	38.3
1.25	0.98	0	27	0	28	0	1787	4.7	211	5.9	0.022	18.1	56
1.16	1.15	0	30	1	29	0	2272	4.8	194	5.9	0.282	23.7	33.4
1.15	0.82	0	27	0	27	0	1640	4.7	239	5.6	<0.012	19.4	34.7
1.37	1.08	0	26	0	27.5	0	1330.3	4.9	228	5.6	<0.012	12.7	23
1.39	1.09	0	27	0	28	0	1628.6	4.5	275	5.8	0.07	26.5	26
0.99	0.76	0	27	0	26.5	3	1437.5	4.3	119	7.8	<0.012	21.2	56.1
1.31	1.05	0	30	0	25.5	1	1561.6	4.7	167	5.2	0.285	23.9	58.3
1.00	1.07	0	28	0	27	1	2252.9	4.7	164	5.1	0.057	18.6	46.2
1.11	1.06	0	25	1	28.5	0	2799.1	5.1	255	5.8	0.049	19.2	51.4
0.98	0.86	0	30	0	27.5	0	2267.3	4.7	224	5	<0.012	26.6	30.1
0.90	0.77	0	28	1	26.5	0	1639.2	4.7	184	5.8	0.07	13.7	54.1
0.80	0.79	0	30	0	29	0	1478.9	4.5	186	5.7	<0.012	21.2	50
0.89	0.71	0	22	2	28	0	3299	4.4	217	5.8	0.343	15.4	61.4
1.05	0.84	0	29	0	27	0	2338.9	4.9	292	5.7	0.052	25.1	30.5
0.96	0.80	0	28	3	26.5	0	2598.8	4.5	241	5.5	0.047	14.3	29.8
0.94	0.85	0	18	2	27	1	2220.7	4.6	158	7.1	0.144	21.4	32.9
0.98	0.90	0	29	0	27.5	1	1241.7	4.6	195	5.9	0.039	15.9	47.5
0.85	0.72	0	19	0	27	0	1698.2	4.8	255	5.5	<0.012	13.3	46.9
0.92	0.87	0	26	0	27.5	1	1193.6	4.5	154	9.3	0.345	22.5	47.9
1.02	0.94	0	27	0	29.5	0	2501.9	4.7	303	6.2	0.118	19	43.6
0.89	0.95	0	26	0	27	0	2237	4.7	159	5.6	0.149	17.8	59.8
0.75	0.68	0	26	0	27.5	2	2378.5	5	194	6.1	0.288	25.2	19.9
1.14	0.83	0	27	0	25.5	0	1380	4.5	222	5.8	0.074	22.3	49.1
1.06	0.99	0	30	0	29.5	0	1652.4	4.2	172	6.6	0.124	22.5	78.3
1.02	0.86	0	30	0	27	1	1829.6	4.8	182	6	<0.012	21.5	42.4
1.17	1.07	0	30	0	24	0	1492.7	4.5	182	5.5	0.089	21.1	40.6
1.07	0.87	0	27	4	26.5	0	1648	4.7	217	5.8	0.286	11.6	35.1
1.09	0.97	0	29	0	25	0	1469.2	4.5	234	5.3	0.013	31.4	39.1
1.47	0.87	-0.5	24	3	27.5	2	1687.8	4.3	196	7.1	0.018	17.9	35.4
1.24	0.94	0	28	0	26.5	0	1587.9	4.9	203	5.8	<0.012	20.3	47.7
1.02	0.98	0	28	0	27.5	0	2332.3	4.5	183	5.6	0.014	22.7	29
0.99	0.73	0	27	0	27.5	1	1768.8	4.7	161	5.1	0.169	15.7	40.1
0.95	0.90	0	29	8	26	5	969.54	3.4	105	6.7	0.058	16.6	24.4
0.96	0.71	0	28	1	26.5	2	2111.7	4.5	278	5.7	0.2	14.3	91.6
0.91	0.89	0	30	0	28	2	1571.8	4.8	255	9.5	0.191	23.8	27.3
0.93	0.61	0	29	1	25.5	2	940.8	4.5	171	5.8	0.325	17.9	53.2
1.03	0.99	0	30	1	29.5	0	2031.3	4.6	255	5.7	0.127	22.8	34.4
0.81	0.71	0	27	0	26.5	0	2151.4	5	207	5.8	0.054	26.3	24

0.76	0.68	0	25	0	25.5	2	2191	4.6	200	6.1	0.203	19.2	33
0.93	0.79	0	30	0	29	0	2475.6	4.4	195	5.7	1.164	18.4	37.8
1.09	0.79	0	29	0	28.5	0	1529.3	4.5	198	5.6	0.17	17.3	31.4
0.83	0.78	0	25	0	26	1	1990.4	4.6	240	6.2	0.02	24.8	41.9
0.98	0.89	0	28	4	29	0	1531.1	4.5	173	5.2	0.159	18.1	41.2
1.52	0.95	0	27	0	28	1	1936.3	4.4	206	6.8	0.128	18.7	16.2
1.00	0.97	0	28	0	28.5	0	1482	4.7	201	4.9	0.106	19.5	25.1
0.83	0.81	0	29	1	25	1	1770.3	4.3	139	5.3	0.086	15.3	61.7
1.11	0.85	0	27	0	26.5	0	1541.3	4.6	155	5.4	1.842	26.1	36.9
0.88	0.71	0	17	1	26.5	0	2118.3	4.9	273	6.7	0.025	20.8	23.1
1.11	0.92	0	19	14	28.5	2	1668.6	4.7	320	5.4	0.054	23.3	26.1
		0	27	0	27.5	0	2272.7	4.5	192	5.8	0.027	15.5	42.1
0.82	0.60	0	17	1	26.5	2	1619.8	4.7	234	5.3	0.272	19.8	37.4
1.07	0.93	0	30	0	29	2	1288	4.8	203	4.4	0.041	23.7	38.4
0.91	0.69	0	26	0	26	3	1470.6	4.9	177	9.4	0.029	28.5	60.2
		0	17	0	28	0	1658.6	4.6	178	6.3	0.082	18.4	50.1
1.14	0.96	0	26	1	26.5	1	1858.5	4.9	217	6	0.138	20.8	36.4
0.87	0.73	0	28	1	25.5	2	1428.6	4.4	206	5.8	0.021	10.7	43.8
0.98	0.82	0	26	0	29	0	2357.6	4.6	235	5.9	0.013	14.6	49.7
0.75	0.49	0	15	0	24.5	2	1681.4	4.3	175	4.7	0.705	39.2	26.4
0.83	0.95	0	24	0	24	0	1358.5	4.5	178	5.2	0.028	22.2	34.3
1.07	0.70	0	20	0	25	0	1328.5	4.5	263	5.8	0.142	20.7	15.1
0.83	0.70	0	19	1	29	3	1471.7	4.4	181	6.9	0.064	17.4	71.5
0.76	0.66	0	17	2	26	2	2670.7	4.6	178	7.9	0.039	22.2	66.9
1.02	0.82	0	27	1	29	0	2937.6	4.3	166	6.3	0.091	17.1	34.9
0.76	0.64	0	23	1	26.5	4	1711	4.6	130	7.5	1.389	11.3	48.7
1.02	0.84	0	19	2	27	4	2277.2	4.6	217	8.2	0.335	12.8	68.6
1.24	0.88	0	28	0	26	2	1658.7	4.1	213	6.4	0.15	16.4	63.8
1.09	0.75	0	28	0	25.5	0	1681.7	4.9	235	5.7	0.161	10.9	66.8
1.29	0.95	0	24	0	27	3	1820.8	4.8	138	6.2	0.314	29.1	5.8
1.13	1.15	0	27	3	27.5	1	3639.7	4.9	196	5.7	0.073	20.9	11.8
0.97	0.98	0	19	8	28.5	0	2079	5.1	166	6	<0.012	17.7	46.1
1.36	1.17	0	29	0	29.5	1	3060.5	4.7	169	6.9	0.33	19.3	93.7
0.89	0.81	0	25	1	27	1	2207.5	5.2	198	5.6	0.24	25.6	55.7
0.79	0.63	0	24	4	24.5	1	1787.9	5.1	159	6.5	0.056	11.9	25.4
1.25	0.94	0	28	0	28.5	0	1691.6	4.3	200	5.2	0.043	20.1	58.5
1.18	0.79	0	28	0	28.5	1	1919.8	4.5	184	6.2	0.056	33	37.5
1.20	0.82	0	21	0	24.5	2	2497	4.9	190	6.1	0.029	18.7	62.3
0.87	0.79	0	24	1	24.5	2	2646.8	4.9	182	5.8	0.393	15.1	46.9
1.08	0.84	0	29	1	25	0	2148.3	4.6	209	6.1	0.199	23.7	35
1.07	1.03	0	28	1	27	0	1390.6	4.6	203	6.3	0.017	33.1	54.7
1.14	0.92	0	28	1	26.5	0	1011	4.5	221	4.7	<0.012	26.1	45.2
1.13	0.90	0	17	0	25.5	0	1155.8	4.7	163	5.7	4.004	27.1	24.3
1.08	0.90	0	29	0	27	1	2088.9	4.9	172	5.8	0.035	23.2	42.8
1.18	1.02	0	27	1	27	1	2731.4	4.7	188	6.1	0.016	24.2	34.7
1.03	0.87	0	27	0	28	1	2028.4	4.3	182	5.8	0.164	22.7	46.8
0.77	0.71	0	30	1	29.5	1	1870.4	4.4	187	5.6	0.028	20	50.8
1.07	0.86	0	30	0	28.5	0	1581.2	4.4	211	5.9	0.066	17.9	43.5
1.13	0.86	0	28	0	26.5	2	4765.2	4.7	227	5.5	<0.012	18.8	14.5
1.21	0.97	0	26	0	27.5	0	2169.1	4.7	217	5.8	0.048	16.3	71
0.76	0.63	0	19	1	28.5	0	1387.6	4.6	194	5.5	0.013	25.3	37.1
1.01	0.83	0	28	1	27	0	2221.4	4.8	221	5.3	0.014	22	46.8
1.27	0.95	0	24	0	30	0	1800.8	4.4	200	5.7	1.237	41.3	43.6
1.26	0.89	0	24	1	27	0	1620.5	4.5	162	6	0.909	19.5	87.4
0.81	0.79	0	20	1	27.5	1	2088.6	4.9	183	5.9	0.026	21.9	36.2
0.71	0.67	0	18	7	28	2	1329.3	4.5	162	6.8	0.018	17.7	50.4
1.08	0.84	0	20	1	25	0	2380.5	4.2	184	5.6	0.051	22.7	66
0.93	0.75	-1.5	25	15	30	1	1629.6	4.8	209	6	0.078	23	26.6
1.02	1.06	0	29	0	28.5	1	2441.7	4.6	184	5.5	0.019	25.9	31.4
1.42	1.05	0	27	2	27	0	2630.9	4.3	223	6	0.042	21.2	25.9
1.08	0.98	0	26	2	26	0	1818	5	179	5.6	0.085	35.1	41.4
1.18	0.94	0	27	1	29	0	2367.3	4.5	205	6.2	0.023	21	26.9
1.08	0.90	0	30	0	29.5	0	1280.6	4.6	310	6.2	<0.012	18	46.3
1.13	0.92	0	26	0	28	0	1509.7	5	218	5.6	0.034	16.8	34.6
1.00	0.87	0	28	0	28.5	0	2301.4	4.7	181	6.4	0.03	19.8	38.6
0.87	0.73	0	29	1	27	2	1691	5.2	234	5.6	0.087	24.4	28.6
1.01	0.85	0	21	1	27.5	0	2268.9	4.8	252	5.5	0.036	19.2	35.1
1.14	0.96	0	27	0	29.5	0	2342.3	4.6	233	6.1	0.174	22.8	65
0.76	0.66	0	15	0	27.5	0	2369.4	4.9	230	5.8	0.075	15.9	52.6
1.00	0.93	0	26	1	26	1	2528.6	5.1	145	6.6	0.013	26.8	23.1
1.03	0.83	0	26	0	28	0	2137.4	4.7	188	5.6	0.031	25.3	18.8
0.86	0.74	0	25	4	26	0	2268	5	205	5.9	0.043	19.7	65.5

0.91	0.79	0	18	2	29.5	0	2799.1	4.8	215	5.8	0.086	19.3	32
1.20	1.08	0	27	0	27.5	0	3768	4.9	242	6.2	0.04	21.4	34.2
1.14	0.90	0	30	1	30	0	2152.2	4.6	239	5.6	<0.012	15.6	20.6
0.75	0.63	0	30	1	26	0	2409.8	4.5	151	5.6	0.127	24.1	200.6
1.00	1.00	0	29	1	27.5	1	1942.2	4.6	173	6.6	0.511	24.1	37.9
0.99	0.93	0	23	1	29.5	0	2600.5	4.7	242	5.4	0.094	28.6	34
1.05	0.94	0	30	0	27.5	0	2668.5	4.7	278	6.4	0.032	23.3	26.2
0.97	1.04	0	27	1	27	2	2177.1	4.8	212	5.7	0.106	43.9	32.7
1.05	0.95	0	30	0	28.5	1	1690.4	4.6	163	6.8	0.087	20.6	34.9
0.78	0.60	0	18	2	27	2	1469.6	4.2	245	6.4	0.038	15.9	117.4
1.07	0.92	0	26	0	28.5	0	1931.9	4.4	209	5.4	0.082	15.1	36.9
	0.88	0	22	0	27	2	1998	4.4	231	6	0.033	23.1	45.6
0.78	0.72	0	14	0	27	3	1850.7	4.3	208	10.5	0.16	14.2	55.5
1.06	1.05	0	26	2	29.5	0	1830.6	4.9	292	6	0.083	16.4	48.6
0.77	0.65	0	28	1	27.5	0	1717.2	4.8	175	5.8	0.105	12.8	28.3
1.09	0.81	0	30	0	29.5	0	2248	4.8	196	6	0.107	23.5	30.8
1.07	0.97	0	29	0	28.5	0	2489.8	4.5	242	5.6	0.018	22.6	46.7
1.06	0.85	-1	28	0	28.5	0	1541	4.8	226	5.5	0.046	15.9	71.3
1.19	1.13	0	28	0	29	0	1798.2	4.5	192	5.2	<0.012	15	46.9
1.17	0.86	0	28	2	27	0	1159.6	4.6	203	5.9	0.063	16.2	23.7
0.94	0.73	0	27	0	27	0	1770.1	4.5	190	5.3	1.154	21.9	48.4
0.73	0.70	0	22	1	24	3	1708.6	4.4	197	7.6	0.405	25.7	45.6
1.11	1.15	0	26	0	28.5	1	1968.6	4.3	169	6.9	0.185	9.5	82
0.89	0.81	0	25	4	29	0	2030.8	4.6	214	6.2	0.044	19.3	41.5
0.89	0.81	0	20	0	26	2	1638.2	4.4	220	5.8	0.102	17.9	44.7
0.93	0.63	0	24	0	27	3	1550.9	4.7	167	7.1	0.029	27.2	25.1
0.99	0.75	0	25	1	27	0	2450.2	5	230	6.2	0.336	14.7	41.3
0.99	0.83	0	28	2	25	4	1631.5	4.5	269	5.8	<0.012	17.4	25.8
1.03	0.87	0	30	6	27.5	0	1590.5	4.8	181	5.9	0.374	22.4	35.4
0.91	0.70	0	28	4	26	2	1598.3	4.8	240	5.7	0.04	21.8	28.9
		0	30	0	27.5	0	2111.2	4.5	213	6	0.663	21.7	27.7
0.94	0.87	0	29	1	27	0	1969.5	4.8	226	5.7	<0.012	21.2	39.3
1.27	0.85	0	16	0	28	2	1041.8	4.8	166	5.7	0.061	20.9	49.9
1.36	1.03	0	28	0	29	0	2378	4.6	142	6.1	1.139	24.4	44
1.06	0.91	0	28	0	27	0	1948.2	4.5	174	5.5	0.095	21.8	32.5
0.76	0.74	0	25	1	29.5	0	1421	4.5	152	5.7	<0.012	28	64.9
0.94	0.83	0	27	0	28	0	2201.7	4.5	277	5.9	0.604	23.3	38.9
1.03	1.05	0	27	0	28	0	2542.9	4.8	252	6.1	0.383	11.9	43.3
1.12	1.09	0	24	0	27.5	0	2488.6	4.8	226	5.6	0.036	17.5	58.3
1.39	1.06	0	29	0	28.5	1	2556.4	4.4	243	6.9	0.11	17.1	25.5
0.98	0.78	0	19	1	27	1	1592.8	4.8	159	9.5	0.108	23.9	34.1
1.04	0.93	0	28	2	25	0	1360.2	4.4	129	5.9	0.278	15.9	66.8
0.96	0.84	0	29	0	28	0	1891.3	4.7	179	6.2	<0.012	22.3	34.7
1.09	1.03	0	27	0	28.5	1	1971.2	4.7	197	5.5	0.287	39.4	44.4
0.87	0.79	0	30	0	30	0	1469.3	4.5	196	5.7	0.07	21.9	43.7
1.10	0.85	0	30	0	27.5	0	2341.2	4.8	195	6.4	0.112	31.1	40.3
1.08	0.67	0	29	0	28	0	1209.3	4.5	219	5.8	0.018	24.2	24.1
1.20	0.89	0	27	0	27.5	0	3710.4	4.9	181	5.6	0.303	24.1	36.2
1.22	0.99	0	29	1	29	0	1589.2	4.7	209	5.4	0.016	17.1	39.7
1.23	0.89	0	25	0	28	1	528.64	4.5	190	8.4	2.523	13.8	51.7
0.96	1.03	0	28	0	30	0	1962.1	4.6	180	5.8	0.015	19.9	60.9
1.24	1.05	0	27	0	28.5	0	2227.8	4.3	191	6.2	0.086	26.4	45.5
1.07	0.85	0	30	0	27.5	0	2102.6	4.3	159	5.4	<0.012	22.3	21.9
1.11	0.91	0	30	2	29.5	0	1838.3	4.9	203	5.9	0.079	14.4	37.3
1.01	0.97	0	30	1	28	0	1988	4.4	214	5.4	<0.012	19.9	46.1
1.09	0.96	0	29	9	26	0	1661.6	4.1	184	5.8	0.022	22.6	68.8
1.04	0.77	0	27	0	27	0	1610.5	4.6	209	5.7	0.317	20	53.4
0.77	0.57	0	26	0	25.5	2	2177.8	4.5	210	5.7	0.945	19.8	37.3
1.18	0.91	-1	24	7	26	2	1818	4.4	146	6	0.708	27.1	31.4
1.18	0.93	0	22	0	28	0	1542.3	4.5	155	5.3	0.044	29.2	55.1
1.00	0.84	0	27	4	26.5	0	1588.7	4.7	164	4.1	0.189	22.2	11.1
1.53	1.12	0	25	0	28.5	2	1980.3	4.8	162	6.6	0.027	36.8	23.3
1.52	1.06	0	30	2	29.5	2	1371.9	4.8	136	7.6	0.096	28.3	30.7
1.12	0.92	0	30	2	25.5	0	1487.7	4.7	181	5.2	1.861	23.3	23.7
1.13	0.92	0	29	0	27.5	0	2870.4	4.7	138	6.2	0.851	37.4	31.3
1.01	0.88	0	25	1	30	0	1717	4.5	215	6	0.025	16	27.8
0.98	0.82	0	17	2	25	2	1499	4.3	218	5.9	<0.012	19.6	35.5
0.70	0.63	0	26	2	28	3	2361.6	4.3	166	7.1	0.124	19.5	38.9
1.08	0.76	0	20	1	26.5	1	2271.2	5	179	6.3	0.157	19.5	26.2
1.09	0.97	0	28	0	27	1	1981.5	4.5	252	9.4	0.056	16.6	24.7
0.93	0.83	0	25	2	27	0	1441.8	4.7	195	5.6	<0.012	23.8	20.8
0.83	0.83	0	25	0	28	0	3271.5	4.9	174	5.9	0.04	25.6	47.5

1.03	0.87	0	30	0	28	1	1998.8	4.9	155	5.1	0.019	7.2	19.9
1.01	0.80	0	28	0	29.5	1	1589	4.7	216	5.4	0.044	14.2	37.2
0.92	0.93	0	29	4	28	0	1952.2	4.6	200	5.7	0.081	22.3	39.1
1.03	0.79	0	22	2	27	0	1828.5	4.5	164	6.3	0.214	21	25.5
0.91	0.95	0	29	2	27	0	1667.4	4.6	134	5.6	0.099	30.3	32.8
1.18	1.08	0	27	4	26.5	0	2178.8	4.4	160	5.4	0.45	39.5	42.2
1.10	0.93	0	27	0	27.5	0	2716.6	4.7	240	6.2	0.49	12.8	31.8
1.16	1.01	0	23	1	28.5	0	1692.1	4.7	212	5.7	0.188	22.6	31.2
0.86	1.00	0	28	4	25.5	2	2368.4	4.2	174	5.5	0.398	20.8	22.9
1.01	0.72	0	25	1	29.5	0	1640.1	4	202	5.8	2.566	27.5	36.9
1.32	0.92	0	23	0	28	0	1732	4.4	199	5.5	0.076	16.5	64.6
1.02	0.85	0	24	5	26.5	0	1840.5	4.3	218	6.1	0.093	21.2	41.2
0.85	0.76	0	26	14	24.5	0	1291.7	4.7	205	5.7	<0.012	15	31.9
1.15	0.84	0	29	1	28	0	1491.1	4.8	257	5.6	<0.012	20.4	22.6
1.10	0.99	0	23	3	24.5	1	1070	4.1	163	4.9	<0.012	14.9	31.2
0.95	0.84	0	22	0	28.5	2	1749.2	4.3	211	7.8	0.04	27.7	33.5
0.89	1.05	0	29	1	28.5	1	2180.6	4.4	193	5.5	0.045	21.9	40
1.00	0.91	0	29	1	26.5	0	1730.4	4.3	160	5.7	0.024	29.3	39.8
0.94	0.70	0	27	11	20.5	2	419.52	4.4	113	5.7	0.078	19.5	39.7
0.97	0.91	0	27	0	28	0	1820.4	4.3	234	6.4	0.088	29.6	48.5
0.92	0.91	0	30	0	28	0	2051.9	4.5	198	5.4	0.034	21	42.5
1.08	0.85	0	30	0	29.5	0	1399.4	4.6	181	5.6	<0.012	23.6	71.6
1.10	0.95	0	27	0	29	0	1891.1	4.6	260	5.5	0.813	23.6	46.8
1.26	0.83	0	23	1	29.5	2	1167.6	4.6	169	5.2	0.084	26.1	50.4
1.11	0.86	0	26	0	29	1	2980.5	4.9	230	6	0.044	23	23
0.87	0.61	0	20	1	27	0	2292	4.4	146	5.7	0.402	15.5	31.1
0.90	0.86	0	25	0	25.5	2	2722.1	4.5	162	6.4	0.086	13.5	31.4
0.82	0.69	0	30	0	25.5	0	1451.4	4.7	146	5.2	0.017	4.8	44.8
1.21	1.08	0	25	1	30	0	1230.1	4.3	174	5.4	<0.012	23.6	26.6
0.98	0.90	0	24	0	27.5	0	1952	4.8	249	6.3	0.751	15.8	69.5
1.12	0.93	0	30	0	29.5	0	1891.3	4.8	286	5.9	0.116	21.1	80.1
1.24	1.07	0	29	0	29	0	1848.2	4.5	210	5.8	<0.012	30.6	31.6
0.95	0.96	0	27	1	29	0	1920.3	4.5	223	5.6	<0.012	21.1	29.9
1.11	0.77	0	28	0	29.5	0	1527.9	4.1	199	5.7	0.037	21.5	46.3
1.15	1.09	0	30	0	28.5	0	2432	4.7	163	5.9	0.063	15.3	64.3
1.11	0.93	0	27	2	27	0	2342.5	4.7	238	5.1	0.036	23.2	37.1
1.53	1.21	0	27	0	28.5	0	1458.2	5.2	241	5.1	0.196	27.3	40.7
1.01	0.64	-7	10	1	25	3	1551	4.5	192	5.5	0.224	13.2	74.2
1.26	1.00	0	28	1	28	1	2326.3	4.4	235	5.3	0.031	14.1	50
1.14	1.14	0	30	0	28.5	0	1892.4	4.2	169	5.5	0.124	24.4	19.9
0.82	0.74	0	30	0	28.5	1	2870.4	5.5	288	6.4	0.336	10.5	60.1
1.15	0.97	0	30	0	30	0	2498.4	4.7	208	5.7	0.08	21.5	28.1
0.93	0.64	0	30	3	28.5	1	3121.6	4.6	205	5.8	0.416	21.5	38.2
0.93	0.79	0	26	1	29.5	0	2232	4.5	282	5.6	0.101	19.1	62.2
1.13	0.92	0	21	1	29	2	2462.5	4.8	254	5.7	0.093	18.7	43.4
1.11	0.80	0	13	1	28.5	0	4161.7	4.4	182	5.7	0.447	23	35.8
1.21	1.13	0	30	0	29	1	3290	4.7	262	6	0.356	23.7	27.8
1.00	0.83	0	29	2	25.5	3	1720.3	4.5	196	5.6	<0.012	26.2	32.8
1.48	1.04	0	30	0	28	0	1730.4	5	198	5.5	<0.012	18.7	41.4
1.04	0.70	0	30	2	28.5	1	1450.4	4.2	202	6.1	0.051	23.9	40.1
1.08	0.91	0	30	0	27	1	1801.2	4.7	177	5.3	<0.012	28.2	19.1
1.32	0.99	0	30	2	29.5	1	1971	4.7	152	6.1	0.032	39.2	31.3
1.09	0.77	0	27	1	29	1	2386.8	4.6	219	6.6	0.139	21.6	43.7
1.13	0.99	0	29	1	27	0	1547.9	4.2	216	5.5	0.181	19.7	29.3
0.92	0.80	0	18	4	26	1	1728.1	4.7	139	6.5	0.212	19.4	47.7
0.82	0.83	0	20	8	26	2	1430.8	4.2	153	5.9	0.481	22.1	42.9
1.00	1.01	0	25	0	27.5	2	1368.4	4.3	193	9.6	0.283	27.8	18.1
1.01	0.89	0	21	0	26.5	3	1401.1	4.4	218	5.4	0.065	25.2	27.1
0.85	0.87	0	27	2	27	1	2646.6	4.5	234	9.5	0.019	17.4	30.3
1.27	1.04	0	26	1	29	0	3349.1	4.6	202	6.2	0.069	20.6	29.4
1.03	0.89	0	28	1	28.5	1	1427.5	4.4	179	5.8	0.074	30.1	46.2
1.28	1.05	0	29	0	29.5	0	2727.5	4.2	191	5.7	0.454	19.8	44.1
1.25	0.96	0	28	1	30	0	1462.2	4.2	201	5.9	0.152	23.3	33.3
1.05	1.03	0	28	0	28.5	0	2821.9	4.7	195	5.3	0.017	22.4	30.8
1.20	1.19	0	29	0	28.5	0	2069.8	4.6	191	5	<0.012	21	21.7
1.18	0.90	0	27	1	29.5	0	2482.2	4.9	174	5.5	0.189	15.2	21
1.17	0.86	0	28	17	28	0	3220.6	4.6	201	5.3	0.202	21.7	45.7
1.26	0.96	0	28	1	28	1	3183.4	4.6	256	5.8	0.018	12.7	43.7
1.16	0.81	0	30	0	28	0	1258.6	4.3	192	5.4	<0.012	25.3	49.2
1.34	0.87	0	30	0	25.5	2	2889.9	4.7	283	5.2	0.019	15.7	30.9
1.01	0.72	0	29	5	26	2	1740.6	4.6	213	4.5	0.024	10.8	33.7
0.99	0.84	0	28	0	24.5	0	1501.6	4.5	219	5.3	0.988	14.6	54.9

1.09	0.99	0	21	1	26.5	3	1208.1	4.6	131	5.4	<0.012	17.5	56.2
1.26	1.02	0	28	0	28.5	3	2356.9	4.7	154	6.6	<0.012	16.8	29.7
1.00	0.80	0	28	1	29.5	0	2247	4.5	159	5.7	0.066	24.3	31.4
1.14	0.87	0	27	0	27	2	2247.5	4.5	232	5.1	0.054	19.8	40.4
1.13	0.91	0	30	0	28.5	3	1857.6	4.6	214	6.1	4.463	14.8	64.5
1.07	0.76	0	24	1	29.5	2	2158.7	4.5	195	5.3	0.02	20.5	30.2
1.00	0.81	0	26	2	26.5	1	1840	4.8	168	6.2	0.013	17.6	98.4
0.98	0.95	0	29	6	28.5	0	1350.5	4.9	166	5.7	0.054	21.3	41.4
0.81	0.91	0	29	2	27	2	2140.7	4.5	209	5.3	0.283	12.5	57.6
1.09	0.96	0	28	0	29	0	1369.7	4.5	195	5.2	0.155	24.3	44.7
1.33	0.91	0	29	1	28	1	1598.5	4.7	168	5	0.076	10.9	39.4
0.98	0.84	0	20	0	27.5	3	1882.3	4.8	198	6.3	0.047	20.5	18.5
1.03	0.87	0	24	0	29	1	1832.9	4.7	213	6.1	0.179	10.4	53.4
1.02	0.71	0	30	3	25.5	2	2122.2	4.7	196	5.9	<0.012	13	48.2
1.14	0.95	0	29	0	25.5	3	2539.5	4.7	132	7.4	0.131	15	23.3
0.95	0.72	0	27	2	29.5	0	1629.5	4.8	197	5.8	0.025	15.7	31.1
1.23	1.21	0	30	0	29	0	1853	4.6	174	4.6	0.018	17.8	52.1
0.78	0.66	0	16	1	24	4	1901.2	4.5	221	5.8	0.103	19.8	51.4
1.12	0.98	0	30	0	28.5	0	2012.4	4.9	233	5.5	0.154	12.3	38.7
0.82	0.59	0	30	0	25.5	2	2851.2	4.7	234	5.9	<0.012	17.6	88.6
1.06	0.76	0	30	0	29.5	0	1391.6	4.5	191	5.7	0.096	10.3	49.7
1.13	0.98	0	29	0	28.5	1	2327.3	4.7	197	6.5	0.23	16.8	41.1
1.06	0.83	0	29	0	25.5	2	1959.3	4.6	210	5.3	<0.012	20.9	35.4
0.84	0.82	0	29	0	29	0	1958.9	4.7	191	4.5	0.987	16.5	24.3
0.97	0.87	0	28	0	27	0	2072.6	4.4	139	5.7	0.191	24.4	47.2
1.00	0.92	0	29	0	28	0	1440.6	4.5	180	5.6	0.286	14.6	13.5
1.04	0.85	0	26	0	27.5	2	1681.4	4.6	170	5.5	0.192	28	47
0.96	0.84	0	28	0	27	0	1212.1	4.5	179	5.9	0.047	29.2	17
1.38	0.99	0	29	0	27	0	1867.6	4.4	214	5.6	0.096	19.9	87.6
0.88	0.86	0	29	1	27.5	0	3227.6	4.8	207	5.7	0.051	22.3	45.4
1.28	1.16	0	30	1	28	1	1901.5	5.1	232	7.2	0.046	16.6	20.8
1.18	1.14	0	28	0	28.5	0	3159.5	4.7	167	5	0.194	21.3	30.2
0.87	0.71	0	27	1	25	0	4219.6	4.7	218	6.2	<0.012	23.5	46
0.99	0.84	0	29	0	29	1	1501.8	4.8	177	6.1	0.146	21.9	50.6
1.00	0.81	0	26	1	29	1	1819.8	4.5	212	5.9	0.176	13.5	44.1
0.84	0.77	0	28	0	27.5	2	2318.4	4.6	162	7.3	0.503	15.8	30.9
1.09	0.81	0	25	0	27.5	0	1417.5	4.4	176	4.2	0.097	19.9	65.3
1.04	0.83	0	30	0	27.5	0	1241.2	4.5	195	5.8	0.016	10.7	41.8
1.03	0.78	0	30	0	27.5	2	1240.3	4.8	231	5.4	<0.012	18.1	20
0.93	0.81	0	29	0	29	1	2869.1	4.7	197	5.8	0.041	17.8	50.2
0.69	0.76	0	20	11	28.5	1	2369.7	4.5	271	11.5	0.311	12.6	34.1
1.20	1.16	0	30	0	28.5	0	2487.7	4.6	204	6.7	0.463	11.1	53.4
0.97	0.89	0	25	2	26.5	2	2052.5	4.5	231	5.7	0.037	25.1	32.8
0.96	0.88	0	27	0	29	0	869	4.3	246	5.4	<0.012	19.3	39.1
1.01	0.96	0	27	3	28.5	0	1782.2	4.3	159	5.9	0.049	25.6	27.4
0.96	0.65	0	26	0	27.5	0	1700.9	4.6	291	5.5	0.013	13.6	61.5
0.89	0.74	0	20	1	25.5	1	2527.6	4.8	257	9.6	0.062	25.6	23
1.01	0.83	0	26	1	27.5	0	1161.2	4.5	153	5.4	2.919	15.1	66.2
1.17	0.87	0	27	0	28	0	1459.1	4.5	182	6	0.024	9.4	46.1
0.70	0.66	0	21	2	28	0	1882.7	4.4	170	5.3	0.055	9.4	45
0.84	0.70	0	28	0	27	0	1860.9	4.8	235	6	0.058	16.2	20.3
1.53	0.99	0	29	2	27.5	0	2583.4	4.6	182	5.5	0.066	19.5	56.5
0.90	0.78	0	21	1	29	2	2018.6	4.8	268	6	0.038	16.6	75.3
1.08	0.83	0	30	0	28	0	1370.2	4.3	169	5.6	0.082	15.5	40.2
1.21	0.94	0	27	0	28	0	2927.9	4.3	235	5.9	0.138	16.2	43.8
1.01	0.91	0	30	0	28	0	1647.3	4.5	170	5.4	0.121	14.9	41.9
1.10	0.90	0	30	1	25	1	1318.1	4.8	207	5.2	0.196	9.3	39.3
0.85	0.80	0	19	0	28	2	2549.6	4.7	200	6.3	0.352	16.3	34.6
1.25	0.95	0	29	0	29.5	1	1958.7	4.5	216	6.2	0.041	27.8	64.1
1.27	0.91	-1	28	6	28.5	3	1290.8	4.4	152	7	0.16	19	36.8
1.62	1.06	0	28	1	29.5	0	1151.3	4.6	172	6.2	0.048	22.5	31.5
1.04	1.01	0	30	0	27.5	0	3256.8	4.5	370	6.4	1.224	17.7	63.3
0.99	0.79	0	29	0	28	1	3207.2	4.4	229	5.9	0.152	21.6	29.8
1.34	1.03	0	30	0	27.5	0	2337.9	4.7	185	5.5	0.141	29.5	39.9
0.93	0.78	0	24	1	26.5	0	1878.8	4.7	231	5.6	0.04	24.7	27.7
1.17	0.86	0	25	2	29.5	0	3081.3	4.8	101	5.6	0.112	19.6	20.2
1.17	0.93	0	24	2	27.5	0	1069.2	4.5	159	5.1	<0.012	4.8	71.8
1.21	1.04	0	29	1	28	0	2211.3	4.8	245	5.5	0.083	16.7	32.5
0.98	0.80	0	29	1	28	2	2387.1	4.4	173	5.1	0.02	16.9	26.7
1.04	0.86	0	27	1	27.5	0	1891.4	4.3	180	6.1	<0.012	18.2	60.2
0.82	0.67	0	26	1	29	2	1621.7	4.1	224	5.8	0.886	23.2	42.1
1.08	0.88	0	29	1	27	0	2157.8	4.5	131	5.5	0.092	15.8	59.2

0.87	0.87	0	24	2	25.5	0	2399.4	5	266	7.2	0.22	20.6	36.2
1.09	0.96	0	30	0	28	2	1647.2	4.2	205	6.1	0.206	27.8	38.3
1.10	0.92	0	29	1	29.5	0	3496.6	4.7	184	6.5	1.073	22.3	39.1
1.20	0.85	0	27	1	30	0	2391	4.4	231	6.2	0.019	16.8	65.9
0.94	0.79	0	30	1	28.5	0	2749.8	4.7	226	5.6	0.016	17.3	43.4
1.06	0.60	0	27	6	29.5	2	1289.6	4.3	181	5.7	7.462	9.4	61.8
1.10	0.71	0	16	0	26	3	1808.1	4.5	183	5.5	<0.012	19.6	52.8
0.81	0.65	0	24	2	28	1	3850.9	4.5	142	5.5	0.097	20.6	24.3
1.18	0.72	0	24	1	27	1	2162.6	4.6	248	5.6	0.152	20.9	44.4
1.00	0.77	0	28	0	28	0	1540.2	4.5	213	6	0.023	15.2	33.5
		0	29	0	26.5	0	1579	4.3	167	5.7	0.141	24.7	38.6
1.24	0.99	0	29	0	27.5	0	1508.9	4.4	172	5.6	0.02	21.4	33.4
1.56	1.24	0	29	0	30	0	2230.2	4.6	158	5.6	0.059	29.4	26.9
0.86	0.74	0	29	3	24.5	3	1328.6	4.2	222	5.7	0.042	22.5	82.2
1.10	1.14	0	28	0	29.5	0	1287.8	4.6	223	5.6	0.191	17.8	43.7
1.19	0.99	0	28	0	29.5	0	1548	4.5	182	5.9	0.025	16.8	27.7
0.96	0.81	0	25	2	27	0	1738.6	4.6	220	5.9	0.015	23.2	43.6
0.89	0.71	0	24	6	25	4	1499.1	4.6	187	6.3	0.197	16.6	58.4
0.99	0.70	0	28	2	26	1	1601.1	4.5	211	5.7	0.793	24.6	38.3
0.93	0.81	0	24	0	27.5	0	2402.6	4.5	213	6.2	0.064	17.5	32.2
1.10	1.19	0	28	0	29.5	0	2410.7	4.3	165	6	2.161	35.1	22.8
1.36	1.26	0	26	0	29.5	0	2032.4	4.6	226	6	0.105	21.3	40.8
0.92	0.73	0	21	1	28	3	2041.1	4.5	189	7.7	0.164	15.2	106.9
1.03	0.91	0	27	0	28	0	1309.7	4.5	213	5.3	0.033	37.6	31.2
1.38	1.04	0	28	1	29.5	0	1506.5	4.5	235	5.6	0.098	27.8	15.6
1.18	1.16	0	29	0	29	0	2740.8	4.9	156	4.8	<0.012	20.1	39.4
1.12	0.91	0	27	0	29.5	0	1421.4	4.7	231	5.5	0.167	20.2	19.3
0.93	0.81	0	29	0	27.5	2	1803.5	4.5	217	5.4	0.02	37.6	45.3
1.16	0.81	0	30	0	28.5	0	1768.9	4.3	198	6.1	0.016	15.7	67.5
1.10	1.06	0	27	0	28.5	1	2181.6	4.5	205	5.6	<0.012	26.7	76.8
1.24	0.69	0	28	0	27	0	1617.6	4.2	175	5.7	<0.012	24.8	26.8
1.09	0.96	0	27	0	28	0	1629.9	4.5	217	6.2	0.284	19.3	50.4
0.97	0.63	0	22	1	27	2	1987.9	4.7	208	5.3	0.295	19.3	28.7
0.97	0.97	0	28	0	28	0	2092.5	4.3	223	4.5	0.021	27	43.5
1.03	0.88	0	24	1	27	1	1839.1	4.5	174	6.1	0.048	30.9	43
0.99	0.83	0	27	0	27.5	0	1499.5	4.3	232	5.7	0.049	26.4	59.9
1.03	0.96	0	28	0	28	0	1711.3	4.6	144	5.8	0.189	20	47.8
1.00	0.89	0	28	0	29	0	1781.8	4.8	171	5.2	0.052	13.3	75.7
1.08	0.99	0	29	0	28.5	0	2301	4.5	211	5.5	0.276	20.3	64.2
0.79	0.75	0	30	8	28.5	0	1697.9	4.8	122	5.4	0.074	12.9	39.9
1.18	0.75	0	19	1	27.5	2	3282.5	5	236	6.5	0.208	24.4	153.7
0.63	0.68	0	23	3	29.5	2	2359.8	4.3	251	6.4	0.06	20.3	37.3
1.16	0.87	0	27	0	28.5	0	2319.9	4.7	175	5	0.013	30.5	64.6
0.80	0.90	0	30	12	27	2	819.34	4.6	218	5.3	0.836	26.1	49.1
1.26	1.07	0	28	1	27	1	2431.3	4.6	161	6.3	0.134	18.8	37.4
0.91	0.77	0	27	2	26	2	1408.3	4.6	171	6.1	0.033	7.4	27.5
1.05	0.91	0	28	2	25.5	0	1741.5	4.5	217	6	0.045	23	24.8
1.03	1.03	0	26	1	28	0	1933.6	4.8	234	6.2	0.026	19.8	35.2
0.96	0.92	0	29	0	28.5	0	2141.2	4.5	164	5.8	0.117	21.7	47.9
1.31	1.07	0	30	0	27.5	0	1562.2	4.8	180	5.4	0.047	30	45.9
0.78	0.63	0	28	0	26	0	1820.4	4.5	221	5.3	<0.012	17.7	19.2
1.02	0.98	0	30	0	28.5	0	1522.2	4.5	207	5.5	0.022	17.1	34.3
0.97	0.82	0	30	0	27	0	2378	4.7	200	6.3	0.065	18.5	24.3
1.18	1.13	0	29	0	28	2	1480.9	4.5	202	5.7	<0.012	27.4	14.6
0.90	0.94	0	27	0	28.5	0	1570.4	4.8	187	5.4	0.159	12.4	69.9
0.86	0.70	0	28	0	26.5	2	1202.9	4.6	188	5.1	0.056	21.3	62
0.89	0.91	0	22	1	27	0	1222.2	4.6	147	5.5	0.032	19.8	64.4
0.87	0.69	0	26	6	27.5	1	1989.6	4.9	146	6.4	<0.012	21	58.7
0.93	0.78	0	29	2	27.5	0	1308.5	4.8	175	5.1	0.093	24.1	48.8
1.14	1.09	0	29	0	29	1	1968.9	4.8	200	8.1	0.142	17.9	15.1
0.89	0.81	0	24	1	28	0	1608.9	4.5	239	5.9	0.028	13.6	152.5
0.86	0.80	0	29	9	27.5	1	1608.8	4.6	193	6.4	0.096	17.6	48.9
1.09	0.83	0	24	1	28.5	0	2239.4	4.5	198	5.6	<0.012	12.9	22.7
1.04	0.82	0	29	0	27.5	0	1930.6	4.7	145	5.7	0.034	39.4	39.7
1.13	0.83	0	26	0	29.5	0	2280.2	4.4	149	6	0.03	23	26.5
1.02	0.98	0	30	1	28	0	1279.7	4.8	130	4.5	<0.012	29	36.3
1.05	0.91	0	28	0	27.5	1	1440.5	4.3	187	6.2	0.169	24	19.1
1.31	1.01	0	27	0	28.5	1	2180	4.2	149	4.8	0.022	25.6	38.4
1.32	1.05	0	30	0	28	0	2272.9	4.5	173	5.9	0.268	14.5	83.6
1.30	1.13	0	30	0	28.5	0	1871.8	4.5	218	5.4	0.014	30.6	40.5
0.93	0.80	0	28	0	27.5	0	1527.8	4.7	119	4.7	0.022	21.5	26
0.81	0.64	0	29	7	26	0	3424.3	4.9	253		2.713	23.9	20.9

0.83	0.74	-1	25	7	25.5	3	4179.6	4.6	152	8.7	2.008	28.3	18.5
1.02	0.85	0	21	0	28.5	0	2050.4	4.6	197	5.3	0.041	19.9	36.4
1.10	0.90	0	27	1	28	1	2579.7	4.6	245	5.6	0.043	21.6	12.6
1.54	0.92	0	20	8	26.5	2	1629.6	4.7	212	5.4	0.045	29	37.7
0.95	0.94	0	21	3	28	2	2031.9	4.6	151	5.2	0.068	26.5	49.5
0.77	0.63	0	22	0	28	0	2230.9	4.3	240	5.3	0.081	17.3	45.7
1.02	0.82	0	30	0	27	0	2910.4	4.8	186	5.5	0.038	16.4	18.1
0.77	0.83	0	30	0	27.5	0	2469.7	4.6	233	6.5	0.069	17.6	25.5
1.36	0.89	0	29	0	29	2	1658.3	4.4	225	5.6	0.03	23.6	41.3
1.01	0.89	0	29	0	27.5	0	2069.8	4.4	208	5.9	0.054	8.1	55.7
0.98	0.81	0	26	0	27.5	0	1119.5	4.6	176	5.6	0.045	21.2	36.2
0.83	0.69	0	27	5	25.5	0	1700.2	4.4	230	5.9	0.071	17.7	34.9
1.19	0.98	0	29	1	25	0	1658.3	4.4	273	5.9	0.022	25	31.7
0.79	0.73	0	20	2	25	2	1431.2	4.7	251	5.5	1.026	21.3	46.6
1.18	0.77	0	28	0	28.5	0	959	4.7	242	5.6	0.027	18.6	29.1
0.94	0.73	0	30	0	28	0	1611.6	4.9	260	5.7	0.164	16.4	44.4
1.18	1.02	0	28	0	28.5	2	1922.6	5	219	5.7	1.552	25.8	11.6
1.10	0.87	0	30	1	29.5	0	3052.7	4.5	218	5.5	0.047	15.3	36.2
1.14	1.04	0	26	1	28.5	0	2259.8	5	202	5.8	0.014	24.6	53.7
0.98	0.79	0	30	0	26	2	1160.3	4.8	206	5.9	0.032	9.7	40
0.95	0.99	0	25	0	28	0	1269.2	4.8	163	5.5	0.38	23.2	89.1
1.09	1.09	0	26	0	28.5	0	1168.1	4.2	208	5.6	0.246	28.5	46.9
1.10	0.83	0	27	1	28.5	0	1799.5	4.6	280	5.2	0.582	19	66.5
1.18	1.13	0	28	0	29	0	1782	4.5	175	5.7	0.028	23.7	39.7
0.96	0.93	0	30	0	29.5	0	3030	4.7	168	5.9	0.061	17.6	67.3
1.34	0.89	0	25	0	28	5	1187.5	4.5	176	8.6	0.077	28	33.6
0.89	0.73	0	22	0	25	3	2406.4	4.3	200	5.9	2.465	19.1	56
0.91	0.74	0	26	1	23	1	1659.7	4.5	212	6.1	0.026	16.9	28.9
1.24	0.92	0	30	0	28	1	1600.2	4.4	188	5.3	0.06	20.9	30.5
1.10	1.11	0	29	0	29.5	0	1827.4	5.3	185	5.9	0.184	10.6	27.6
0.87	0.73	0	29	1	27.5	1	2068.2	5	195	6.9	0.043	25.8	26.4
1.11	0.82	0	28	3	29.5	1	2432.9	4.7	255	7.2	0.026	18.7	52.4
1.14	0.75	0	28	5	27.5	1	1669.8	4.4	151	6.6	1.181	18	52.5
1.21	0.72	0	28	1	27	0	1299.6	4.5	226	5.5	<0.012	21.5	32.4
0.88	0.70	0	29	0	28.5	1	2871.7	4.3	188	6	0.088	11.2	43.4
1.11	0.83	0	24	0	26	1	3717.6	4.7	141	6.4	0.388	17.9	17.2
1.33	1.04	0	30	0	27.5	0	2546.9	4.7	299	5.7	0.318	15.3	40.2
1.30	0.89	0	29	2	28	0	1858.6	4.5	206	5.9	0.227	13.5	28.6
1.01	0.81	0	22	6	26.5	2	1520.3	4.2	167	5.5	0.351	21.9	39.4
1.26	0.84	0	26	0	28	0	1158.8	4.8	173	5.3	0.025	15	18.5
0.74	0.56	0	18	0	26.5	2	1407.8	4.1	150	5.2	0.056	21.1	36.2
0.91	0.77	0	27	0	26.5	0	2310.9	4.6	149	5.8	<0.012	16.3	37.3
0.96	0.85	0	30	1	26.5	1	770.57	4.1	167	4.6	0.039	18.8	39.2
1.01	0.86	0	29	8	27	1	1230	4.6	224	5.5	1.85	18.3	57.6
0.96	0.81	0	30	1	25.5	2	2828.9	4.2	219	5.7	0.06	24.5	36.9
0.79	0.62	-2	13	1	27	2	2209.7	4.6	178	5.9	0.054	18.8	61.7
0.94	0.73	0	8	0	25	3	1328.4	4.5	143	6.2	<0.012	30.3	53.9
1.26	0.89	0	29	0	28.5	0	2101.9	4.6	195	6	0.023	18.6	42.9
1.03	0.72	0	27	0	30	0	1820.3	4.7	228	5.8	<0.012	23.5	31.6
1.06	0.88	0	30	0	28.5	2	851.15	4.8	179	5.5	0.012	18.9	42.9
0.92	0.88	0	27	0	27	0	2608.3	4.3	189	6	0.039	28.6	18.5
1.05	0.86	0	30	0	29.5	0	1440	4.8	171	5.5	0.113	20.4	52.2
0.97	0.91	0	30	0	25.5	0	2337.7	4.3	215	6.1	0.105	14.6	34.6
1.04	0.83	0	28	0	29	0	2362.4	4.9	192	5.9	0.145	20.7	28.9
0.88	0.78	0	27	1	24	2	1342	4.7	225	6.1	0.162	18.2	48.5
0.96	0.81	0	25	2	27.5	2	2430.4	4.5	189	7.1	0.176	27.3	24.8
1.04	0.90	0	28	0	29.5	0	2050.9	4.4	178	5.7	0.018	35.1	32.2
0.99	0.79	0	29	1	26.5	1	2328.5	4.7	156	7.6	0.077	12.6	11.6
1.41	0.68	0	25	0	27	0	2000.7	4.4	189	6.1	0.091	18.3	30.2
0.91	0.73	0	29	5	24.5	2	2400.1	4.7	128	6	0.067	25.8	22
0.91	0.80	0	27	2	26	1	1841	4.4	126	6.7	0.021	12.2	77.1
1.21	1.01	0	28	1	28	0	1951.6	4.7	214	5.5	0.03	21.7	47.3
0.95	0.86	0	29	0	27.5	0	2936.9	4.9	153	6.3	0.019	22.7	48.2
1.00	0.82	0	23	0	27.5	1	2527.5	4.6	155	10.3	0.028	18.4	46
1.07	0.87	0	30	0	28	1	1869.8	5.1	195	8.8	0.069	23.2	20.6
1.26	0.86	0	27	0	30	0	4380.3	4.6	210	6.3	0.567	22.9	31.1
0.96	0.91	0	19	5	28.5	1	1418.3	4.3	160	6	0.037	35.8	30
1.16	0.82	0	27	0	24.5	1	2201.1	4.5	165	5	0.457	20.4	52.8
1.11	0.86	0	30	0	24.5	2	1289.2	4.2	192	5.6	<0.012	21.5	46.6
1.35	1.10	0	30	1	27.5	0	2557.8	4.7	224	5.8	0.045	22.8	45.5
1.16	1.06	0	30	1	27.5	0	1777.8	4.5	190	5.1	0.014	22	61.8
1.04	0.95	0	27	0	27.5	2	1557.9	4.4	223	5.9	0.014	22.7	37.1

1.02	0.66	0	27	0	28	3	2588.3	4.4	223	5.7	0.073	21.8	37.2
0.93	0.95	0	29	0	27.5	1	1762.2	4.9	160	5.3	0.1	22.8	39.5
1.14	0.89	0	27	0	28	0	1520.4	4.4	182	6	0.211	19.9	87.1
0.91	0.91	0	27	1	27	0	2308.9	4.6	164	5.5	<0.012	21.7	18.5
0.93	0.77	0	27	0	28.5	0	2282.3	4.7	188	6.5	0.017	25.3	43.2
1.14	0.87	0	29	1	30	2	2160.1	4.5	245	7.8	0.221	17.3	40.5
0.90	0.71	0	29	5	26	0	1288.6	4.6	180	5.1	0.021	18	50.1
1.10	0.76	0	29	2	28.5	0	2530.8	4.4	195	6	0.479	20.7	37.8
0.81	0.67	0	30	0	24.5	2	1900.1	4.6	173	5.8	0.467	25.6	27.7
1.24	0.84	0	30	5	29	1	2298.7	4.4	145	5.5	0.053	17.7	44.7
0.85	0.76	0	24	0	25.5	0	998.4	4	159	4.8	0.102	32.9	50.6
0.86	0.72	-15	18	7	26.5	0	1310.5	4.6	162	5.5	0.041	19.3	28.2
1.49	0.95	0	26	0	29	0	2579.9	5	200	5.8	0.067	26.1	44.5
1.02	0.78	0	29	0	28.5	0	2093.5	5.1	249	5.5	0.184	16.5	41.6
1.22	1.17	0	29	1	28	0	1538.7	4.5	202	5.3	0.02	22.9	15.1
1.10	1.13	0	29	0	25.5	3	1470.2	4.6	203	8	0.157	21.9	48.9
1.51	1.01	-2	24	1	25	1	2429	4.4	112	9.1	0.015	25.5	36.7
1.16	0.82	0	27	0	24.5	0	1869	4.5	152	6	<0.012	20.2	37.6
1.15	0.75	0	28	1	26	3	1571.3	4.7	236	6.3	0.051	19.9	45
1.12	0.79	0	29	1	28.5	0	2489.3	4.6	177	5.9	0.013	22.5	45.8
1.24	0.88	0	29	0	28.5	0	1758.2	4.5	264	5.7	0.019	20.2	45.9
1.13	1.15	0	23	1	27.5	0	1940.5	4.6	197	6.3	1.06	21.4	43.5
0.83	0.73	0	29	0	26	0	1819.8	4.6	141	5.4	0.059	14.6	71.9
1.37	1.03	0	30	2	28.5	1	1578	4.1	169	5.5	0.061	18.7	44.5
0.74	0.70	0	22	0	27	2	1687.7	4.7	213	5.7	<0.012	19.4	52.6
0.92	0.75	0	22	0	29	2	1821.2	4.6	193	5.5	0.026	15.9	55
0.82	0.73	0	28	1	27.5	0	1482.2	4.6	160	4.8	0.386	41.3	30.5
1.07	0.70	0	26	0	28	0	1761.8	4.4	213	5.6	0.029	22.3	38.4
0.90	0.72	0	29	2	26	0	1809.6	4.7	153	5.3	<0.012	29.9	24.7
1.06	1.02	0	27	3	28.5	0	2332.2	4.3	189	6	0.027	19.3	33.6
		0	29	0	27	0	909.72	4.4	181	6	<0.012	23.9	16.5
1.60	1.12	0	29	1	27	1	1767.3	4.5	200	6	0.092	28	31.1
1.08	0.80	0	25	2	28.5	0	2278.5	4.3	194	5.6	0.012	31.9	47.2
0.83	0.85	0	28	3	28	0	2692.2	4.5	271	6	0.027	18.8	48.7
0.95	0.90	0	27	0	27.5	0	1749.4	4.6	207	5	0.036	24	53.7
1.24	0.97	0	28	0	28.5	0	2207.4	4.9	228	5.1	0.089	19.4	68
0.71	0.62	0	18	1	24.5	3	1478.5	3.4	156	5.1	0.098	32.4	50.5
1.39	0.85	0	29	6	25.5	4	900.22	3.6	169	6.1	2.644	17.4	79.3
0.87	0.81	0	24	2	26.5	4	1742.6	4.5	168	5.7	0.091	31	28.6
0.95	0.90	0	28	0	27.5	0	1937.5	4.7	215	5.3	<0.012	30.3	27.4
1.04	0.88	0	23	1	28	1	2748.6	4.5	215	9	0.386	16.7	43.2
1.18	0.99	0	28	0	29.5	1	1312.1	4.6	186	5.6	0.128	48.1	41.2
1.19	0.89	0	28	1	28.5	1	1248.6	4.5	204	5.4	0.028	21.4	39.7
1.05	0.80	0	30	0	27	0	2300.4	4.6	207	5.7	0.028	22.4	70.6
0.90	0.90	0	30	0	26	0	1869.6	4.3	172	5.8	<0.012	11	49.7
0.98	1.01	0	28	0	27.5	0	1838.9	4.8	164	5.3	0.036	26.9	53.8
1.08	1.00	0	25	0	29	2	2720.3	4.2	121	5.5	0.225	21.8	22.8
1.21	1.03	0	28	1	28	1	1759.1	4.8	204	6.4	0.261	13.7	58.5
0.66	0.69	0	24	4	30	1	1770.3	5	213	5.7	0.114	21.9	90.1
0.89	0.79	0	27	1	27.5	0	1752.1	4.5	204	5.5	<0.012	42.2	25.7
1.09	0.70	0	29	0	28.5	0	2327.1	4.2	194	6.6	0.102	22.1	29.9
1.16	0.81	0	27	0	28.5	0	1588	5.1	201	5.3	0.036	39.9	69.9
0.72	0.74	0	29	3	25.5	1	2082.3	4.2	157	5.9	<0.012	32.4	26.8
1.05	0.85	0	29	0	30	0	3313.2	4.6	207	5.5	0.043	24.2	26.8
1.09	0.86	0	28	0	26	0	1970.9	4.7	177	5.5	<0.012	31.8	34
1.03	0.98	0	24	0	27.5	1	1238.8	4.3	202	6.2	0.083	28	33.9
0.90	0.62	0	30	0	28	0	2157.8	4.5	228	6	0.057	13.2	21.4
0.72	0.71	0	28	1	30	0	3237.5	4.8	160	5.6	0.284	18.2	24.1
1.10	0.92	0	30	0	28.5	0	1170.9	4.6	208	5.8	0.163	18.4	61.2
1.30	0.89	0	24	1	27	1	2222.3	4.9	209	5.2	<0.012	14.9	64.8
1.36	0.93	0	27	1	27	0	1888.3	4.2	199	5.7	0.038	25.5	42.1
1.17	0.81	0	29	0	27	1	1613.3	4.5	252	5.7	0.129	18.9	64.4
0.79	0.75	0	24	4	26.5	2	1540.4	4.4	223	5.7	0.318	15.5	53.8
1.06	0.85	0	28	0	27	2	2330.2	4.8	156	5.7	<0.012	27	34.6
1.06	0.81	0	28	1	27	0	1611.6	4.7	203	5	<0.012	13.8	56.1
0.93	0.76	0	14	3	27.5	0	2101.6	4.6	203	5.4	0.205	20.4	52.1
1.37	0.93	0	30	0	28.5	0	1687.8	4.5	217	5.4	0.159	18.7	38.1
1.23	0.85	0	24	0	29.5	0	2318.6	4.6	206	5.5	0.136	28.9	54.2
1.15	0.88	0	29	0	26.5	0	1452.1	4.6	132	4.7	0.123	19.8	54
1.20	0.94	0	18	0	27.5	1	2388.2	4.5	179	8.7	0.456	25.2	43.1
0.92	0.77	0	28	7	24.5	0	1692.6	4.8	255	5.8	0.042	24	49.3
1.23	0.96	0	29	2	29	0	1769.7	4.5	179	5.4	0.072	22.6	42.8

0.80	0.68	0	23	1	29	1	1249.3	4.6	164	5.7	0.118	33.2	35.4
0.90	0.88	0	29	1	26	0	1308.8	4.3	204	5.4	0.22	39.5	46.2
1.03	0.70	0	23	4	29	0	2249	4.2	171	6	<0.012	26.1	42.9
0.82	0.68	0	20	1	27	0	2007.4	4.4	207	6.3	0.14	38.5	43.7
1.33	0.80	0	26	4	23	4	2670.8	4.6	175	10.6	0.172	27.3	58.3
1.29	1.00	0	25	4	27.5	1	2317.5	4.5	252	6.7	0.311	11.2	45.8
		0	15	0	27.5	4	2549	4.5	213	5.6	0.046	31.7	33.3
1.30	0.97	0	28	0	29	0	2230.2	4.3	187	5.9	0.043	34.3	41
1.37	1.16	0	29	0	29.5	0	1469.4	4.5	227	6.7	0.147	20.3	8.8
1.20	1.00	0	29	1	28	0	1892.4	4.9	209	5.8	0.069	27.1	45.4
1.30	1.12	0	28	0	28.5	2	1508.7	4.5	201	6	0.132	35.1	37.8
1.24	0.95	0	29	0	27.5	1	1941.1	4.5	214	10.2	0.114	21.6	55.4
1.04	1.00	0	27	10	27.5	0	1930.2	4.5	220	5.9	0.023	22	26.3
0.90	0.78	0	29	1	27.5	0	2716.1	4.3	223	6.4	0.068	16	29.3
0.92	0.64	0	27	3	26	0	1812.4	4.5	206	6.5	0.507	20.9	68.5
1.04	0.76	0	29	3	27.5	0	2382.1	4.1	213	5.6	0.067	13.9	31.9
0.85	0.80	0	28	2	26.5	0	1578.8	4.6	183	6	0.046	16.5	41.2
0.93	0.77	0	18	1	26	3	2203.2	4.6	203	5.9	0.104	18.7	37.3
0.73	0.61	0	20	2	28	3	1292	4.5	152	5.6	<0.012	20.6	48.8
1.00	0.79	0	25	1	27	2	2410.8	4.8	180	5.5	0.085	15.8	42.2
1.18	0.90	-20.5	27	13	26	1	2159.7	4.5	205	6.1	0.064	19.3	31.4
0.80	0.73	0	29	1	26	4	1310.1	4.4	158	6.2	0.022	35.5	71.3
0.90	0.81	0	26	1	27.5	0	1730.4	4.1	194	6.5	0.843	23.7	64.2
0.86	0.72	0	21	2	27	0	1559.5	4.5	235	5.8	0.117	16.1	43.2
1.06	0.85	0	29	0	28.5	0	2257.9	4.6	238	6.3	0.066	19.8	80.5
1.02	0.89	0	25	0	29.5	1	4321.3	4.5	287	6	0.081	14.8	43
1.04	0.64	0	22	3	27	2	1139.5	4.5	169	5	0.261	30.1	53.7
1.08	0.73	0	29	1	25.5	0	1988.3	4.1	194	5.8	0.013	17.9	51.3
1.34	0.87	0	24	1	29.5	0	2552.5	4.4	163	6.2	0.23	27.3	26.1
1.05	0.91	0	29	1	29	3	1711.3	4.5	131	7.4	0.107	35.4	37.8
0.95	0.70	0	25	1	28.5	1	2080.4	4.4	287	9.3	0.144	20.6	13.8
1.16	0.89	0	28	2	29.5	1	2731.3	4.8	187	6.8	0.245	18.8	20
0.96	0.77	0	22	2	21.5	1	2770.6	4.6	175	7.9	<0.012	21.5	29.2
0.87	0.76	0	22	2	27.5	2	1582.6	4.2	194	6.4	0.024	25	45.7
0.88	0.74	0	22	0	25.5	0	2111	4.5	167	5.6	0.196	36.3	33.3
0.83	0.67	0	20	1	27	0	2579.1	4.6	164	6.1	0.056	26.4	39.5
0.96	0.86	0	20	0	29	0	1957.8	4.5	181	5.4	0.016	24.2	35.4
0.83	0.75	0	19	0	29	3	3500.1	4.3	209	9.1	0.157	17.5	48.9
1.05	0.82	0	24	1	27.5	2	928.68	4.5	130	5.6	0.399	37.6	26.9
1.08	0.83	0	19	17	27	1	1931.7	4.8	188	5.9	0.81	23	106.1
1.11	0.93	0	28	0	27.5	0	1214.5	4.7	216	5.5	<0.012	20	36.3
1.03	0.86	0	30	0	28.5	0	1918.1	4.6	190	5.3	0.038	36.6	43.5
1.05	0.81	0	30	0	29	0	1519.9	4.4	221	5.6	0.135	13.4	57.9
1.09	1.02	0	27	2	28	1	2167.1	5	133	8.9	0.07	20.2	24
0.86	0.70	0	27	1	28	0	1059.8	4.7	181	5.5	0.017	20.3	52.9
1.06	0.78	0	28	0	28.5	0	3642.8	4.7	209	5.7	0.021	15.1	56.7
1.30	0.94	0	24	8	23.5	0	2052.2	4.8	214	5.8	0.021	19.3	48.6
1.09	0.86	0	28	0	29.5	0	2630.6	4.9	210	5.8	0.624	41	34
0.99	0.78	0	26	1	29	1	1831.4	4.2	178	8.4	0.116	25.3	34.2
0.87	0.86	0	28	9	26.5	2	2743.5	4.7	209	6.5	0.443	25.7	23.4
1.08	0.96	0	30	1	26.5	1	1576.8	4.9	181	9.8	0.377	17.7	49.1
1.25	0.85	0	28	1	27.5	0	2671.1	4.7	218	6.9	0.458	29.3	36.4
1.06	0.78	0	28	1	26.5	0	2409.2	4.7	238	5.5	<0.012	28.6	34.2
0.96	0.86	0	25	0	28.5	3	1911.4	4.3	148	6.7	<0.012	24.1	31.5
1.12	0.82	0	29	1	28.5	1	1462.8	4.7	159	5.5	0.096	17.2	90.1
0.80	0.65	0	18	5	28	0	1561.4	4.6	186	5.8	<0.012	30.9	40.9
0.79	0.77	0	23	0	29.5	2	2019.6	4.5	208	5.8	0.032	21.7	37.6
0.96	0.82	0	28	0	26.5	0	2261.8	4.4	188	5.8	0.818	24.9	64.4
0.93	0.74	0	26	1	27.5	0	3060	4.6	190	6.3	0.157	22.6	47.6
1.25	0.82	0	27	1	29	0	1500.2	4.4	208	6.5	0.161	13.9	59.1
1.08	0.93	0	28	9	26.5	0	3646.5	5	161	5.7	0.043	44.8	34.6
0.67	0.68	0	19	2	24	3	1848.8	4.5	256	5.7	<0.012	19.3	60.5
1.33	1.10	0	28	0	28	1	1352.1	4.5	161	5	0.044	27.6	44.5
0.92	0.77	-1	22	0	24.5	4	1156.2	4.6	104	6.9	<0.012	21.2	35.1
1.08	0.89	0	23	0	27	3	1429.1	4.3	241	6	0.033	23.6	56.6
1.12	1.08	0	30	0	28.5	0	2483.5	5	189	5.8	0.308	31.8	23.4
1.15	0.88	0	25	1	30	0	2548.8	4.6	227	6.4	0.015	31.1	28.7
1.26	1.08	0	27	2	26.5	0	2140.8	4.5	187	5.8	0.095	32.9	25.4
1.07	0.89	0	29	1	24	0	2690.3	4.7	183	6	0.042	25.3	54
1.01	0.73	0	30	0	27	2	3061.5	4.8	181	7.2	0.015	30.2	62.9
0.93	0.78	0	19	1	27	1	2672.2	4.2	199	7	0.916	28.8	37.5
1.00	0.76	0	22	2	23.5	1	2122.5	4.6	191	4.9	0.024	31	32.5

1.20	1.02	0	27	0	27	1	2537.9	4.4	202	6.7	0.075	24.5	70.6
0.92	0.67	0	18	1	25	2	2208.1	5	171	5.5	0.131	19.9	18.9
1.04	0.84	0	29	0	25.5	0	3050.7	4.3	223	4.6	0.1	26.6	65.2
0.85	0.80	0	29	0	28	0	1750.8	4.9	280	5.9	0.041	27	34
0.91	0.69	0	29	0	25	2	2402.1	4.8	220	5.6	0.018	30.6	22
1.14	0.91	0	29	5	29	1	1582.2	4.7	153	5.5	<0.012	30.9	56
1.17	0.80	0	20	1	27	0	1659.7	4.4	182	6	<0.012	27.7	34
0.71	0.66	0	22	1	28.5	0	3138.8	4.6	204	6	0.305	21.6	39.7
1.00	0.71	0	11	0	28.5	0	2302	4.6	239	6.4	<0.012	17.6	56.5
1.11	0.76	0	30	0	29	0	2729.6	4.8	231	5.8	0.075	21.2	42
1.27	1.19	0	24	3	25.5	1	889.35	4.7	218	5.3	3.303	42.9	22.9
0.97	0.93	0	26	0	29	1	1498	5.1	180	5.8	0.017	43.8	22.5
0.86	1.03	0	29	0	27	1	1799.7	4.6	178	5.7	0.018	29.4	25.2
	0.79	0	26	0	25.5	0	1891.5	4.6	235	5.8	<0.012	18.2	37.5
0.87	0.72	0	15	0	30	0	2540.3	4.7	204	5.8	0.042	17.5	52.3
0.85	0.76	0	27	2	26.5	0	1480.5	4.6	209	6.3	<0.012	18.9	47.4
0.84	0.78	0	28	5	26.5	0	1898.6	4.4	197	5.9	0.252	26.5	30.6
0.80	0.72	0	21	0	28	2	1889.3	4.7	180	5.6	0.016	25	56.3
0.96	0.87	0	23	0	27.5	2	2282.9	4.4	190	6.1	0.114	22.5	67.8
0.72	0.69	0	26	1	19	2	1258.4	4.5	151	5.6	<0.012	15.9	44.3
0.75	0.66	0	14	1	27.5	2	1558.6	4.2	199	5.6	<0.012	14.2	37
0.85	0.73	0	27	2	26	2	1438.1	4.3	211	5.8	0.569	24.4	25.6
1.13	0.94	0	21	0	28.5	0	1068.2	4.5	161	6	1.232	28.4	61.2
1.17	0.81	0	28	2	25.5	2	1892.2	4.2	120	6.4	0.561	41.6	13.2
0.99	0.87	0	20	0	27.5	1	2339.3	4.9	158	5.9	<0.012	43.8	42
1.07	1.06	0	28	0	25.5	0	1532.1	4.4	191	5.3	0.283	25.9	75.8
1.15	0.97	0	29	12	27	0	1310.4	3.6	162	4.5	0.055	38.1	35.8
0.85	0.74	0	28	0	28.5	0	1468.6	4.7	231	5.7	<0.012	19.4	61.8
0.71	0.73	0	25	6	23.5	2	1751.7	4.5	200	5.7	<0.012	26.5	54.2
0.83	0.82	0	21	1	25	0	1887.7	5	136	6	0.029	36.6	23.6
1.11	0.91	0	21	3	27.5	3	1633.6	4.5	154	6.8	2.21	34.2	32.1
0.98	0.85	0	29	0	28.5	1	2241.1	5	144	7.3	0.069	34.4	24.9
1.30	0.87	0	22	0	26.5	0	2157.5	4.7	227	5.8	0.265	31.8	30.9
1.20	0.83	0	28	6	23.5	0	2008	4.8	220	5.9	<0.012	31.6	33.8
1.30	0.93	0	29	0	27	2	1799.5	4.3	120	6	0.042	30.5	73.7
0.91	0.84	0	19	2	28.5	0	3032.8	4.5	206	6	0.076	34.3	30.7
0.98	0.76	0	20	1	27	0	1389.5	4.2	184	6	0.306	25.1	57.3
		0	27	0	25.5	0	2927.5	5	349	5.9	0.122	18.1	20.4
0.98	0.71	0	26	2	29	0	2249.5	4.6	219	7	0.042	17.3	37.7
0.82	0.78	0	26	1	23.5	3	1581.7	5	184	5.9	0.481	22.7	21.9
0.77	0.71	0	18	1	22	3	1732.4	4.4	158	6.8	0.386	18.3	19.3
1.24	0.94	0	19	3	26	2	1729.3	4.5	210	6.3	0.246	29.1	26.8
0.92	0.79	0	23	1	28	2	1738.9	4.5	190	5.9	<0.012	31.1	30.4
1.16	0.73	0	24	0	28.5	0	2528.4	4.7	254	6.6	0.074	24.1	35.5
1.07	0.61	0	21	1	24	0	1368.6	4.7	168	6	<0.012	18.4	51.4
1.00	0.68	0	25	3	26.5	2	2169.4	4.7	198	5.9	0.04	17.4	40.7
0.98	0.76	0	11	2	25.5	3	1699.2	4.5	151	6.3	0.029	27.8	35.7
0.91	0.63	0	20	2	28.5	2	1512	4.7	182	5.3	0.012	20	39.1
1.15	1.10	0	29	1	28	1	2077.6	4.9	227	6.9	0.041	33.2	18
1.36	1.18	0	22	1	27.5	1	2840.2	4.5	190	8.3	0.205	23.2	43.9
1.30	1.04	0	22	2	23.5	3	1701	5	148	9.6	0.026	31.7	183.5
1.10	0.98	0	30	0	27	1	3526.9	5	172	8.3	0.023	31.2	24.3
1.01	0.85	0	28	1	25	0	2401.9	4.6	235	6.2	0.097	21.5	39.4
1.09	0.86	0	27	0	23	0	1642.1	4.6	256	5.8	0.059	23.8	40.7
1.10	0.82	0	27	1	29	1	1928.1	4.9	107	6.1	0.032	17.1	29.5
0.77	0.77	0	22	0	24.5	0	1887.4	4.3	247	5.8	0.635	22.2	56.2
1.43	1.34	0	21	0	27.5	1	1659	4.9	162	8.2	<0.012	33.1	34.4
0.95	0.73	0	27	0	28	1	2088.9	4.8	209	9.6	0.1	22.8	27.4
1.03	1.04	0	28	2	28	0	3075.3	4.4	209	6.1	0.383	27.9	44.4
1.04	0.91	0	30	0	26.5	0	1860.2	4.8	165	5.7	0.05	27.6	29.3
1.03	0.84	0	25	0	28	0	2329.8	4.6	146	5	0.012	24.3	59.3
1.12	0.62	0	29	1	29.5	0	2137.3	4.4	213	5.3	0.046	19.4	32.9
1.23	0.86	0	26	1	28	0	819.55	4.4	179	6.4	0.207	17.2	41.6
1.38	0.81	0	16	0	28	3	2397.1	5	156	6.8	0.406	29.1	40.6
0.86	0.70	0	25	1	27	1	1691.6	4.5	144	5.7	0.045	21.2	33.8
		0	29	1	29.5	0	3030.7	4.4	209	6.1	0.022	24.6	42.2
0.80	0.71	0	16	4	27.5	2	2421.9	4.2	216	6.2	0.066	28.3	51.4
0.94	0.83	0	27	1	26.5	1	2481.5	4.6	221	5.6	0.113	20.9	19.2
0.76	0.76	0	28	5	25	2	1500.9	4.6	218	5.3	0.602	15.7	52.3
	0.70	0	28	0	28.5	2	1669.7	4.3	162	5.5	0.044	31.8	90.6
0.97	0.95	0	21	1	26.5	0	1361.4	4.5	167	6.8	0.109	17.5	42.9
1.10	0.94	0	24	14	24	0	3472	4.4	141	6.4	0.015	39.3	24

1.06	0.71	0	26	11	24.5	3	2179.3	4.4	170	5.8	0.041	16.8	77
1.00	0.73	0	27	0	26	0	2217.1	4.4	191	5.9	0.03	37	35.2
1.04	0.89	0	28	3	25	0	2447.8	4.5	198	5.8	0.046	13.6	40.2
1.07	0.72	0	27	2	22.5	0	2206.6	4.4	171	6.1	0.012	23.5	38.1
0.96		0	30	2	26	1	842.24	4.4	185	5	1.373	18.8	51.7
1.34	1.03	0	28	5	25	1	1652.5	4.6	194	5.9	0.026	30.6	40.6
1.36	0.88	0	29	0	29	0	2058.4	4.7	175	6.2	0.453	20	32.9
1.26	0.78	0	30	0	29	0	1498.1	4.9	179	5.8	0.085	21.2	60.9
1.09	0.79	0	27	0	27	0	1470.6	4.7	172	5.8	0.016	12.4	46.3
1.29	0.93	0	25	0	28	1	3421.7	4.2	195	5.8	0.074	23	21.3
1.08	0.84	0	30	1	26.5	1	2702.7	4.5	161	5.4	0.095	20.8	49.3
0.91	0.67	0	27	0	22	2	2636.7	4.5	180	6.3	0.042	18.1	32.9
1.55	1.33	0	18	0	26	2	1639.3	4.4	179	5.7	0.033	49.8	26.7
0.95	0.95	0	29	0	27	0	2002.8	4.9	236	6.1	0.11	26.1	37.4
0.84	0.75	0	20	0	26.5	1	2396.8	4.8	173	7.2	0.089	27.7	40.1
1.01	0.76	0	28	0	28	0	2609.7	4.7	204	5.8	0.194	19.2	25.6
0.88	0.89	0	24	0	27	0	1339	4.8	202	6	0.024	38.5	13.8
1.08	0.78	0	27	6	26	0	1687.8	5.1	170	6.1	0.024	22.2	29.7
1.22	0.88	0	17	0	28.5	2	1401.6	4.4	183	5.7	0.022	26.9	31.9
0.88	0.67	0	27	0	27	0	1601.1	4.5	207	5.9	0.013	20	39.8
0.93	0.73	0	29	0	25.5	2	1719.8	4.6	210	6.2	0.014	28.3	41.3
0.98	0.82	0	26	7	24.5	2	1971	4.6	235	6.1	0.25	23.4	60.9
1.02	0.86	0	27	1	26.5	2	1307.8	4.6	189	5.4	0.032	32.9	37.5
0.78	0.66	0	21	7	23	3	1408.8	4.5	197	5.6	0.131	23.3	79.5
0.80	0.64	0	26	0	25	0	2178	4.7	223	5.7	0.869	19.9	27
1.19	1.00	0	21	4	27	2	1549.1	4.6	181	5.8	2.148	26.3	25.9
0.86	0.94	0	25	0	25	2	2119.5	4.2	218	6.3	<0.012	16.2	44.3
1.02	0.75	0	25	0	26	0	2491.9	4.6	234	5.9	0.204	27.9	37.9
1.36	1.00	0	29	0	28	0	1841.6	4.7	195	5.8	0.055	37.3	30.8
0.89	0.75	0	30	0	27	0	1752.2	4.5	146	6.1	0.099	24.7	35.7
1.34	1.09	0	29	0	27	0	1909.9	4.6	180	5.7	0.044	30.6	19.2
1.22	0.99	0	26	1	24.5	0	959.37	4.4	166	5.3	0.194	26.4	52.3
1.01	0.92	0	30	0	26	1	2260.1	4.5	219	6.7	0.202	20.4	47.8
0.91	0.63	0	19	1	23	2	1679.6	4.5	165	5.7	0.818	21.8	44
1.18	0.95	0	28	0	29	0	1597.2	4.5	200	6	0.199	32.4	29.3
1.23	0.98	0	30	0	27	0	2140.7	4.6	209	5.7	0.038	20.6	29.2
0.84	0.74	0	16	0	26	0	3489.4	4.9	182	6.4	0.024	21.6	43.3
1.08	1.06	0	25	0	29	1	1382.1	4.7	204	6.7	0.027	14.5	46.2
1.03	0.80	0	26	0	25	1	3483	4.7	128	6	0.16	23.6	79.2
1.19	0.89	0	29	3	24	0	2221	4.7	169	5	0.08	22	121.4
0.95	0.84	0	27	0	29	0	2200	4.7	189	6.1	0.038	25	49.9
1.01	0.81	0	26	0	28.5	1	3490.6	4.5	198	5.9	0.084	19.5	37.8
0.65	0.66	0	28	0	27	0	1868.4	4.3	223	6.1	<0.012	26	50.9
1.31	0.99	0	22	0	29	2	1868.4	4.6	152	7.3	0.031	44	29.8
1.09	0.76	0	26	0	28.5	1	2089.8	4.5	217	5.9	0.08	19.5	73.4
1.28	1.07	0	23	2	28	1	1409.9	4.6	212	7.1	0.351	17.2	52.4
0.84	0.74	0	30	4	27	2	1697.8	4.3	233	5.9	1.013	25.4	32.8
0.62	0.53	-2.5	6	4	25	3	1038.6	4.3	156	6.9	<0.012	21.1	38.8
0.76	0.54	-1	16	0	24	0	1161.3	4.7	176	5.4	0.078	26.9	23.4
0.89	0.83	0	24	0	26.5	0	1570	4.3	166	5.6	1.156	55.2	27.1
0.92	0.74	0	25	2	27	0	2349.5	4.3	156	5.4	<0.012	35	27.7
0.97	0.83	0	26	1	28	3	1861	4.5	182	7.3	0.467	22.7	21.6
0.71	0.62	0	29	0	25.5	3	1478.5	4.4	197	6.4	0.034	17.6	29.3
0.74	0.63	0	29	1	25	3	2672.4	5	230	6	0.048	21.4	22.7
1.11	0.86	0	28	0	29	0	1438.2	4.6	194	5.7	0.018	17.2	59.2
1.06	1.13	0	29	0	29	0	1989	4.5	204	5.9	0.183	21	42.9
1.12	0.98	0	27	0	26	4	1863.8	4.2	118	7.3	0.266	23	34.8
1.08	0.95	0	19	1	28	2	3033.1	4.2	207	6	0.245	30.2	20.5
1.13	0.84	0	27	2	25.5	0	2037.4	4.9	185	6.2	0.99	15.4	42.9
0.75	0.79	0	29	0	26.5	0	2032.2	4.7	144	6.3	0.312	9.9	47.1
		0	22	14	25	2	2552.7	4.3	186	5.7	0.263	21.3	27.1
	0.77	0	25	0	25.5	3	1862.9	4.3	184	5.8	0.07	21.9	42.7
1.20	0.93	0	25	0	27.5	0	2349.9	5.2	213	5.3	0.058	27.4	34.5
0.90	0.73	0	28	1	25.5	1	2803	4.5	247	5.6	0.084	26.2	26.6
1.27	1.13	0	30	0	28	0	2070.3	4.4	192	5.8	0.834	21.3	33.3
0.90	0.70	0	25	1	23.5	0	1250.8	4.7	155	6	0.569	27.7	14.8
1.32	0.90	0	25	1	27	1	2088.5	4.8	194	6.3	0.091	22.4	27.5
0.88	0.67	0	26	0	27	2	1667.8	4.6	181	5.9	0.12	23.5	42
0.85	0.66	0	22	0	26	2	1882.4	4.5	225	5.9	<0.012	13.7	33.7
0.99	0.75	0	27	0	28.5	0	1331.8	4.7	135	6.2	0.162	22.6	35.4
1.00	0.84	0	22	0	27.5	0	2599.1	4.7	256	5.8	0.179	19.3	21
1.33	1.26	0	29	0	27	3	1339.8	4.5	161	4.7	0.038	33.1	45.7

1.02	0.80	0	21	1	28	0	4747.6	4.8	221	5.7	0.07	23.2	33
0.94	0.74	0	16	5	28.5	3	2317.3	4.4	209	7.6	0.036	14.8	40.2
	0.69	0	17	0	28	2	2663.1	4.7	217	6.4	1.173	22.3	28.3
1.24	0.91	0	25	0	29	1	2067.9	4.7	195	8.2	0.028	17.9	16.2
0.83	0.83	0	19	0	25.5	0	2023.5	4.5	177	5.6	0.428	30	77.1
1.12	0.95	0	21	1	29.5	3	1550.3	4.9	162	6.1	0.406	22.3	51.2
0.96	0.88	0	27	1	29.5	0	4020.5	4.7	170	6.7	0.712	17.5	44.6
0.84	0.74	0	29	7	26.5	0	601.44	4.6	177	4.9	0.569	31.1	35.1
0.97	1.02	0	27	1	26.5	0	3197.5	4.9	208	6.4	<0.012	22.8	17.2
0.82	0.63	0	29	0	27.5	0	3318.9	4.5	202	5.8	<0.012	14.6	39.2
1.55	1.16	0	22	0	28.5	1	2441.1	4.5	162	14.6	<0.012	29	48.3
0.83	0.67	0	23	5	25.5	2	1759.9	4.5	190	5.5	0.015	22.4	35
0.88	0.76	0	27	0	26.5	2	869.4	4.5	197	5.8	0.052	26.8	78.3
1.02	0.77	0	27	2	26.5	1	1596.6	4.5	199	6	0.025	25.8	38.4
1.05	0.90	0	20	3	26	2	2328.5	4.5	225	9.7	0.226	17.5	37.5
1.08	0.95	0	25	0	28	0	3403.8	5	301	5.7	0.368	27.3	20.2
1.16	0.81	0	26	1	28	0	1380	4.5	220	6.1	0.058	20.4	54.6
1.75	1.13	0	25	0	29	1	2208.3	4.8	167	6.3	0.131	30	30
1.10	0.94	0	25	1	27	0	2312.5	4.7	249	5.8	0.542	22.5	39.3
1.10	0.98	0	27	0	27.5	1	2221.3	5	172	6.1	0.1	29.3	21.1
0.93	0.72	0	19	7	27.5	1	2898.8	4.9	202	5.9	0.159	23.5	41.3
0.84	0.92	0	21	2	28	0	2208.1	4.3	146	6.2	0.037	20.6	47.9
0.89	0.64	0	25	3	27.5	2	1630.6	4.6	212	6.1	0.061	14.3	31.4
0.91	0.83	0	19	2	29.5	3	2669.3	4.9	235	7.1	0.101	17.1	34.3
0.73	0.73	0	24	1	25.5	0	2068	4.5	152	5.9	0.04	13.2	58.4
0.79	0.60	0	14	1	29	2	2877	4.5	155	6.2	0.377	19.4	57.5
0.86	0.89	0	23	2	28.5	2	1300.5	4.5	218	5.7	0.38	15.1	16.7
1.02	0.74	0	24	2	24	2	2703.1	4.9	223	5.8	0.088	28.6	43
0.88	0.86	0	29	6	26	1	1662.4	4.8	222	5.2	0.229	28.2	27.3
0.86	0.84	0	30	4	22	0	1639.7	4.6	256	5.6	0.393	17.8	17.9
1.17	0.91	0	27	1	27.5	2	1601.4	4.7	348	5.3	0.512	24.1	40.6
1.25	0.99	0	28	2	28.5	0	2278.2	4.8	164	6	0.085	30.1	24.8
1.12	0.94	0	29	0	28	0	1840.2	4.4	182	5.4	0.049	25.6	33
0.94	0.87	0	28	0	25.5	1	1602.3	4.5	178	5.7	<0.012	22.5	53.2
0.77	0.82	0	28	1	27.5	0	3338.6	4.7	261	6.2	0.102	17.8	32.7
0.82	0.74	0	26	0	28.5	1	1568.5	4.6	197	5.7	0.026	15.8	52.6
0.95	0.81	0	27	0	27.5	3	1431.1	5	214	5.7	0.984	34.2	35.4
1.01	0.80	0	24	0	26.5	0	1862.6	4.8	165	5.5	0.584	24.1	22.7
0.91	0.74	0	29	2	27	3	1761.7	4.7	205	7.4	<0.012	26.6	35.6
0.89	0.80	0	20	0	26.5	3	1238	4.3	211	5.3	0.084	22.8	42.5
1.19	0.82	0	20	1	28.5	0	2422.7	4.4	226	5.3	0.518	22.2	26.4
0.84	0.73	0	29	0	29.5	0	2320.7	4.5	235	6.4	0.108	26.2	20.9
1.19	0.95	0	26	0	29	0	841.5	4.8	188	4.9	0.034	19.9	37.5
1.10	0.88	0	28	0	27.5	0	2580.5	4.8	281	5.8	0.019	16	37.2
0.80	0.62	0	28	1	26.5	3	910.14	4.6	143	5.9	0.091	17.5	57.5
1.73	0.80	0	26	0	27.5	2	1550.3	4.5	186	5.8	0.419	14.4	61.4
0.87	0.60	0	25	4	26.5	0	3283.1	4.4	228	5.3	0.052	6.9	23.7
1.01	0.71	0	17	9	27.5	1	1221.1	4.5	188	5.9	<0.012	20.8	42.6
0.94	1.02	0	28	0	29	0	2032	4.6	238	5.4	0.217	26.3	27.8
0.93	0.89	0	27	1	29	0	2258	4.6	159	5.8	0.029	16.7	53.7
1.12	1.03	0	28	0	28	3	1619.1	4.7	155	6.9	1.294	23.1	25.4
0.86	0.73	0	25	1	27.5	0	3012.9	4.5	253	6.1	0.166	20.7	29.8
0.85	0.68	0	20	0	27.5	3	2606.4	4.4	140	8.8	0.094	20.3	38.9
1.12	0.96	0	24	0	19.5	4	1338.5	4.3	172	4.3	0.026	17.2	22.3
1.16	0.89	-9	28	5	26	1	2327.5	4.6	184	8.9	0.199	6.7	46.5
1.23	0.96	0	25	7	29	0	880.77	4.5	144	5	<0.012	16.1	41
1.33	1.02	0	30	4	25.5	0	1038.9	4.9	198	4.8	<0.012	16.8	36.3
1.09	0.78	0	30	0	28.5	0	1931.2	4.7	247	5.8	0.014	19	22.7
0.87	0.79	0	27	5	26	1	2741.2	5	184	6.3	<0.012	16.5	20.5
0.91	0.69	0	30	4	28	0	1499.4	5	192	5.6	<0.012	7.2	25.9
1.10	0.91	0	26	5	26.5	0	2099.9	4.9	162	5.5	0.018	22.4	51.3
1.30	0.84	0	29	0	28.5	0	1838.3	4.5	199	5.7	0.014	16.9	45.1
1.05	0.86	0	30	2	27	0	2800.2	4.8	250	6.6	0.182	24.7	31.6
1.01	0.96	0	26	0	29	0	3232	4.4	225	5.7	0.295	24	35.7
1.13	0.88	0	28	0	27	0	1958	5	193	5.2	0.137	26.3	20.6
0.96	0.81	0	30	0	27	0	1598.2	4.5	200	5.7	<0.012	13.6	35.8
1.19	1.02	0	25	2	28	2	3830	4.7	185	6.3	0.236	15.4	55.4
1.24	0.96	0	28	2	28	0	1870.2	4.7	143	6.3	0.022	14.6	28.9
1.08	1.00	0	28	0	27	0	1591.4	4.6	200	5.3	0.049	22	22.4
0.78	0.67	0	28	0	25.5	1	1200.4	4.7	181	5.4	<0.012	13.4	48.3
1.29	0.92	0	28	1	28.5	0	1877.6	4.6	194	5.5	0.016	16.2	19
1.08	0.92	0	19	25	23	1	1930	4.4	176	5.8	0.985	14.3	35.1

0.89	0.81	0	22	2	29.5	0	1540	4.7	163	5.2	0.108	14.2	60.6
1.23	1.07	0	28	0	29	0	2349.5	4.4	221	5.7	0.077	20.1	56.8
1.31	0.99	0	29	1	25.5	0	3454.3	3.9	146	5.8	0.17	5.7	123.3
0.96	0.87	0	20	1	29	1	3261.9	4.4	259	11.2	1.78	13.1	29.8
0.96	0.83	0	28	2	27	0	1071.5	4.6	239	5.4	0.034	18	51.1
1.12	0.83	-1.5	23	0	28	1	2131.6	4.3	156	5.9	0.18	23.2	59.4
1.42	0.95	0	30	0	29	0	1570.8	4.8	222	6	0.089	18.2	50.3
1.12	0.98	0	29	0	27.5	1	1767.6	5	127	5.5	0.566	20.9	22.2
1.24	0.98	0	29	0	30	0	1868.1	4.7	183	5.4	0.027	25.4	30.7
1.17	0.87	0	29	0	28	0	1389.2	4.7	181	4.9	<0.012	13.2	61.2
1.11	0.82	0	29	1	25	0	1400.4	4.5	227	5.5	0.017	19.4	61.9
0.92	0.95	0	29	0	28.5	0	1689.8	4.4	240	5.4	0.102	19.6	52.7
1.16	0.83	0	29	2	27.5	0	1863	4.4	266	6.5	0.38	14.7	29.7
0.84	0.84	0	29	3	26.5	4	1610.3	4.5	235	5.5	<0.012	17.7	54.6
0.95	0.64	0	27	1	27	3	1878.8	4.5	139	7.1	0.145	14.4	72.6
1.11	0.79	0	26	0	30	0	2619.8	4.4	209	5.7	0.25	15.4	16.2
0.90	0.90	0	28	0	25.5	0	1449.3	4.5	216	5.6	<0.012	25.9	30.7
0.98	0.93	0	30	0	28.5	0	2741	4.8	158	5.5	<0.012	27.3	36.7
1.29	1.11	0	28	0	28	0	1930.5	4.3	204	6.2	0.04	19.2	110.2
0.93	0.69	0	29	0	30	0	1979.8	4.5	202	5.4	0.166	22.6	39.9
0.96	0.81	0	28	0	29	0	1468.2	4.2	184	5.7	0.076	19	57.9
1.19	1.10	0	29	2	27.5	0	1539.5	4.7	247	5.7	1.024	13.5	47.6
1.07	1.11	0	28	1	26	0	1088.2	4.4	231	5.8	<0.012	19.8	59.6
0.89	0.82	0	29	0	29	0	2813.1	5	211	4.9	0.044	18.9	32.7
1.18	1.09	0	26	0	28.5	0	2629.4	4.7	150	6	0.018	16.5	67.2
1.27	0.98	0	29	0	28.5	0	2341.4	4.3	185	5.2	0.041	24	21.6
1.37	1.02	0	28	3	24.5	2	1261.5	4.2	199	5.6	0.023	20.5	50.9
1.00	0.67	-3	27	3	28	4	1331.7	4.7	232	5.8	0.471	19.4	306.7
0.91	0.70	0	29	0	27.5	0	1678.7	4.5	166	5.8	0.089	16.5	22.5
0.82	0.60	0	29	0	22	2	1149.3	4.3	147	5.1	<0.012	4.2	1534.8
1.05	0.87	0	29	2	28.5	0	3020.7	4.9	248	5.9	0.077	18.7	47.9
1.05	0.91	0	30	0	27	0	1510.3	4.6	180	5.3	<0.012	27.6	32
1.10	0.93	0	25	1	26.5	2	2102.6	4.5	167	5.3	2.411	32.7	2.5
1.23	1.03	0	28	0	27.5	2	2180	4.6	172	5.3	0.052	17	58.1
0.83	0.74	0	25	0	21.5	0	2158.2	4.5	222	6.6	0.142	17.1	99.8
1.21	0.95	0	27	0	28.5	0	1940.5	4.6	151	5.4	0.071	22.2	50.4
0.78	0.63	0	30	1	22.5	0	1330.6	4.5	215	5.1	<0.012	20	24.8
1.06	0.89	0	29	1	28	0	1668.6	4.6	201	5.3	0.096	19.3	31.9
1.13	1.01	0	30	1	28	1	1398.7	4.4	170	5.9	0.03	17.5	59.3
0.95	0.92	0	30	1	26.5	0	1489.2	4.6	173	5.8	0.145	13.9	18.6
